# Supplementary material for: The combination of asymmetric hydrogenation of olefins and direct reductive amination
Source: Nat Commun. 2020 Jan 30;11:621. doi: 10.1038/s41467-020-14475-x (PMC6992772; doi:10.1038/s41467-020-14475-x)
Supplement: Supplementary file 2 — Supplementary Information [file 41467_2020_14475_MOESM2_ESM.pdf]

Supplementary Information

The Combination of Asymmetric Hydrogenation of Olefin and Direct Reductive Amination

Yuan et al.

Supplementary methods

General remarks. All reactions were performed in the nitrogen–filled glovebox or under nitrogen using standard Schlenk techniques unless otherwise noted. Column chromatography was performed using silica gel 60 (200–300 mesh). ^{1}H NMR, ^{13}C NMR spectral data were obtaineded from Bruker 500 MHz or Bruker AVANCE III HD (400 MHz) spectrometers. Chemical shifts are reported in ppm. Enantiomeric excess values were determined by chiral HPLC on an Agilent 1220 Series instrument. All new products were further characterized by HRMS. A positive ion mass spectrum of sample was acquired on a Thermo Scientific LTQ Orbitrap XL mass spectrometer.

General procedure A for the synthesis of substrate:

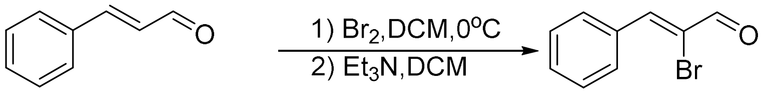

Supplementary Figure 1. Bromination of the α,β-unsaturated aldehyde.

Step 1: To a solution of cinnamaldehyde (10.0g, 75.5 mmol) in DCM (100 mL) was added Br_2 (4.7 mL, 91.5 mmol, 1.2 equiv.) at 0 °C. The reaction mixture was stirred for 15 min, followed by the addition of Et_3N (18.0 mL, 129 mmol, 1.7 equiv.). After stirring for an additional 15 min, the reaction mixture was diluted with DCM and washed sequentially with a 10% $NaHSO_3$ solution, H_2O, and brine. The organic layer was separated and dried over anhydrous Na_2SO_4, filtered, and concentrated to yield orange oil. After keeping for 3 days at room temperature, the mixture crystallized completely as a bright yellow solid which was confirmed to be the desired (*Z*)–2–bromo–3–phenylacrylaldehyde (15.6 g, 98% yield).[1]

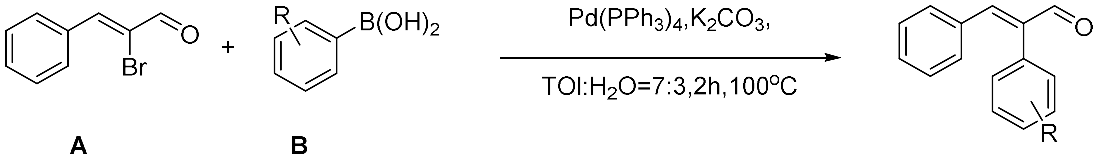

Supplementary Figure 2. Synthesis of 2,3-diphenyl acrolein and its derivatives.

Step 2: A mixture of A (10 mmol), B (1.1 equiv, 11 mmol), $Pd(PPh_3)_4$ (2%,0.2 mmol), and K_2CO_3 (4 equiv, 40 mmol) in toluene:H_2O (7:3, 25 mL) was heated at 100 °C for 2 h. The reaction mixture was passed through a plug of Florisil® eluting with hexane:AcOEt = 5:1, and then concentrated under reduced pressure. The residue was purified by column chromatography on silica gel (hexane:AcOEt = 15:1) to afford **1a–1q**.[2]

General procedure B for the synthesis of substrate:

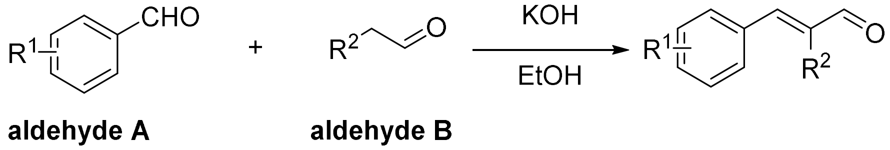

Supplementary Figure 3. Synthesis of 2,3-diphenyl acrolein and its derivatives.

To a stirred solution of KOH (0.56g, 0.01 mol) in EtOH (25 mL, 95%) was added aldehyde A (0.01 mol), the mixture was cooled to 0 °C and aldehyde B (0.02 mol) was added slowly so that the reaction temperature did not exceed 10 °C. After being stirred for 6 h, the reaction was quenched by addition of HCl (25 mL, 3 M, aq.) and extraction with Et_2O (3×20 mL). The organic layer was dried over $MgSO_4$ and concentrated to give the crude product as a color residue. The crude product was purified by column chromatography on silica gel (petroleum ether/EtOAc = 9/1) to afford substituted cinnamyl aldehydes **1r–1za**.[3]

Known Compounds:**1a**[4], **1b**[5], **1c**[6], **1e**[7], **1g**[8], **1k**[8], **1l**[8], **1r**[9], **1s**[4], **1u**[4], **1v**[4], **1za**[10].

(*E*)-2-([1,1'-biphenyl]-4-yl)-3-phenylacrylaldehyde (1d): White solid; ^{1}H NMR (400 MHz, $CDCl_3$): δ 9.72 (s, 1H), 7.69–7.49 (m, 4H), 7.44–7.35 (m, 2H), 7.34 (s, 1H), 7.32–7.11 (m, 8H); ^{13}C NMR (100 MHz, $CDCl_3$): δ 192.94, 149.35, 140.33, 140.00, 139.53, 132.98, 131.14, 129.72, 129.26, 128.82, 128.79, 127.78, 127.53, 126.49, 126.45, 126.05. HRMS (ESI) m/z calcd for $C_{21}H_{17}O^+$ $(M+H)^+$ 285.12739, found 285.12762.

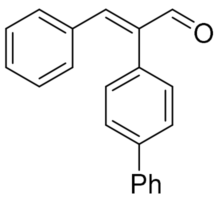

(*E*)-2-(4-chlorophenyl)-3-phenylacrylaldehyde (1f): White solid; ^{1}H NMR (400 MHz, $CDCl_3$): δ 9.75 (s, 1H), 7.72–6.90 (m, 10H); ^{13}C NMR (100 MHz, $CDCl_3$):δ 192.46, 149.72, 139.51, 133.34, 132.66, 130.58, 129.85, 129.64, 129.44, 128.12, 127.62. HRMS (ESI) m/z calcd for $C_{15}H_{12}ClO^+$ $(M+H)^+$ 243.05712, found 243.05711.

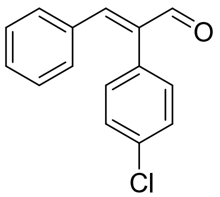

(*E*)-2-(3-methoxyphenyl)-3-phenylacrylaldehyde (1h): White solid;^{1}H NMR (400 MHz, $CDCl_3$): δ 9.68 (s, 1H), 7.30 (s, 1H), 7.29–7.11 (m, 6H), 6.92–6.79 (m, 1H), 6.72–6.67 (m, 1H), 6.66 (dd, *J* = 2.6, 1.4 Hz, 1H) , 3.69 (s, 3H); ^{13}C NMR (100 MHz, $CDCl_3$): δ 192.74, 158.91, 149.05, 140.60, 133.69, 132.88, 129.77, 129.27, 128.98, 127.49, 120.50, 113.53, 113.07, 54.19. HRMS (ESI) m/z calcd for $C_{16}H_{15}O_2^+$ $(M+H)^+$ 239.10666, found 239.10670.

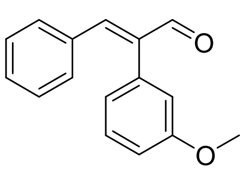

(*E*)-*N*-(3-(3-oxo-1-phenylprop-1-en-2-yl)phenyl)acetamide (1i): White solid; ^{1}H NMR (400 MHz, $CDCl_3$): δ 9.74 (s, 1H), 7.92 (s, 1H), 7.64 (d, *J* = 2.2 Hz, 1H), 7.40 (s, 1H), 7.36–7.17 (m, 7H), 6.85 (d, *J* = 7.5 Hz, 1H), 2.00 (s, 3H); ^{13}C NMR (100 MHz, $CDCl_3$): δ 194.52, 168.60, 151.20, 141.29, 139.03, 133.88, 133.67, 130.94, 130.58, 129.63, 128.60, 124.50, 120.10, 119.80, 24.44. HRMS (ESI) m/z calcd for $C_{17}H_{16}NO_2^+$ $(M+H)^+$ 266.11756, found 266.11777.

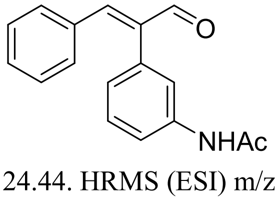

(*E*)-2-(3,5-dimethylphenyl)-3-phenylacrylaldehyde (1j): White solid; ^{1}H NMR (400 MHz, $CDCl_3$): δ 9.75 (s, 1H), 7.34 (s, 1H), 7.32–7.19 (m, 5H), 7.01 (s, 1H), 6.79 (s, 2H), 2.30 (s, 6H); ^{13}C NMR (100 MHz, $CDCl_3$): δ 194.22, 149.77, 142.13, 138.41, 134.12, 133.24, 130.84, 130.18, 130.04, 128.46, 126.69, 21.34. HRMS (ESI) m/z calcd for $C_{17}H_{17}O^+$ $(M+H)^+$ 237.12739, found 237.12732.

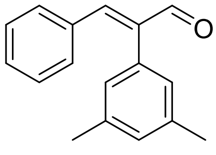

(*E*)-2-(2-fluorophenyl)-3-phenylacrylaldehyde (1m): Pale yellow solid; ^{1}H NMR (500 MHz, $CDCl_3$): δ 9.77 (s, 1H), 7.54 (s, 1H), 7.45–7.36 (m, 1H), 7.35–7.29 (m, 1H), 7.27–7.10 (m, 7H); ^{13}C NMR (125 MHz, $CDCl_3$): δ 192.85, 160.85, 158.88, 151.59, 136.11, 133.91, 131.36, 130.66, 130.41, 128.71, 124.60, 124.58, 121.37, 116.23. HRMS (ESI) m/z calcd for $C_{15}H_{12}FO^+$ $(M+H)^+$ 227.08667, found 227.08652.

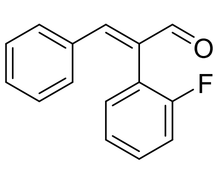

(*E*)-2-(2-chlorophenyl)-3-phenylacrylaldehyde (1n): Pale yellow solid; ^{1}H NMR (400 MHz, $CDCl_3$): δ 9.76 (s, 1H), 7.53 (s, 1H), 7.51 (dd, *J* = 7.9, 1.4 Hz, 1H), 7.40–7.28 (m, 3H), 7.28–7.21 (m, 2H), 7.19–7.09 (m, 3H); ^{13}C NMR (100 MHz, $CDCl_3$): δ 191.71, 149.72, 138.47, 132.74, 132.52, 132.10, 129.89, 129.63, 129.48, 128.94, 128.85, 127.69, 126.33. HRMS (ESI) m/z calcd for $C_{15}H_{12}ClO^+$ $(M+H)^+$ 243.05712, found 243.05714.

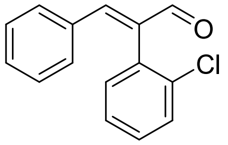

(*E*)-2-(naphthalen-2-yl)-3-phenylacrylaldehyde (1o): White solid; ^{1}H NMR (400 MHz, $CDCl_3$): δ 9.84 (s, 1H), 7.95–7.78 (m, 3H), 7.75 (s, 1H), 7.57–7.47 (m, 2H), 7.46 (s, 1H), 7.33–7.13 (m, 6H); ^{13}C NMR (100 MHz, $CDCl_3$): δ 193.95, 150.38, 141.51, 133.97, 133.50, 133.07, 130.79, 130.70, 130.30, 128.75, 128.54, 128.51, 128.26, 127.75, 127.02, 126.41, 126.13.. HRMS (ESI) m/z calcd for $C_{19}H_{15}O^+$ $(M+H)^+$ 259.11174, found 259.11185.

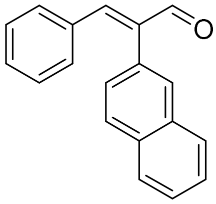

(*E*)-2-(naphthalen-1-yl)-3-phenylacrylaldehyde (1p): White solid; ^{1}H NMR (400 MHz, $CDCl_3$): δ 9.92 (s, 1H), 7.96–7.87 (m, 2H), 7.70 (s, 1H), 7.61 (d, *J* = 1.1 Hz, 1H), 7.56–7.40 (m, 2H), 7.42–7.32 (m, 1H), 7.28 (dd, *J* = 7.1, 1.2 Hz, 1H), 7.25–7.17 (m, 1H), 7.15–6.98 (m, 4H); ^{13}C NMR (100 MHz, $CDCl_3$): δ 192.94, 150.14, 139.42, 132.80, 132.76, 130.69, 130.07, 129.81, 129.43, 127.82, 127.57, 127.54, 125.97, 125.50, 125.16, 124.79, 123.81. HRMS (ESI) m/z calcd for $C_{19}H_{15}O^+$ $(M+H)^+$ 259.11174, found 259.11194.

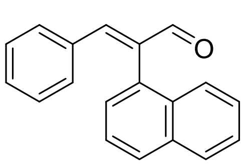

(*Z*)-3-phenyl-2-(thiophen-2-yl)acrylaldehyde (1q): Brown solid; ^{1}H NMR (400 MHz, $CDCl_3$): δ 9.73 (s, 1H), 7.44 (s, 1H), 7.43 (dd, *J* = 4.9, 1.4 Hz, 1H), 7.40–7.26 (m, 5H), 7.12–7.02 (m, 2H); ^{13}C NMR (125 MHz, $CDCl_3$): δ 191.77, 149.96, 133.64, 132.93, 131.66, 129.41, 129.38, 127.58, 127.39, 126.48, 126.22. HRMS (ESI) m/z calcd for $C_{13}H_{11}OS^+$ $(M+H)^+$ 215.05251, found 215.05241.

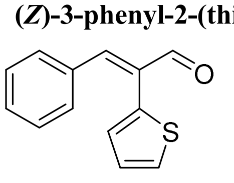

(*E*)-3-(4-(tert-butyl)phenyl)-2-phenylacrylaldehyde (1t): White solid; ^{1}H NMR (400 MHz, $CDCl_3$): δ 9.67 (s, 1H), 7.39–7.31 (m, 3H), 7.28 (s, 1H), 7.21–7.11 (m, 4H), 7.10–7.04 (m, 2H), 1.19 (s, 9H); ^{13}C NMR (100 MHz, $CDCl_3$): δ 194.03, 154.02, 150.25, 141.08, 133.71, 131.18, 130.77, 129.28, 128.88, 128.20, 125.55, 34.88, 31.02. HRMS (ESI) m/z calcd for $C_{19}H_{21}O^+$ $(M+H)^+$ 265.15869, found 265.15881.

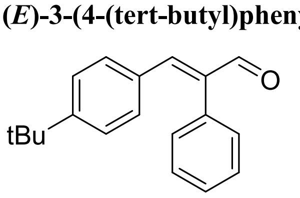

(*E*)-2-phenyl-3-(3-(trifluoromethyl)phenyl)acrylaldehyde (1w): White solid; ^{1}H NMR (400 MHz, $CDCl_3$): δ 9.81 (s, 1H), 7.59–7.50 (m, 1H), 7.48–7.38 (m, 5H), 7.37–7.30 (m, 2H), 7.24–7.12 (m, 2H); ^{13}C NMR (125 MHz, $CDCl_3$): δ 192.40, 146.55, 142.17, 133.73, 132.37, 131.50, 128.10, 128.03, 127.96, 127.71, 126.31 (q, *J* = 4.0 Hz), 125.44 (q, *J* = 3.7 Hz), 123.87, 121.16. HRMS (ESI) m/z calcd for $C_{16}H_{12}F_3O^+$ $(M+H)^+$ 277.08348, found 277.08362.

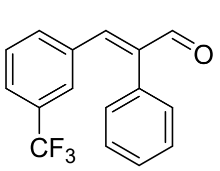

(*E*)-3-(3,5-dichlorophenyl)-2-phenylacrylaldehyde (1x): Pale yellow solid; ^{1}H NMR (400 MHz, $CDCl_3$): δ 9.77 (s, 1H), 7.48–7.40 (m, 3H), 7.27 (t, *J* = 1.9 Hz, 1H), 7.25 (s, 1H), 7.19–7.12 (m, 2H), 7.04 (d, *J* = 1.8 Hz, 2H); ^{13}C NMR (100 MHz, $CDCl_3$): δ 193.18, 145.99, 143.73, 136.84, 135.05, 132.08, 129.66, 129.08, 129.04, 128.95, 128.56. HRMS (ESI) m/z calcd for $C_{15}H_{11}Cl_2O^+$ $(M+H)^+$ 277.01815, found 277.01849.

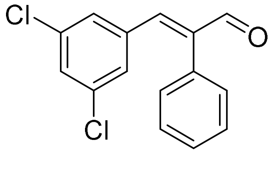

(*E*)-3-(2-chlorophenyl)-2-phenylacrylaldehyde (1y): White solid; ^{1}H NMR (400 MHz, $CDCl_3$): δ 9.86 (s, 1H), 7.72 (s, 1H), 7.44 (dd, *J* = 8.1, 1.2 Hz, 1H), 7.39–7.28 (m, 3H), 7.25–7.09 (m, 3H), 7.00–6.85 (m, 2H); ^{13}C NMR (100 MHz, $CDCl_3$): δ 193.56, 145.99, 143.06, 135.12, 132.60, 132.27, 131.04, 130.71, 129.81, 129.59, 128.63, 128.42, 126.32. HRMS (ESI) m/z calcd for $C_{15}H_{12}ClO^+$ $(M+H)^+$ 243.05712, found 243.05719.

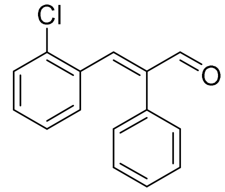

(*E*)-3-(2-fluorophenyl)-2-phenylacrylaldehyde (1z): White solid; ^{1}H NMR (400 MHz, $CDCl_3$): δ 9.81 (s, 1H), 7.64 (s, 1H), 7.47–7.33 (m, 3H), 7.32–7.24 (m, 1H), 7.23–7.16 (m, 2H), 7.14–7.03 (m, 1H), 6.98–6.88 (m, 1H), 6.87–6.80 (m, 1H); ^{13}C NMR (100 MHz, $CDCl_3$): δ 192.57, 161.40, 158.88, 141.87, 131.79, 130.94, 129.41, 129.39, 128.27, 127.78, 122.79, 122.76, 114.86. HRMS (ESI) m/z calcd for $C_{15}H_{12}FO^+$ $(M+H)^+$ 227.08667, found 227.08650.

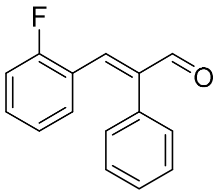

General Procedure for Asymmetric Reductive Amination

In a nitrogen–filled glovebox, $[Rh(COD)Cl]_2$ (5 μmol) and L (11 μmol) was dissolved in anhydrous CH_3COOCH_3 (1.0 mL), stirred for 20 min, and equally divided into 10 vials charged with aldehyde (0.1 mmol) and aniline (0.1 mmol) in anhydrous CH_3COOCH_3 solution (0.5 mL). Then 4Cl–$PhSO_3H$ (0.3 equiv.), $NaSbF_6$ (0.05 equiv), were added and the total solution was made to 2.0 mL (MeOAc:DMF = 4:1) for each vial. The resulting vials were transferred to an autoclave, which was charged with 50 atm of H_2, and stirred at 60 °C for 24 h. The hydrogen gas was released slowly and the solution was quenched with aqueous sodium bicarbonate solution. The organic phase was concentrated and passed through a short column of silica gel to remove the metal complex to give the crude products, which were purified by column chromotography and then analyzed by chiral HPLC determine the enantiomeric excesses.

***N*–(2,3–diphenylpropyl)aniline (3a)**: 98% yield, 98% ee, colorless oil. ^{1}H NMR (500 MHz, $CDCl_3$): δ 7.38 (t, *J* = 7.5 Hz, 2H), 7.34–7.28 (m, 3H), 7.26 (d, *J* = 7.4 Hz, 3H), 7.22 – 7. 12 (m, 4H), 6.76 (t, *J* = 7.3 Hz, 1H), 6.54 (d, *J* = 7.9 Hz, 2H), 3.54 (dd, *J* = 12.2, 5.0 Hz, 1H), 3.39–3.31 (m, 1H), 3.31–3,23 (m, 1H), 3.07 (dd, *J* = 7.3, 3.5 Hz, 2H); ^{13}C NMR (125 MHz, $CDCl_3$): δ 148.04, 142.72, 139.94, 129.27, 129.17, 128.77, 128.40, 127.97, 126.93, 126.25, 117.48, 113.09, 48.58, 47.07, 41.14. HRMS (ESI) m/z calcd for $C_{21}H_{22}N^{+}$ $(M+H)^{+}$ 288.17468, found 288.17487. Enantiomeric excess was determined by chiral HPLC: Chiralpak IA–3 column, Hex/IPA=95:5, 1 mL/min, 220 nm, 6.76 min (minor), 8.53 min (major).

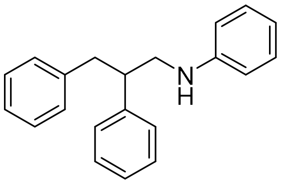

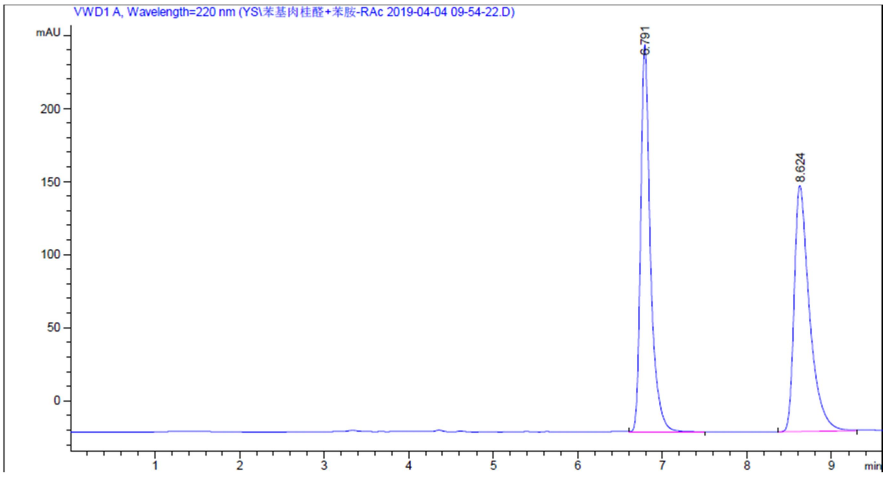

峰 #	保留时间 [min]	类型	峰宽 [min]	峰面积 [mAU*s]	峰高 [mAU]	峰面积 %
1	6.791	VB	0.1198	2128.31982	264.57648	50.1104
2	8.624	BV	0.1868	2118.93872	168.37059	49.8896

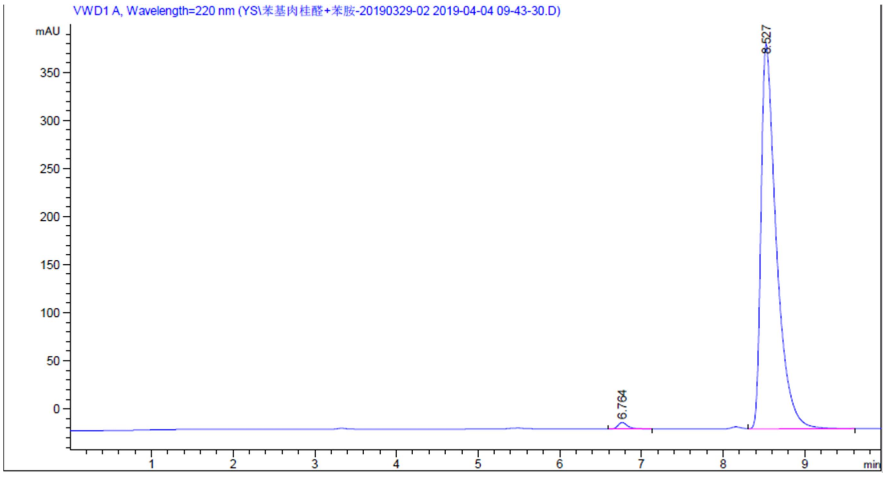

峰 #	保留时间 [min]	类型	峰宽 [min]	峰面积 [mAU*s]	峰高 [mAU]	峰面积 %
1	6.764	BB	0.1235	57.07546	6.90130	1.0796
2	8.527	VV	0.1922	5229.55566	400.91171	98.9204

Supplementary Figure 4. HPLC spectra for racemic and chiral **3a**.

***N*–(3–phenyl–2–(p–tolyl)propyl)aniline (3b)**: 95% yield, 99% ee, colorless oil. ^{1}H NMR (500 MHz, $CDCl_3$):δ 7.25–7.20 (m, 2H), 7.19–7.13 (m, 1H), 7.13–7.03 (m, 8H), 6.65 (t, *J* = 7.3 Hz, 1H), 6.42 (d, *J* = 7.8 Hz, 2H), 3.41 (dd, *J* = 12.1, 4.8 Hz, 1H), 3.55–3.30 (m, 2H), 3.18–3.10 (m, 1H), 2.96 (d, *J* = 7.3 Hz, 2H), 2.31 (s, 3H); ^{13}C NMR (125 MHz, $CDCl_3$): δ 148.14, 140.10, 139.66, 136.43, 129.48, 129.26, 129.19, 128.40, 127.82, 126.22, 117.45, 113.13, 48.67, 46.63, 41.24, 21.18. HRMS (ESI) m/z calcd for $C_{22}H_{24}N^{+}$ $(M+H)^{+}$ 302.19033, found 302.19040. Enantiomeric excess was determined by chiral HPLC: Chiralpak IA–3 column, Hex/IPA=90:10, 1 mL/min, 220 nm, 5.44 min (minor), 6.03 min (major).

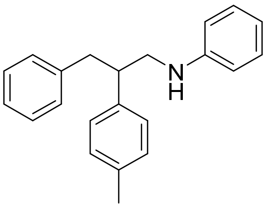

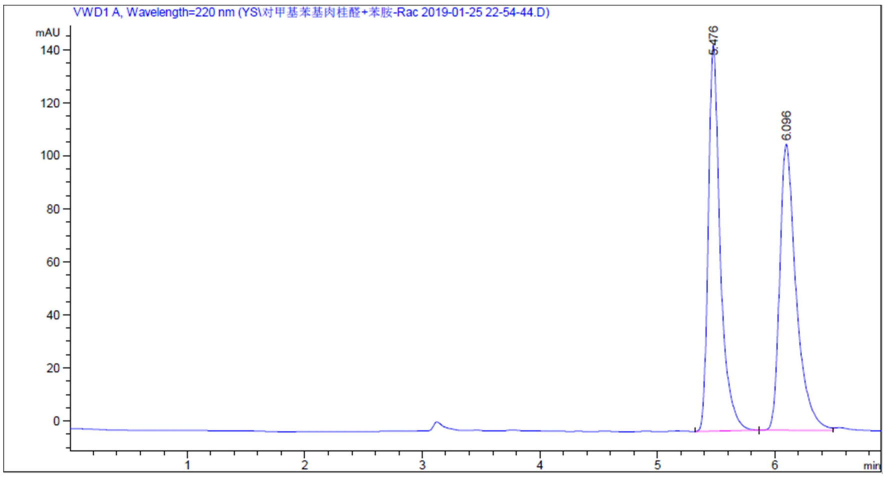

峰 #	保留时间 [min]	类型	峰宽 [min]	峰面积 [mAU*s]	峰高 [mAU]	峰面积 %
1	5.476	BB	0.1055	1029.08850	145.46310	49.5194
2	6.096	BV	0.1445	1049.06323	107.85089	50.4806

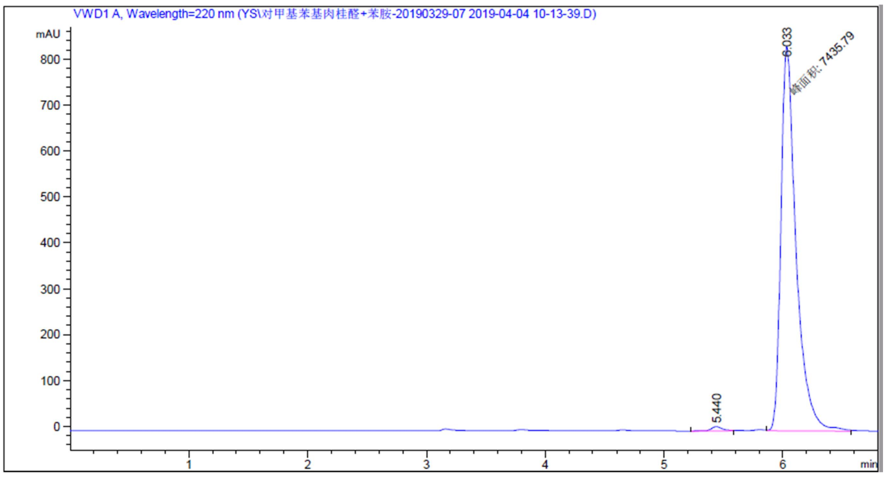

峰 #	保留时间 [min]	类型	峰宽 [min]	峰面积 [mAU*s]	峰高 [mAU]	峰面积 %
1	5.440	BV	0.0951	57.06266	9.10463	0.7606
2	6.033	VB	0.1318	7445.01904	836.95844	99.2394

Supplementary Figure 5. HPLC spectra for racemic and chiral **3b**.

***N*–(2–(4–(tert–butyl)phenyl)–3–phenylpropyl)aniline (3c)**: 97% yield, 97% ee, colorless oil. ^{1}H NMR (500 MHz, $CDCl_3$): δ 7.32 (d, *J* = 8.0 Hz, 2H), 7.27–7.05 (m, 9H), 6.64 (t, *J* = 7.3 Hz, 1H), 6.41 (d, *J* = 7.9 Hz, 2H), 3.47 (s, 1H), 3.40 (dd, *J* = 12.0, 4.5 Hz, 1H), 3.26–3.10 (m, 2H), 3.05–2.90 (m, 2H), 1.31 (s, 9H); ^{13}C NMR (125 MHz, $CDCl_3$): δ149.72, 148.15, 140.24, 139.80, 129.25, 129.22, 128.43, 127.52, 126.25, 125.67, 117.41, 113.13, 48.47, 46.44, 41.16, 34.57, 31.52. HRMS (ESI) m/z calcd for $C_{25}H_{30}N^+$ (M+H)$^+$ 344.23728, found 344.23734. Enantiomeric excess was determined by chiral HPLC: Chiralpak IA–3 column, Hex/IPA=99:1, 1 mL/min, 220 nm, 5.80 min (major), 6.41 min (minor).

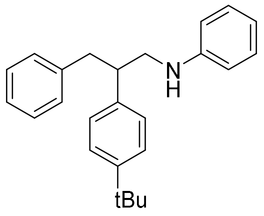

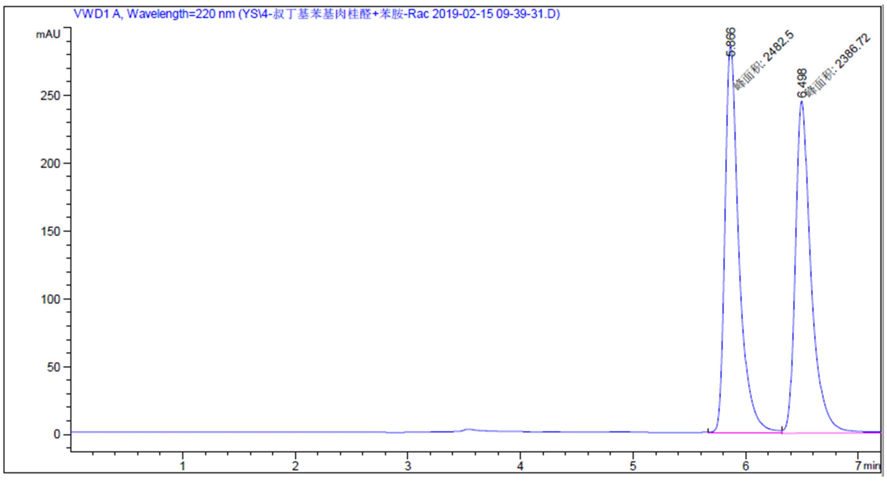

峰 #	保留时间 [min]	类型	峰宽 [min]	峰面积 [mAU*s]	峰高 [mAU]	峰面积 %
1	5.866	MF	0.1450	2482.49634	285.29184	50.9835
2	6.498	FM	0.1625	2386.72168	244.79846	49.0165

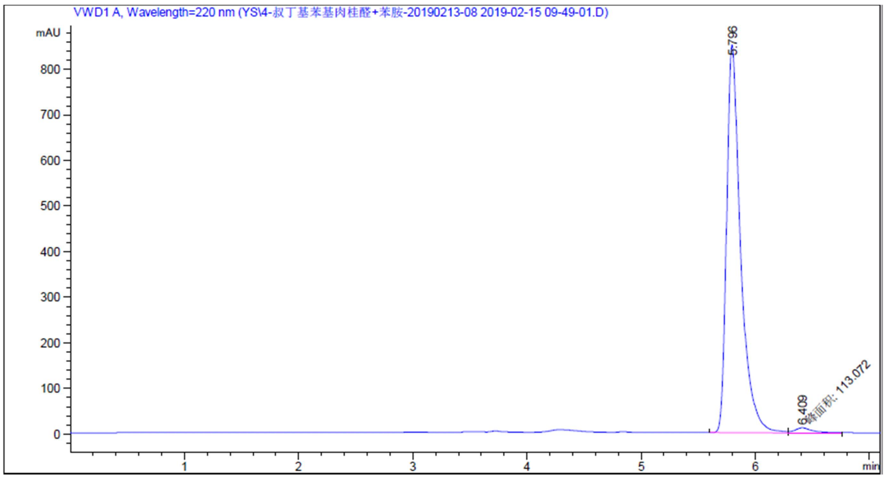

峰 #	保留时间 [min]	类型	峰宽 [min]	峰面积 [mAU*s]	峰高 [mAU]	峰面积 %
1	5.796	BV	0.1274	7246.18359	850.37054	98.4635
2	6.409	MF	0.1739	113.07193	10.83664	1.5365

Supplementary Figure 6. HPLC spectra for racemic and chiral **3c**.

***N*–(2–([1, 1'–biphenyl]–4–yl)–3–phenylpropyl)aniline (3d)**: 94% yield, 97% ee, colorless oil.

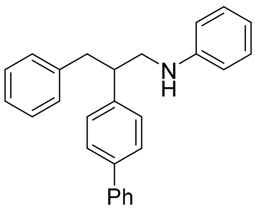

^{1}H NMR (500 MHz, $CDCl_3$): δ 7.58 (d, *J* = 7.7 Hz, 2H), 7.53 (d, *J* = 7.9 Hz, 2H), 7.42 (t, *J* = 7.6 Hz, 2H), 7.35–7.03 (m, 10H), 6.66 (t, *J* = 7.3 Hz, 1H), 6.45 (d, *J* = 8.0 Hz, 2H), 3.47 (dd, *J* = 11.8, 4.5 Hz, 2H), 3.36–3.16 (m, 2H), 3.01 (d, *J* = 7.0 Hz, 2H); ^{13}C NMR (125 MHz, $CDCl_3$): δ148.06, 141.88, 140.89, 139.93, 139.78, 129.32, 129.22, 128.90, 128.47, 128.40, 127.46, 127.34, 127.11, 126.32, 117.55, 113.15, 48.64, 46.76, 41.15. HRMS (ESI) m/z calcd for $C_{27}H_{26}N^{+}$ $(M+H)^{+}$ 364.20598, found 364.20612. Enantiomeric excess was determined by chiral HPLC: Chiralpak IA–3 column, Hex/IPA=90:10, 1 mL/min, 220 nm, 7.21 min (major), 7.76 min (minor).

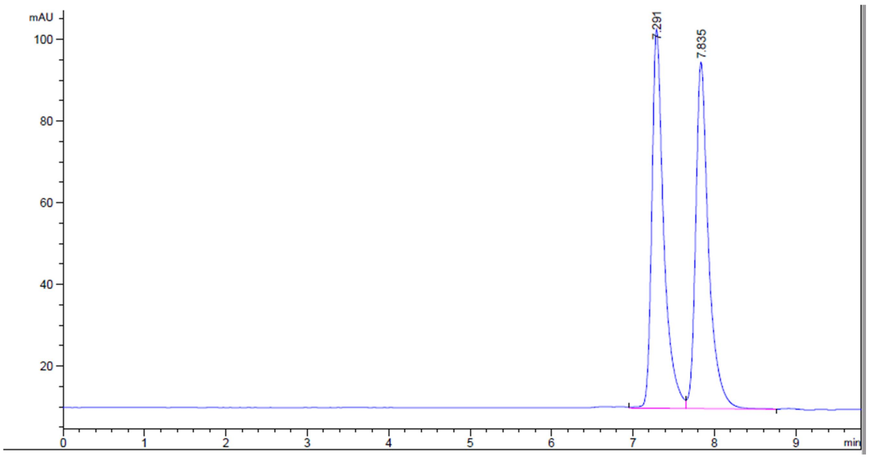

峰 #	保留时间 [min]	类型	峰宽 [min]	峰面积 [mAU*s]	峰高 [mAU]	峰面积 %
1	7.291	VV	0.1507	944.66406	92.81993	50.3038
2	7.835	VB	0.1633	933.25568	84.87425	49.6962

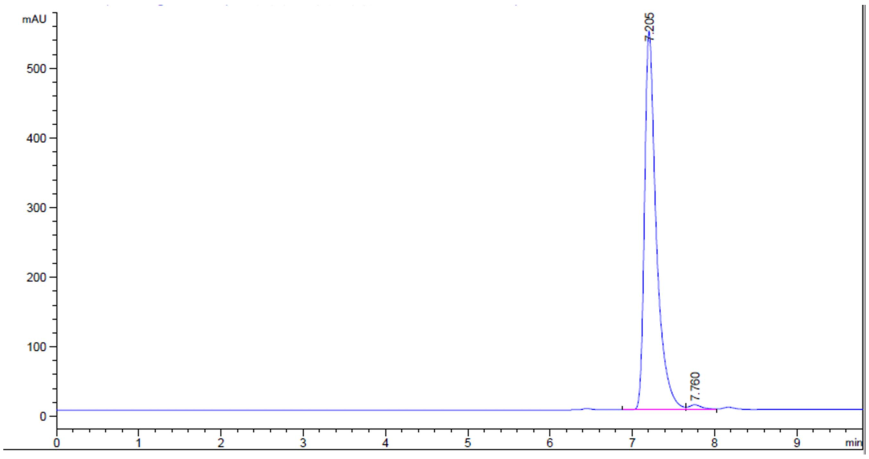

峰 #	保留时间 [min]	类型	峰宽 [min]	峰面积 [mAU*s]	峰高 [mAU]	峰面积 %
1	7.205	BV	0.1484	5425.64746	543.58844	98.5133
2	7.760	VV	0.1673	81.87818	7.05830	1.4867

Supplementary Figure 7. HPLC spectra for racemic and chiral **3d**.

4–(1–phenyl–3–(phenylamino)propan–2–yl)phenol (3e): 92% yield, 97% ee, colorless oil. ^{1}H NMR δ 7.25–7.18 (m, 2H), 7.16 (d, *J* = 7.4 Hz, 1H), 7.14–7.08 (m, 2H), 7.08–7.02 (m, 3H), 7.00 (d, *J* = 8.5 Hz, 2H), 6.74–6.68 (m, 3H), 6.46 (s, 2H), 4.07 (d, *J* = 1.5 Hz, 1H), 3.42 (dd, *J* = 12.1, 4.8 Hz, 1H), 3.19 (dd, *J* = 12.1, 9.1 Hz, 1H), 3.15–3.08 (m, 1H), 2.98–2.86 (m, 2H); ^{13}C NMR (125 MHz, CD_3OHD): δ 153.93, 146.76, 138.76, 132.04, 127.23, 127.04, 127.00, 126.08, 123.84, 115.09, 113.25, 111.23, 47.23, 44.49, 38.93. HRMS (ESI) m/z calcd for $C_{21}H_{22}NO^+$ $(M+H)^+$ 304.16959, found 304.16980. Enantiomeric excess was determined by chiral HPLC: Chiralpak IA–3 column, Hex/IPA = 90:10, 1 mL/min, 220 nm, 16.17 min (major), 20.48 min (minor).

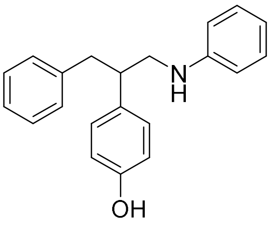

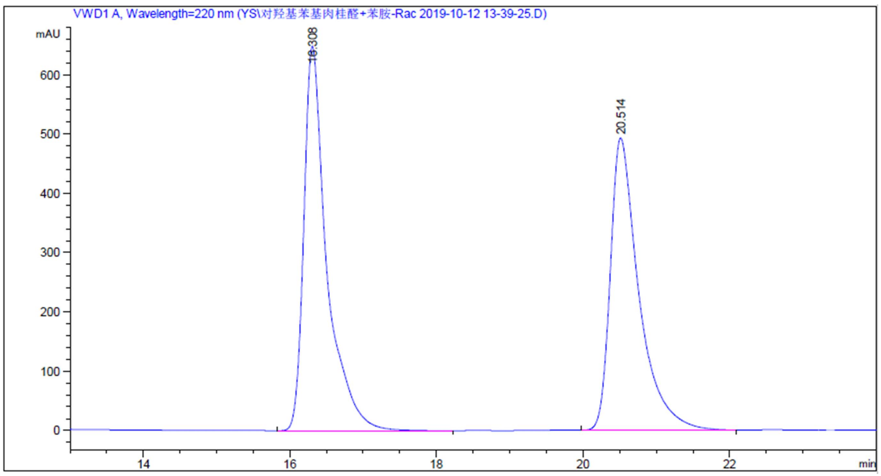

峰 #	保留时间 [min]	类型	峰宽 [min]	峰面积 [mAU*s]	峰高 [mAU]	峰面积 %
1	16.308	BB	0.3157	1.41043e4	648.59125	51.3497
2	20.514	VV	0.3961	1.33628e4	493.23627	48.6503

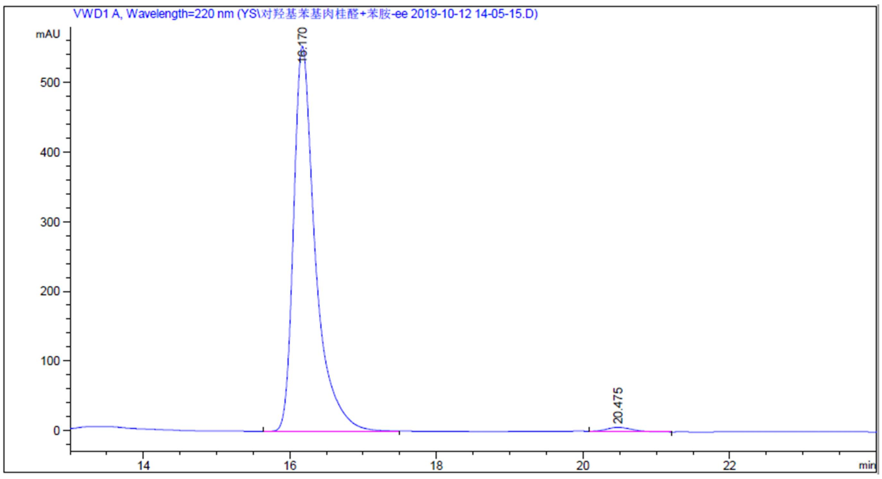

峰 #	保留时间 [min]	类型	峰宽 [min]	峰面积 [mAU*s]	峰高 [mAU]	峰面积 %
1	16.170	BV	0.3073	1.14380e4	553.03314	98.5934
2	20.475	VB	0.3918	163.18629	6.48101	1.4066

Supplementary Figure 8. HPLC spectra for racemic and chiral **3e**.

***N*–(2–(4–chlorophenyl)–3–phenylpropyl)aniline (3f)**: 98% yield, 97% ee, colorless oil. ^{1}H NMR (500 MHz, $CDCl_3$): δ 7.27–6.99 (m, 11H), 6.66 (t, J = 7.3 Hz, 1H), 6.43 (d, J = 8.0 Hz, 2H), 3.43 (dd, J = 12.2, 4.9 Hz, 2H), 3.26–3.11 (m, 2H), 3.02–2.84 (m, 2H); ^{13}C NMR (125 MHz, $CDCl_3$): δ 147.85, 141.18, 139.50, 132.58, 129.34, 129.13, 128.87, 128.46, 126.37, 117.67, 113.11, 48.68, 46.59, 40.99. HRMS (ESI) m/z calcd for $C_{21}H_{21}ClN^+$ $(M+H)^+$ 322.13570, found 322.13586. Enantiomeric excess was determined by chiral HPLC: Chiralpak IB–3 column, Hex/IPA=95:5, 1 mL/min, 220 nm, 9.90 min (minor), 11.04 min (major).

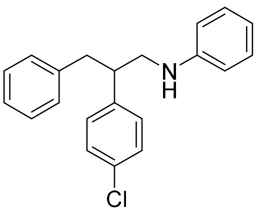

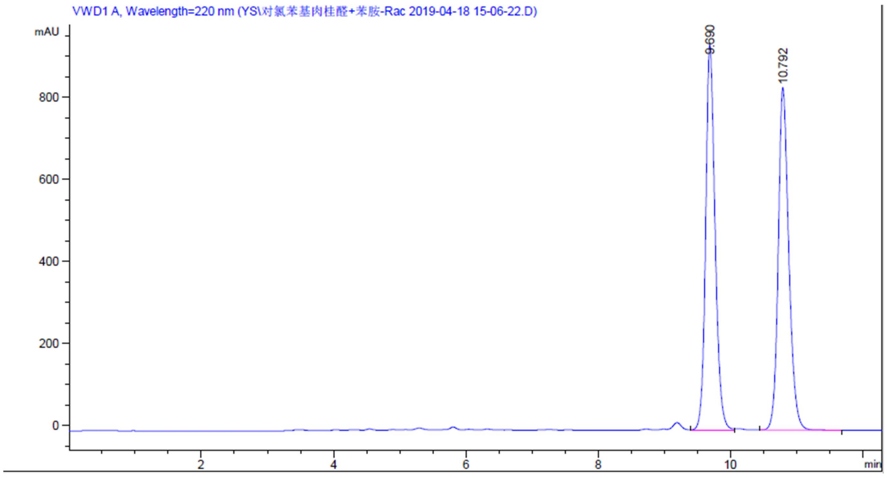

峰 #	保留时间 [min]	类型	峰宽 [min]	峰面积 [mAU*s]	峰高 [mAU]	峰面积 %
1	9.690	BV	0.1490	9270.61230	940.41315	50.0575
2	10.792	VB	0.1674	9249.30859	834.14990	49.9425

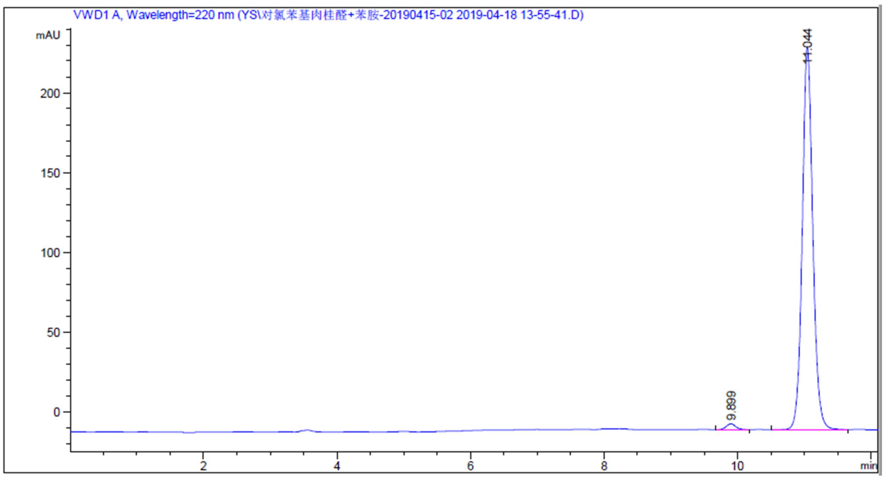

峰 #	保留时间 [min]	类型	峰宽 [min]	峰面积 [mAU*s]	峰高 [mAU]	峰面积 %
1	9.899	VV	0.1548	38.92135	3.78913	1.4568
2	11.044	BV	0.1682	2632.84131	239.59183	98.5432

Supplementary Figure 9. HPLC spectra for racemic and chiral **3f**.

***N*–(3–phenyl–2–(4–(trifluoromethyl)phenyl)propyl)aniline (3g)**: 98% yield, 97% ee, colorless oil.

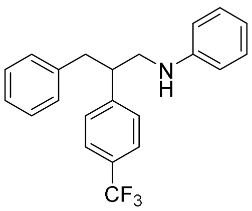

^{1}H NMR (500 MHz, $CDCl_3$): δ 7.66 (d, J = 7.7 Hz, 2H), 7.41–7.26 (m, 5H), 7.24 (t, J = 7.9 Hz, 2H), 7.19–7.14 (m, 2H), 6.80 (t, J = 7.3 Hz, 1H), 6.56 (d, J = 8.2 Hz, 2H), 3.58 (dd, J = 19.1, 9.5 Hz, 2H), 3.45–3.32 (m, 2H), 3.19–3.00 (m, 2H); ^{13}C NMR (125 MHz, $CDCl_3$): δ 147.72, 147.01, 139.28, 129.39, 129.33, 129.12, 129.07, 128.54, 128.36, 126.50, 125.67 (q, J = 3.7 Hz), 125.41, 123.25, 117.77, 113.09, 48.58, 47.06, 40.81. HRMS (ESI) m/z calcd for $C_{22}H_{21}F_3N^+$ $(M+H)^+$ 356.16206, found 356.16217. Enantiomeric excess was determined by chiral HPLC: Chiralpak IB–3 column, Hex/IPA=95:5, 1 mL/min, 220 nm, 13.10 min (minor), 14.49 min (major).

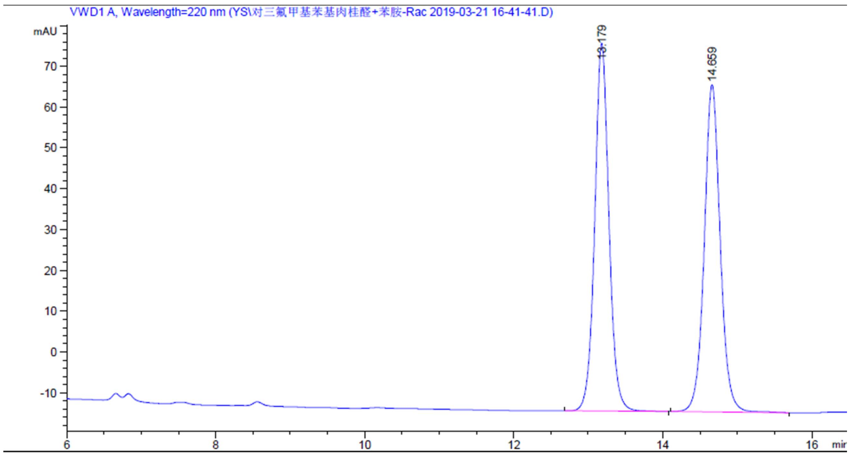

峰 #	保留时间 [min]	类型	峰宽 [min]	峰面积 [mAU*s]	峰高 [mAU]	峰面积 %
1	13.179	BB	0.2010	1197.93445	90.14127	50.0599
2	14.659	BB	0.2259	1195.06787	80.19238	49.9401

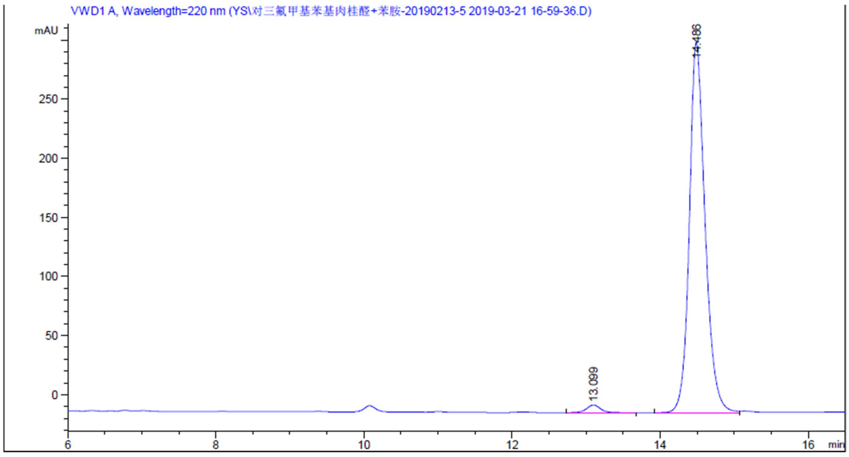

峰 #	保留时间 [min]	类型	峰宽 [min]	峰面积 [mAU*s]	峰高 [mAU]	峰面积 %
1	13.099	VB	0.1957	83.05169	6.47145	1.7682
2	14.486	BV	0.2199	4613.85059	313.09222	98.2318

Supplementary Figure 10. HPLC spectra for racemic and chiral **3g**.

***N*–(2–(3–methoxyphenyl)–3–phenylpropyl)aniline (3h)**: 98% yield, 98% ee, colorless oil. ^{1}H NMR (500 MHz, $CDCl_3$): 7.28–7.14 (m, 4H), 7.14–7.06 (m, 4H), 6.82–6.73 (m, 2H), 6.73–6.62 (m, 2H), 6.43 (t, J = 6.0 Hz, 2H), 3.77–3.71 (m, 3H), 3.61–3.38 (m, 2H), 3.27–3.11 (m, 2H), 2.97 (t, J = 6.6 Hz, 2H); ^{13}C NMR (125 MHz, $CDCl_3$): δ 159.89, 148.04, 144.45, 139.92, 129.73, 129.26, 129.16, 128.40, 126.25, 120.23, 117.47, 113.89, 113.12, 112.06, 55.24, 48.56, 47.10, 41.02. HRMS (ESI) m/z calcd for $C_{22}H_{24}NO^{+}$ $(M+H)^{+}$ 318.18524, found 318.18509. Enantiomeric excess was determined by chiral HPLC: Chiralpak IB–3 column, Hex/IPA=95:5, 1 mL/min, 220 nm, 10.11 min (major), 11.13 min (minor).

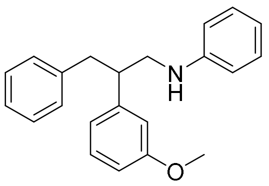

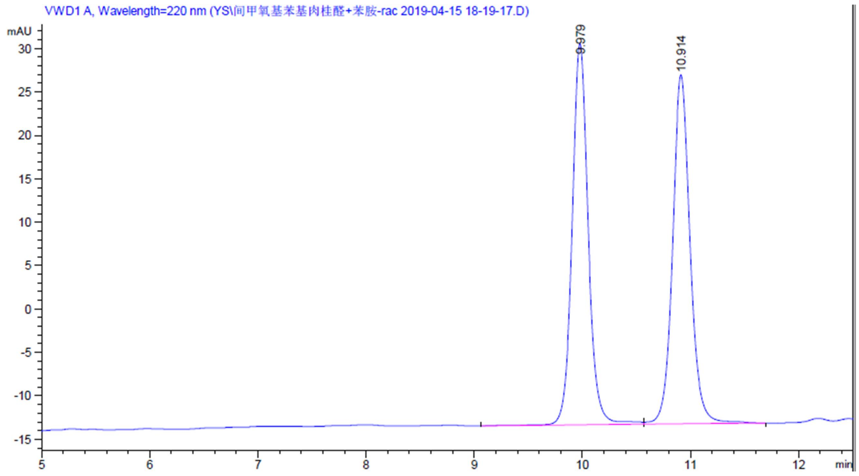

峰 #	保留时间 [min]	类型	峰宽 [min]	峰面积 [mAU*s]	峰高 [mAU]	峰面积 %
1	9.979	BV	0.1564	453.24146	43.92325	50.0865
2	10.914	VB	0.1711	451.67603	40.18555	49.9135

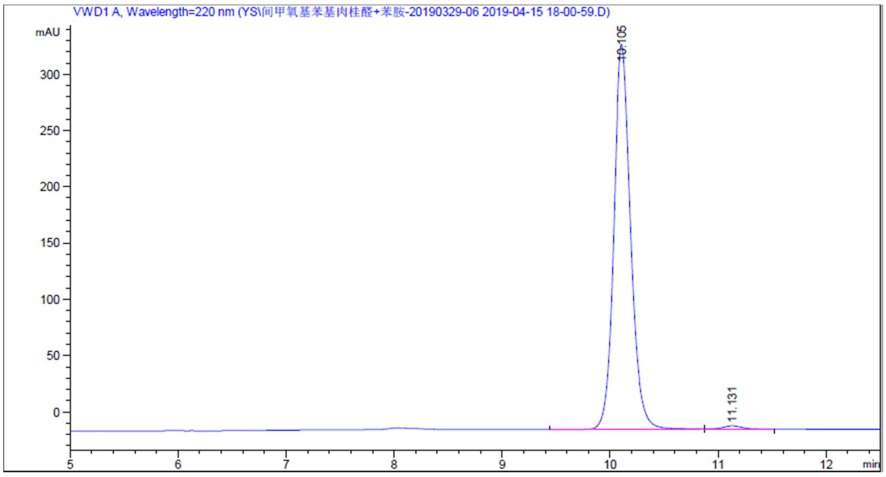

峰 #	保留时间 [min]	类型	峰宽 [min]	峰面积 [mAU*s]	峰高 [mAU]	峰面积 %
1	10.105	BV	0.1637	3686.14868	342.07248	98.9467
2	11.131	VV	0.1983	39.24051	2.96728	1.0533

Supplementary Figure 11. HPLC spectra for racemic and chiral **3h**.

***N*–(3–(1–phenyl–3–(phenylamino)propan–2–yl)phenyl)acetamide (3i)**: 91% yield, 95% ee, colorless oil. ^{1}H NMR (500 MHz, $CDCl_3$): δ 7.48 (s, 1H), 7.44–7.38 (m, 1H), 7.32 (s, 1H), 7.27–7.19 (m, 4H), 7.19–7.13 (m, 1H), 7.13–7.04 (m, 4H), 6.91 (d, J = 7.6 Hz, 1H), 6.65 (t, J = 7.3 Hz, 1H), 6.42 (d, J = 8.0 Hz, 2H), 3.50 (s, 1H), 3.41 (dd, J = 12.3, 4.9 Hz, 1H), 3.23 (dd, J = 12.2, 8.9 Hz, 1H), 3.20–3.10 (m, 1H), 2.96 (d, J = 7.3 Hz, 2H), 2.12 (s, 3H); ^{13}C NMR (125 MHz, $CDCl_3$): δ 168.57, 147.93, 143.73, 139.75, 138.34, 129.31, 129.23, 129.10, 128.37, 126.23, 123.85, 119.15, 118.44, 117.42, 113.00, 48.47, 47.01, 40.91, 24.64. HRMS (ESI) m/z calcd for $C_{23}H_{25}N2O^+$ $(M+H)^+$ 345.19614, found 345.19608. Enantiomeric excess was determined by chiral HPLC: Chiralpak IA–3 column, Hex/IPA=90:10, 1 mL/min, 220 nm, 19.06 min (major), 21.66 min (minor).

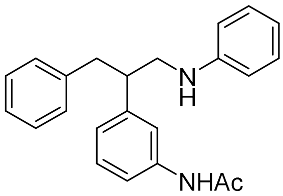

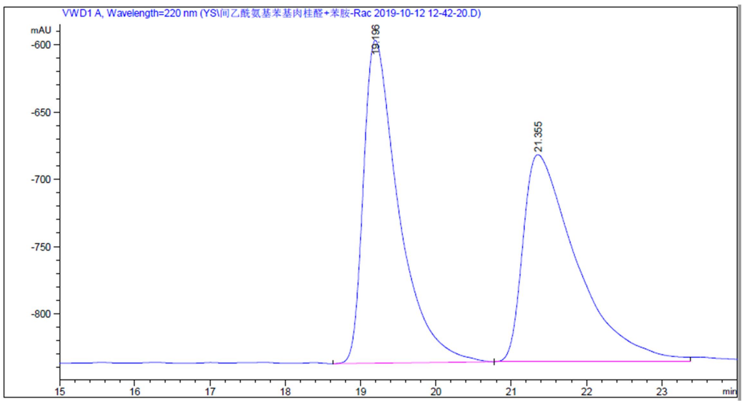

峰 #	保留时间 [min]	类型	峰宽 [min]	峰面积 [mAU*s]	峰高 [mAU]	峰面积 %
1	19.196	BB	0.4815	7799.98584	239.79781	50.5370
2	21.355	BV	0.7306	7634.20801	153.92505	49.4630

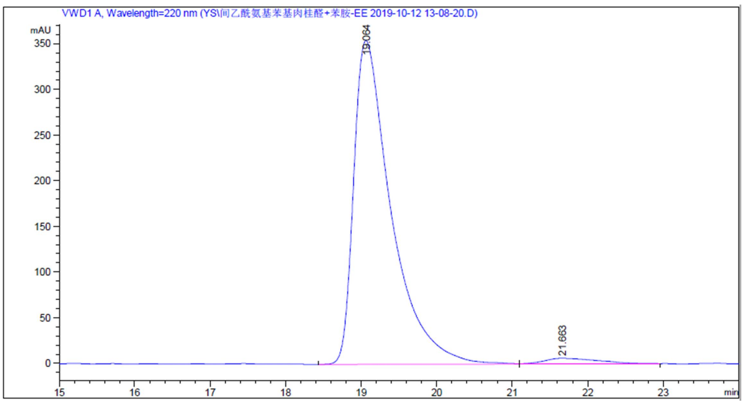

峰 #	保留时间 [min]	类型	峰宽 [min]	峰面积 [mAU*s]	峰高 [mAU]	峰面积 %
1	19.064	BV	0.5087	1.22374e4	354.50641	97.3088
2	21.663	VV	0.7995	338.44708	6.37347	2.6912

Supplementary Figure 12. HPLC spectra for racemic and chiral **3i**.

***N*–(2–(3,5–dimethylphenyl)–3–phenylpropyl)aniline (3j)**: 97% yield, 98% ee, colorless oil. ^{1}H

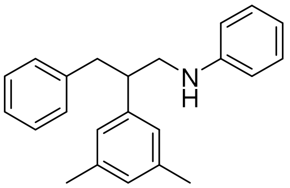

NMR (500 MHz, $CDCl_3$): δ 7.24 (t, J = 7.4 Hz, 2H), 7.21–7.04 (m, 5H), 6.87 (s, 1H), 6.80 (s, 2H), 6.65 (t, J = 7.3 Hz, 1H), 6.42 (d, J = 7.9 Hz, 2H), 3.39 (dd, J = 12.2, 4.9 Hz, 2H), 3.20 (dd, J = 12.2, 8.9 Hz, 1H), 3.15–3.06 (m, 1H), 3.02–2.88 (m, 2H), 2.28 (s, 6H); ^{13}C NMR (125 MHz, $CDCl_3$): δ 148.02, 142.68, 140.08, 138.06, 129.10, 129.05, 128.47, 128.27, 126.09, 125.58, 117.29, 113.02, 48.32, 46.76, 41.14, 21.33. HRMS (ESI) m/z calcd for $C_{23}H_{26}N^{+}$ $(M+H)^{+}$ 316.20598, found 316.20593. Enantiomeric excess was determined by chiral HPLC: Chiralpak IB–3 column, Hex/IPA=99.2:0.8, 1 mL/min, 220 nm, 10.89 min (minor), 11.62 min (major).

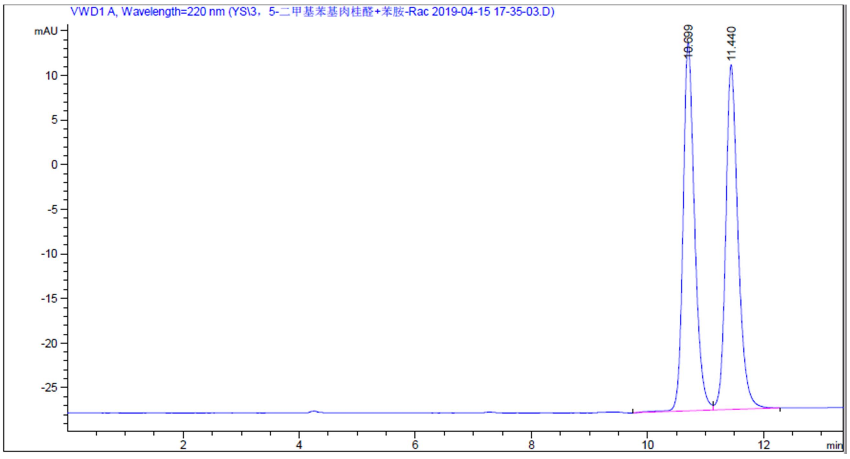

峰 #	保留时间 [min]	类型	峰宽 [min]	峰面积 [mAU*s]	峰高 [mAU]	峰面积 %
1	10.699	BV	0.2075	570.63269	41.23009	50.0493
2	11.440	VB	0.2220	569.50891	38.61846	49.9507

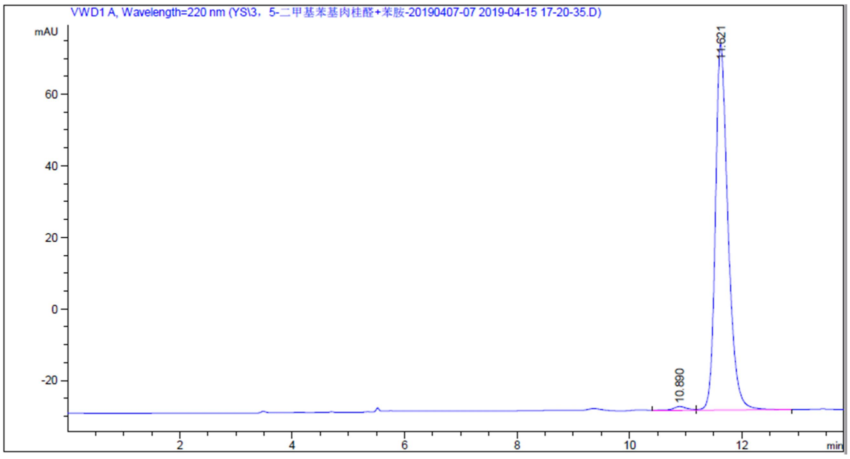

峰 #	保留时间 [min]	类型	峰宽 [min]	峰面积 [mAU*s]	峰高 [mAU]	峰面积 %
1	10.890	VV	0.2808	21.19745	1.09025	1.3048
2	11.621	VB	0.2348	1603.39832	102.33669	98.6952

Supplementary Figure 13. HPLC spectra for racemic and chiral **3j**.

3–(1–phenyl–3–(phenylamino)propan–2–yl)benzonitrile (3k): 94% yield, 95% ee, colorless oil.

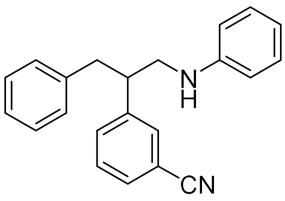

^{1}H NMR (500 MHz, $CDCl_3$): δ 7.54–7.45 (m, 1H), 7.42 (s, 1H), 7.40–7.34 (m, 2H), 7.27–7.20 (m, 2H), 7.20–7.15 (m, 1H), 7.13 (t, J = 8.5, 7.2 Hz, 2H), 7.03 (d, J = 6.8 Hz, 1H), 6.73–6.65 (m, 1H), 6.45 (d, J = 7.5 Hz, 2H), 3.55–3.40 (m, 2H), 3.33–3.19 (m, 2H), 3.04 (dd, J = 13.7, 6.7 Hz, 1H), 2.91 (dd, J = 13.7, 7.5 Hz, 1H); ^{13}C NMR (125 MHz, $CDCl_3$): δ 147.50, 144.30, 138.84, 132.57, 131.52, 130.62, 129.42, 129.36, 129.01, 128.52, 126.54, 118.86, 117.84, 113.00, 112.71, 48.51, 46.92, 40.64. HRMS (ESI) m/z calcd for $C_{22}H_{21}N_2^{+}$ $(M+H)^{+}$ 313.16993, found 313.17020. Enantiomeric excess was determined by chiral HPLC: Chiralpak IB–3 column, Hex/IPA=80:20, 1 mL/min, 220 nm, 13.57 min (minor), 14.71 min (major).

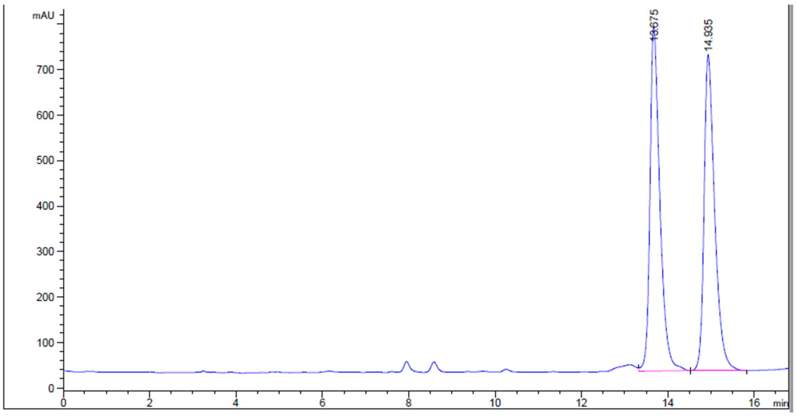

峰 #	保留时间 [min]	类型	峰宽 [min]	峰面积 [mAU*s]	峰高 [mAU]	峰面积 %
1	13.675	VB	0.2376	1.20504e4	757.48792	50.3621
2	14.935	BB	0.2554	1.18771e4	694.92328	49.6379

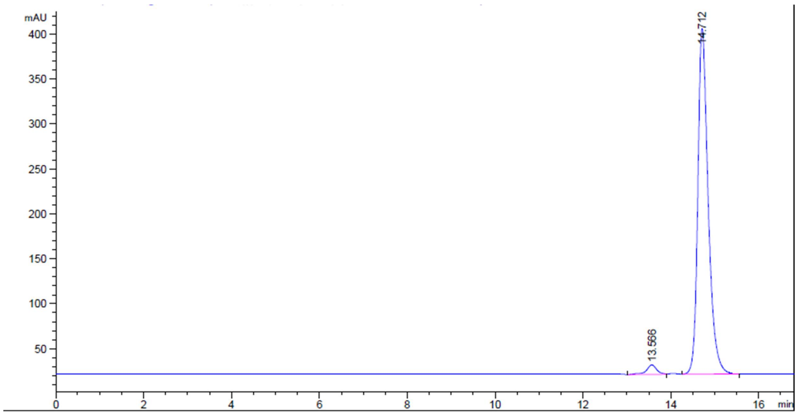

峰 #	保留时间 [min]	类型	峰宽 [min]	峰面积 [mAU*s]	峰高 [mAU]	峰面积 %
1	13.566	BV	0.2409	172.39459	10.59290	2.6691
2	14.712	VB	0.2459	6286.61426	384.10437	97.3309

Supplementary Figure 14. HPLC spectra for racemic and chiral **3k**.

***N*–(3–phenyl–2–(o–tolyl)propyl)aniline (3l)**: 94% yield, 96% ee, colorless oil. ^{1}H NMR (500 MHz, $CDCl_3$): δ 7.28 (d, J = 7.8 Hz, 1H), 7.25–7.02 (m, 10H), 6.65 (t, J = 7.3 Hz, 1H), 6.44 (d, J = 7.9 Hz, 2H), 3.61–3.36 (m, 3H), 3.31 (dd, J = 11.9, 8.4 Hz, 1H), 3.03–2.85 (m, 2H), 2.08 (s, 3H); ^{13}C NMR (125 MHz, $CDCl_3$): δ 148.19, 140.95, 140.08, 137.10, 130.64, 129.30, 129.15, 128.41, 126.58, 126.50, 126.27, 125.98, 117.51, 113.13, 48.31, 41.90, 41.45, 19.71. HRMS (ESI) m/z calcd for $C_{22}H_{24}N^{+}$ $(M+H)^{+}$ 302.19033, found 302.19019. Enantiomeric excess was determined by chiral HPLC: Chiralpak IB–3 column, Hex/IPA=98:2, 1 mL/min, 220 nm, 11. 01 min (minor), 12. 01 min (major).

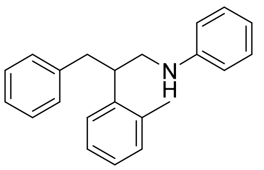

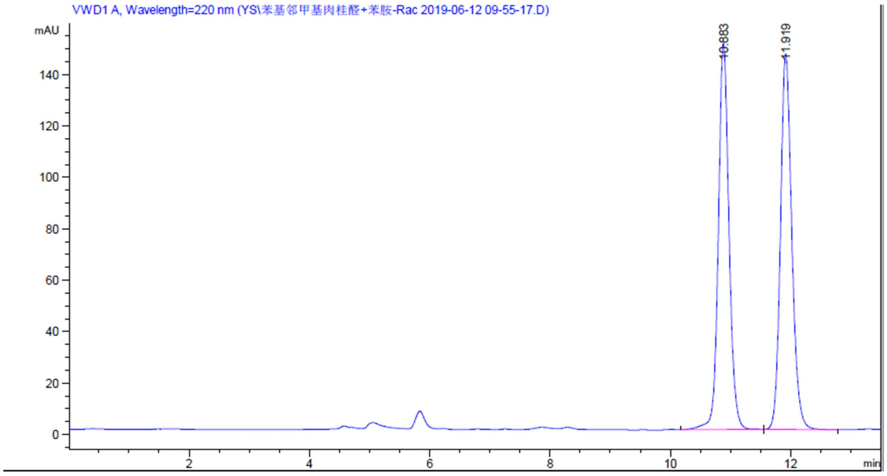

峰 #	保留时间 [min]	类型	峰宽 [min]	峰面积 [mAU*s]	峰高 [mAU]	峰面积 %
1	10.883	VB	0.1875	1850.68555	150.44888	49.2235
2	11.919	BB	0.1986	1909.07202	145.98317	50.7765

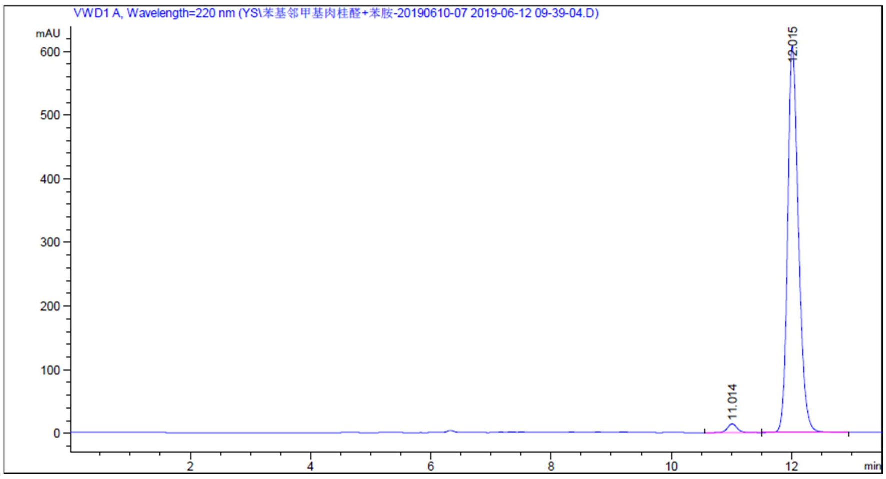

峰 #	保留时间 [min]	类型	峰宽 [min]	峰面积 [mAU*s]	峰高 [mAU]	峰面积 %
1	11.014	BB	0.1699	154.74762	13.89967	1.9958
2	12.015	BV	0.1901	7598.97803	606.95984	98.0042

Supplementary Figure 15. HPLC spectra for racemic and chiral **3l**.

***N*–(2–(2–fluorophenyl)–3–phenylpropyl)aniline (3m)**: 88% yield, 97% ee, colorless oil. ^{1}H NMR (500 MHz, $CDCl_3$): δ 7.29–6.96 (m, 11H), 6.66 (t, *J* = 7.3 Hz, 1H), 6.46 (d, *J* = 7.9 Hz, 2H), 3.60–3.32 (m, 4H), 3.08–2.95 (m, 2H); ^{13}C NMR (125 MHz, $CDCl_3$): δ 162.42, 160.47, 148.04, 139.75, 129.51, 129.40, 129.30, 129.24, 129.12, 128.43, 128.38, 128.32, 126.33, 124.38, 124.36, 117.52, 115.91, 115.73, 113.01, 47.54, 41.17, 39.66. HRMS (ESI) m/z calcd for $C_{21}H_{21}FN^{+}$ $(M+H)^{+}$ 306.16525, found 306.16534. Enantiomeric excess was determined by chiral HPLC: Chiralpak IA–3 column, Hex/IPA=95:5, 1 mL/min, 220 nm, 7. 26 min (minor), 7. 87 min (major).

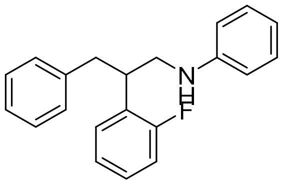

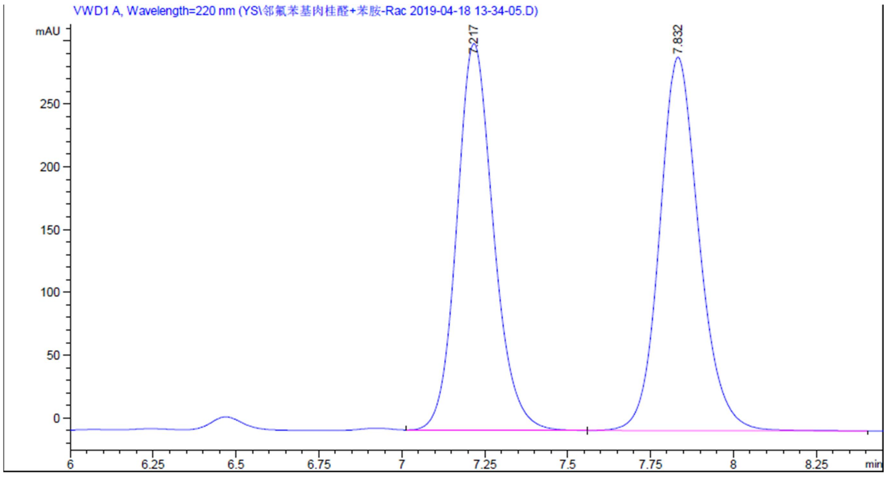

峰 #	保留时间 [min]	类型	峰宽 [min]	峰面积 [mAU*s]	峰高 [mAU]	峰面积 %
1	7.217	VB	0.1176	2362.55835	307.68054	49.0445
2	7.832	BB	0.1265	2454.61230	296.84650	50.9555

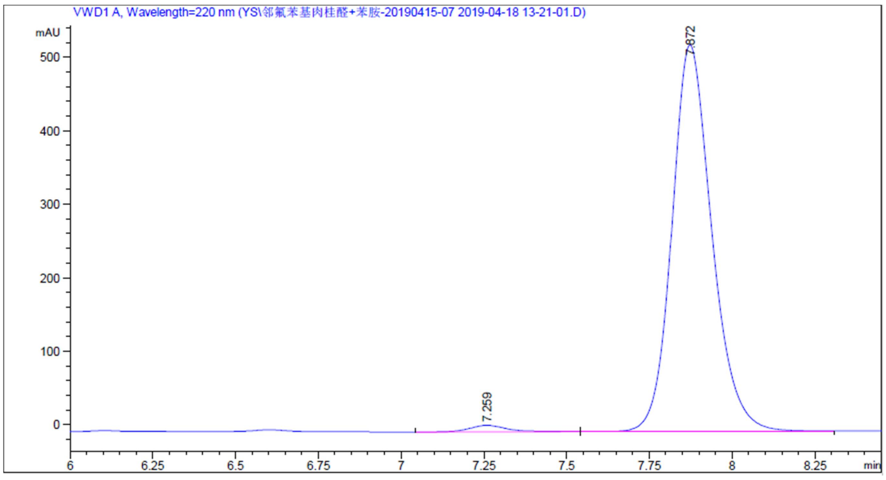

峰 #	保留时间 [min]	类型	峰宽 [min]	峰面积 [mAU*s]	峰高 [mAU]	峰面积 %
1	7.259	BV	0.1231	73.49397	8.82854	1.6354
2	7.872	VV	0.1280	4420.58057	526.37775	98.3646

Supplementary Figure 16. HPLC spectra for racemic and chiral **3m**.

2–(2–chlorophenyl)–*N*–(4–methoxybenzyl)–3–phenylpropan–1–amine (3n): 92% yield, 96% ee, colorless oil. ^{1}H NMR (500 MHz, $CDCl_3$): δ 7.32 (dd, J = 7.9, 1.4 Hz, 1H), 7.27–7.05 (m, 8H), 6.70 (d, J = 8.9 Hz, 2H), 6.41 (d, J = 9.0 Hz, 2H), 3.93–3.78 (m, 1H), 3.67 (s, 3H), 3.40–3.25 (m, 2H), 3.18 (s, 1H), 3.02–2.83 (m, 2H); ^{13}C NMR (125 MHz, $CDCl_3$): δ 152.22, 142.35, 140.19, 139.57, 134.81, 129.98, 129.21, 128.46, 128.10, 127.92, 127.25, 126.38, 114.92, 114.32, 55.87, 48.43, 42.72, 40.09. HRMS (ESI) m/z calcd for $C_{22}H_{23}ClNO^+$ $(M+H)^+$ 352.14627, found 352.14603. Enantiomeric excess was determined by chiral HPLC: Chiralpak AS-H column, Hex/IPA=95:5, 1 mL/min, 220 nm, 9.83 min (minor), 10.62 min (major).

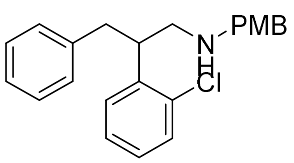

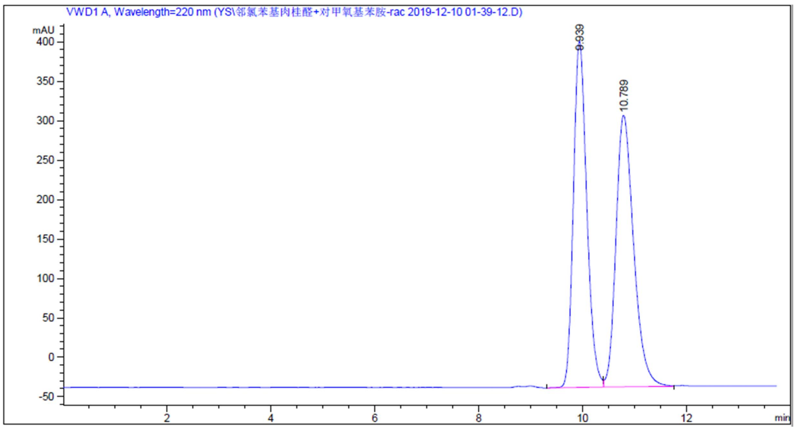

峰 #	保留时间 [min]	类型	峰宽 [min]	峰面积 [mAU*s]	峰高 [mAU]	峰面积 %
1	9.939	BV	0.2755	7843.07959	438.90320	49.0480
2	10.789	VV	0.3631	8147.53174	344.22058	50.9520

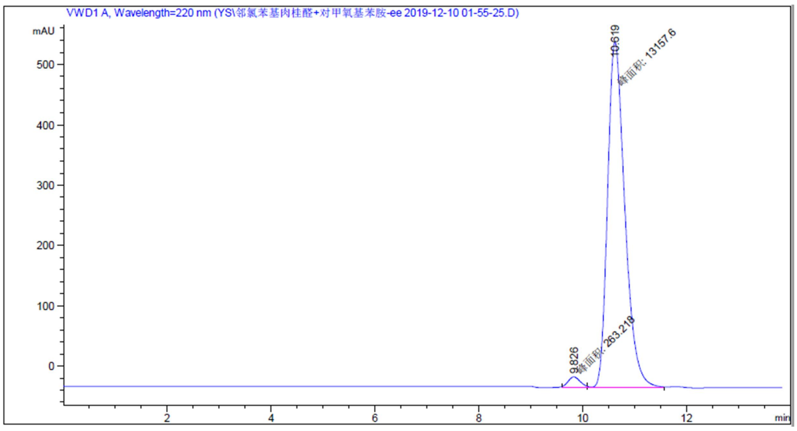

峰 #	保留时间 [min]	类型	峰宽 [min]	峰面积 [mAU*s]	峰高 [mAU]	峰面积 %
1	9.826	MF	0.2613	263.21762	16.78812	1.9613
2	10.619	FM	0.3834	1.31576e4	572.04242	98.0387

Supplementary Figure 17. HPLC spectra for racemic and chiral **3n**.

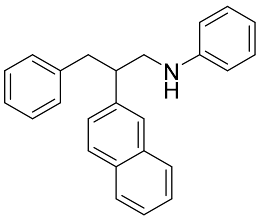

***N*–(2–(naphthalen–2–yl)–3–phenylpropyl)aniline (3o)**: 95% yield, 98% ee, colorless oil. ^{1}H NMR (500 MHz, $CDCl_3$): δ 7.83–7.69 (m, 3H), 7.58 (s, 1H), 7.47–7.37 (m, 2H), 7.32 (dd, *J* = 8.5, 1.9 Hz, 1H), 7.20 (t, *J* = 7.3 Hz, 2H), 7.17–7.05 (m, 5H), 6.65 (t, *J* = 7.3 Hz, 1H), 6.42 (d, *J* = 7.9 Hz, 2H), 3.75–3.43 (m, 2H), 3.40–3.28 (m, 2H), 3.06 (d, *J* = 6.5 Hz, 2H).; ^{13}C NMR (125 MHz, $CDCl_3$): δ 147.85, 140.00, 139.72, 133.48, 132.53, 129.14, 129.03, 128.41, 128.28, 127.62, 127.61, 126.75, 126.14, 126.06, 125.75, 125.56, 117.41, 113.01, 48.43, 47.09, 40.95. HRMS (ESI) m/z calcd for $C_{25}H_{24}N^{+}$ $(M+H)^{+}$ 338.19033, found 338.19052. Enantiomeric excess was determined by chiral HPLC: Chiralpak IB–3 column, Hex/IPA=95:5, 1 mL/min, 220 nm, 10.29 min (minor), 11.19 min (major).

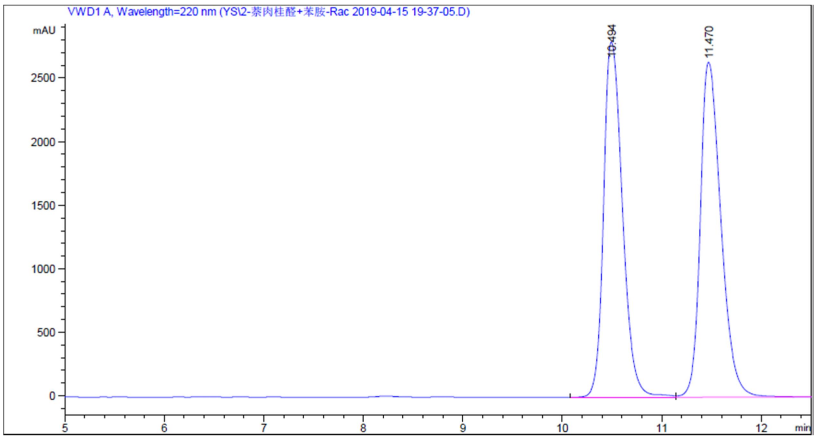

峰 #	保留时间 [min]	类型	峰宽 [min]	峰面积 [mAU*s]	峰高 [mAU]	峰面积 %
1	10.494	BV	0.2038	3.68553e4	2795.11304	49.2628
2	11.470	VB	0.2221	3.79584e4	2633.85449	50.7372

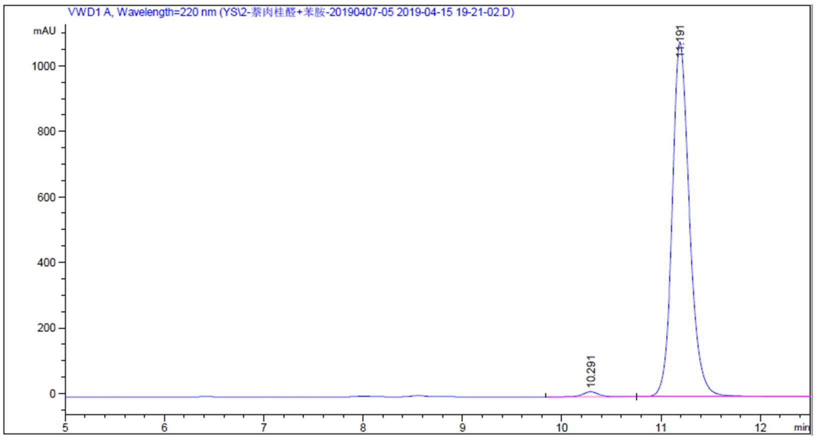

峰 #	保留时间 [min]	类型	峰宽 [min]	峰面积 [mAU*s]	峰高 [mAU]	峰面积 %
1	10.291	BB	0.1673	169.46053	15.53405	1.2746
2	11.191	BV	0.1834	1.31261e4	1082.87939	98.7254

Supplementary Figure 18. HPLC spectra for racemic and chiral **3o**.

***N*–(2–(naphthalen–1–yl)–3–phenylpropyl)aniline (3p)**: 95% yield, 96% ee, colorless oil. ^{1}H NMR (500 MHz, $CDCl_3$): δ 8.11–7.99 (m, 1H), 7.89–7.82 (m, 1H), 7.76 (d, J = 7.6 Hz, 1H), 7.58–7.37 (m, 4H), 7.28–7.11 (m,5H), 7.08 (t, J = 7.7 Hz, 2H), 6.64 (t, J = 7.3 Hz, 1H), 6.38 (d, J = 8.0 Hz, 2H), 4.20–4.05 (m, 1H), 3.55 (dd, J = 12.3, 5.3 Hz, 1H), 3.52–3.30 (m, 2H), 3.21–2.99 (m, 2H); ^{13}C NMR (125 MHz, $CDCl_3$): δ 147.96, 139.89, 138.79, 134.02, 132.12, 129.10, 129.08, 129.00, 128.36, 127.26, 126.24, 126.07, 125.56, 125.52, 122.90, 117.32, 112.88, 47.71, 40.89. HRMS (ESI) m/z calcd for $C_{25}H_{24}N^{+}$ $(M+H)^{+}$ 338.19033, found 338.19019. Enantiomeric excess was determined by chiral HPLC: Chiralpak IB–3 column, Hex/IPA=95:5, 1 mL/min, 220 nm, 14. 88 min (minor), 16. 27 min (major).

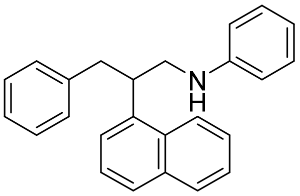

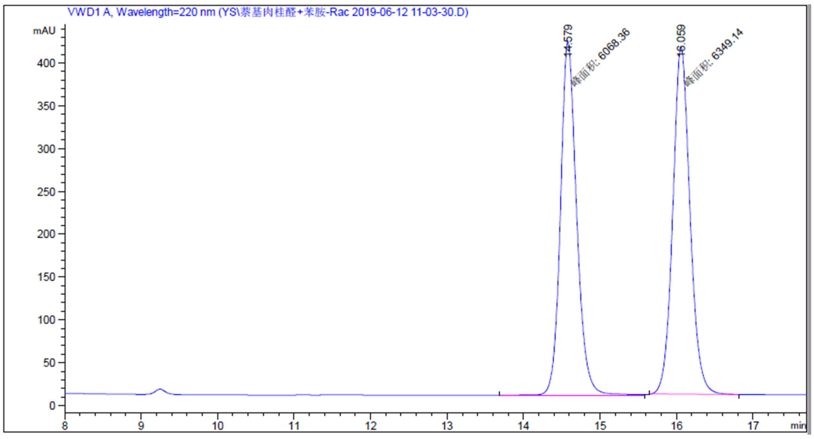

峰 #	保留时间 [min]	类型	峰宽 [min]	峰面积 [mAU*s]	峰高 [mAU]	峰面积 %
1	14.579	MM	0.2452	6068.35693	412.50659	48.8694
2	16.059	MM	0.2610	6349.13867	405.41147	51.1306

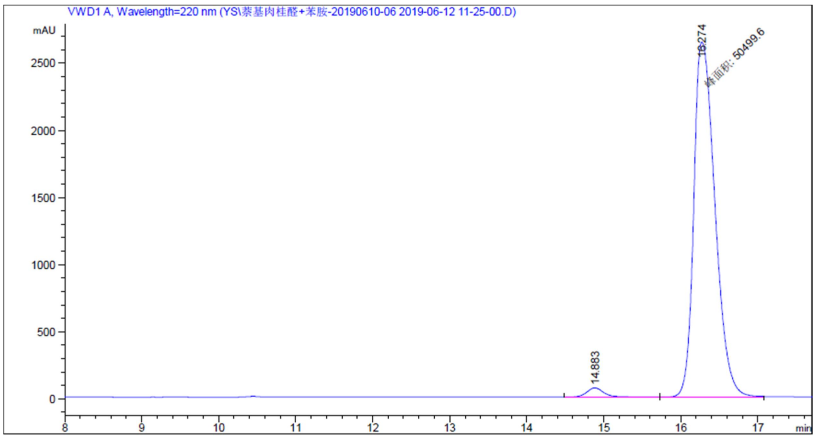

峰 #	保留时间 [min]	类型	峰宽 [min]	峰面积 [mAU*s]	峰高 [mAU]	峰面积 %
1	14.883	VB	0.2332	1054.66724	68.65220	2.0457
2	16.274	MF	0.3182	5.04996e4	2644.81958	97.9543

Supplementary Figure 19. HPLC spectra for racemic and chiral **3p**.

***N*–(3–phenyl–2–(thiophen–2–yl)propyl)aniline (3q)**: 95% yield, 95% ee, colorless oil. ^{1}H NMR (500 MHz, $CDCl_3$): δ 7.25 (t, *J* = 7.4 Hz, 2H), 7.22–7.15 (m, 2H), 7.15–7.07 (m, 4H), 6.92 (dd, *J* = 5.2, 3.4 Hz, 1H), 6.78 (d, *J* = 3.4 Hz, 1H), 6.67 (t, *J* = 7.3 Hz, 1H), 6.45 (d, *J* = 7.9 Hz, 2H), 3.65 (s, 1H), 3.55–3.41 (m, 2H), 3.22 (dd, *J* = 12.4, 8.2 Hz, 1H), 3.03 (d, *J* = 7.3 Hz, 2H); ^{13}C NMR (125 MHz, $CDCl_3$): δ 147.71, 146.37, 139.36, 129.18, 128.99, 128.33, 126.72, 126.32, 124.71, 123.71, 117.52, 113.08, 49.33, 42.50, 41.72. HRMS (ESI) m/z calcd for $C_{19}H_{20}NS^{+}$ $(M+H)^{+}$ 294.13110, found 294.13116. Enantiomeric excess was determined by chiral HPLC: Chiralpak IB–3 column, Hex/IPA=95:5, 1 mL/min, 220 nm, 7. 52 min (minor), 8. 24 min (major).

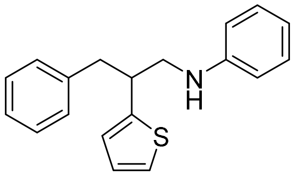

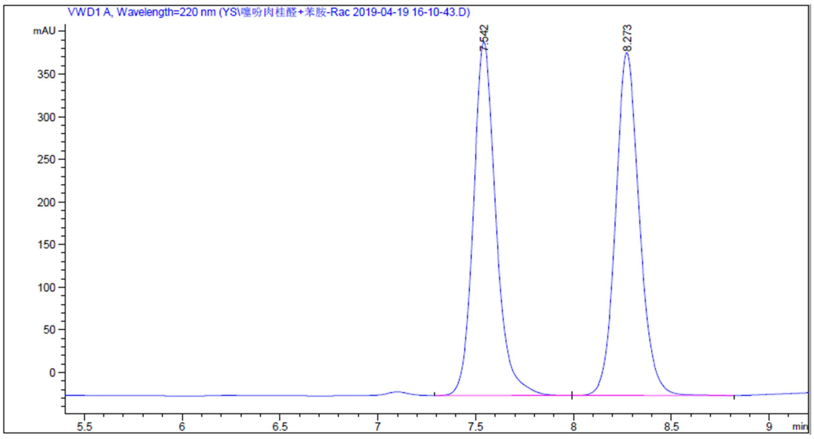

峰 #	保留时间 [min]	类型	峰宽 [min]	峰面积 [mAU*s]	峰高 [mAU]	峰面积 %
1	7.542	VB	0.1214	3319.08691	414.49908	49.3874
2	8.273	BB	0.1288	3401.42725	401.82404	50.6126

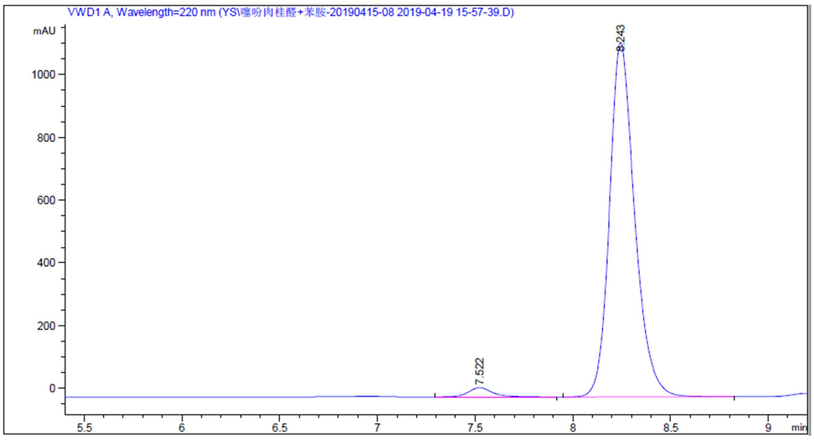

Z

峰 #	保留时间 [min]	类型	峰宽 [min]	峰面积 [mAU*s]	峰高 [mAU]	峰面积 %
1	7.522	VB	0.1276	253.25006	29.67105	2.4444
2	8.243	BB	0.1353	1.01071e4	1130.37097	97.5556

Supplementary Figure 20. HPLC spectra for racemic and chiral **3q**.

***N*–(2–methyl–3–phenylpropyl)aniline (3r)**: 92% yield, 94% ee, colorless oil. ^{1}H NMR(500 MHz, $CDCl_3$): δ 7.27 (t, *J* = 7.4 Hz, 2H), 7.22–7.09 (m, 5H), 6.67 (t, *J* = 7.3 Hz, 1H), 6.52 (d, *J* = 7.9 Hz, 2H), 3.65 (s, 1H), 3.07 (dd, *J* = 12.5, 6.0 Hz, 1H), 2.93 (dd, *J* = 12.5, 7.0 Hz, 1H), 2.74 (dd, *J* = 13.5, 6.4 Hz, 1H), 2.48 (dd, *J* = 13.5, 7.8 Hz, 1H), 2.14–1.98 (m, 1H), 0.96 (d, *J* = 6.8 Hz, 3H); ^{13}C NMR (125 MHz, $CDCl_3$): δ 148.35, 140.47, 129.17, 129.10, 128.26, 125.93, 117.08, 112.72, 49.85, 41.36, 35.00, 18.04. Enantiomeric excess was determined by chiral HPLC: Chiralpak IB–3 column, Hex/IPA=98:2, 1 mL/min, 220 nm, 7.69 min (minor), 8.11 min (major).

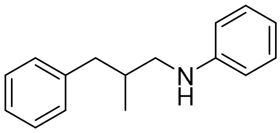

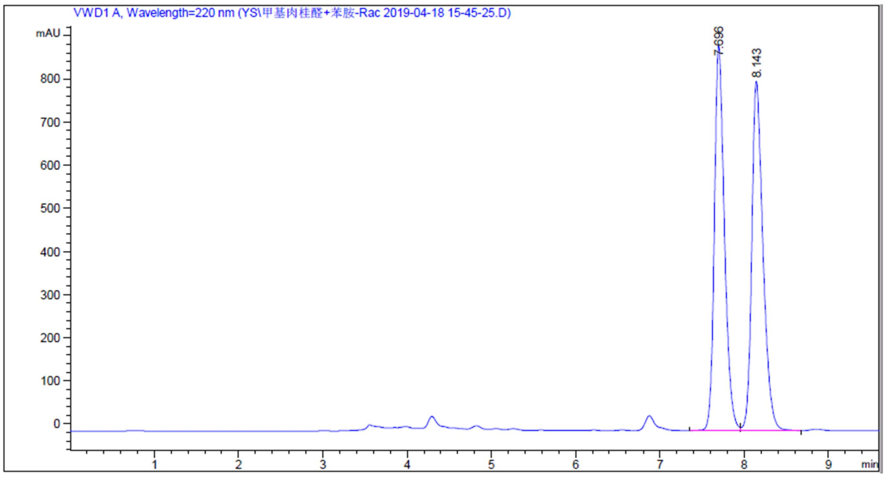

峰 #	保留时间 [min]	类型	峰宽 [min]	峰面积 [mAU*s]	峰高 [mAU]	峰面积 %
1	7.696	BV	0.1271	7431.67188	892.74091	49.8292
2	8.143	VB	0.1398	7482.60840	809.60950	50.1708

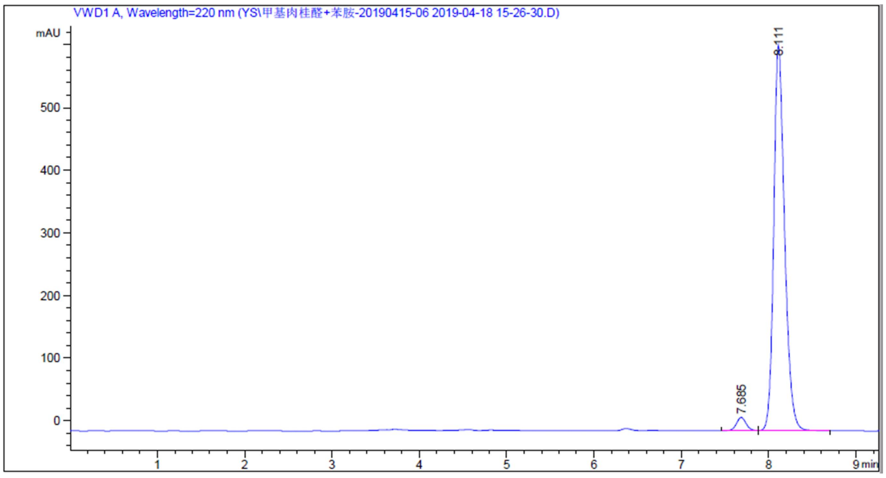

峰 #	保留时间 [min]	类型	峰宽 [min]	峰面积 [mAU*s]	峰高 [mAU]	峰面积 %
1	7.685	BV	0.1207	166.50104	21.42282	3.0359
2	8.111	VV	0.1308	5317.93506	615.53784	96.9641

Supplementary Figure 21. HPLC spectra for racemic and chiral **3r**.

***N*–(2–phenyl–3–(p–tolyl)propyl)aniline (3s)**: 92% yield, 99% ee, colorless oil. ^{1}H NMR(500 MHz, $CDCl_3$): δ 7.29 (t, J = 7.6 Hz, 2H), 7.24–7.14 (m, 3H), 7.13–7.06 (m, 2H), 7.03 (d, J = 7.8 Hz, 2H), 6.97 (d, J = 8.1 Hz, 2H), 6.65 (t, J = 7.4 Hz, 1H), 6.46–6.40 (m, 2H), 3.54–3.38 (m, 2H), 3.23 (dd, J = 12.1, 8.9 Hz, 1H), 3.20–3.11 (m, 1H), 2.94 (d, J = 7.3 Hz, 2H), 2.29 (s, 3H); ^{13}C NMR (125 MHz, $CDCl_3$): δ 147.96, 142.73, 136.66, 135.53, 129.11, 128.94, 128.89, 128.60, 127.84, 126.73, 117.30, 112.96, 48.45, 46.96, 40.56, 20.99. HRMS (ESI) m/z calcd for $C_{22}H_{24}N^+$ $(M+H)^+$ 302.19033, found 302.19049. Enantiomeric excess was determined by chiral HPLC: Chiralpak IA–3 column, Hex/IPA=90:10, 1 mL/min, 220 nm, 5. 16 min (minor), 5.94 min (major).

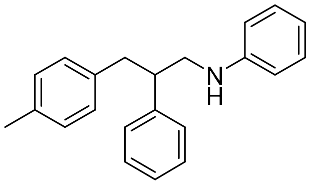

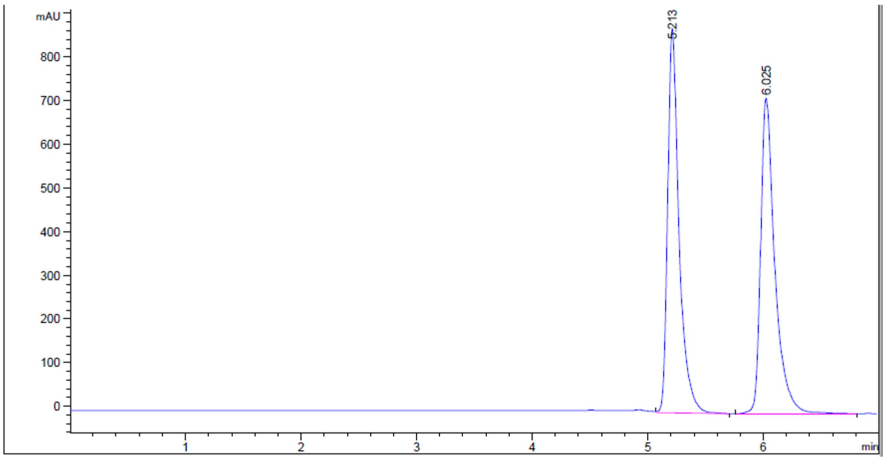

峰 #	保留时间 [min]	类型	峰宽 [min]	峰面积 [mAU*s]	峰高 [mAU]	峰面积 %
1	5.213	VB	0.1017	6011.88379	879.87762	49.4247
2	6.025	BB	0.1253	6151.84668	722.65240	50.5753

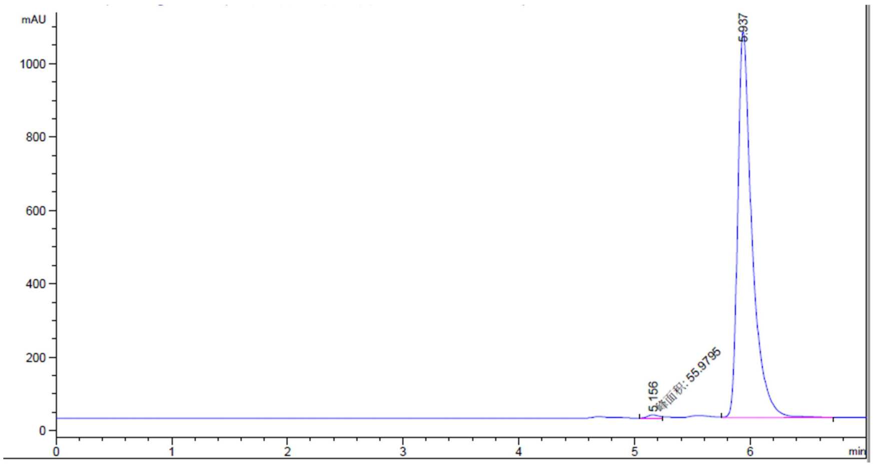

峰 #	保留时间 [min]	类型	峰宽 [min]	峰面积 [mAU*s]	峰高 [mAU]	峰面积 %
1	5.156	MF	0.1063	55.97951	8.77294	0.6119
2	5.937	VB	0.1269	9092.93359	1051.77014	99.3881

Supplementary Figure 22. HPLC spectra for racemic and chiral **3s**.

***N*–(3–(4–(tert–butyl)phenyl)–2–phenylpropyl)aniline (3t)**: 97% yield, 98% ee, colorless oil. ^{1}H NMR(500 MHz, $CDCl_3$): δ 7.47–7.29 (m, 7H), 7.24–7.14 (m, 4H), 6.77 (t, *J* = 7.3 Hz, 1H), 6.52 (d, *J* = 8.0 Hz, 2H), 3.56 (dd, *J* = 12.1, 4.8 Hz, 2H), 3.41–3.25 (m, 2H), 3.15–3.01 (m, 2H), 1.42 (s, 9H); ^{13}C NMR (125 MHz, $CDCl_3$): δ148.91, 147.91, 143.01, 136.77, 129.07, 128.66, 128.61, 127.78, 126.71, 125.15, 117.26, 112.93, 48.46, 46.80, 40.47, 34.32, 31.36. HRMS (ESI) m/z calcd for $C_{25}H_{30}N^+$ $(M+H)^+$ 344.23728, found 344.23737. Enantiomeric excess was determined by chiral HPLC: Chiralpak IA–3 column, Hex/IPA=99:1, 1 mL/min, 220 nm, 5. 65 min (minor), 7. 15 min (major).

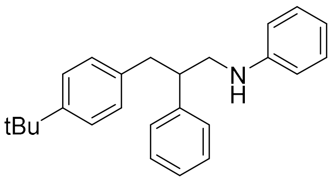

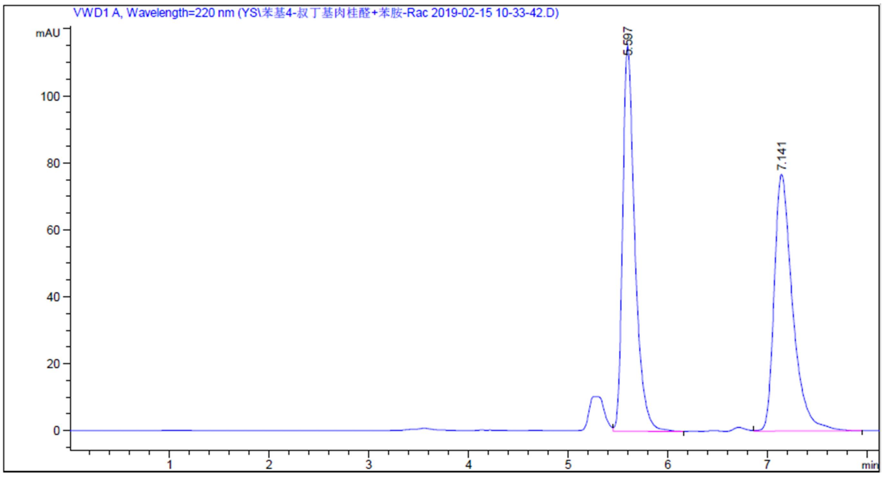

峰 #	保留时间 [min]	类型	峰宽 [min]	峰面积 [mAU*s]	峰高 [mAU]	峰面积 %
1	5. 597	VB	0. 1284	980. 59686	115. 10069	49. 8791
2	7. 141	VB	0. 1940	985. 35120	76. 63815	50. 1209

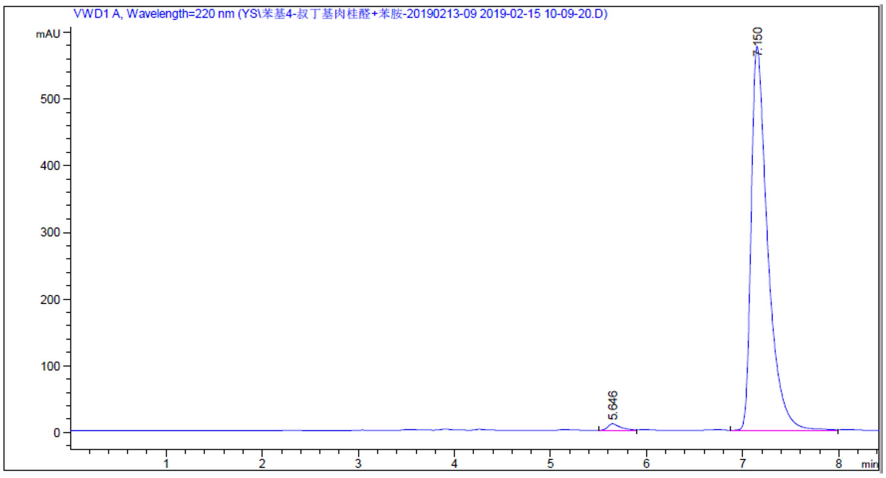

峰 #	保留时间 [min]	类型	峰宽 [min]	峰面积 [mAU*s]	峰高 [mAU]	峰面积 %
1	5. 646	VV	0. 1330	89. 41028	9. 75174	1. 2832
2	7. 150	VV	0. 1774	6878. 23047	575. 55450	98. 7168

Supplementary Figure 23. HPLC spectra for racemic and chiral **3t**.

***N*–(3–(4–chlorophenyl)–2–phenylpropyl)aniline (3u)**: 97% yield, 96% ee, colorless oil.^{1}H NMR(500 MHz, $CDCl_3$): δ 7.29 (t, J = 7.4 Hz, 2H), 7.25–7.19 (m, 1H), 7.19–7.07 (m, 6H), 6.96 (d, J = 8.1 Hz, 2H), 6.67 (t, J = 7.3 Hz, 1H), 6.46 (d, J = 8.0 Hz, 2H), 3.48 (s, 1H), 3.43 (dd, J = 12.4, 5.3 Hz, 1H), 3.26 (dd, J = 12.4, 8.8 Hz, 1H), 3.19–3.08 (m, 1H), 3.04–2.85 (m, 2H); ^{13}C NMR (125 MHz, $CDCl_3$): δ 147.84, 141.99, 138.22, 131.85, 130.34, 129.19, 128.69, 128.32, 127.83, 126.93, 117.51, 113.00, 48.55, 46.98, 40.18. HRMS (ESI) m/z calcd for $C_{21}H_{21}ClN^{+}$ $(M+H)^{+}$ 322.13570, found 322.13599. Enantiomeric excess was determined by chiral HPLC: Chiralpak IB–3 column, Hex/IPA=98:2, 1 mL/min, 220 nm, 11. 62 min (minor), 12. 10 min (major).

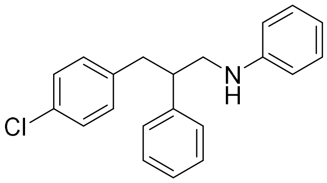

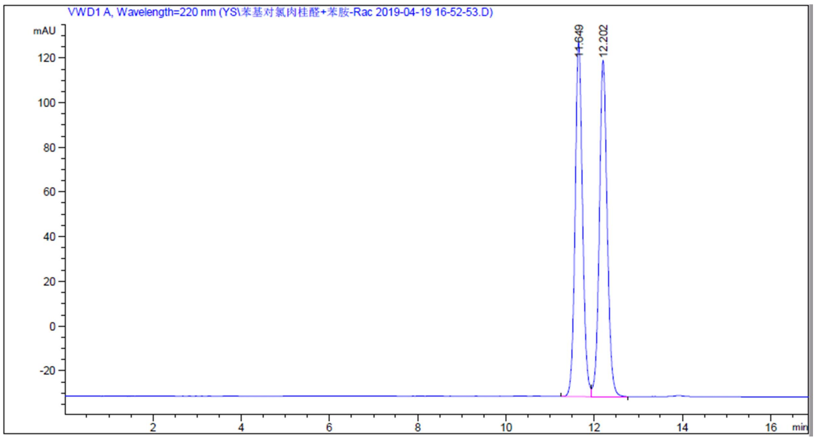

峰 #	保留时间 [min]	类型	峰宽 [min]	峰面积 [mAU*s]	峰高 [mAU]	峰面积 %
1	11.649	BV	0.1780	1849.40295	158.70419	49.7987
2	12.202	VB	0.1886	1864.35669	150.42131	50.2013

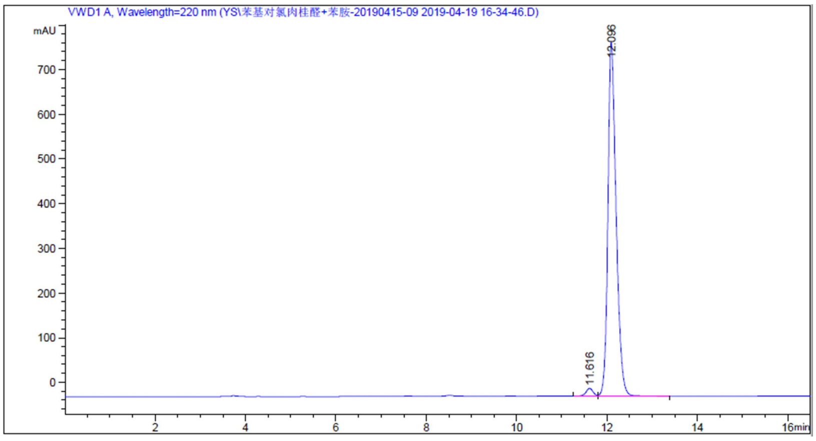

峰 #	保留时间 [min]	类型	峰宽 [min]	峰面积 [mAU*s]	峰高 [mAU]	峰面积 %
1	11.616	VV	0.1758	210.19321	18.05209	2.0496
2	12.096	VV	0.1898	1.00451e4	793.01660	97.9504

Supplementary Figure 24. HPLC spectra for racemic and chiral **3u**.

***N*–(2–phenyl–3–(m–tolyl)propyl)aniline (3v)**: 98% yield, 97% ee, colorless oil.^{1}H NMR(500 MHz, $CDCl_3$): δ 7.29 (t, *J* = 7.5 Hz, 2H), 7.25–7.04 (m, 6H), 6.98 (d, *J* = 7.6 Hz, 1H), 6.91 (s, 1H), 6.88 (d, *J* = 7.6 Hz, 1H), 6.65 (t, *J* = 7.3 Hz, 1H), 6.41 (d, *J* = 7.9 Hz, 2H), 3.42 (dd, *J* = 12.1, 4.8 Hz, 2H), 3.28–3.11 (m, 2H), 3.00–2.86 (m, 2H), 2.28 (s, 3H); ^{13}C NMR (125 MHz, $CDCl_3$): δ147.92, 142.80, 139.72, 137.77, 129.81, 129.10, 128.60, 128.12, 127.80, 126.84, 126.73, 126.04, 117.29, 112.96, 48.42, 46.81, 40.94, 21.33. HRMS (ESI) m/z calcd for $C_{22}H_{24}N^+$ $(M+H)^+$ 302.19033, found 302.19040. Enantiomeric excess was determined by chiral HPLC: Chiralpak IB–3 column, Hex/IPA=95:5, 1 mL/min, 220 nm, 6. 70 min (minor), 7. 10 min (major).

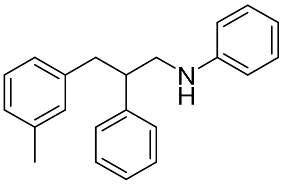

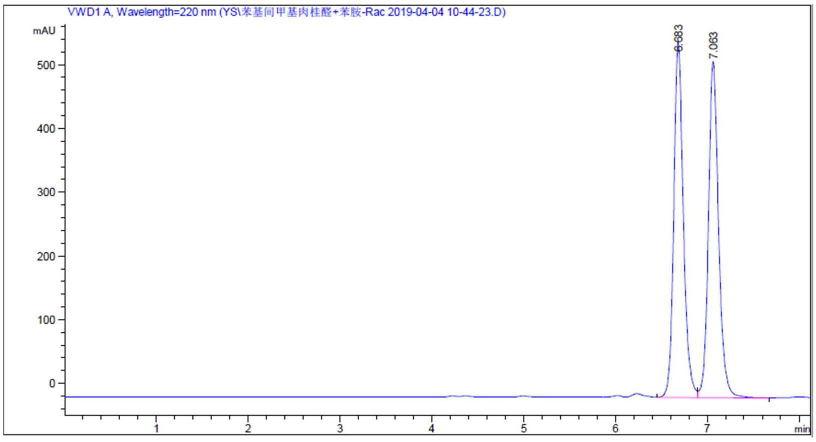

峰 #	保留时间 [min]	类型	峰宽 [min]	峰面积 [mAU*s]	峰高 [mAU]	峰面积 %
1	6.683	BV	0.1094	4032.63550	557.24103	49.9109
2	7.063	VB	0.1166	4047.03345	526.90094	50.0891

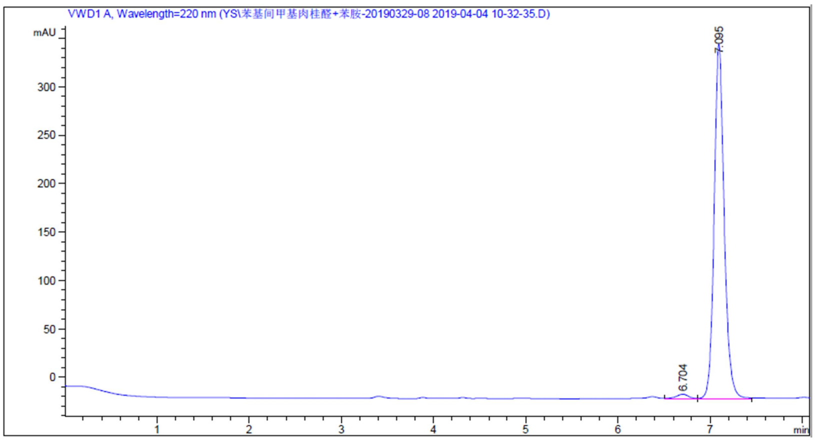

峰 #	保留时间 [min]	类型	峰宽 [min]	峰面积 [mAU*s]	峰高 [mAU]	峰面积 %
1	6.704	VV	0.1375	41.85034	4.62759	1.4905
2	7.095	VV	0.1141	2765.93213	366.09775	98.5095

Supplementary Figure 25. HPLC spectra for racemic and chiral **3v**.

***N*–(2–phenyl–3–(3–(trifluoromethyl)phenyl)propyl)aniline (3w)**: 98% yield, 95% ee, colorless oil.

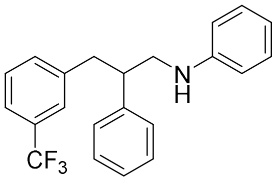

^{1}H NMR(500 MHz, $CDCl_3$): δ 7.41 (d, J = 7.7 Hz, 1H), 7.35–7.25 (m, 4H), 7.25–7.16 (m, 2H), 7.16–7.07 (m, 4H), 6.68 (t, J = 7.3 Hz, 1H), 6.46 (d, J = 7.9 Hz, 2H), 3.44 (dd, J = 12.5, 5.3 Hz, 2H), 3.29 (dd, J = 12.5, 8.8 Hz, 1H), 3.23–3.13 (m, 1H), 3.12–2.94 (m, 2H); ^{13}C NMR (125 MHz, $CDCl_3$): δ 147.78, 141.70, 140.70, 132.41, 130.53 (q, J = 32.0 Hz), 129.22, 128.74, 128.60, 127.82, 127.05, 125.72 (q, J = 3.8 Hz), 125.22, 123.14, 117.62, 113.03, 48.53, 46.92, 40.61. HRMS (ESI) m/z calcd for $C_{22}H_{21}F_3N^+$ $(M+H)^+$ 356.16206, found 356.16229. Enantiomeric excess was determined by chiral HPLC: Chiralpak IB–3 column, Hex/IPA=95:5, 1 mL/min, 220 nm, 10.06 min (minor), 10.72 min (major).

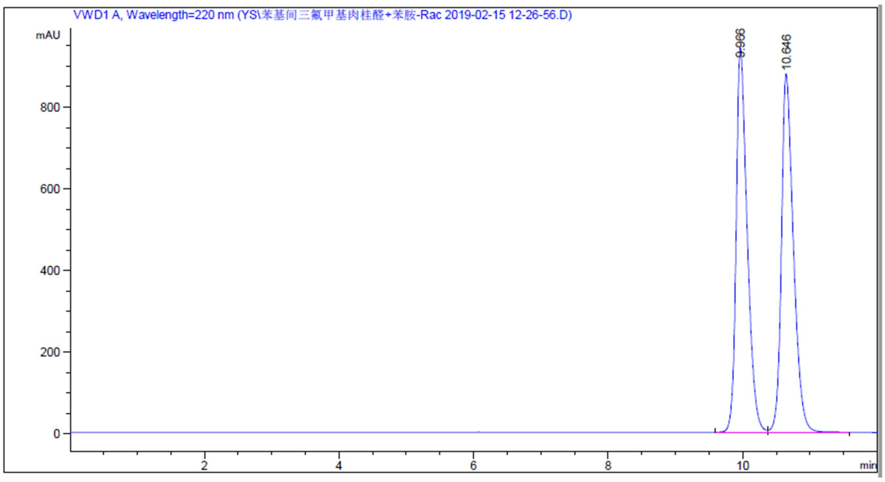

Z

峰 #	保留时间 [min]	类型	峰宽 [min]	峰面积 [mAU*s]	峰高 [mAU]	峰面积 %
1	9.966	BV	0.1700	1.07392e4	942.21100	49.7864
2	10.646	VV	0.1850	1.08313e4	877.53125	50.2136

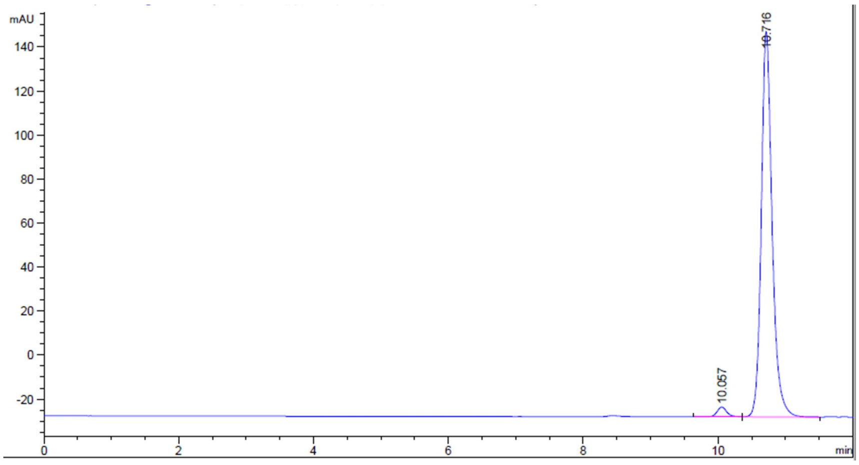

峰 #	保留时间 [min]	类型	峰宽 [min]	峰面积 [mAU*s]	峰高 [mAU]	峰面积 %
1	10.057	BV	0.1565	46.51004	4.50388	2.3198
2	10.716	VB	0.1685	1958.40039	175.08743	97.6802

Supplementary Figure 26. HPLC spectra for racemic and chiral **3w**.

***N*–(3–(3,5–dichlorophenyl)–2–phenylpropyl)aniline (3x)**: 93% yield, 91% ee, colorless oil. ^{1}H NMR(500 MHz, $CDCl_3$): δ 7.31 (t, J = 7.4 Hz, 2H), 7.27–7.20 (m, 1H), 7.18–7.09 (m, 5H), 6.92 (d, J = 1.8 Hz, 2H), 6.69 (t, J = 7.3 Hz, 1H), 6.51–6.44 (m, 2H), 3.42 (dd, J = 12.5, 5.4 Hz, 2H), 3.26 (dd, J = 12.6, 8.7 Hz, 1H), 3.19–3.09 (m, 1H), 3.01–2.83 (m, 2H); ^{13}C NMR (125 MHz, $CDCl_3$): δ 147.66, 143.14, 141.37, 134.58, 129.25, 128.80, 127.74, 127.47, 127.17, 126.39, 117.66, 113.03, 48.51, 46.60, 40.15. HRMS (ESI) m/z calcd for $C_{21}H_{20}Cl_2N^+$ $(M+H)^+$ 356.09673, found 356.09702. Enantiomeric excess was determined by chiral HPLC: Chiralpak IA–3 column, Hex/IPA=98:2, 1 mL/min, 220 nm, 7.45 min (minor), 7.86 min (major).

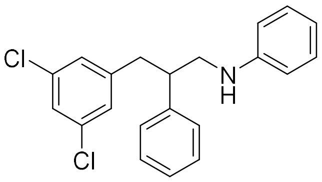

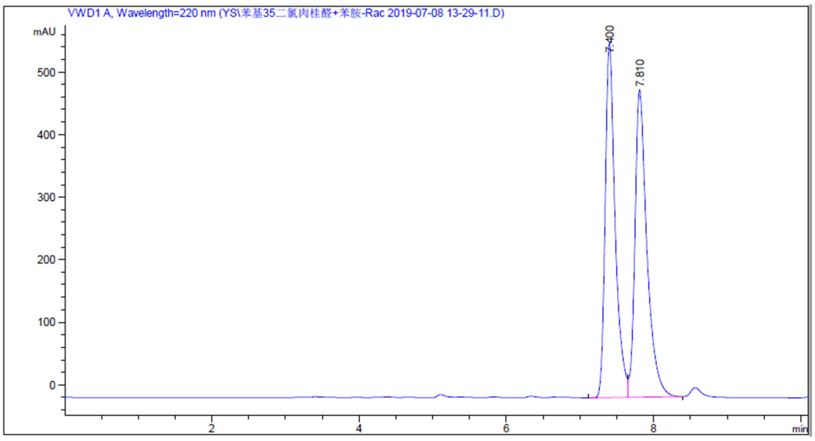

峰 #	保留时间 [min]	类型	峰宽 [min]	峰面积 [mAU*s]	峰高 [mAU]	峰面积 %
1	7.400	BV	0.1421	5339.85889	565.96735	49.0489
2	7.810	VB	0.1678	5546.94775	491.05417	50.9511

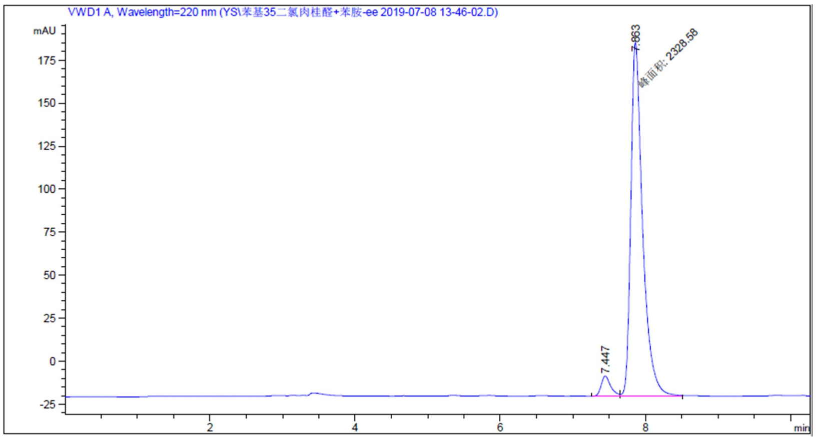

峰 #	保留时间 [min]	类型	峰宽 [min]	峰面积 [mAU*s]	峰高 [mAU]	峰面积 %
1	7.447	BV	0.1381	108.87811	11.75020	4.4669
2	7.863	MF	0.1886	2328.58008	205.77347	95.5331

Supplementary Figure 27. HPLC spectra for racemic and chiral **3x**.

3–(2–chlorophenyl)–*N*–(4–methoxybenzyl)–2–phenylpropan–1–amine (3y): 63% yield, 94% ee, colorless oil. ^{1}H NMR(500 MHz, $CDCl_3$): δ 7.37–7.27 (m, 3H), 7.22 (d, J = 7.3 Hz, 1H), 7.20–7.16 (m, 2H), 7.14–7.09 (m, 1H), 7.08–7.04 (m, 1H), 6.96 (d, J = 7.6 Hz, 1H), 3.72 (s, 3H), 3.47–3.38 (m, 1H), 3.34–3.26 (m, 2H), 3.24–3.17 (m, 1H), 3.03–2.94 (m, 1H); ^{13}C NMR (125 MHz, $CDCl_3$):δ152.15, 142.37, 142.19, 137.57, 134.21, 131.36, 129.57, 128.71, 127.91, 127.68, 126.93, 126.53, 114.85, 114.35, 55.82, 49.37, 45.25, 38.77. HRMS (ESI) m/z calcd for $C_{22}H_{23}ClNO^+$ $(M+H)^+$ 352.14627, found 352.14472. Enantiomeric excess was determined by chiral HPLC: Chiralpak IB–3 column, Hex/IPA=98:2, 1 mL/min, 220 nm, 11.41 min (minor), 12.03 min (major).

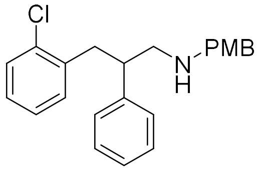

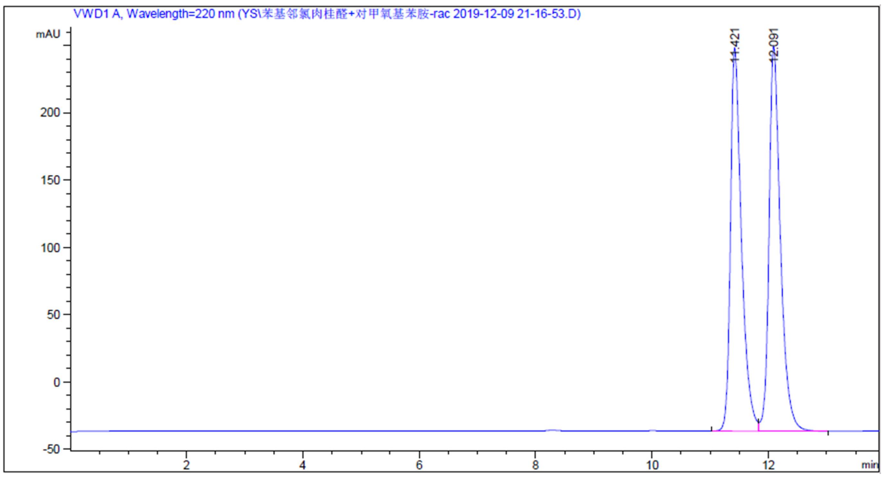

峰 #	保留时间 [min]	类型	峰宽 [min]	峰面积 [mAU*s]	峰高 [mAU]	峰面积 %
1	11.421	BV	0.1974	3745.24170	284.87762	48.9943
2	12.091	VB	0.2030	3898.99023	286.09387	51.0057

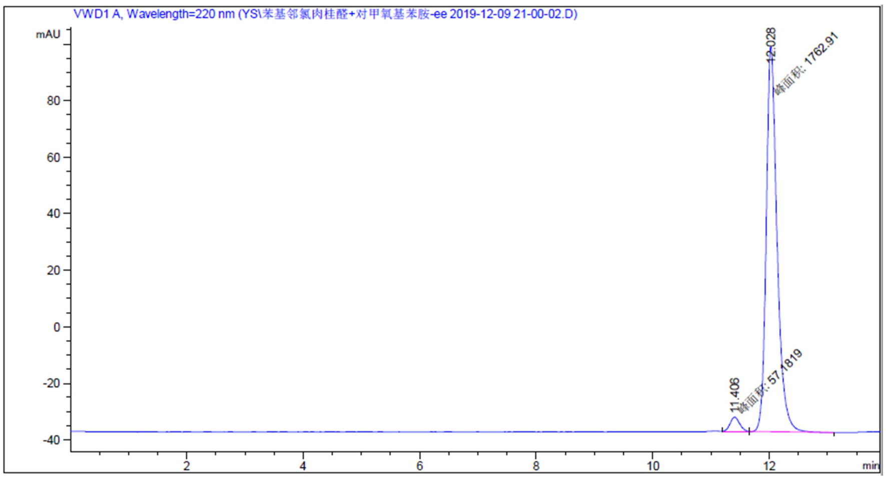

峰 #	保留时间 [min]	类型	峰宽 [min]	峰面积 [mAU*s]	峰高 [mAU]	峰面积 %
1	11.406	MF	0.1890	57.18188	5.04131	3.1417
2	12.028	FM	0.2155	1762.90735	136.31967	96.8583

Supplementary Figure 28. HPLC spectra for racemic and chiral **3y**.

3–(2–fluorophenyl)–*N*–(4–methoxybenzyl)–2–phenylpropan–1–amine (3z): 91% yield, 95% ee, colorless oil. ^{1}H NMR(500 MHz, $CDCl_3$): δ 7.29 (t, J = 7.4 Hz, 2H), 7.26–7.08 (m, 4H), 7.05–6.91 (m, 3H), 6.72 (d, J = 8.9 Hz, 2H), 6.43 (d, J = 8.9 Hz, 2H), 3.42 (dd, J = 11.4, 4.1 Hz, 1H), 3.34–3.16 (m, 2H), 3.01 (ddd, J = 78.1, 13.6, 6.7 Hz, 2H); ^{13}C NMR (125 MHz, $CDCl_3$): δ 162.21, 160.27, 152.18, 142.40, 142.20, 131.36 (d, J = 4.7 Hz), 128.68, 127.94 (d, J = 8.0 Hz), 127.85, 126.92, 123.84 (d, J = 3.5 Hz), 115.34, 115.16, 114.87, 114.39, 99.99, 55.82, 49.49, 45.86, 34.11. HRMS (ESI) m/z calcd for $C_{22}H_{23}FNO^+$ $(M+H)^+$ 336.17582, found 336.17575. Enantiomeric excess was determined by chiral HPLC: Chiralpak IB–3 column, Hex/IPA=98:2, 1 mL/min, 220 nm, 10. 74 min (major), 11. 46 min (minor).

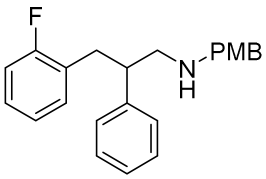

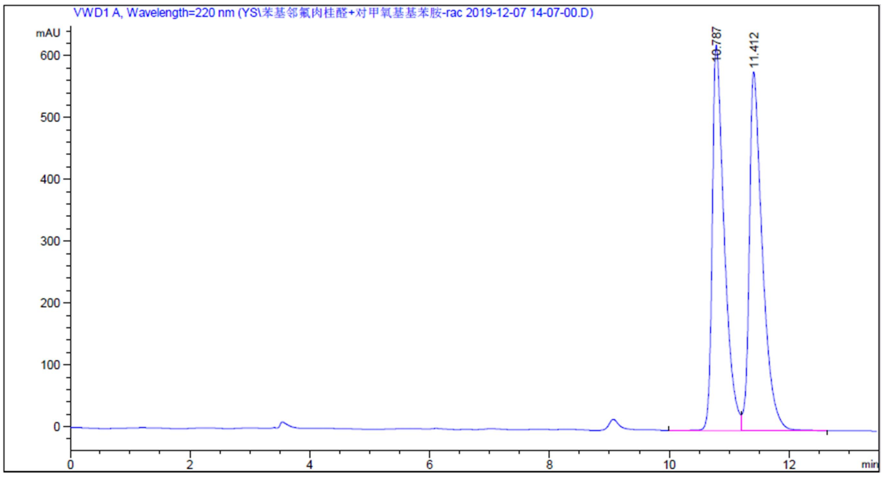

峰 #	保留时间 [min]	类型	峰宽 [min]	峰面积 [mAU*s]	峰高 [mAU]	峰面积 %
1	10.787	BV	0.2046	8689.03516	623.41895	49.5598
2	11.412	VB	0.2238	8843.37695	580.33606	50.4402

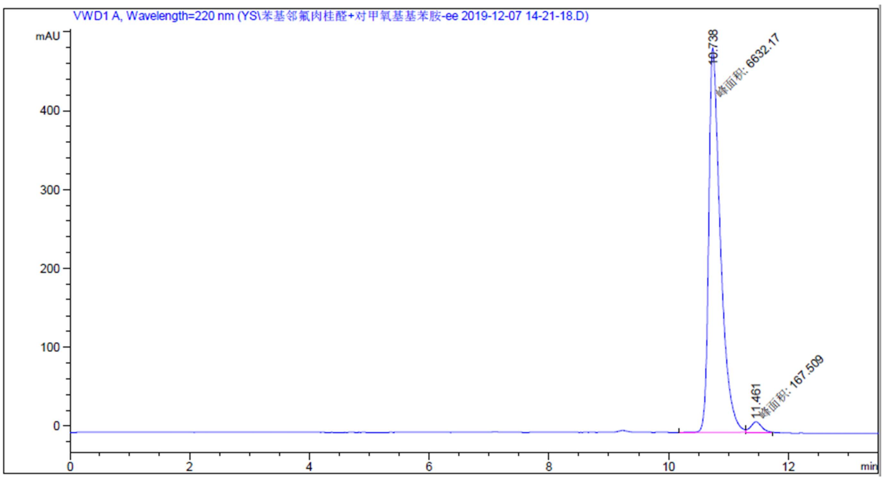

峰 #	保留时间 [min]	类型	峰宽 [min]	峰面积 [mAU*s]	峰高 [mAU]	峰面积 %
1	10.738	MF	0.2270	6632.17139	486.95319	97.5365
2	11.461	FM	0.2098	167.50859	13.30424	2.4635

Supplementary Figure 29. HPLC spectra for racemic and chiral **3z**.

***N*–(3–(furan–2–yl)–2–phenylpropyl)aniline (3za)**: 93% yield, 95% ee, colorless oil.^{1}H NMR(500 MHz, $CDCl_3$): δ 7.35–7.27 (m, 3H), 7.26–7.21 (m, 1H), 7.18 (d, *J* = 7.5 Hz, 2H), 7.12 (t, *J* = 7.7 Hz, 2H), 6.67 (t, *J* = 7.3 Hz, 1H), 6.49 (d, *J* = 7.9 Hz, 2H), 6.24 (t, *J* = 2.4 Hz, 1H), 5.90 (d, *J* = 3.1 Hz, 1H), 3.51 (s, 1H), 3.47 (dd, *J* = 11.7, 4.5 Hz, 1H), 3.35–3.20 (m, 2H), 3.08–2.92 (m, 2H); ^{13}C NMR (125 MHz, $CDCl_3$): δ 153.66, 147.90, 142.33, 141.10, 129.18, 128.67, 127.64, 126.91, 117.40, 112.98, 110.21, 106.49, 48.64, 44.18, 32.95. HRMS (ESI) m/z calcd for $C_{19}H_{20}NO^+$ $(M+H)^+$ 278.15394, found 278.15408. Enantiomeric excess was determined by chiral HPLC: Chiralpak IA–3 column, Hex/IPA=90:10, 1 mL/min, 220 nm, 6.07 min (minor), 7.38 min (major).

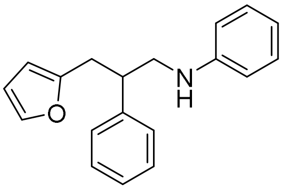

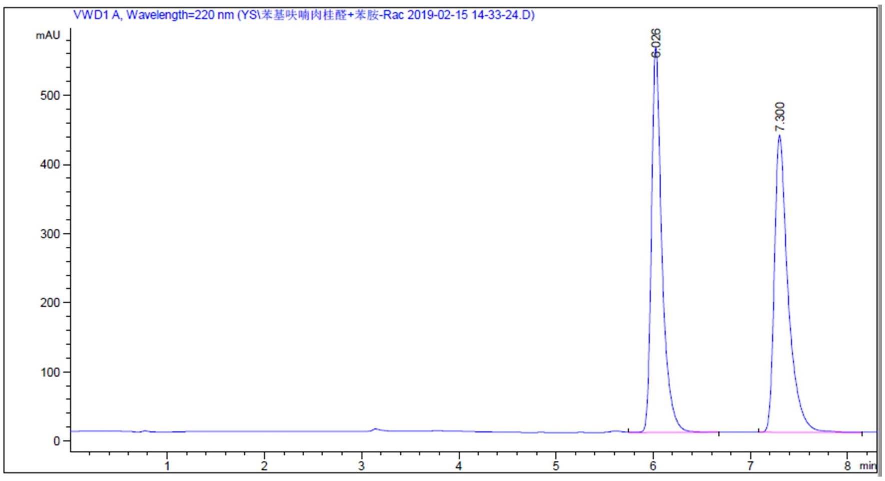

峰 #	保留时间 [min]	类型	峰宽 [min]	峰面积 [mAU*s]	峰高 [mAU]	峰面积 %
1	6.026	VV	0.1116	4179.08398	556.56714	49.7188
2	7.300	BV	0.1447	4226.35156	430.06265	50.2812

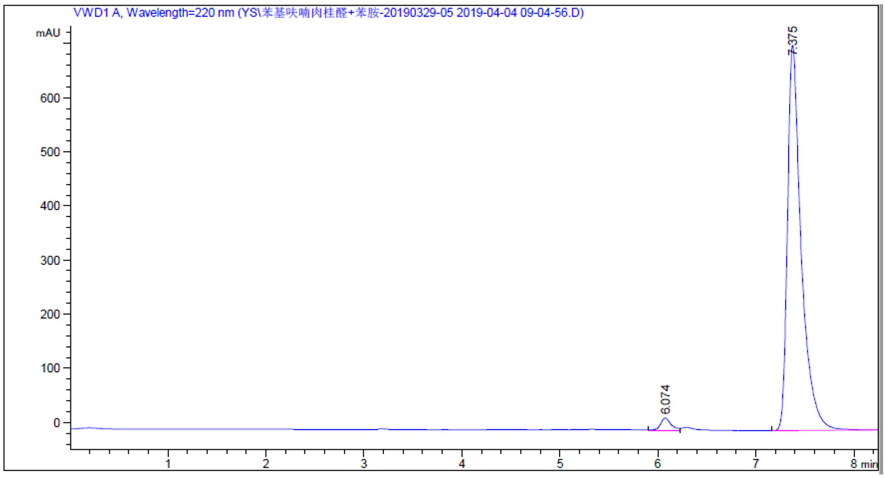

峰 #	保留时间 [min]	类型	峰宽 [min]	峰面积 [mAU*s]	峰高 [mAU]	峰面积 %
1	6.074	VV	0.1081	167.73373	22.71933	2.3639
2	7.375	BBA	0.1437	6927.76758	710.86682	97.6361

Supplementary Figure 30. HPLC spectra for racemic nd chiral **3za**.

***N*-(2-methylpentyl)aniline (3zb)**: 56% yield, 17% ee, colorless oil.^{1}H NMR(500 MHz, $CDCl_3$): δ 7.18 (d, J = 7.5 Hz, 2H), 6.68 (t, J = 7.2 Hz, 1H), 6.60 (d, J = 7.9 Hz, 2H), 3.67 (s, 1H), 3.06 (dd, J = 12.2, 5.9 Hz, 1H), 2.89 (dd, J = 12.2, 7.2 Hz, 1H), 1.80–1.71 (m, 1H), 1.50–1.38 (m, 2H), 1.37–1.29 (m, 1H), 1.23–1.12 (m, 1H), 0.97 (d, J = 6.7 Hz, 3H), 0.92 (t, J = 6.9 Hz, 3H); ^{13}C NMR (125 MHz, $CDCl_3$): δ 148.65, 129.21, 116.92, 112.63, 50.35, 37.09, 32.72, 20.10, 18.04, 14.35. HRMS (ESI) m/z calcd for $C_{12}H_{20}N^+$ $(M+H)^+$ 178.15903, found 178.15909. Enantiomeric excess was determined by chiral HPLC: Chiralpak OJ–3 column, Hex/IPA=99:1, 1 mL/min, 220 nm, 9.33 min (major), 9.72 min (minor).

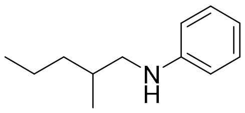

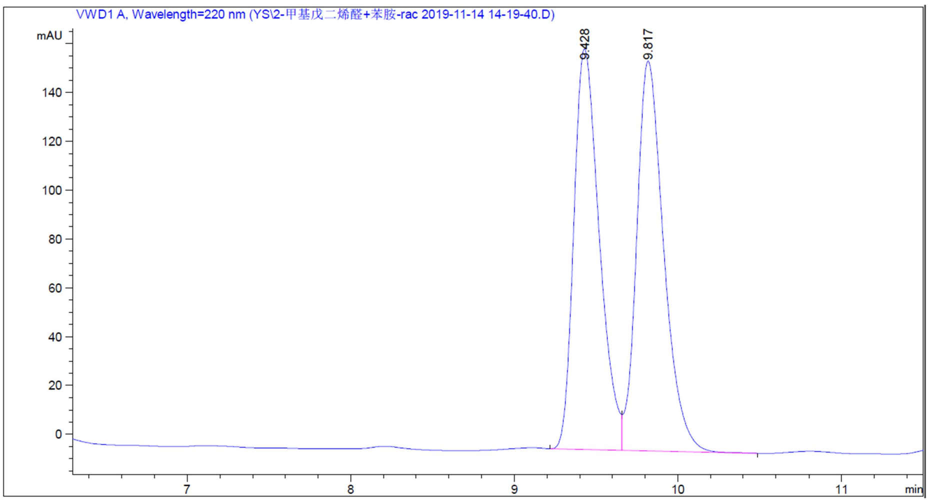

峰 #	保留时间 [min]	类型	峰宽 [min]	峰面积 [mAU*s]	峰高 [mAU]	峰面积 %
1	9.428	BV	0.1665	1780.13843	164.15874	49.1294
2	9.817	VB	0.1766	1843.22949	159.70799	50.8706

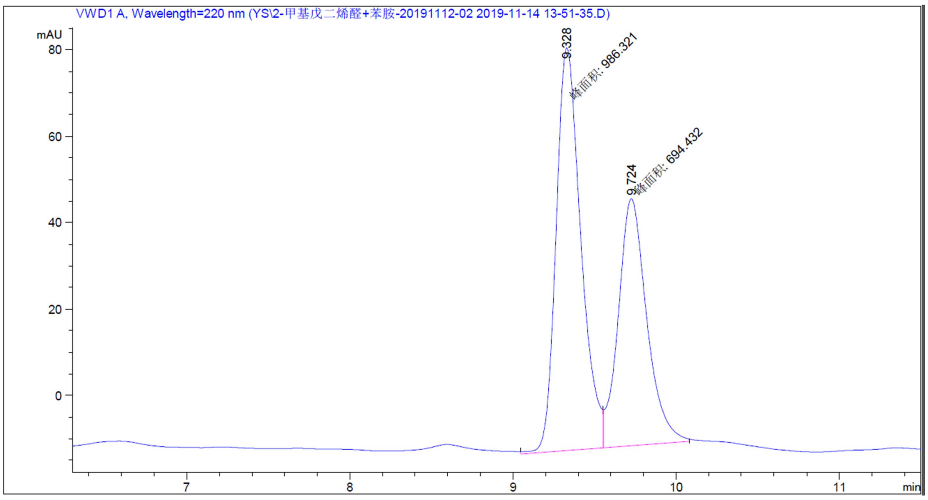

峰 #	保留时间 [min]	类型	峰宽 [min]	峰面积 [mAU*s]	峰高 [mAU]	峰面积 %
1	9.328	MF	0.1764	986.32117	93.21584	58.6833
2	9.724	FM	0.2023	694.43164	57.19940	41.3167

Supplementary Figure 31. HPLC spectra for racemic and chiral **3zb**.

***N*-(3-phenylbutyl)aniline (3zc)**[11]: 34% yield, <5% ee, colorless oil. ^{1}H NMR(500 MHz, $CDCl_3$): δ 7.31 (dd, *J* = 8.2, 7.0 Hz, 2H), 7.23–7.18 (m, 3H), 7.13 (dd, *J* = 8.5, 7.2 Hz, 2H), 6.71–6.63 (m, 1H), 6.50 (d, *J* = 7.5 Hz, 2H), 3.49 (s, 1H), 3.09–2.93 (m, 2H), 2.85 (h, *J* = 7.1 Hz, 1H), 1.98–1.80 (m, 2H), 1.30 (d, *J* = 6.9 Hz, 3H).Enantiomeric excess was determined by chiral HPLC: Chiralpak IB–3 column, Hex/IPA=99:1, 1 mL/min, 220 nm, 15.20 min (minor), 15.90 min (major).

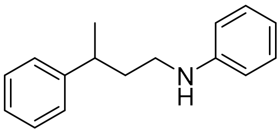

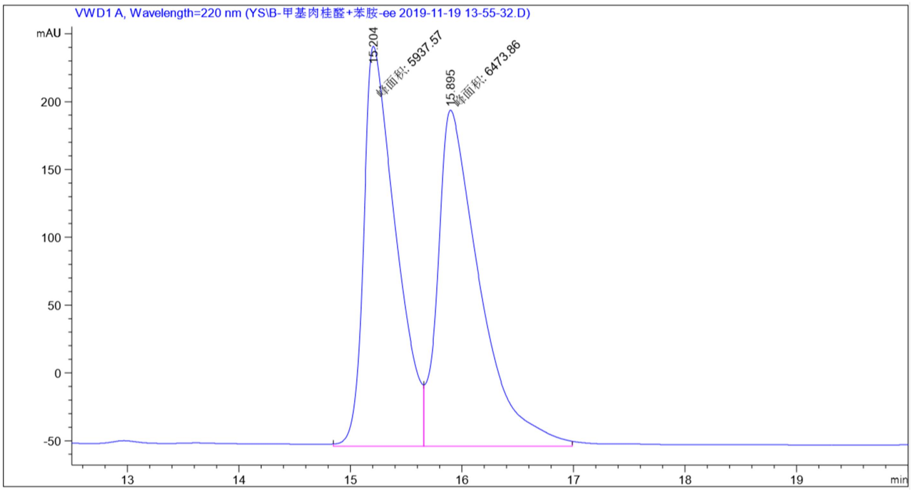

峰 #	保留时间 [min]	类型	峰宽 [min]	峰面积 [mAU*s]	峰高 [mAU]	峰面积 %
1	15.204	MF	0.3355	5937.57373	294.95187	47.8395
2	15.895	FM	0.4353	6473.85938	247.85402	52.1605

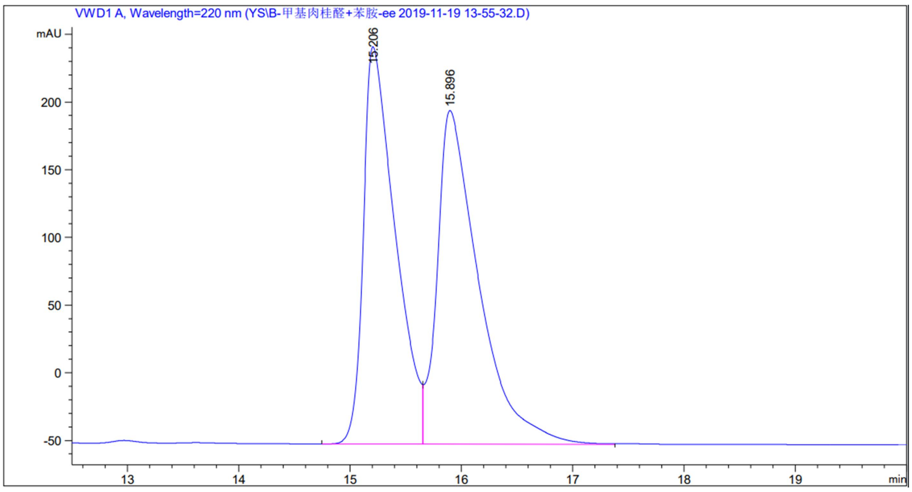

峰 #	保留时间 [min]	类型	峰宽 [min]	峰面积 [mAU*s]	峰高 [mAU]	峰面积 %
1	15.206	BV	0.2906	5851.93359	293.47162	47.7690
2	15.896	VV	0.3750	6398.56299	246.37225	52.2310

Supplementary Figure 32. HPLC spectra for racemic and chiral **3zc**.

***N*-phenethyl-2,3-diphenylpropan-1-amine (3zd)**: 70% yield, 26% ee, colorless oil.^{1}H NMR(500 MHz, $CDCl_3$): δ 7.25–7.10 (m, 11H), 7.06 (d, *J* = 7.5 Hz, 2H), 7.03 (d, *J* = 7.3 Hz, 2H), 3.13–3.03 (m, 1H), 2.98–2.57 (m, 9H). ^{13}C NMR (125 MHz, $CDCl_3$): δ 143.16, 140.20, 140.03, 129.09, 128.62, 128.54, 128.39, 128.17, 127.80, 126.55, 126.04, 125.93, 54.45, 51.16, 47.84, 41.30, 36.27. HRMS (ESI) m/z calcd for $C_{23}H_{26}N^+$ $(M+H)^+$ 316.20598, found 316.20624. Enantiomeric excess was determined by chiral HPLC: Chiralpak OD-H column, Hex/IPA=90:10, DEA 0.1%, TFA 0.2%, 1 mL/min, 220 nm, 8.93 min (minor), 10.17 min (major).

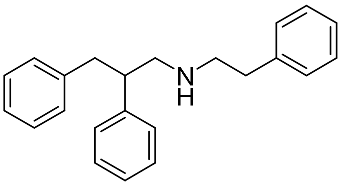

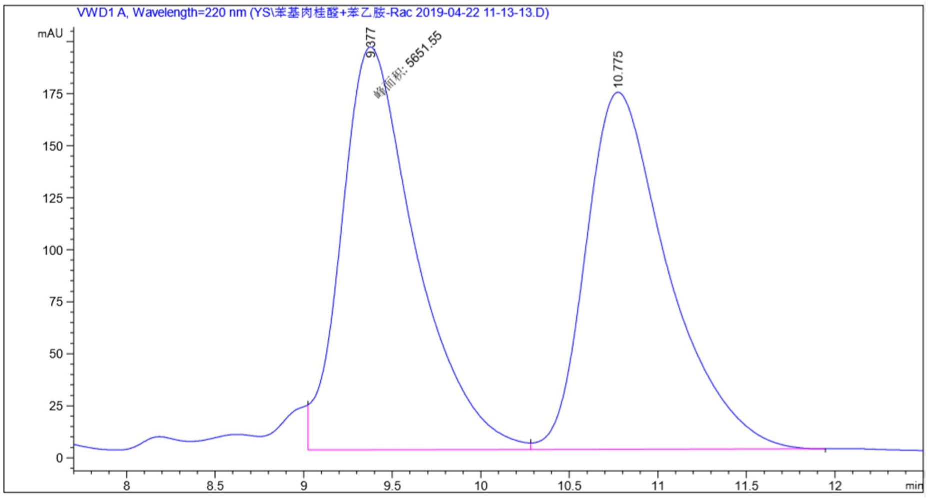

峰 #	保留时间 [min]	类型	峰宽 [min]	峰面积 [mAU*s]	峰高 [mAU]	峰面积 %
1	9.377	FM	0.4870	5651.54883	193.42178	50.4421
2	10.775	VB	0.4837	5552.47314	171.51688	49.5579

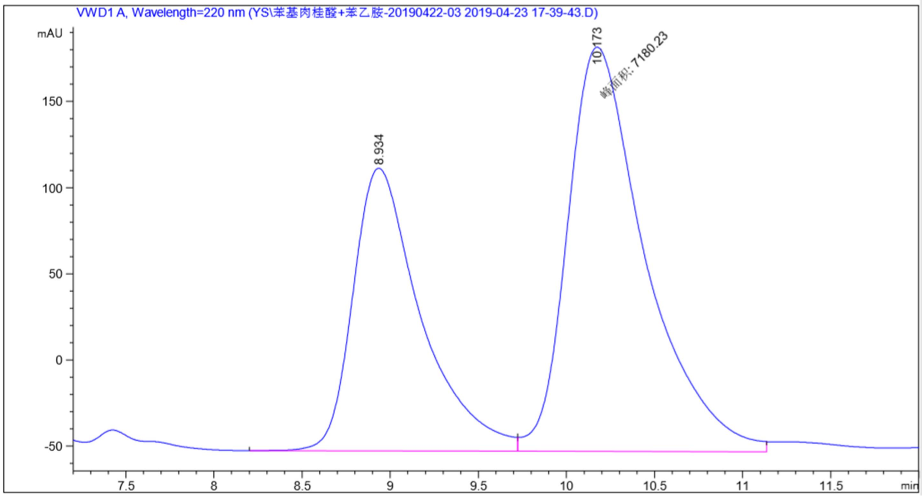

峰 #	保留时间 [min]	类型	峰宽 [min]	峰面积 [mAU*s]	峰高 [mAU]	峰面积 %
1	8.934	BV	0.3950	4399.76611	164.01610	37.9945
2	10.173	MF	0.5103	7180.23486	234.50676	62.0055

Supplementary Figure 33. HPLC spectra for racemic and chiral **3zd**.

***N*–(2,3–diphenylpropyl)–4–methoxyaniline (3ab)**: 93% yield, 98% ee, colorless oil.^{1}H NMR(500 MHz, $CDCl_3$): δ 7.29 (t, *J* = 7.4 Hz, 2H), 7.24–7.19 (m, 3H), 7.18–7.13 (m, 3H), 7.10–7.03 (m, 2H), 6.70 (d, *J* = 8.5 Hz, 2H), 6.39 (d, *J* = 8.6 Hz, 2H), 3.70 (s, 3H), 3.39 (dd, *J* = 11.5, 4.1 Hz, 1H), 3.29–3.08 (m, 3H), 2.97 (dd, *J* = 7.1, 3.3 Hz, 2H); ^{13}C NMR (125 MHz, $CDCl_3$): δ 152.06, 142.67, 142.12, 139.85, 129.01, 128.59, 128.21, 127.82, 126.73, 126.05, 114.77, 114.34, 55.71, 49.51, 46.93, 41.01. HRMS (ESI) m/z calcd for $C_{22}H_{24}NO^+$ $(M+H)^+$ 318.18524, found 318.18527. Enantiomeric excess was determined by chiral HPLC: Chiralpak IB–3 column, Hex/IPA=95:5, 1 mL/min, 220 nm, 9.18 min (major), 9.85 min (minor).

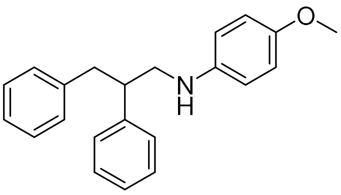

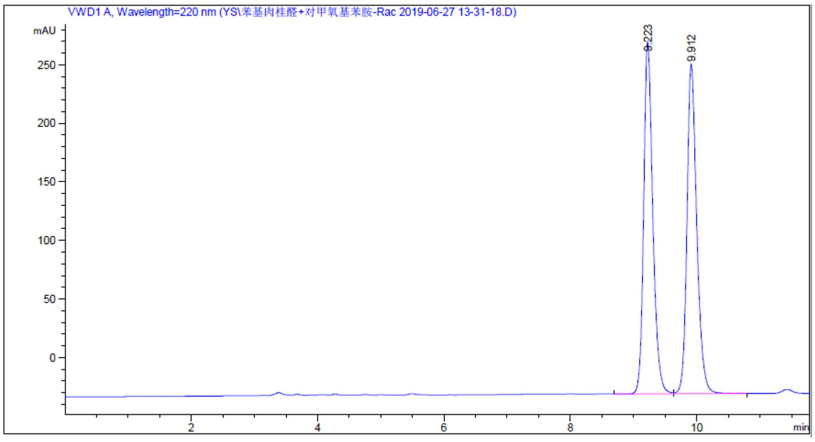

峰 #	保留时间 [min]	类型	峰宽 [min]	峰面积 [mAU*s]	峰高 [mAU]	峰面积 %
1	9.223	VV	0.1509	3011.13843	300.49478	49.8923
2	9.912	VB	0.1615	3024.13696	281.03146	50.1077

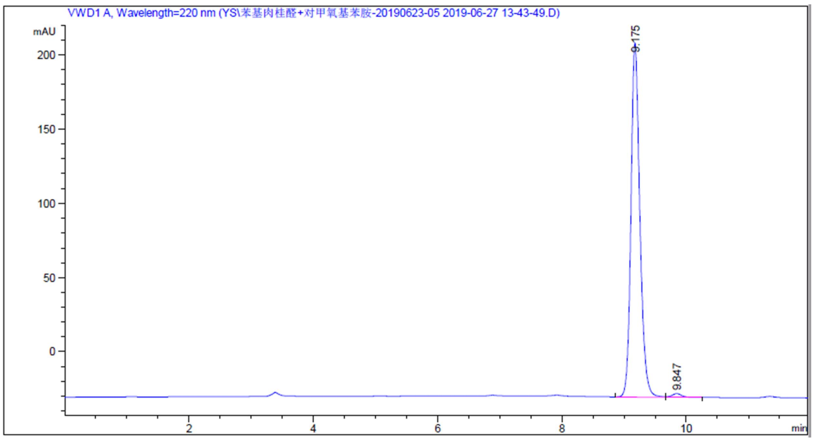

峰 #	保留时间 [min]	类型	峰宽 [min]	峰面积 [mAU*s]	峰高 [mAU]	峰面积 %
1	9.175	BV	0.1518	2367.91138	238.60042	98.8734
2	9.847	VV	0.1628	26.98104	2.48252	1.1266

Supplementary Figure 34. HPLC spectra for racemic and chiral **3ab**.

N–(2,3–diphenylpropyl)–4–methylaniline (3ac): 96% yield, 97% ee, colorless oil. ^{1}H NMR(500 MHz, $CDCl_3$): δ 7.38–7.13 (m, 8H), 7.07 (d, J = 7.7 Hz, 2H), 6.91 (d, J = 8.3 Hz, 2H), 6.35 (d, J = 8.3 Hz, 2H), 3.42 (dd, J = 11.7, 4.5 Hz, 1H), 3.34 (s, 1H), 3.29–3.12 (m, 2H), 3.09–2.90 (m, 2H), 2.20 (s, 3H); ^{13}C NMR (125 MHz, $CDCl_3$): δ 145.66, 142.69, 139.86, 129.62, 129.03, 128.59, 128.22, 127.84, 126.72, 126.54, 126.06, 113.18, 48.89, 46.92, 41.00, 20.33. HRMS (ESI) m/z calcd for $C_{22}H_{24}N^+$ $(M+H)^+$ 302.19033, found 302.19031. Enantiomeric excess was determined by chiral HPLC: Chiralpak IB–3 column, Hex/IPA=95:5, 1 mL/min, 220 nm, 5.83 min (minor), 6.119 min (major).

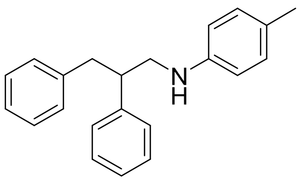

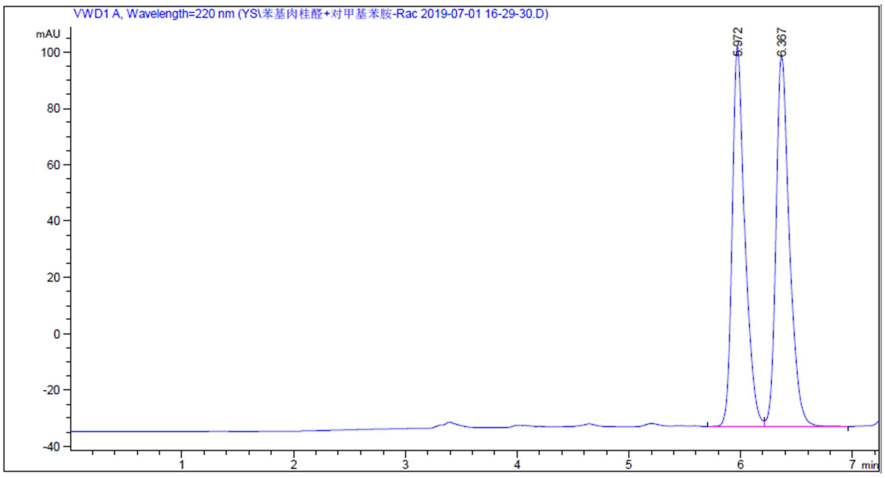

峰 #	保留时间 [min]	类型	峰宽 [min]	峰面积 [mAU*s]	峰高 [mAU]	峰面积 %
1	5.972	BV	0.1167	1072.30322	134.90921	49.4613
2	6.367	VB	0.1230	1095.65918	131.75912	50.5387

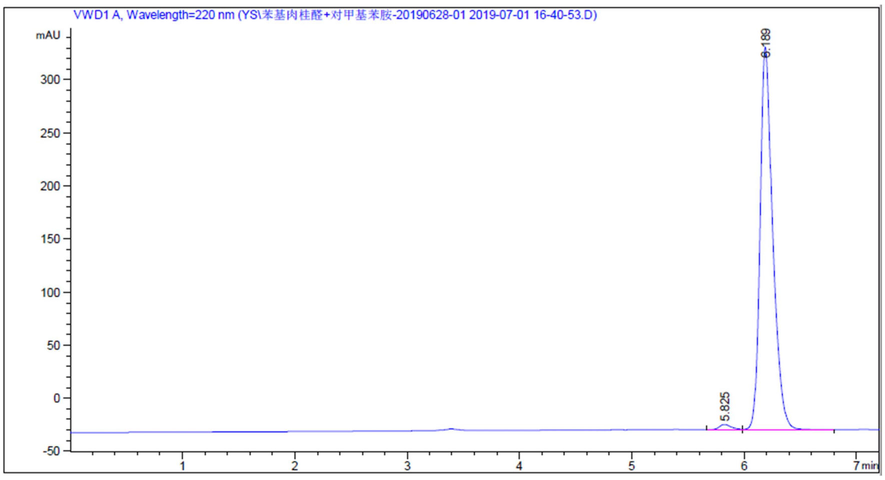

峰 #	保留时间 [min]	类型	峰宽 [min]	峰面积 [mAU*s]	峰高 [mAU]	峰面积 %
1	5.825	BV	0.1093	36.34295	4.97291	1.2624
2	6.189	VB	0.1180	2842.46240	360.44916	98.7376

Supplementary Figure 35. HPLC spectra for racemic and chiral **3ac**.

***N*–(2,3–diphenylpropyl)–4–fluoroaniline (3ad)**: 98% yield, 98% ee, colorless oil. ^{1}H NMR(500 MHz, $CDCl_3$): δ 7.30 (t, *J* = 7.4 Hz, 2H), 7.23 (dd, *J* = 7.4, 4.9 Hz, 3H), 7.19–7.13 (m, 3H), 7.10–7.05 (m, 2H), 6.80 (t, *J* = 8.7 Hz, 2H), 6.37–6.28 (m, 2H), 3.39 (dd, *J* = 11.8, 4.5 Hz, 1H), 3.33 (s, 1H), 3.25–3.09 (m, 2H), 2.97 (d, *J* = 7.1 Hz, 2H); ^{13}C NMR (125 MHz, $CDCl_3$): δ 156.68, 154.82, 144.24, 142.53, 139.76, 129.01, 128.67, 128.29, 127.79, 126.85, 126.15, 115.61, 115.43, 113.82, 113.77, 49.14, 46.89, 41.01. HRMS (ESI) m/z calcd for $C_{21}H_{21}FN^+$ $(M+H)^+$ 306.16525, found 306.16510. Enantiomeric excess was determined by chiral HPLC: Chiralpak IA–3 column, Hex/IPA=90:10, 1 mL/min, 220 nm, 5.81 min (minor), 7.65 min (major).

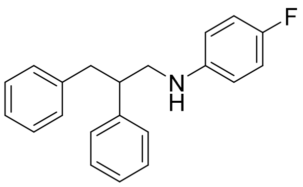

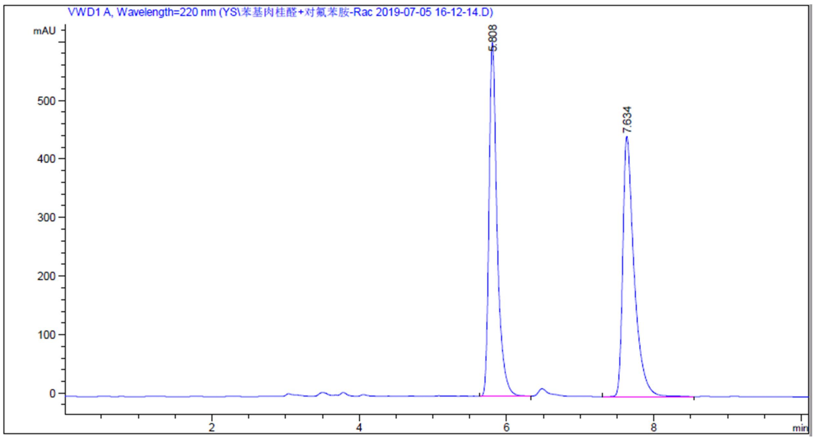

峰 #	保留时间 [min]	类型	峰宽 [min]	峰面积 [mAU*s]	峰高 [mAU]	峰面积 %
1	5.808	BV	0.1159	4653.00244	603.90485	49.4571
2	7.634	BV	0.1570	4755.16504	443.92905	50.5429

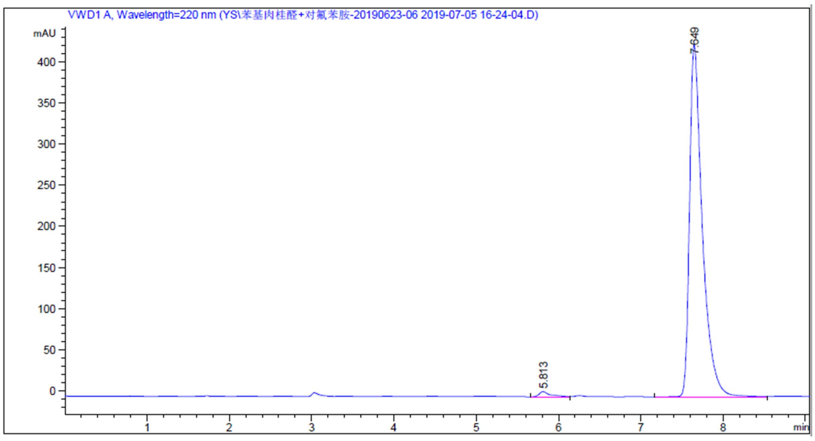

峰 #	保留时间 [min]	类型	峰宽 [min]	峰面积 [mAU*s]	峰高 [mAU]	峰面积 %
1	5.813	BV	0.1336	58.61099	6.23826	1.2488
2	7.649	BV	0.1583	4634.95020	428.10815	98.7512

Supplementary Figure 36. HPLC spectra for racemic and chiral **3ad**.

***N*–(2,3–diphenylpropyl)–3–methylaniline (3ae)**: 98% yield, 98% ee, colorless oil.^{1}H NMR(500 MHz, $CDCl_3$): δ 7.34–7.25 (m, 2H), 7.26–7.19 (m, 3H), 7.19–7.12 (m, 3H), 7.08 (d, J = 7.4 Hz, 2H), 6.99 (t, J = 7.7 Hz, 1H), 6.48 (d, J = 7.5 Hz, 1H), 6.27–6.17 (m, 2H), 3.43 (dd, J = 12.1, 4.8 Hz, 2H), 3.28–3.10 (m, 2H), 3.03–2.91 (m, 2H), 2.20 (s, 3H); ^{13}C NMR (125 MHz, $CDCl_3$): δ 147.93, 142.71, 139.86, 138.85, 129.05, 128.98, 128.60, 128.24, 127.82, 126.74, 126.08, 118.28, 113.68, 110.16, 48.46, 46.95, 40.94, 21.53. HRMS (ESI) m/z calcd for $C_{22}H_{24}N^{+}$ $(M+H)^{+}$ 302.19033, found 302.19034. Enantiomeric excess was determined by chiral HPLC: Chiralpak IA–3 column, Hex/IPA=90:10, 1 mL/min, 220 nm, 4.92 min (minor), 5.67 min (major).

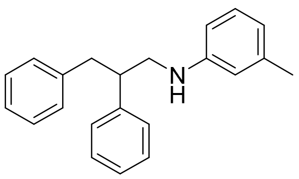

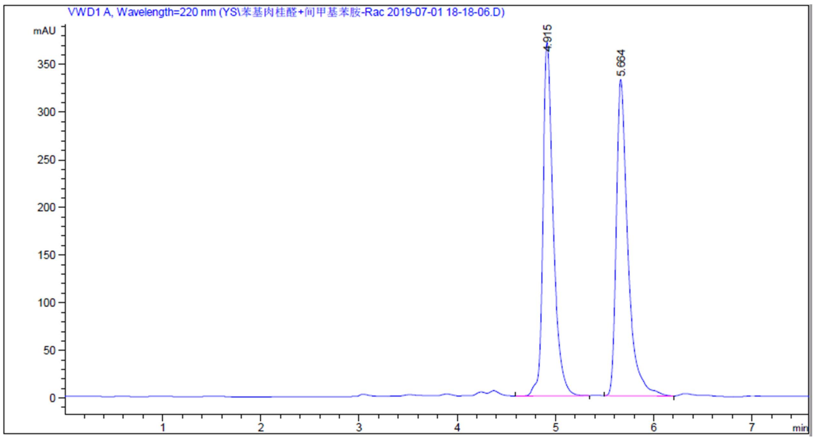

峰 #	保留时间 [min]	类型	峰宽 [min]	峰面积 [mAU*s]	峰高 [mAU]	峰面积 %
1	4.915	BV	0.1093	2711.49365	370.76077	49.8140
2	5.664	BV	0.1221	2731.74292	331.65561	50.1860

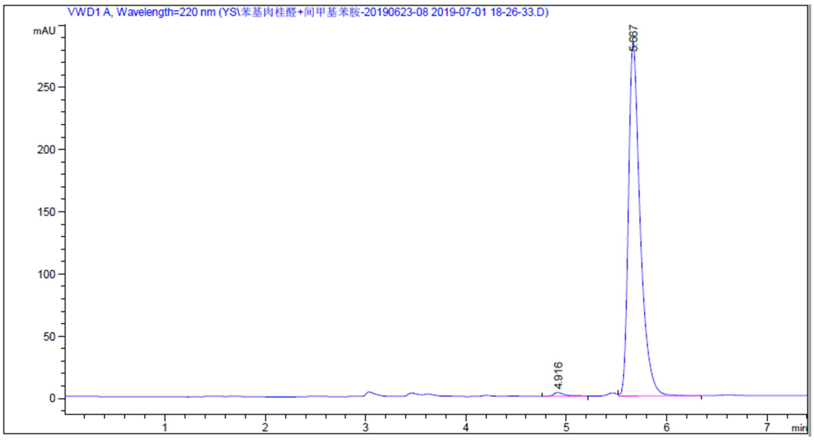

峰 #	保留时间 [min]	类型	峰宽 [min]	峰面积 [mAU*s]	峰高 [mAU]	峰面积 %
1	4.916	BB	0.1120	22.65114	2.96972	0.9737
2	5.667	VB	0.1215	2303.65649	284.39969	99.0263

Supplementary Figure 37. HPLC spectra for racemic and chiral **3ae**.

N–(2,3–diphenylpropyl)–3–(trifluoromethyl)aniline (3af): 98% yield, 95% ee, colorless oil. ^{1}H NMR(500 MHz, $CDCl_3$): δ 7.30 (t, J = 7.4 Hz, 2H), 7.27–7.12 (m, 7H), 7.09 (d, J = 7.4 Hz, 2H), 6.87 (d, J = 7.6 Hz, 1H), 6.59 (s, 1H), 6.51 (dd, J = 8.2, 2.3 Hz, 1H), 3.63 (s, 1H), 3.44 (dd, J = 12.4, 4.9 Hz, 1H), 3.26 (dd, J = 12.4, 9.0 Hz, 1H), 3.21–3.12 (m, 1H), 3.05–2.90 (m, 2H).; ^{13}C NMR (125 MHz, $CDCl_3$): δ148.03, 142.31, 139.60, 131.45 (q, J = 31.7 Hz), 129.48, 128.97, 128.76, 128.39, 127.76, 126.99, 126.28, 125.39, 123.23, 115.90, 113.66 (q, J = 4.0 Hz), 108.94 (q, J = 3.9 Hz), 48.18, 46.83, 40.94. HRMS (ESI) m/z calcd for $C_{22}H_{21}F_3N^+$ $(M+H)^+$ 356.16206, found 356.16202. Enantiomeric excess was determined by chiral HPLC: Chiralpak IA–3 column, Hex/IPA=98:2, 1 mL/min, 220 nm, 6.53 min (minor), 7.45 min (major).

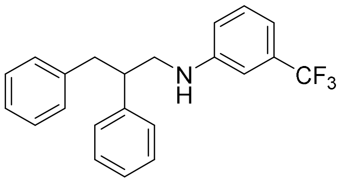

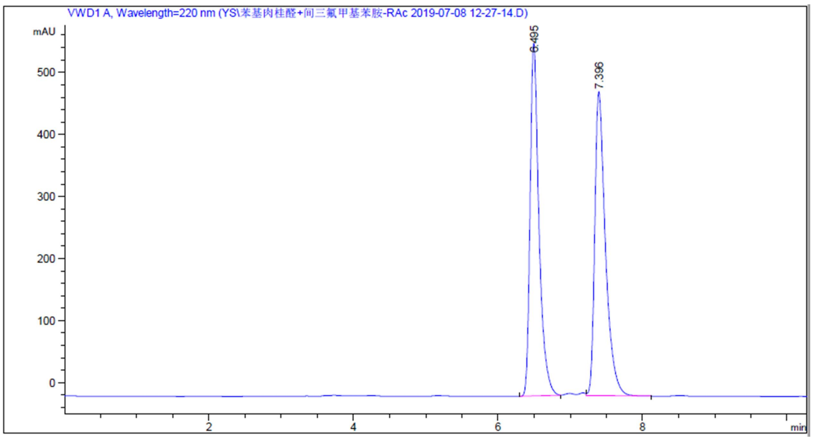

峰 #	保留时间 [min]	类型	峰宽 [min]	峰面积 [mAU*s]	峰高 [mAU]	峰面积 %
1	6.495	BB	0.1276	4857.46875	568.76630	49.1170
2	7.396	VB	0.1539	5032.11475	489.67462	50.8830

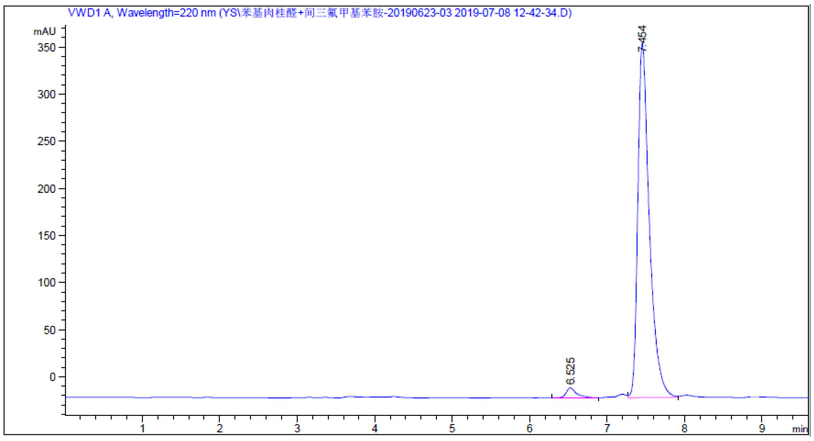

峰 #	保留时间 [min]	类型	峰宽 [min]	峰面积 [mAU*s]	峰高 [mAU]	峰面积 %
1	6.525	VB	0.1343	96.02958	10.43823	2.3974
2	7.454	VV	0.1551	3909.54980	376.52710	97.6026

Supplementary Figure 38. HPLC spectra for racemic and chiral **3af**.

***N*–(2,3–diphenylpropyl)–3,5–dimethylaniline (3ag)**: 95% yield, 96% ee, colorless oil.^{1}H NMR(500 MHz, $CDCl_3$): δ 7.29 (t, *J* = 7.5 Hz, 2H), 7.26–7.14 (m, 6H), 7.13–7.05 (m, 2H), 6.32 (s, 1H), 6.05 (s, 2H), 3.43 (dd, *J* = 12.0, 4.5 Hz, 1H), 3.38 (s, 1H), 3.27–3.09 (m, 2H), 2.97 (dd, *J* = 7.2, 2.1 Hz, 2H), 2.17 (s, 6H); ^{13}C NMR (125 MHz, $CDCl_3$): δ 148.00, 142.82, 139.92, 138.73, 129.08, 128.59, 128.25, 127.83, 126.72, 126.08, 119.36, 110.91, 48.46, 46.99, 40.92, 21.41. HRMS (ESI) m/z calcd for $C_{23}H_{26}N^+$ $(M+H)^+$ 316.20598, found 316.20578. Enantiomeric excess was determined by chiral HPLC: Chiralpak IB–3 column, Hex/IPA=95:5, 1 mL/min, 220 nm, 5.59 min (major), 6.69 min (minor).

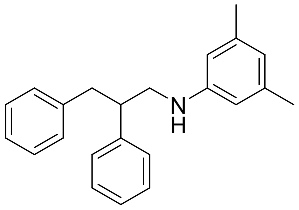

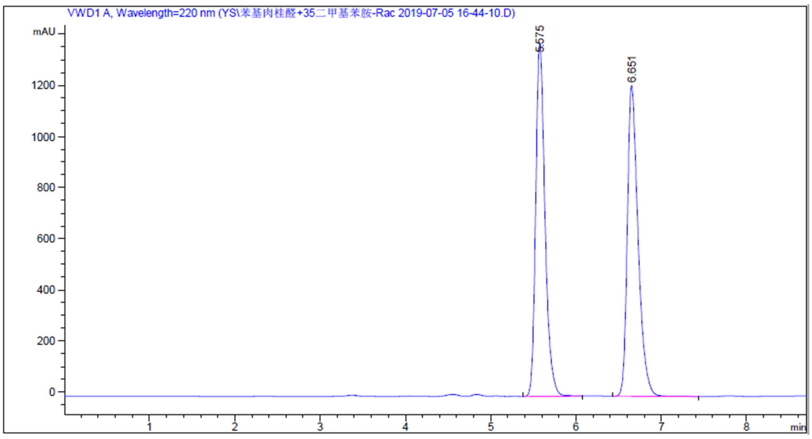

峰 #	保留时间 [min]	类型	峰宽 [min]	峰面积 [mAU*s]	峰高 [mAU]	峰面积 %
1	5.575	BV	0.1165	1.04815e4	1381.78674	49.6330
2	6.651	BB	0.1321	1.06365e4	1215.89832	50.3670

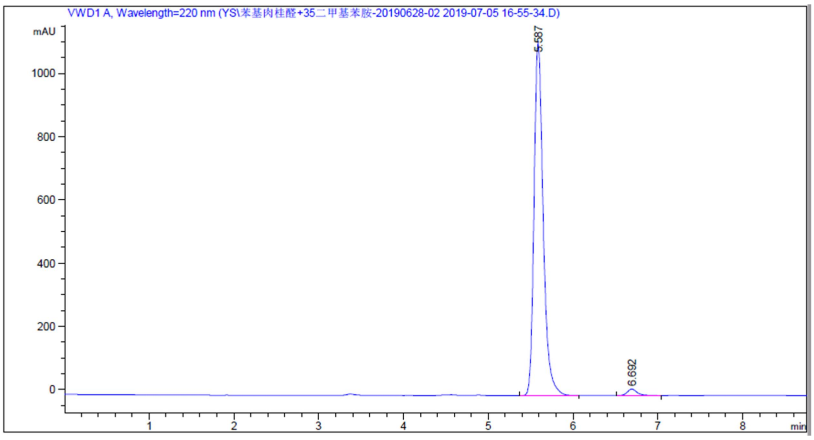

峰 #	保留时间 [min]	类型	峰宽 [min]	峰面积 [mAU*s]	峰高 [mAU]	峰面积 %
1	5.587	BV	0.1148	8385.06445	1114.56799	98.0653
2	6.692	VB	0.1230	165.42299	20.32131	1.9347

Supplementary Figure 39. HPLC spectra for racemic and chiral **3ag**.

***N*–(2,3–diphenylpropyl)–2–methylaniline (3ah)**: 96% yield, 97% ee, colorless oil.^{1}H NMR(500 MHz, $CDCl_3$): δ 7.29 (t, *J* = 7.5 Hz, 2H), 7.26–7.14 (m, 6H), 7.11 (d, *J* = 7.4 Hz, 2H), 7.08–7.02 (m, 1H), 6.96 (d, *J* = 7.2 Hz, 1H), 6.61 (t, *J* = 7.3 Hz, 1H), 6.48 (d, *J* = 8.0 Hz, 1H), 3.49 (dd, *J* = 10.0, 6.6 Hz, 1H), 3.32 (s, 1H), 3.28–3.19 (m, 2H), 3.02 (d, *J* = 6.2 Hz, 2H), 1.80 (s, 3H); ^{13}C NMR (125 MHz, $CDCl_3$): δ 145.86, 142.67, 139.86, 129.95, 129.02, 128.65, 128.29, 127.76, 127.00, 126.83, 126.13, 122.10, 116.88, 109.83, 48.61, 46.85, 40.83, 17.01. HRMS (ESI) m/z calcd for $C_{22}H_{24}N^+$ $(M+H)^+$ 302.19033, found 302.19034. Enantiomeric excess was determined by chiral HPLC: Chiralpak IB–3 column, Hex/IPA=95:5, 1 mL/min, 220 nm, 6.40 min (minor), 7.50 min (major).

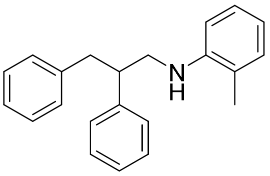

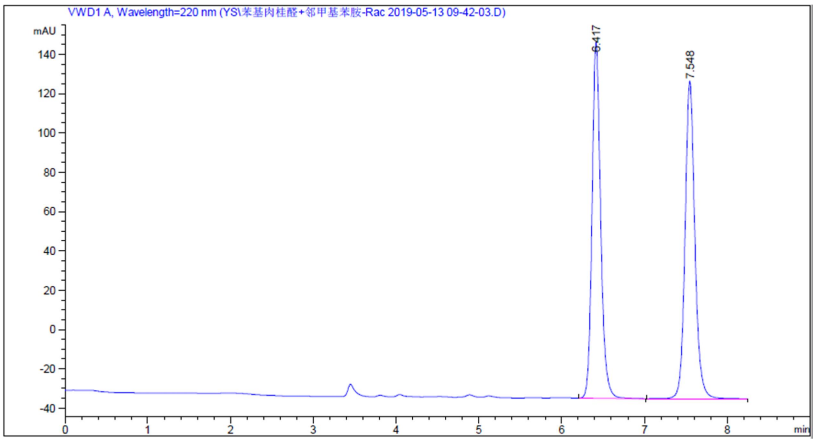

峰 #	保留时间 [min]	类型	峰宽 [min]	峰面积 [mAU*s]	峰高 [mAU]	峰面积 %
1	6.417	VB	0.1105	1311.97095	181.12910	49.6629
2	7.548	BBA	0.1261	1329.78149	161.48921	50.3371

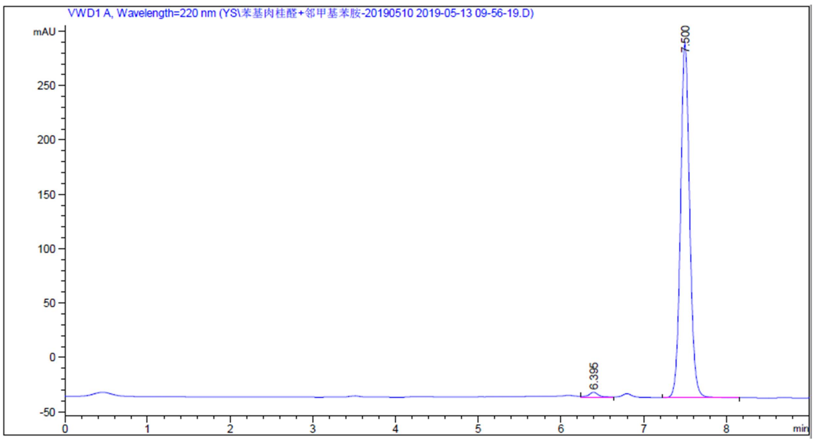

峰 #	保留时间 [min]	类型	峰宽 [min]	峰面积 [mAU*s]	峰高 [mAU]	峰面积 %
1	6.395	VB	0.1143	33.24929	4.39539	1.3083
2	7.500	BB	0.1178	2508.10645	325.97748	98.6917

Supplementary Figure 40. HPLC spectra for racemic and chiral **3ah**.

2–((2,3–diphenylpropyl)amino)phenol (3ai): 93% yield, 97% ee, brown oil.^{1}H NMR(500 MHz, $CDCl_3$): δ 7.31–7.11 (m, 9H), 7.05 (d, *J* = 7.4 Hz, 2H), 6.77 (t, *J* = 7.6 Hz, 1H), 6.63–6.51 (m, 2H), 3.41 (dd, *J* = 12.2, 5.2 Hz, 1H), 3.26 (dd, *J* = 12.2, 8.5 Hz, 1H), 3.19 (p, *J* = 7.1 Hz, 1H), 3.07–2.90 (m, 2H); ^{13}C NMR (125 MHz, $CDCl_3$): δ 144.02, 142.63, 139.84, 136.72, 129.04, 128.54, 128.18, 127.81, 126.68, 126.01, 121.32, 117.93, 114.42, 112.73, 49.11, 47.04, 40.75. HRMS (ESI) m/z calcd for $C_{21}H_{22}NO^+$ $(M+H)^+$ 304.16959, found 304.16949. Enantiomeric excess was determined by chiral HPLC: Chiralpak IA–3 column, Hex/IPA=90:10, 1 mL/min, 220 nm, 16.93 min (major), 18.00 min (minor).

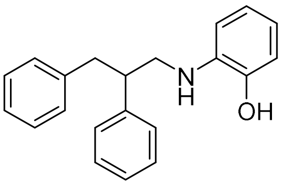

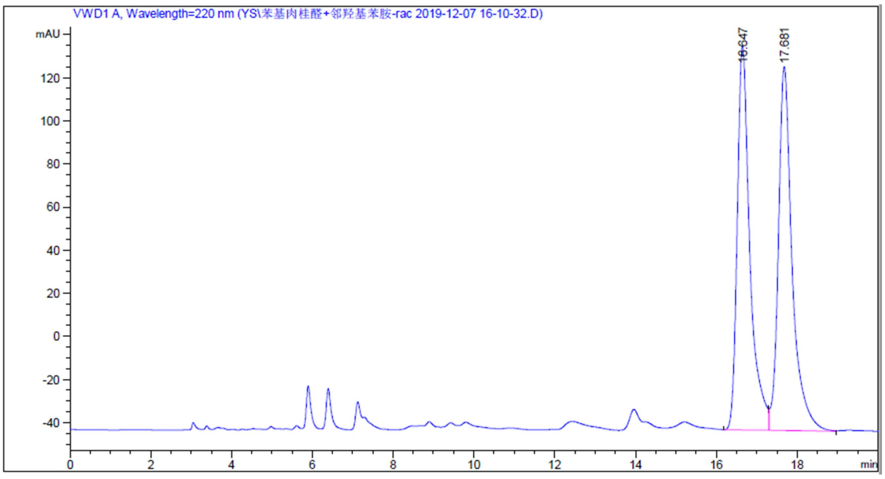

峰 #	保留时间 [min]	类型	峰宽 [min]	峰面积 [mAU*s]	峰高 [mAU]	峰面积 %
1	16.647	BV	0.3240	3879.57593	178.17125	49.2528
2	17.681	VV	0.3499	3997.29199	169.02762	50.7472

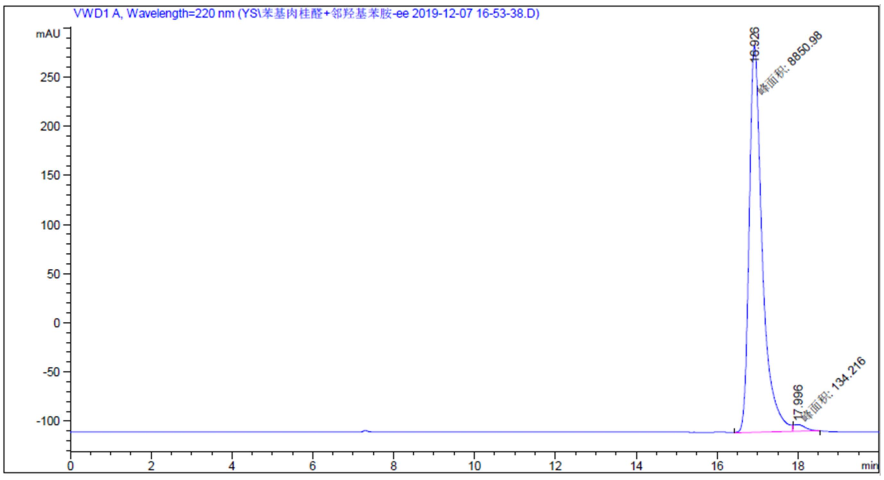

峰 #	保留时间 [min]	类型	峰宽 [min]	峰面积 [mAU*s]	峰高 [mAU]	峰面积 %
1	16.926	MF	0.3755	8850.97559	392.81287	98.5062
2	17.996	FM	0.3355	134.21637	6.66814	1.4938

Supplementary Figure 41. HPLC spectra for racemic and chiral **3ai**.

2–chloro–*N*–(2,3–diphenylpropyl)aniline (3aj): 88% yield, 99% ee, colorless oil. ^{1}H NMR(500 MHz, $CDCl_3$): δ 7.31 (t, *J* = 7.5 Hz, 2H), 7.26–7.13 (m, 7H), 7.10 (d, *J* = 7.5 Hz, 2H), 7.05 (t, *J* = 7.7 Hz, 1H), 6.57 (t, *J* = 7.6 Hz, 1H), 6.48 (d, *J* = 8.1 Hz, 1H), 4.21 (s, 1H), 3.52–3.39 (m, 1H), 3.33–3.15 (m, 2H), 3.03 (d, *J* = 7.3 Hz, 2H); ^{13}C NMR (125 MHz, $CDCl_3$): δ143.72, 142.31, 139.71, 129.04, 128.69, 128.31, 127.74, 127.66, 126.90, 126.18, 119.28, 117.08, 111.24, 48.25, 46.84, 40.47. HRMS (ESI) m/z calcd for $C_{21}H_{21}ClN^+$ $(M+H)^+$ 322.13570, found 322.13568. Enantiomeric excess was determined by chiral HPLC: Chiralpak IB–3 column, Hex/IPA=99:1, 1 mL/min, 220 nm, 6.04 min (minor), 6.25 min (major).

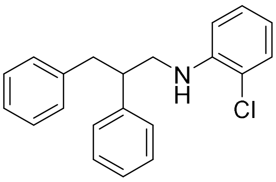

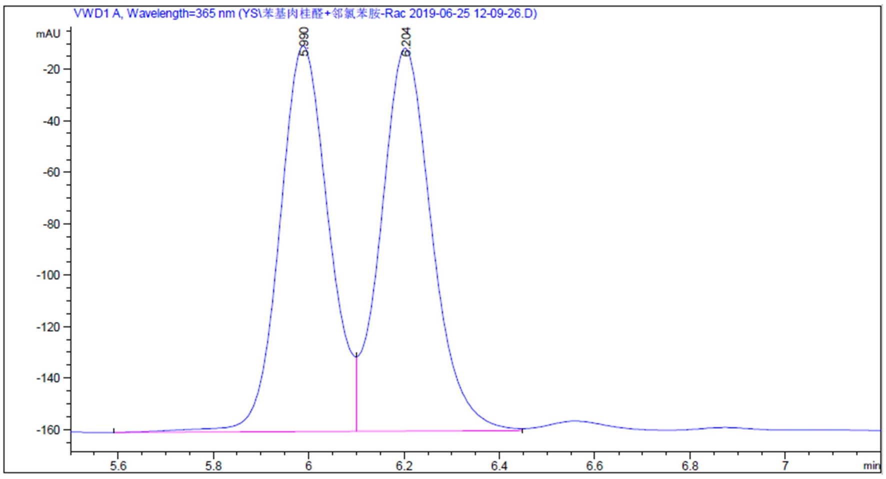

峰 #	保留时间 [min]	类型	峰宽 [min]	峰面积 [mAU*s]	峰高 [mAU]	峰面积 %
1	5.990	BV	0.1091	1068.76929	149.98941	49.0297
2	6.204	VV	0.1140	1111.07312	148.90907	50.9703

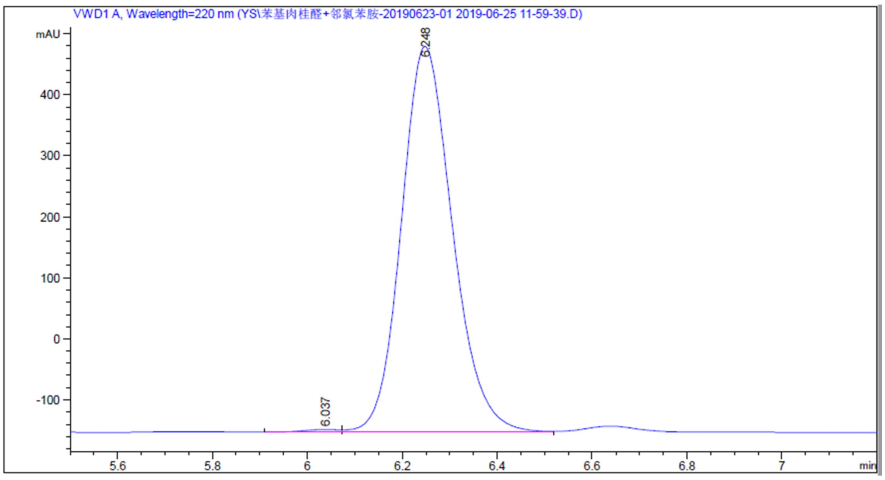

峰 #	保留时间 [min]	类型	峰宽 [min]	峰面积 [mAU*s]	峰高 [mAU]	峰面积 %
1	6.037	BV	0.0797	22.78313	4.45034	0.4782
2	6.248	VV	0.1155	4741.33984	631.84583	99.5218

Supplementary Figure 42. HPLC spectra for racemic and chiral **3aj**.

***N*–(2,3–diphenylpropyl)naphthalen–1–amine (3ak)**: 95% yield, 97% ee, colorless oil. ^{1}H NMR(500 MHz, $CDCl_3$): δ 7.72 (d, *J* = 8.1 Hz, 1H), 7.41–7.08 (m, 15H), 6.47 (d, *J* = 7.5 Hz, 1H), 4.18 (s, 1H), 3.58 (dd, *J* = 11.1, 4.4 Hz, 1H), 3.44–3.28 (m, 2H), 3.07 (d, *J* = 6.8 Hz, 2H); ^{13}C NMR (125 MHz, $CDCl_3$): δ 143.11, 142.66, 139.84, 134.22, 129.08, 128.77, 128.50, 128.36, 127.78, 126.95, 126.52, 126.18, 125.58, 124.52, 123.41, 119.66, 117.31, 104.35, 48.75, 46.88, 41.06. HRMS (ESI) m/z calcd for $C_{25}H_{24}N^{+}$ $(M+H)^{+}$ 338.19033, found 338.19031. Enantiomeric excess was determined by chiral HPLC: Chiralpak IB–3 column, Hex/IPA=90:10, 1 mL/min, 220 nm, 9.15 min (minor), 11.56 min (major).

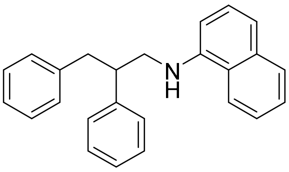

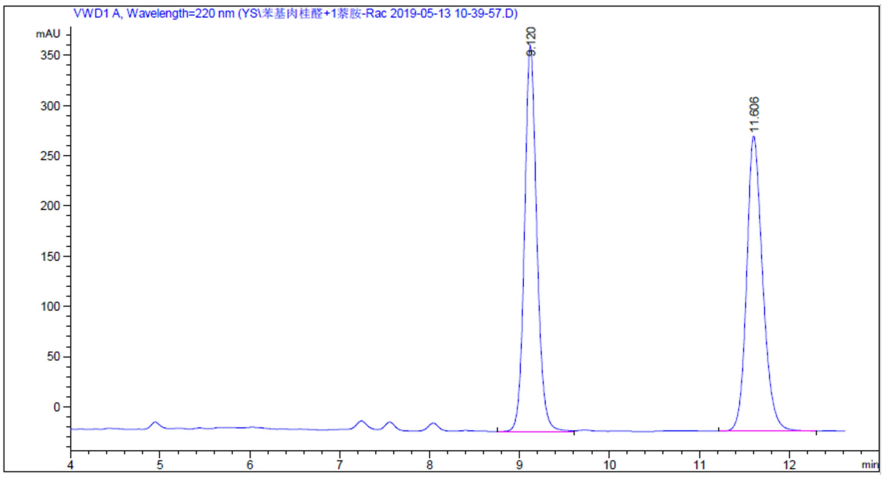

峰 #	保留时间 [min]	类型	峰宽 [min]	峰面积 [mAU*s]	峰高 [mAU]	峰面积 %
1	9.120	BV	0.1452	3654.87012	383.45068	49.9773
2	11.606	BV	0.1873	3658.18970	293.81552	50.0227

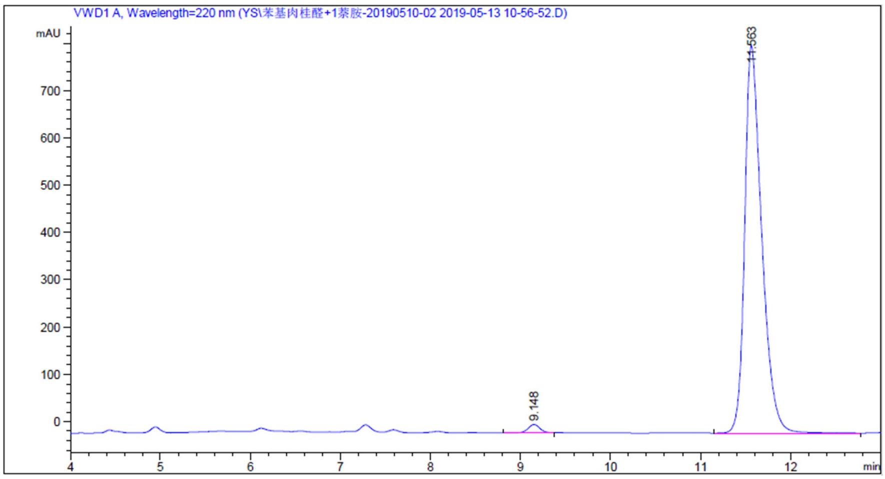

峰 #	保留时间 [min]	类型	峰宽 [min]	峰面积 [mAU*s]	峰高 [mAU]	峰面积 %
1	9.148	BV	0.1438	170.15536	18.08735	1.5568
2	11.563	BB	0.1973	1.07598e4	818.93884	98.4432

Supplementary Figure 43. HPLC spectra for racemic and chiral **3ak**.

(*E*)–*N*–(2,3–diphenylallyl)aniline (4): colorless oil.^{1}H NMR(500 MHz, $CDCl_3$): δ 7.38–7.25 (m, 3H), 7.25–7.13 (m, 4H), 7.11–7.03 (m, 3H), 6.98–6.90 (m, 2H), 6.71 (t, *J* = 7.3 Hz, 1H), 6.65 (d, *J* = 8.3 Hz, 3H), 4.10 (s, 2H), 3.94 (s, 1H); ^{13}C NMR (125 MHz, $CDCl_3$): δ 147.99, 139.56, 139.36, 136.70, 129.28, 129.24, 128.85, 128.68, 127.97, 127.51, 127.19, 126.71, 117.64, 113.19, 52.28 .

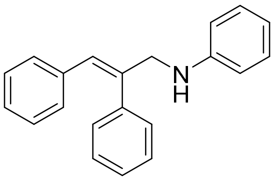

Procedure for the derivatization of 3y and 3n

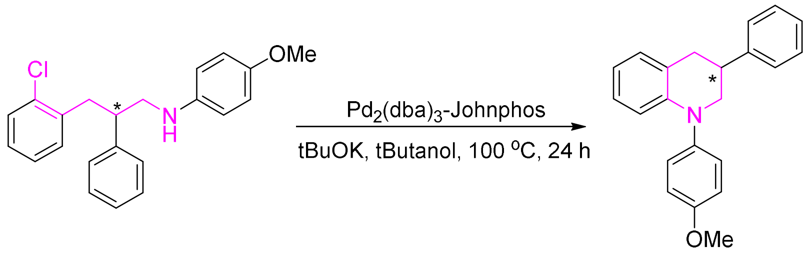

3y 94% ee — **6** 95% yield, 94% ee

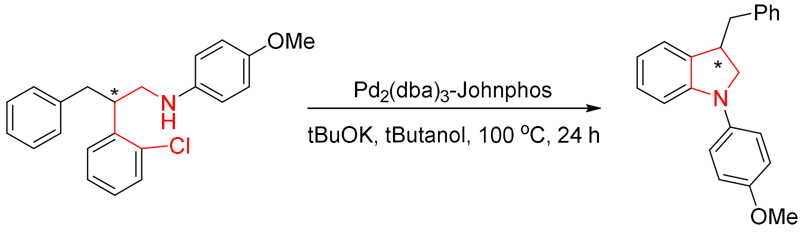

3n 96% ee — **7** 93% yield, 96% ee

Supplementary Figure 44. Derivatization of **3y** and **3n** for the synthesis of cyclic chiral amine **6** and **7**.

General procedure:[12]

An oven–dried vial equipped with a magnetic stir bar and fitted with a Teflon septum was charged with $Pd_2(dba)_3$ (0.05 equiv) and JohnPhos (0.1 equiv). The vessel was evacuated three times and backfilled with argon. Subsequently, the TAA (3 mL) was added via a syringe and the mixture was preheated at 100 °C for 30 min. Another oven–dried vial was charged with tBuOK (1.4 equiv) andsubstrate 3y or 3n. Also, this vessel was evacuated three times and backfilled with argon. The solution of the activated catalyst was transferred from the first vial into the second one via a syringe. The vessel was then heated at 100 °C until the starting component was fully consumed (control by TLC). The mixture was then diluted with EtOAc and filtered through a small plug of Celite® S which was subsequently thoroughly washed with EtOAc. The filtrate was concentrated in vacuo and the residue was purified by column chromatography.

1–(4–methoxyphenyl)–3–phenyl–1,2,3,4–tetrahydroquinoline(6): 95% yield, 94% ee, colorless oil. ^{1}H NMR(500 MHz, $CDCl_3$): δ 7.44–7.36 (m, 2H), 7.36–7.30 (m, 3H), 7.27–7.20 (m, 2H), 7.14 (dd, J = 7.4, 1.4 Hz, 1H), 7.04–6.94 (m, 3H), 6.78–6.72 (m, 1H), 6.63 (dd, J = 8.3, 1.2 Hz, 1H), 3.87 (s, 3H), 3.79–3.65 (m, 2H), 3.43–3.34 (m, 1H), 3.26–3.06 (m, 2H); ^{13}C NMR (125 MHz, $CDCl_3$): δ 156.88, 144.89, 143.44, 140.96, 129.49, 128.65, 127.58, 127.25, 126.75, 122.90, 117.44, 114.89, 114.37, 57.68, 55.53, 38.79, 35.37. HRMS (ESI) m/z calcd for $C_{21}H_{22}NO^+$ $(M+H)^+$ 316.16959, found 316.20612. Enantiomeric excess was determined by chiral HPLC: Chiralpak IA–3 column, Hex/IPA=90:10, 1 mL/min, 220 nm, 7.78 min (minor), 8.75 min (major).

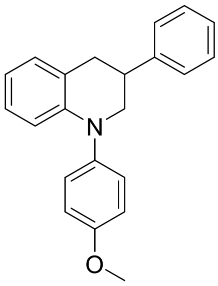

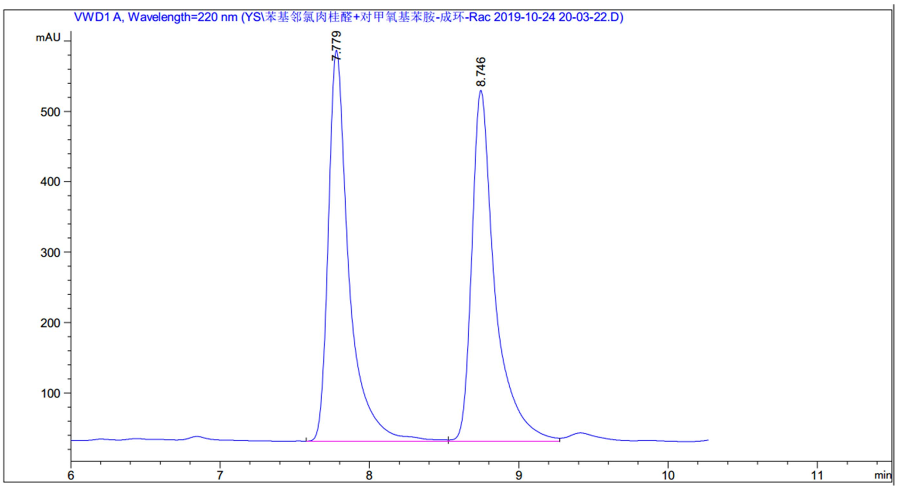

峰 #	保留时间 [min]	类型	峰宽 [min]	峰面积 [mAU*s]	峰高 [mAU]	峰面积 %
1	7.779	BV	0.1427	5358.72363	554.89233	49.8869
2	8.746	VV	0.1580	5383.02539	498.36472	50.1131

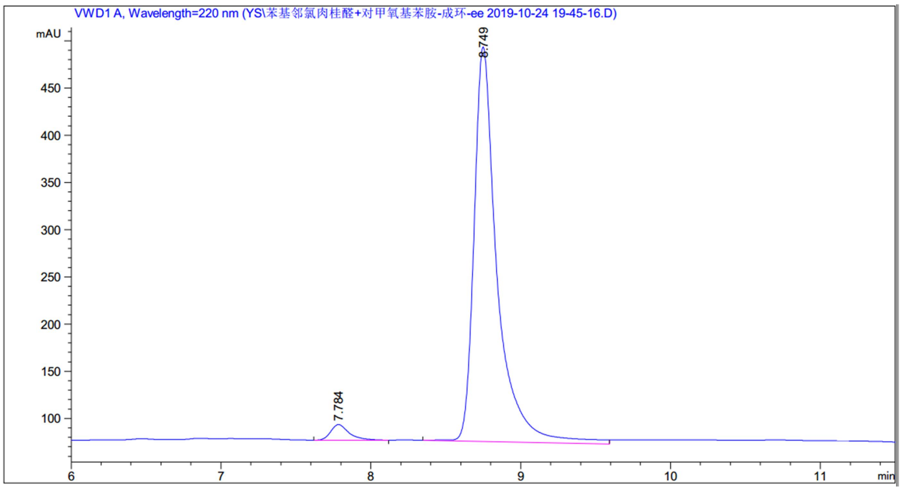

峰 #	保留时间 [min]	类型	峰宽 [min]	峰面积 [mAU*s]	峰高 [mAU]	峰面积 %
1	7.784	BB	0.1298	141.91850	16.59289	3.0660
2	8.749	BV	0.1573	4486.80127	417.60306	96.9340

Supplementary Figure 45. HPLC spectra for racemic and chiral **6**

3–benzyl–1–(4–methoxyphenyl)indoline(7): 93% yield, 96% ee, colorless oil.^{1}H NMR(500 MHz, $CDCl_3$): δ 7.30 (t, *J* = 7.6 Hz, 2H), 7.25–7.20 (m, 3H), 7.14 (d, *J* = 9.0 Hz, 2H), 7.09–7.00 (m, 2H), 6.94–6.85 (m, 3H), 6.69 (t, *J* = 7.3 Hz, 1H), 3.90–3.83 (m, 1H), 3.79 (s, 3H), 3.69–3.53 (m, 2H), 3.15 (dd, *J* = 13.8, 5.7 Hz, 1H), 2.85 (dd, *J* = 13.8, 9.4 Hz, 1H); ^{13}C NMR (125 MHz, $CDCl_3$): δ 154.81, 148.23, 139.82, 137.75, 133.79, 129.08, 128.50, 127.65, 126.32, 124.36, 120.64, 118.16, 114.53, 107.55, 58.62, 55.61, 41.90, 40.79. HRMS (ESI) m/z calcd for $C_{21}H_{22}NO^+$ $(M+H)^+$ 316.16959, found 316.20627. Enantiomeric excess was determined by chiral HPLC: Chiralpak IA–3 column, Hex/IPA=90:10, 1 mL/min, 220 nm, 8.30 min (major), 9.05 min (minor).

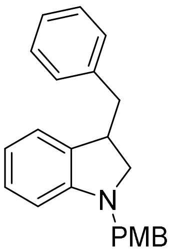

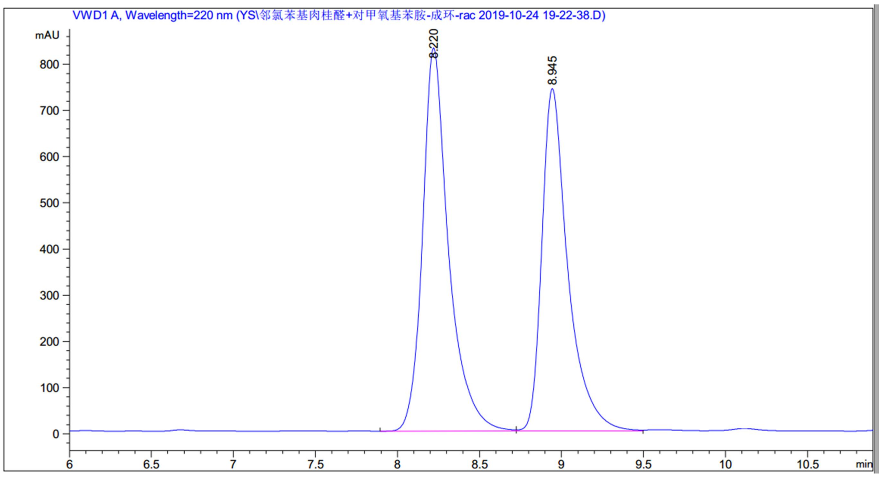

峰 #	保留时间 [min]	类型	峰宽 [min]	峰面积 [mAU*s]	峰高 [mAU]	峰面积 %
1	8.220	BV	0.1635	9204.32031	829.07086	52.8927
2	8.945	VV	0.1651	8197.54297	740.75647	47.1073

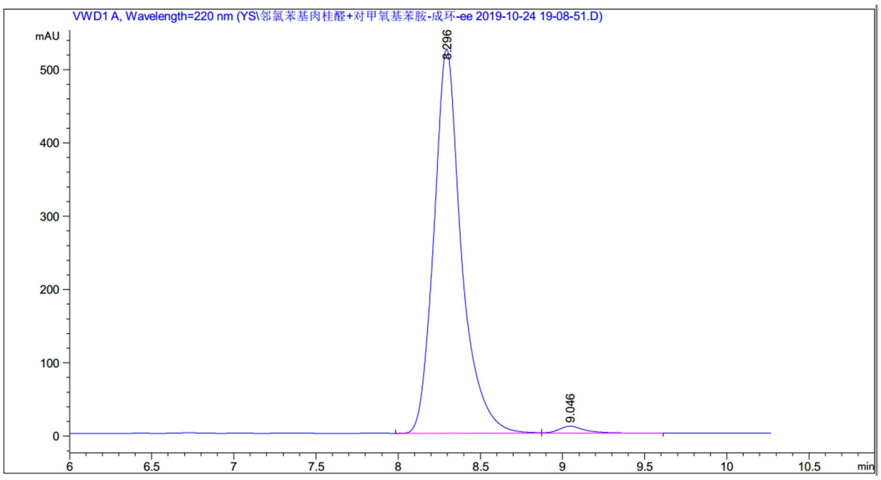

峰 #	保留时间 [min]	类型	峰宽 [min]	峰面积 [mAU*s]	峰高 [mAU]	峰面积 %
1	8.296	BV	0.1700	6110.89209	524.25092	98.2407
2	9.046	VB	0.1662	109.43569	9.65883	1.7593

Supplementary Figure 46. HPLC spectra for racemic and chiral **7**.

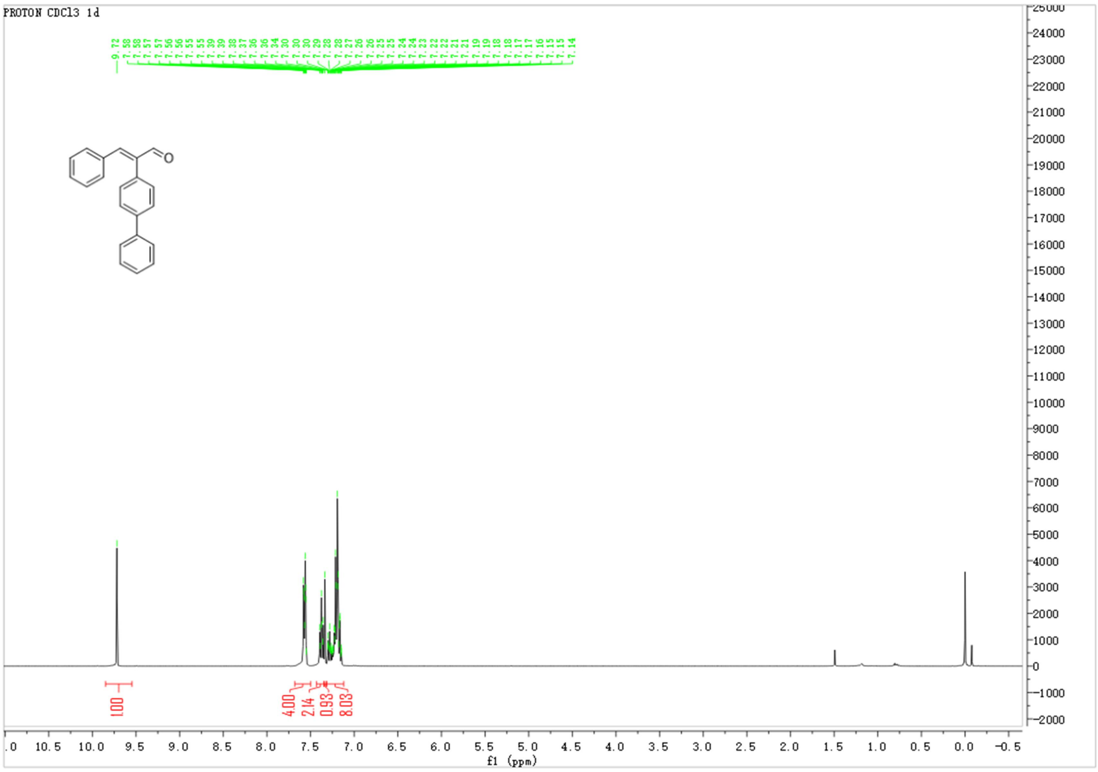

Supplementary Figure 47. ^{1}H NMR spectrum of **1d** in $CDCl_3$.

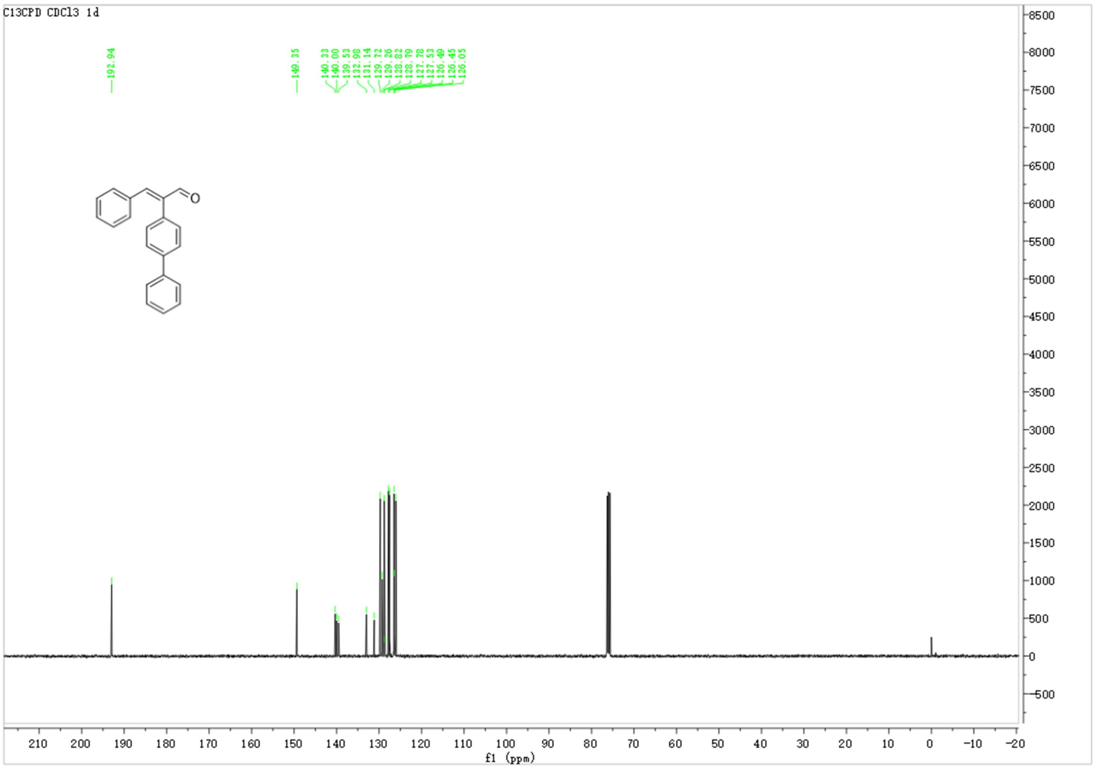

Supplementary Figure 48. ^{13}C NMR spectrum of **1d** in $CDCl_3$.

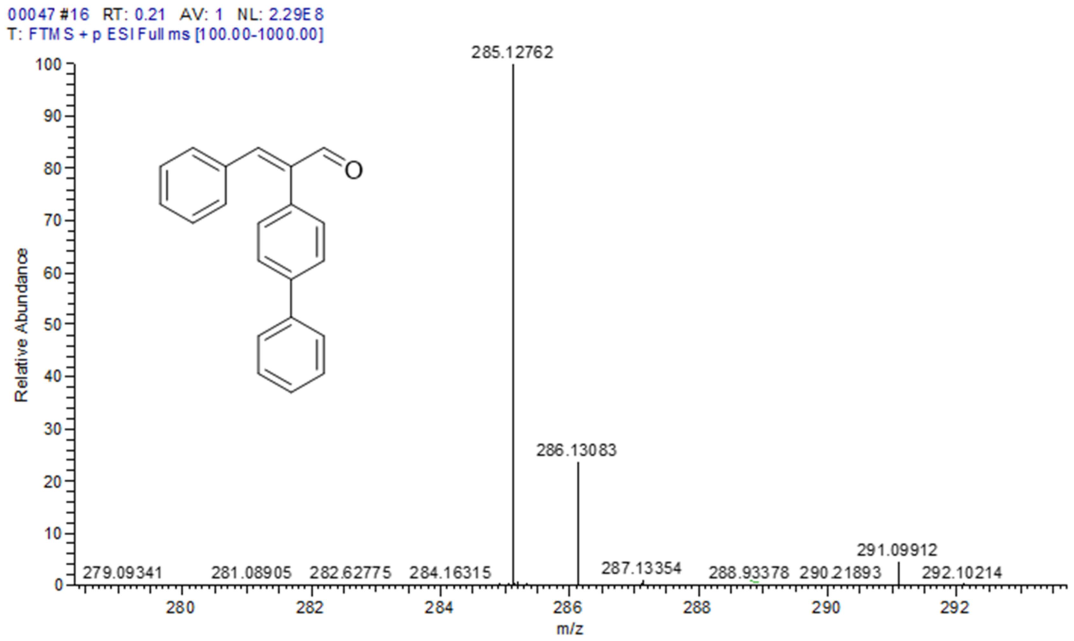

Supplementary Figure 49. HRMS of **1d**.

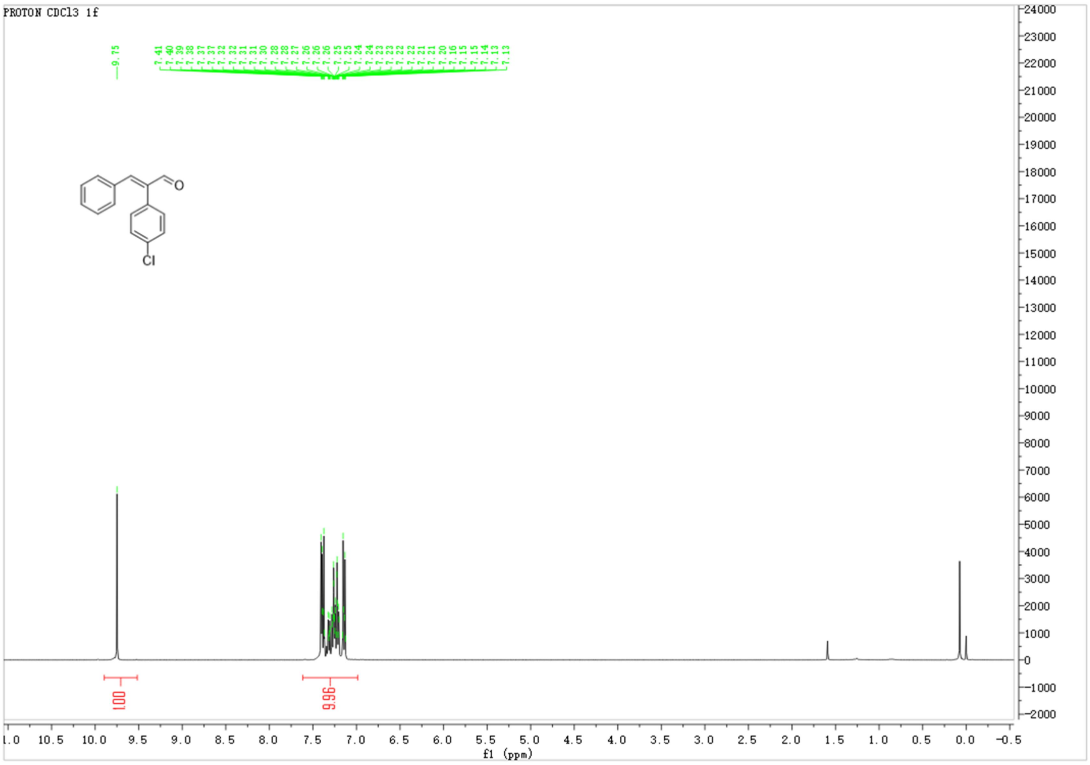

Supplementary Figure 50. ^{1}H NMR spectrum of **1f** in $CDCl_3$.

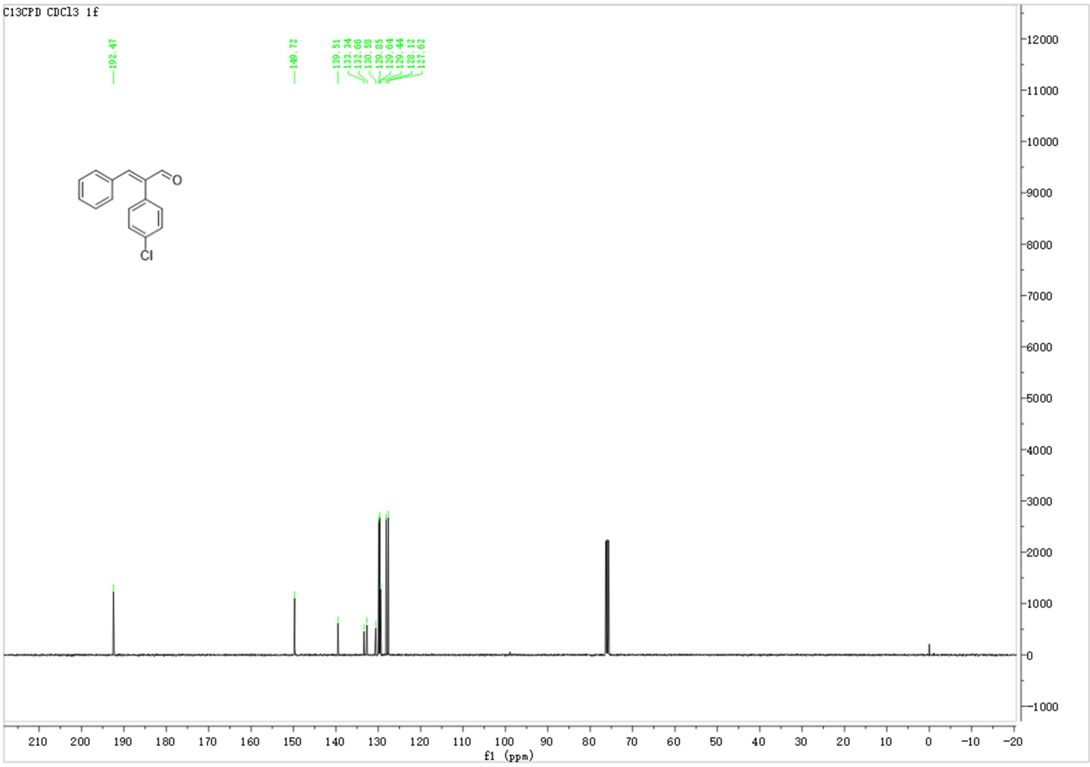

Supplementary Figure 51. ^{13}C NMR spectrum of **1f** in $CDCl_3$.

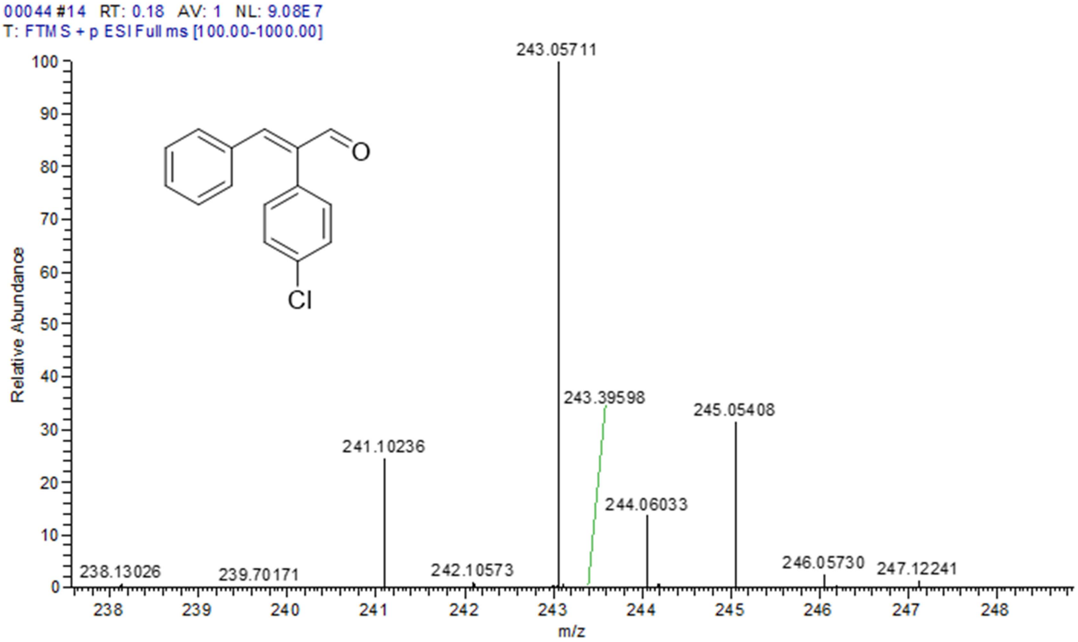

Supplementary Figure 52. HRMS of **1f**.

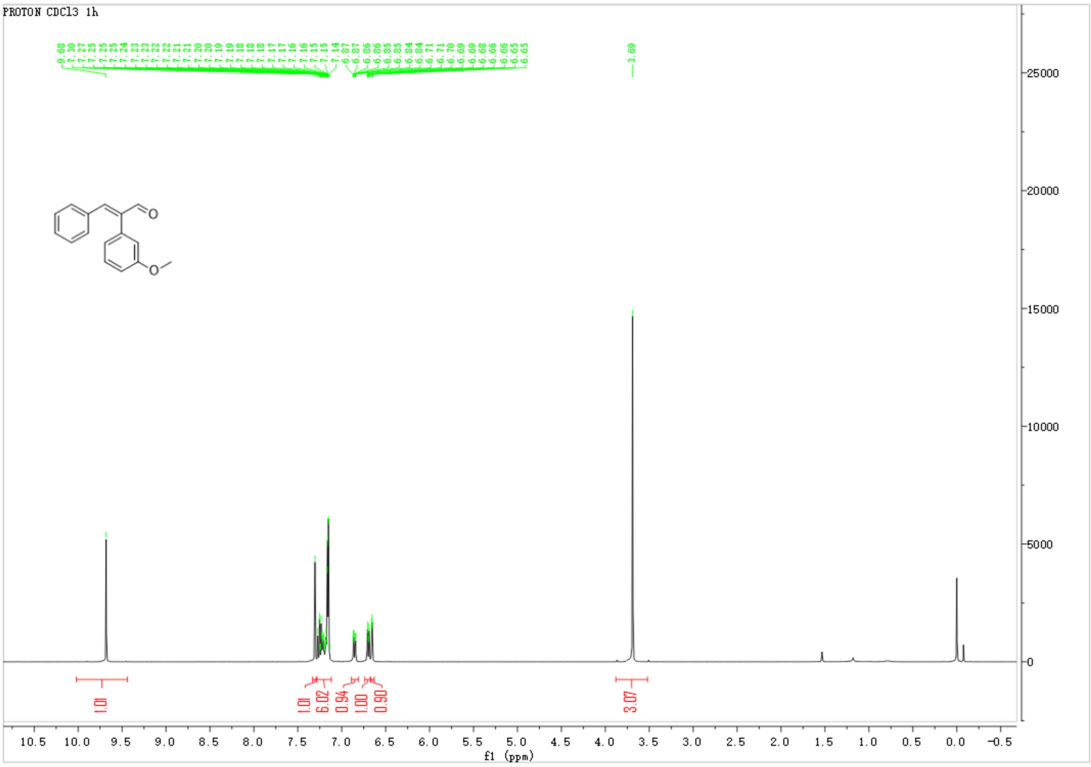

Supplementary Figure 53. ^{1}H NMR spectrum of **1h** in $CDCl_3$.

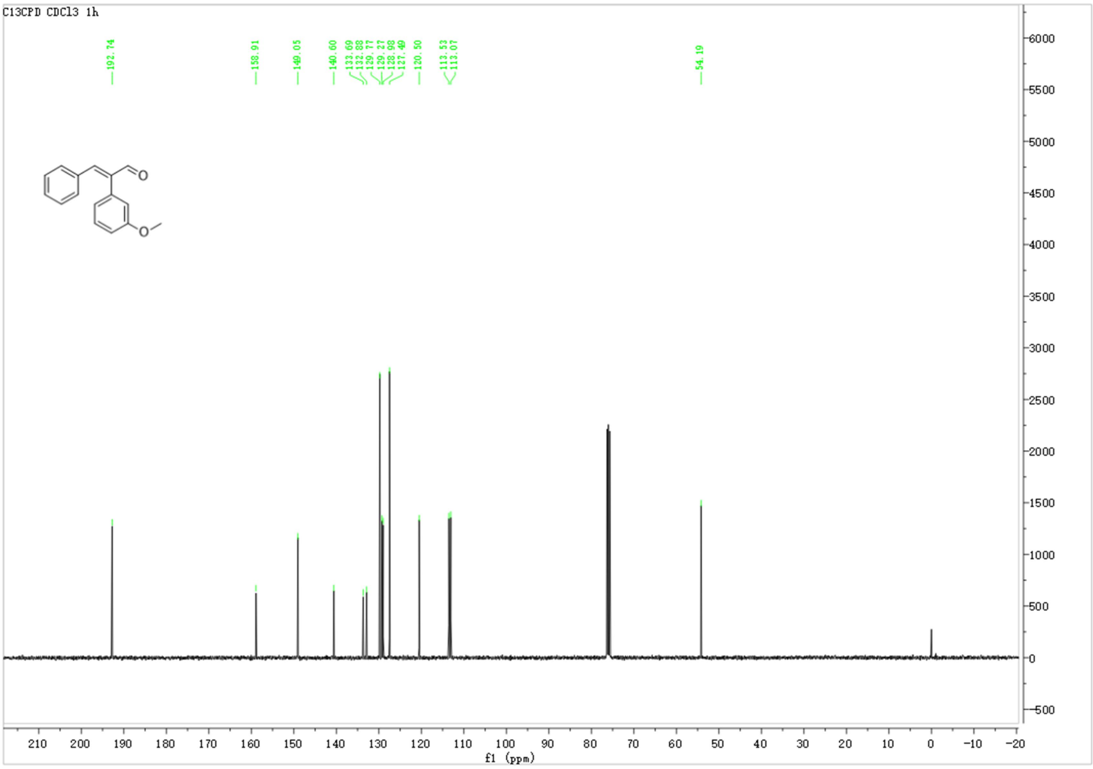

Supplementary Figure 54. ^{13}C NMR spectrum of **1h** in $CDCl_3$.

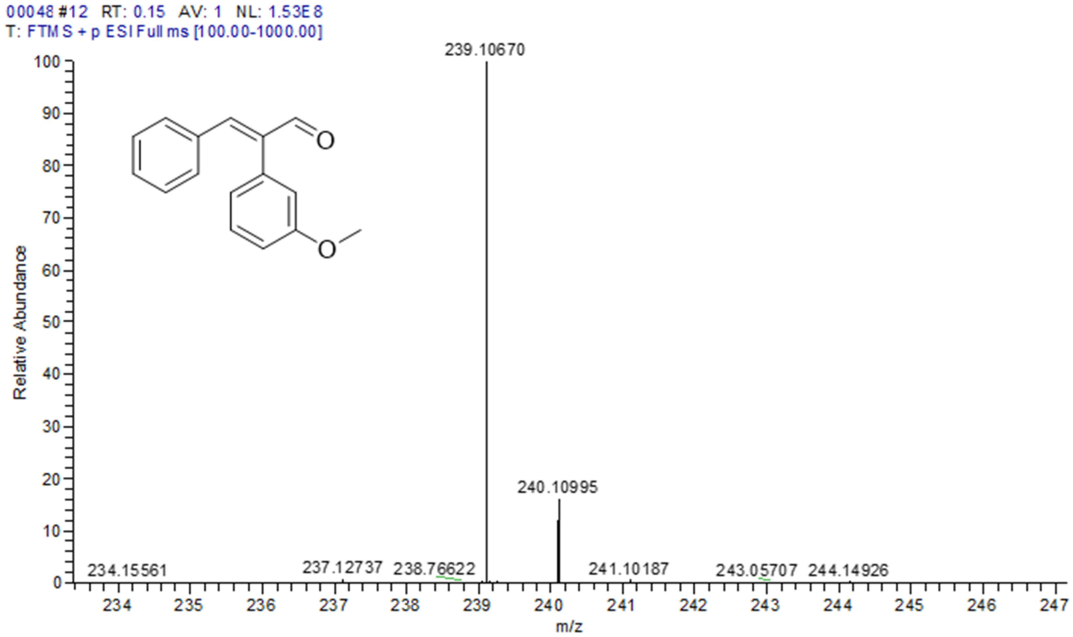

Supplementary Figure 55. HRMS of **1h**.

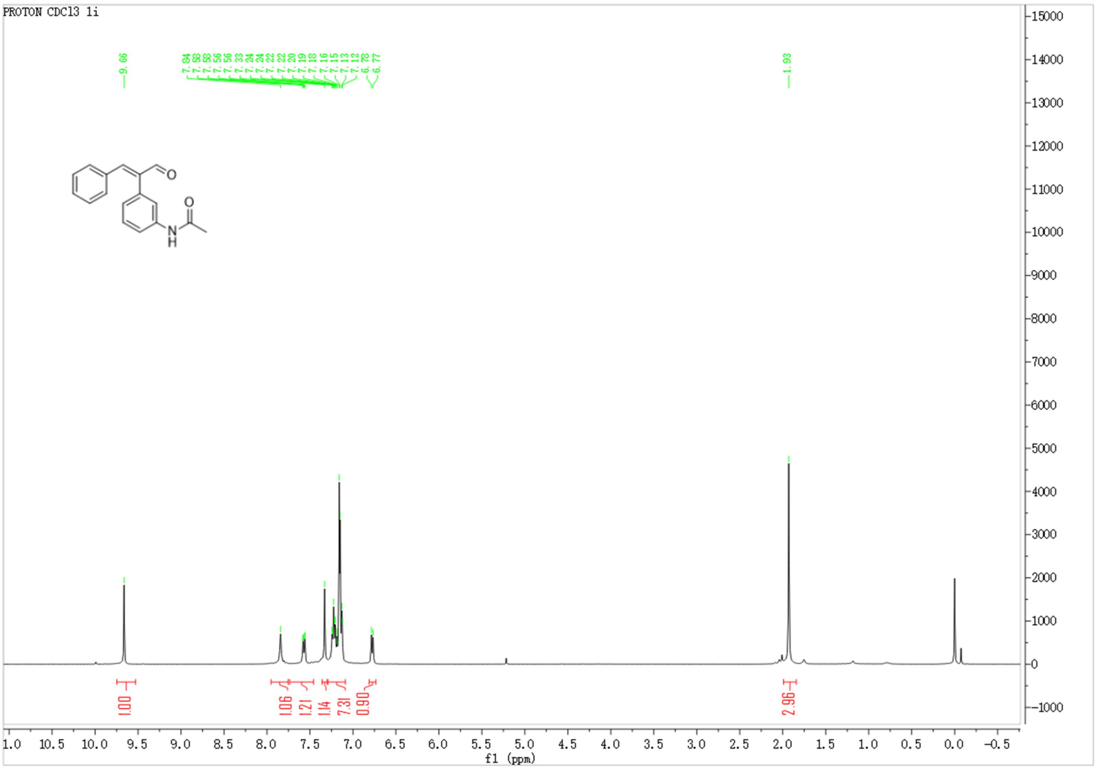

Supplementary Figure 56. ^{1}H NMR spectrum of **1i** in $CDCl_3$.

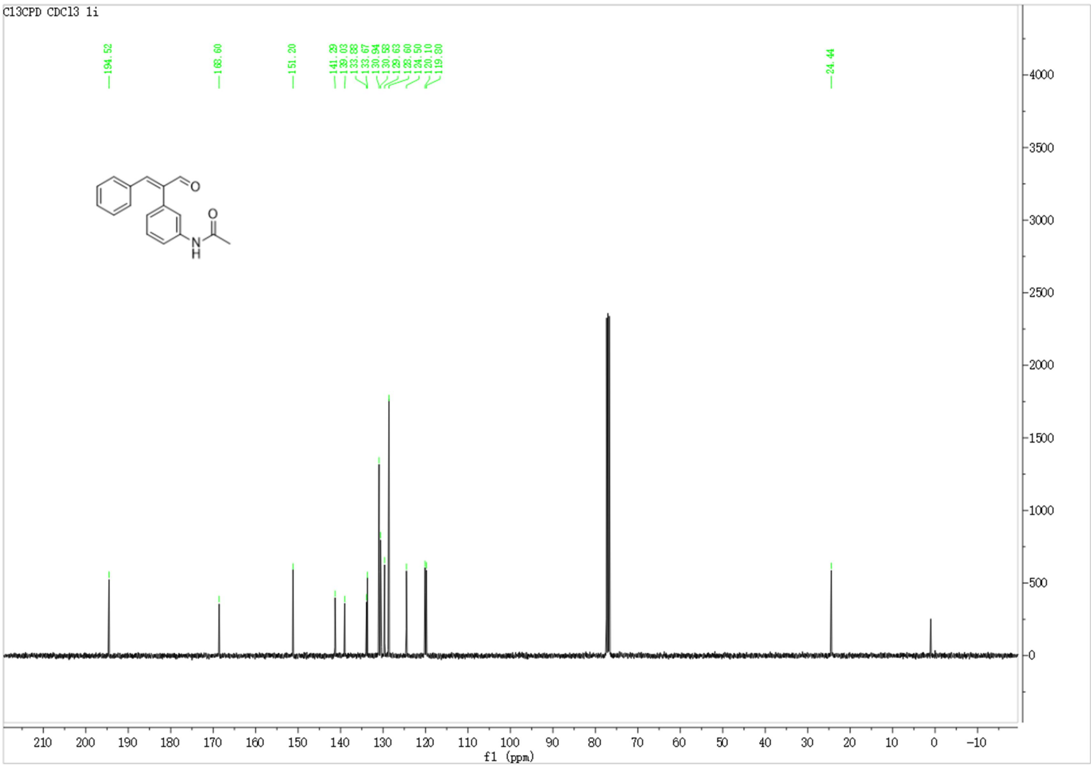

Supplementary Figure 57. ^{13}C NMR spectrum of **1i** in $CDCl_3$.

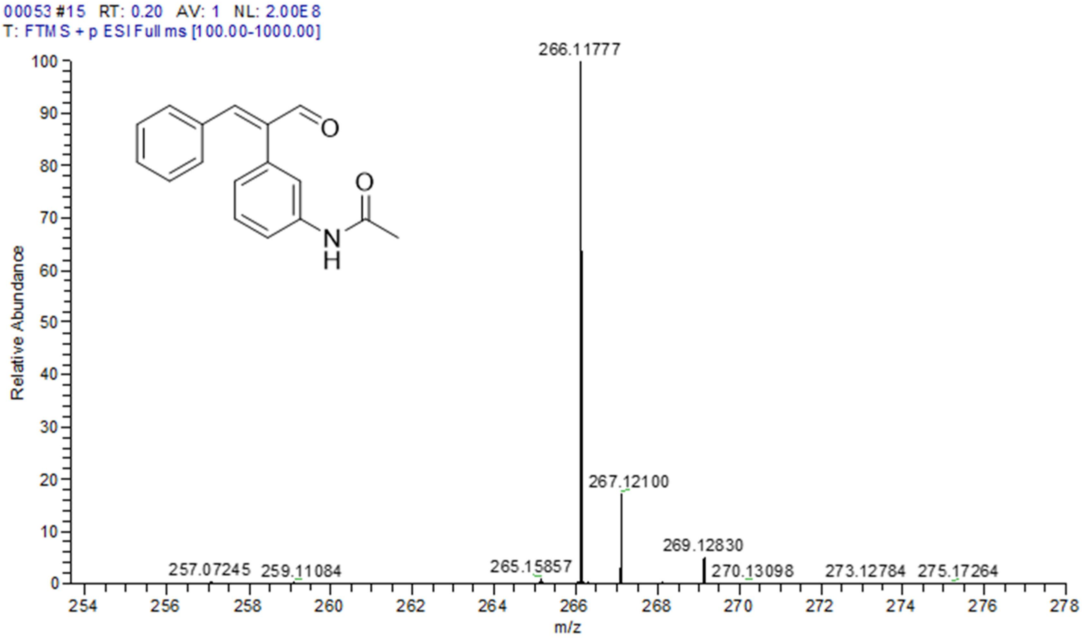

Supplementary Figure 58. HRMS of **1i**.

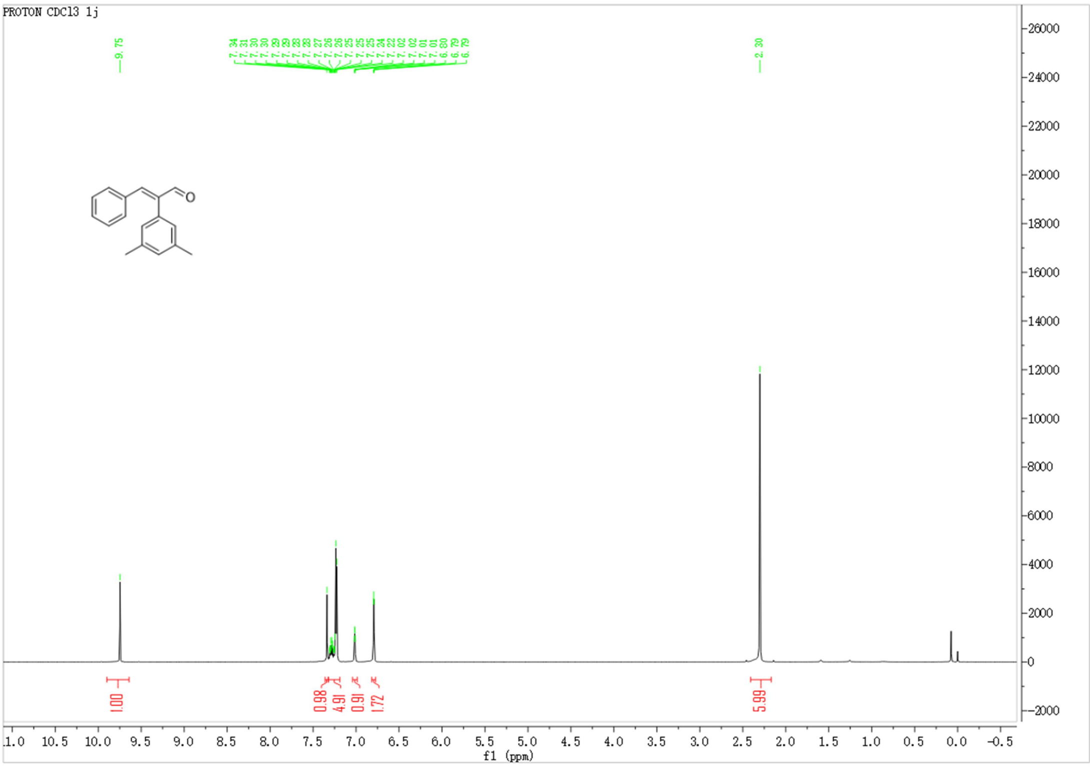

Supplementary Figure 59. ^{1}H NMR spectrum of **1j** in $CDCl_3$.

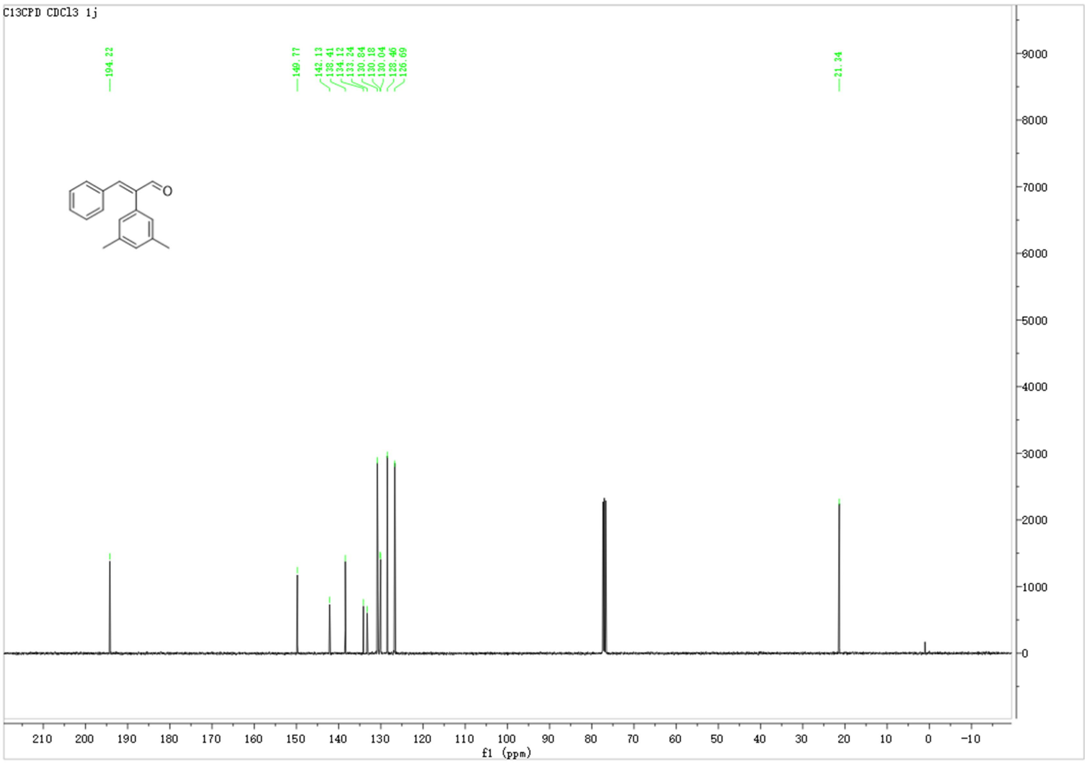

Supplementary Figure 60. ^{13}C NMR spectrum of **1j** in $CDCl_3$.

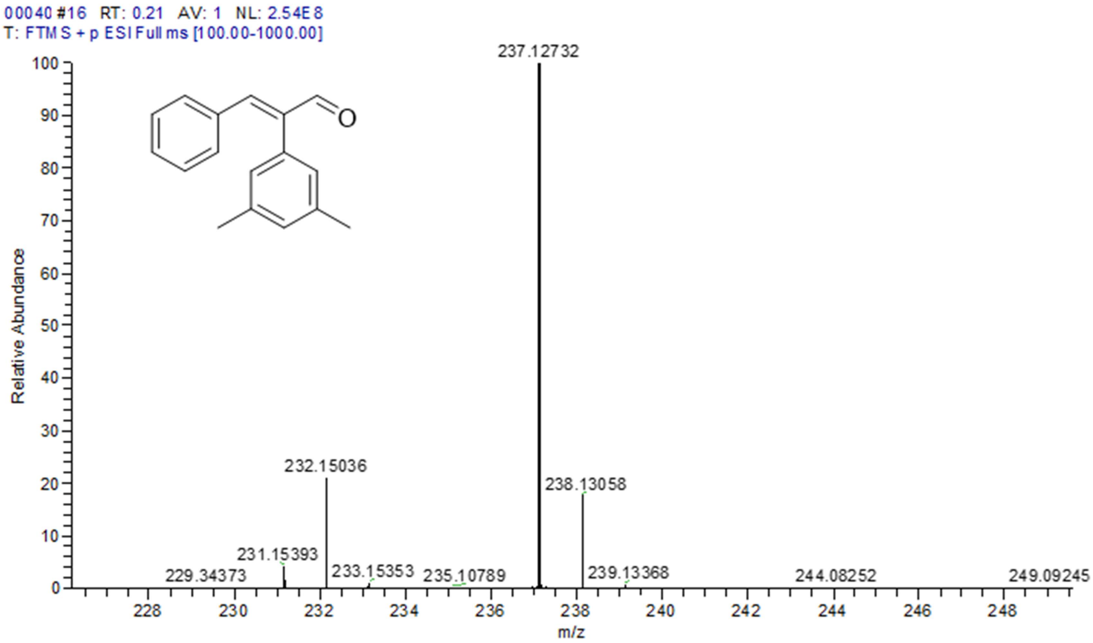

Supplementary Figure 61. HRMS of **1j**.

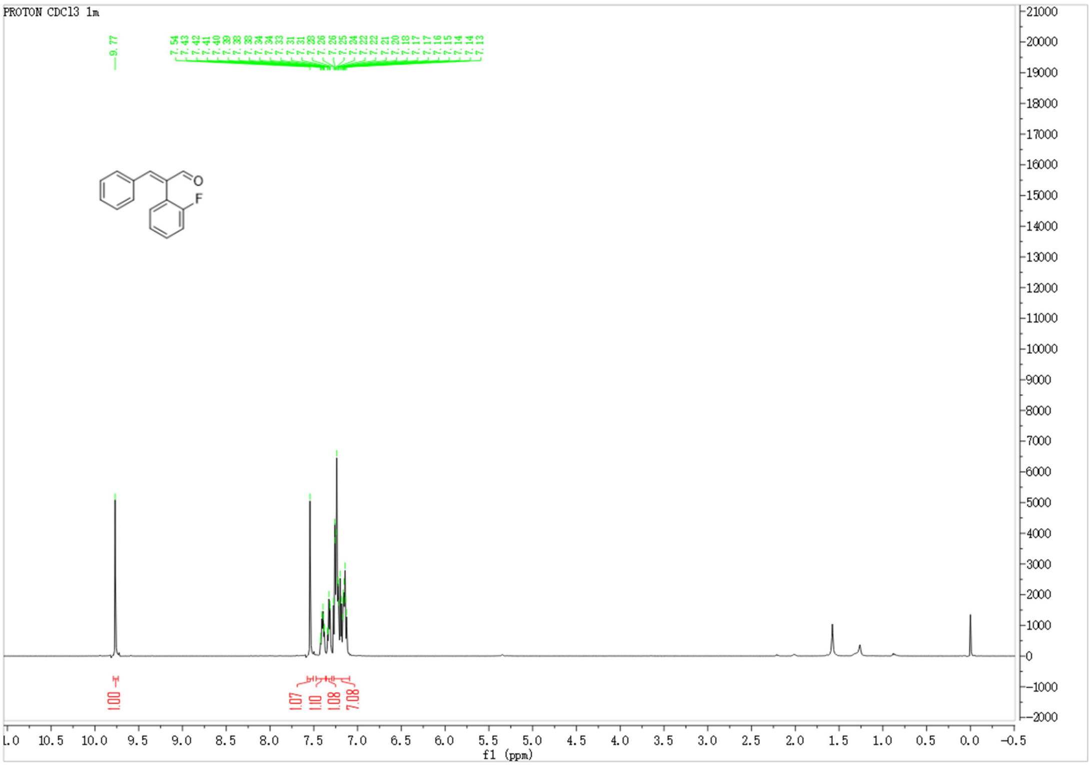

Supplementary Figure 62. ^{1}H NMR spectrum of **1m** in $CDCl_3$.

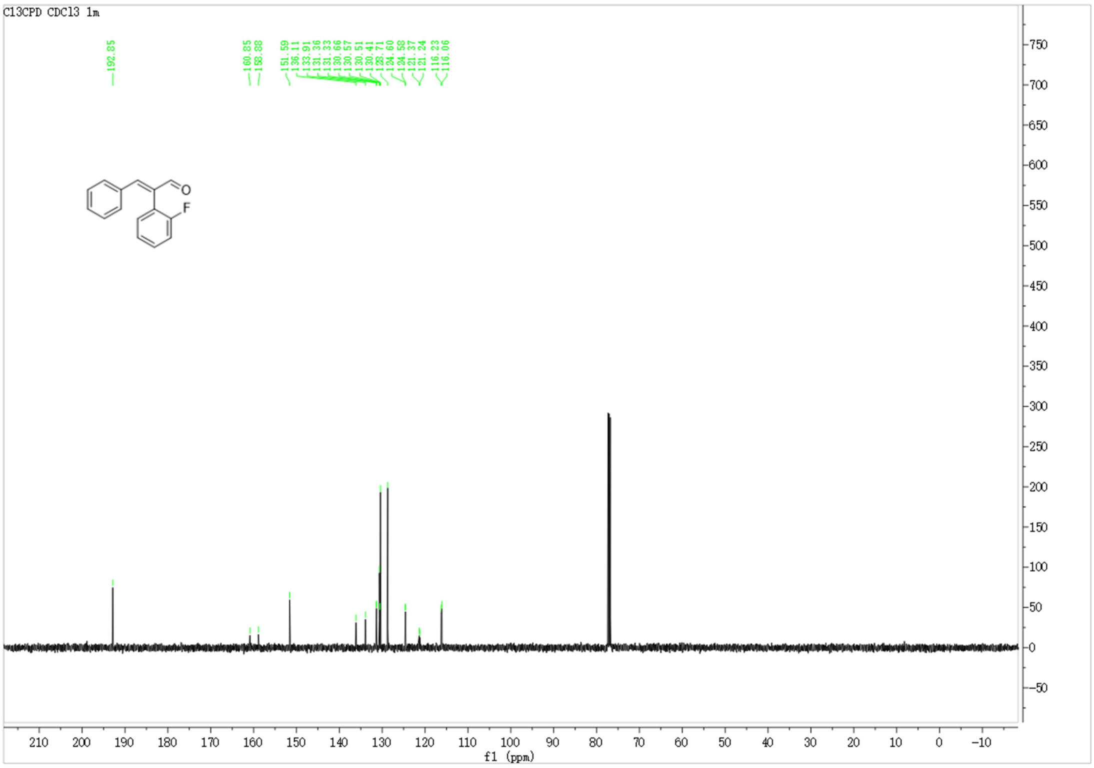

Supplementary Figure 63. ^{13}C NMR spectrum of **1m** in $CDCl_3$.

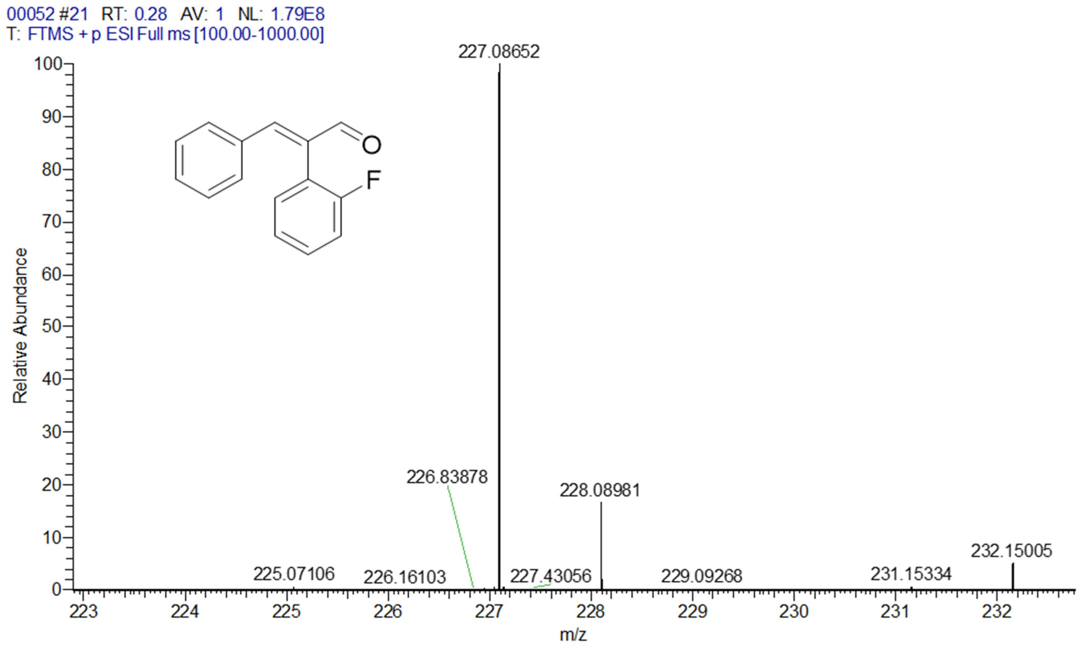

Supplementary Figure 64. HRMS of **1m**.

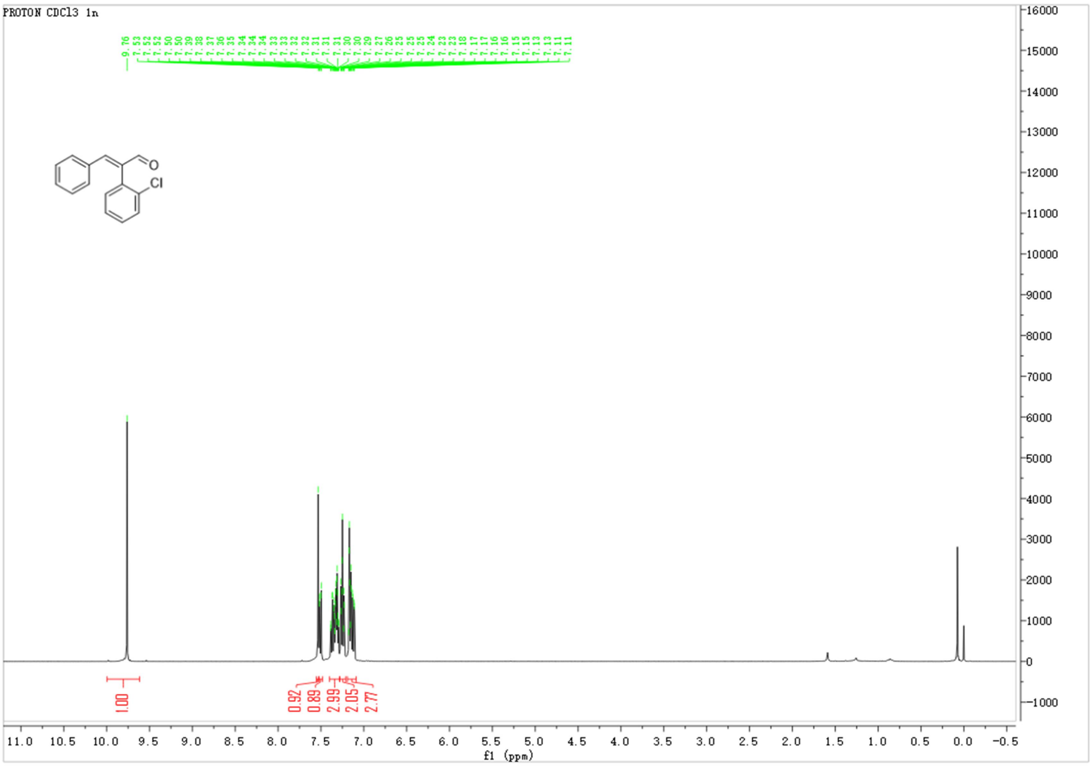

Supplementary Figure 65. ^{1}H NMR spectrum of **1n** in $CDCl_3$.

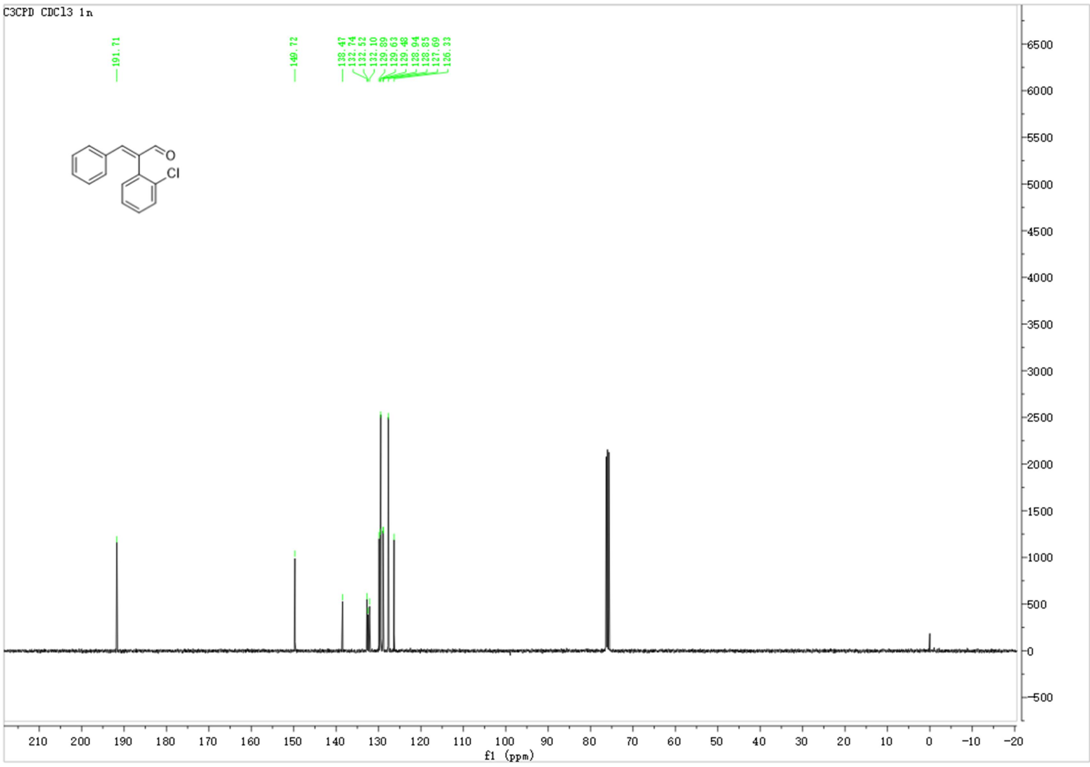

Supplementary Figure 66. ^{13}C NMR spectrum of **1n** in $CDCl_3$.

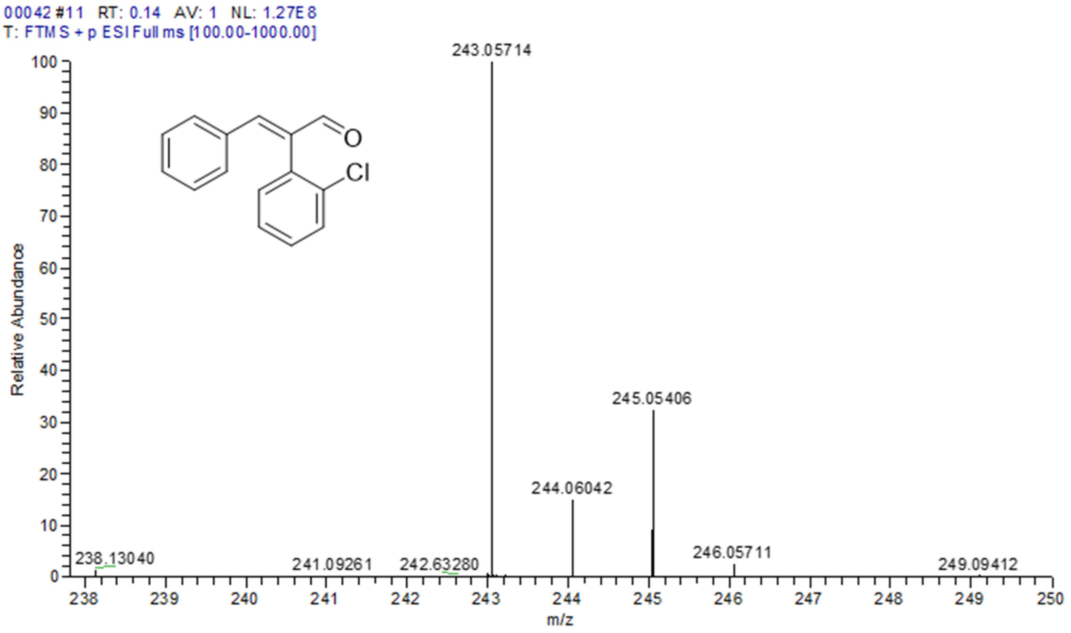

Supplementary Figure 67. HRMS of **1n**.

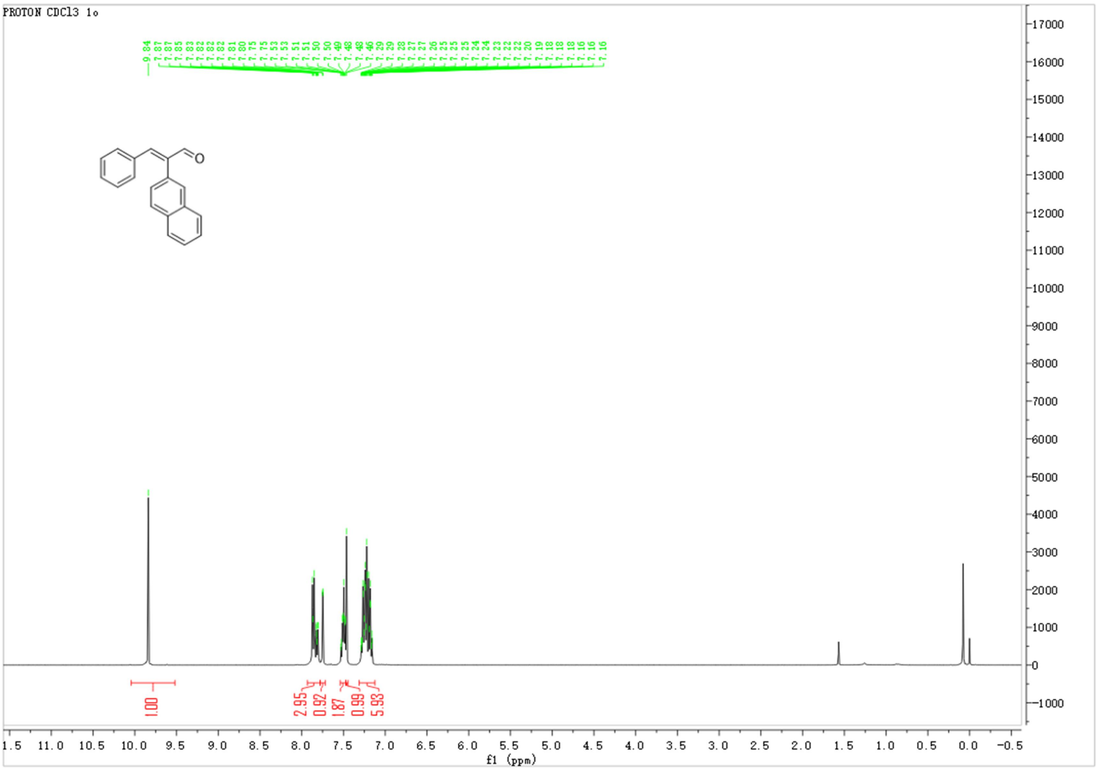

Supplementary Figure 68. ^{1}H NMR spectrum of **1o** in $CDCl_3$.

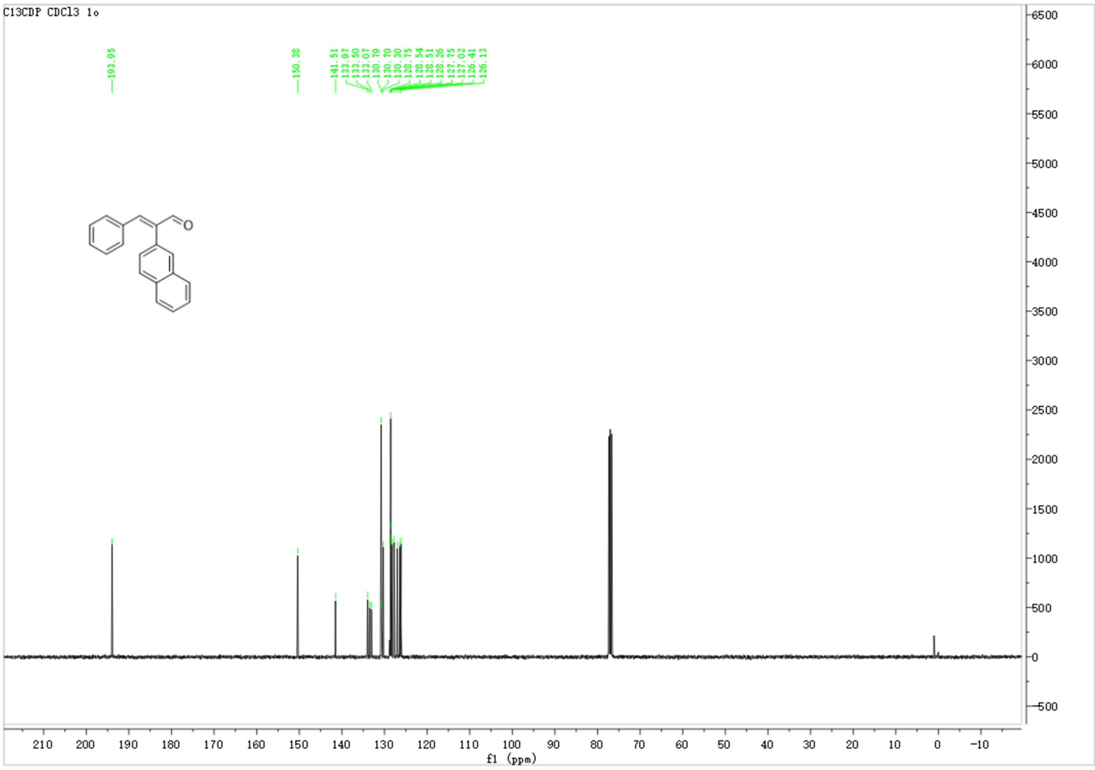

Supplementary Figure 69. ^{13}C NMR spectrum of **1o** in $CDCl_3$.

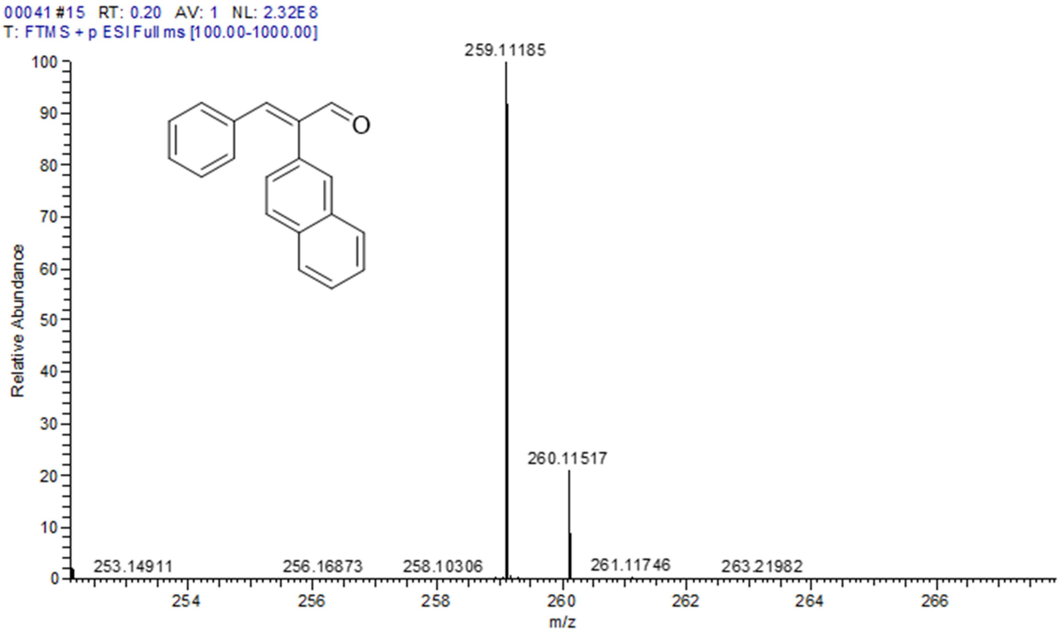

Supplementary Figure 70. HRMS of **1o**.

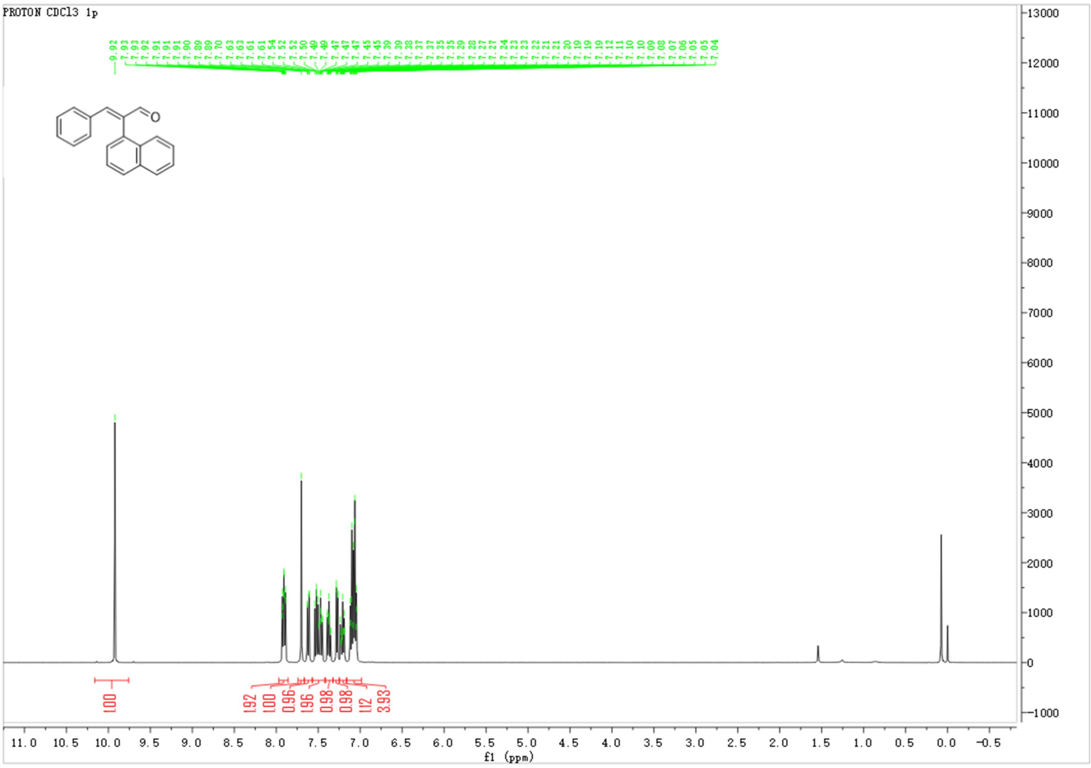

Supplementary Figure 71. ^{1}H NMR spectrum of **1p** in $CDCl_3$.

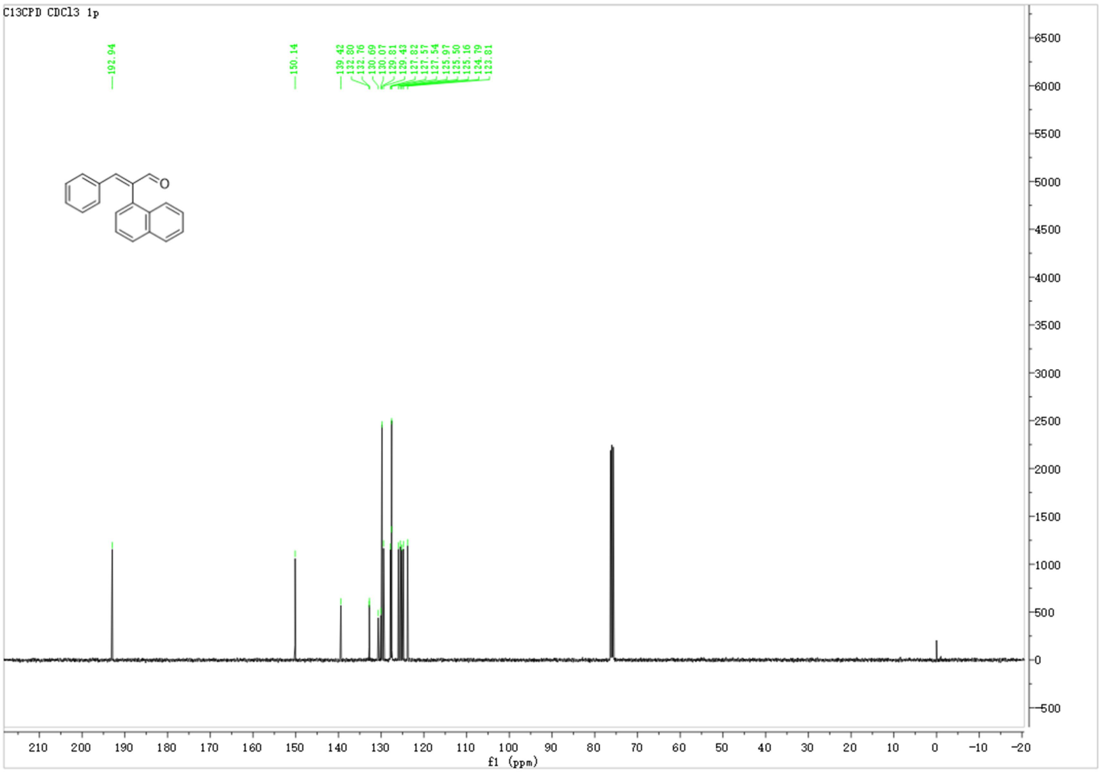

Supplementary Figure 72. ^{13}C NMR spectrum of **1p** in $CDCl_3$.

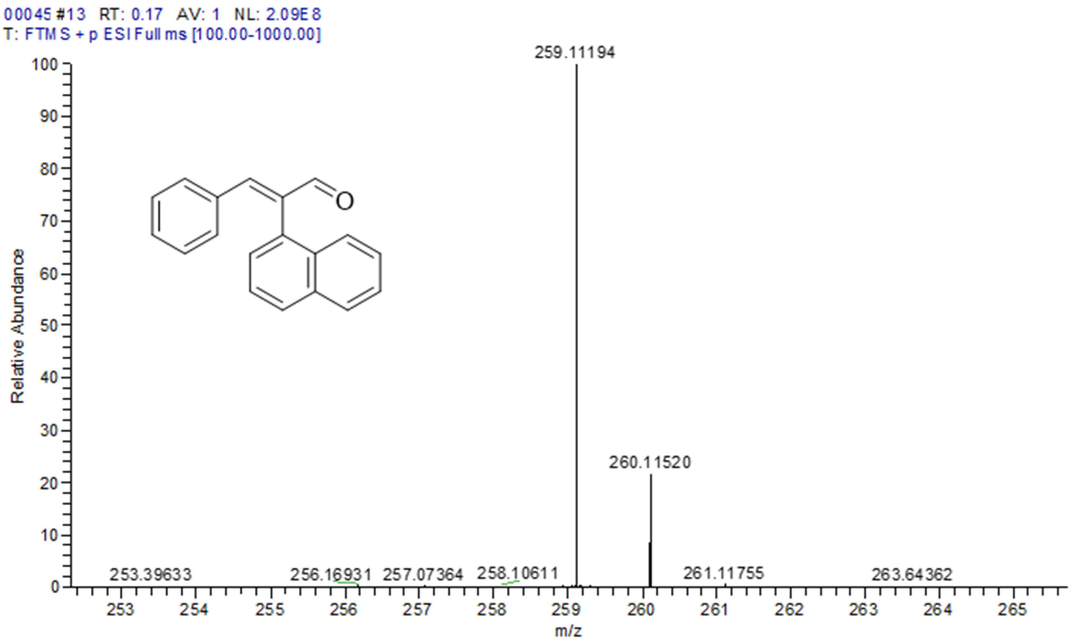

Supplementary Figure 73. HRMS of **1p**.

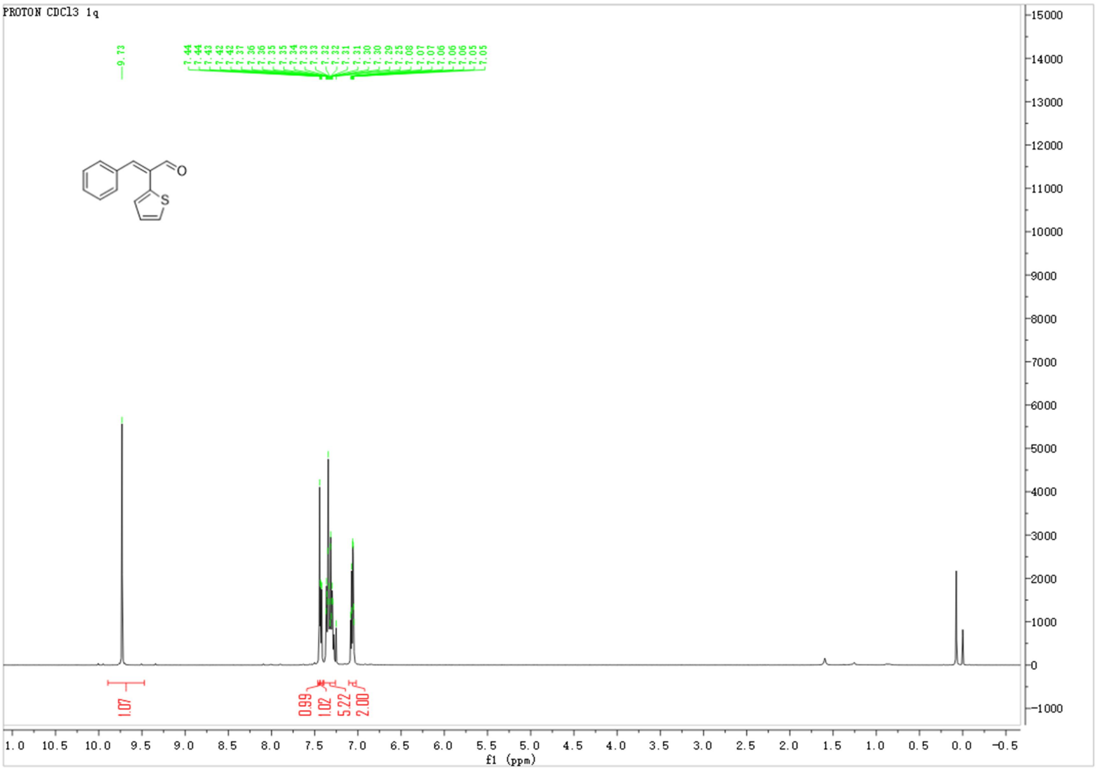

Supplementary Figure 74. ^{1}H NMR spectrum of **1q** in $CDCl_3$.

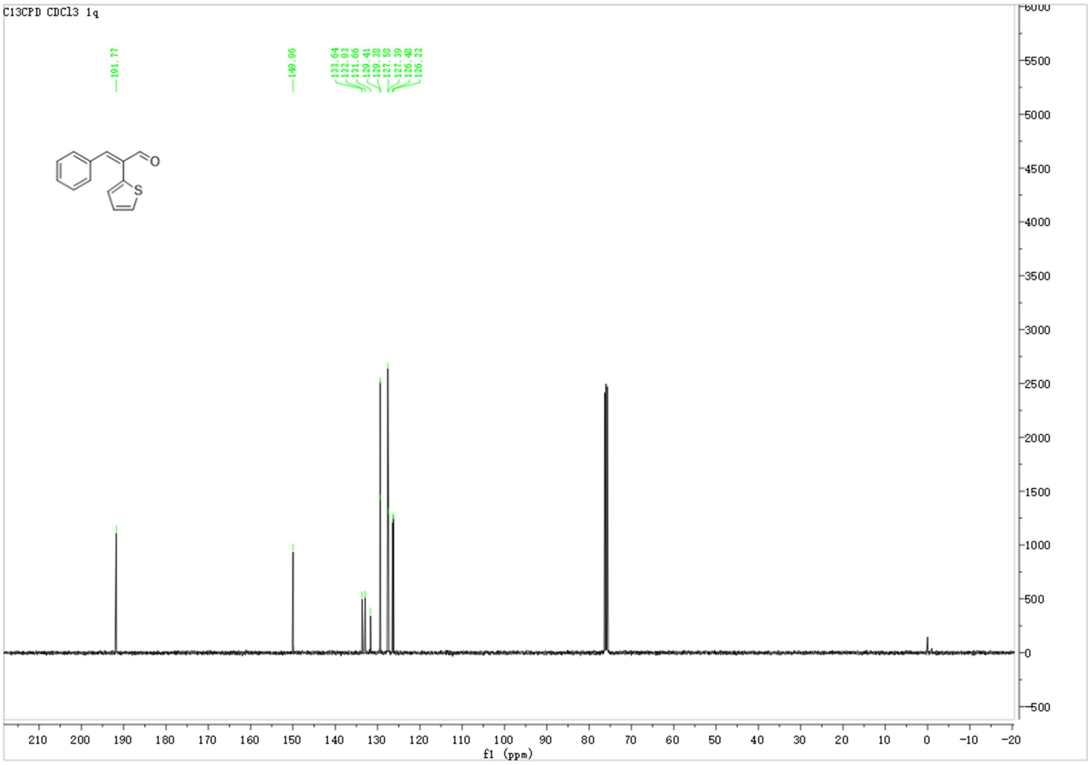

Supplementary Figure 75. ^{13}C NMR spectrum of **1q** in $CDCl_3$.

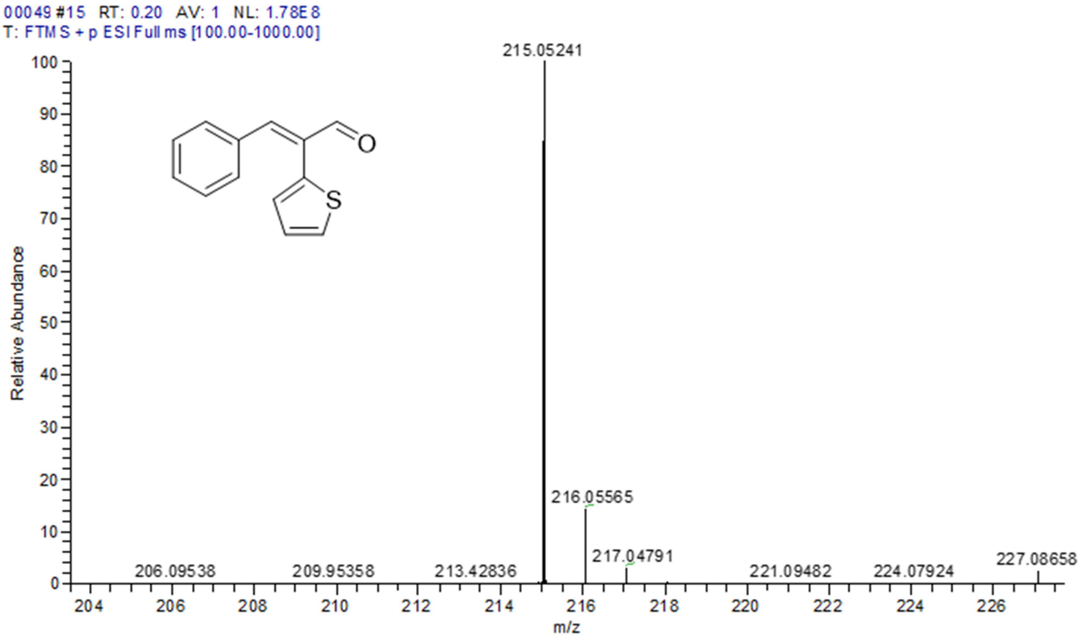

Supplementary Figure 76. HRMS of **1q**.

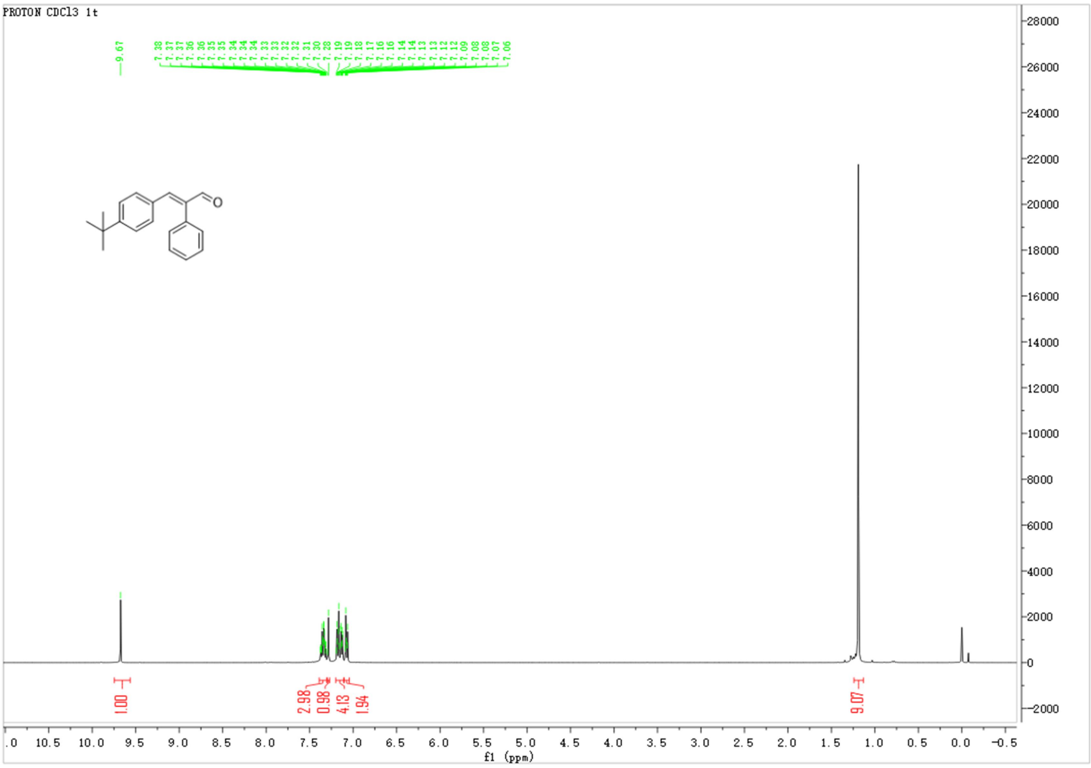

Supplementary Figure 77. ^{1}H NMR spectrum of **1t** in $CDCl_3$.

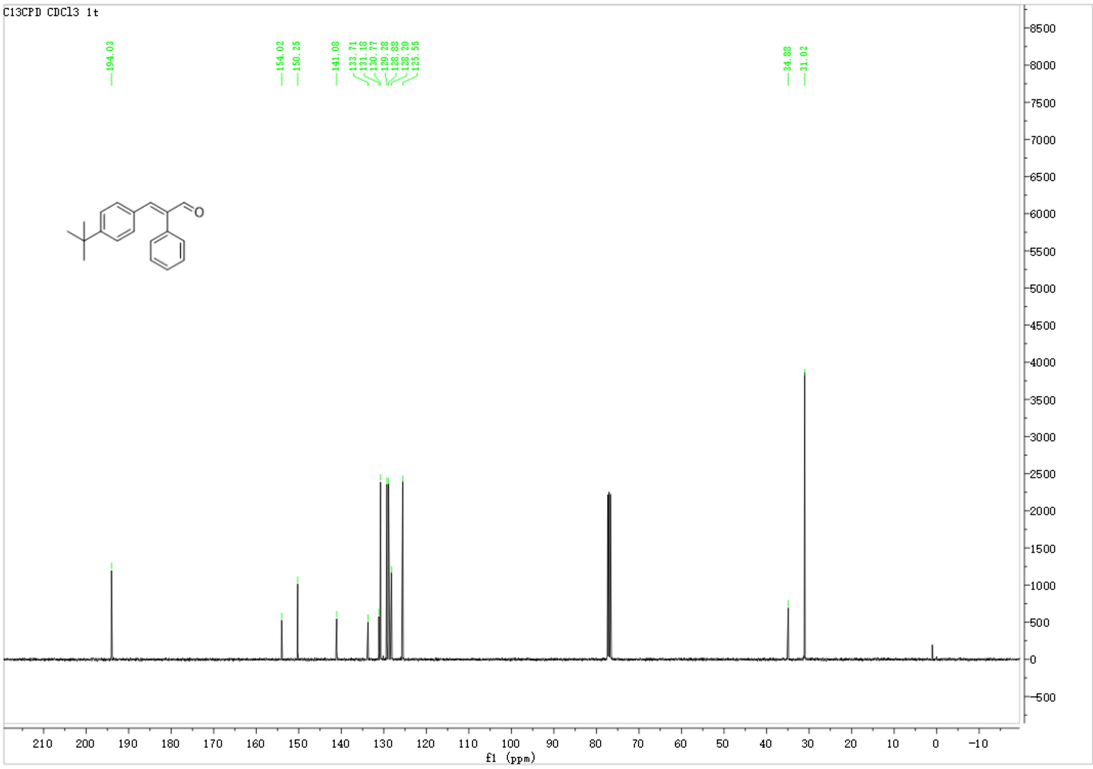

Supplementary Figure 78. ^{13}C NMR spectrum of **1t** in $CDCl_3$.

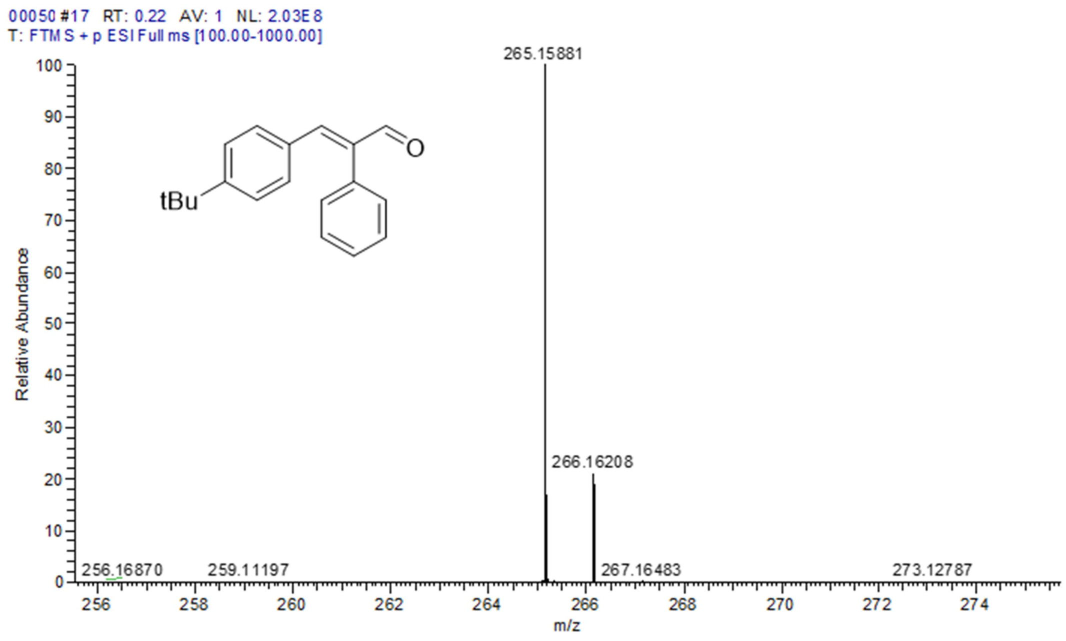

Supplementary Figure 79. HRMS of **1t**.

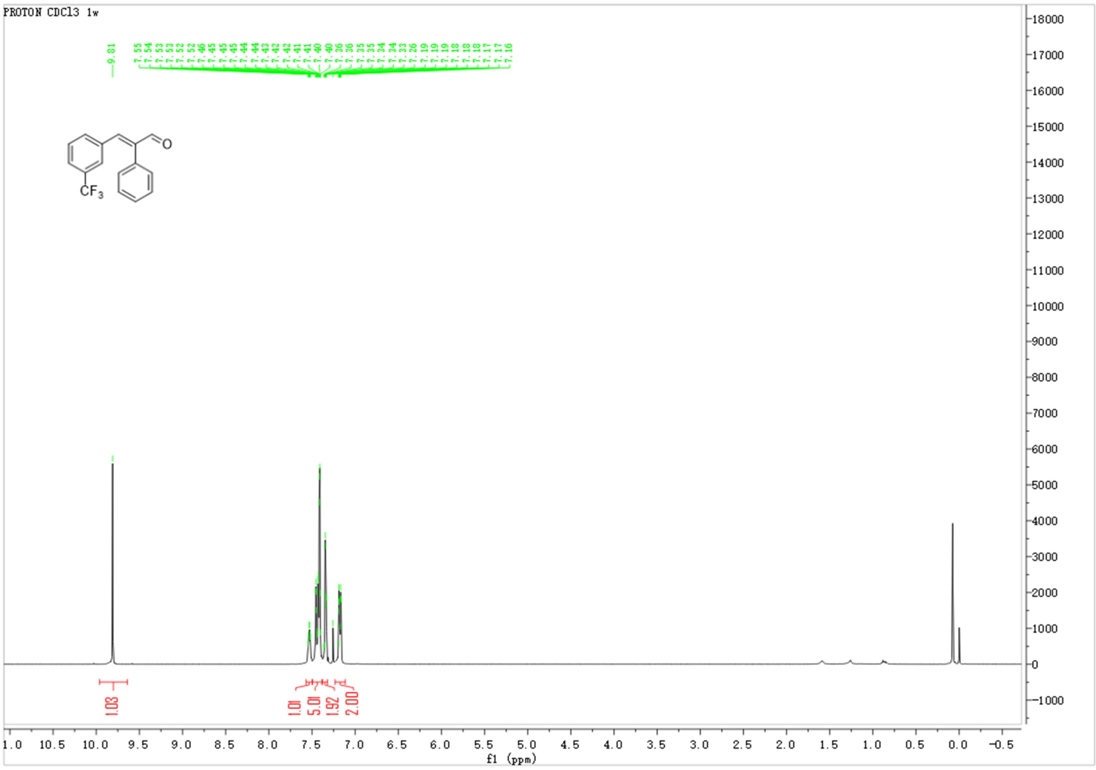

Supplementary Figure 80. ^{1}H NMR spectrum of **1w** in $CDCl_3$.

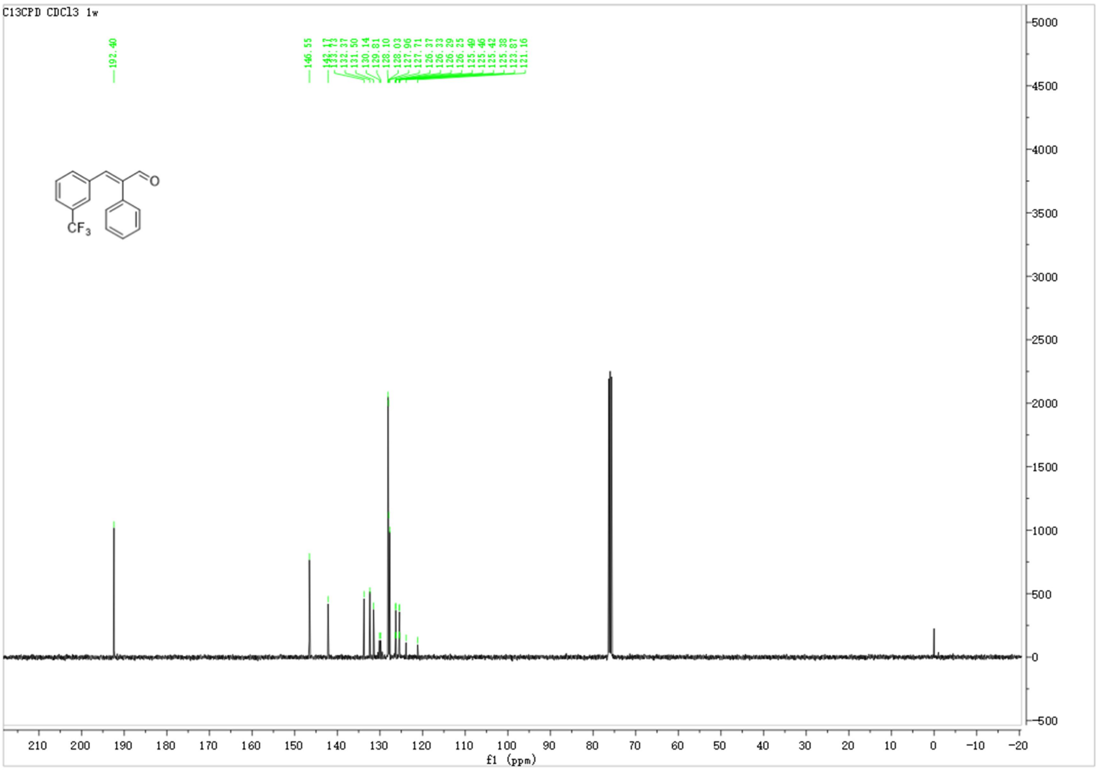

Supplementary Figure 81. ^{13}C NMR spectrum of **1w** in $CDCl_3$.

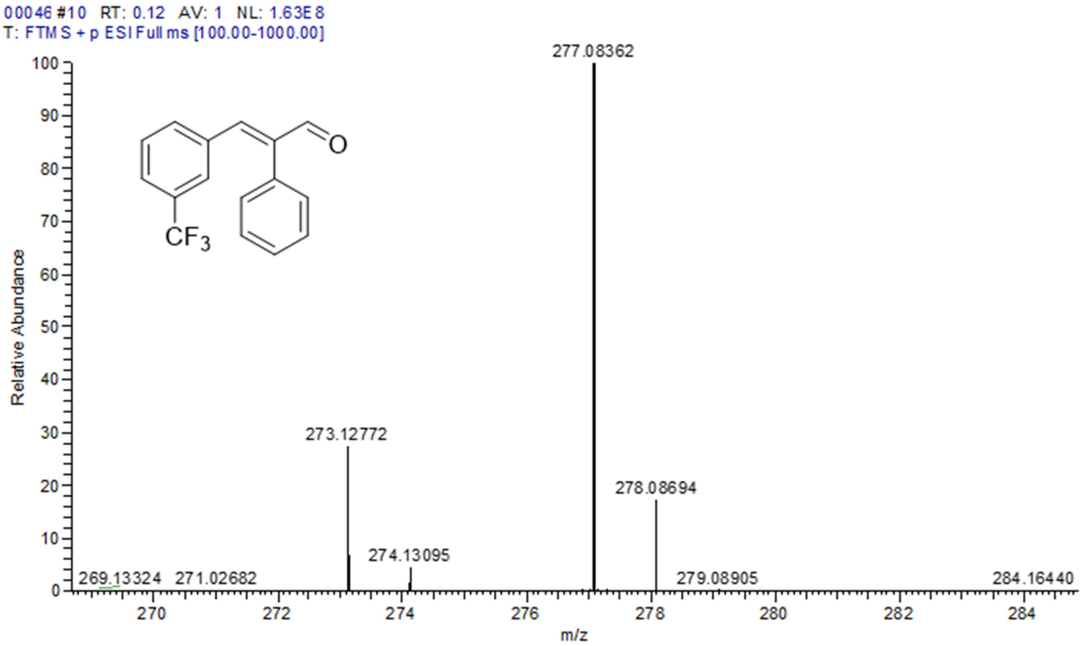

Supplementary Figure 82. HRMS of **1w**.

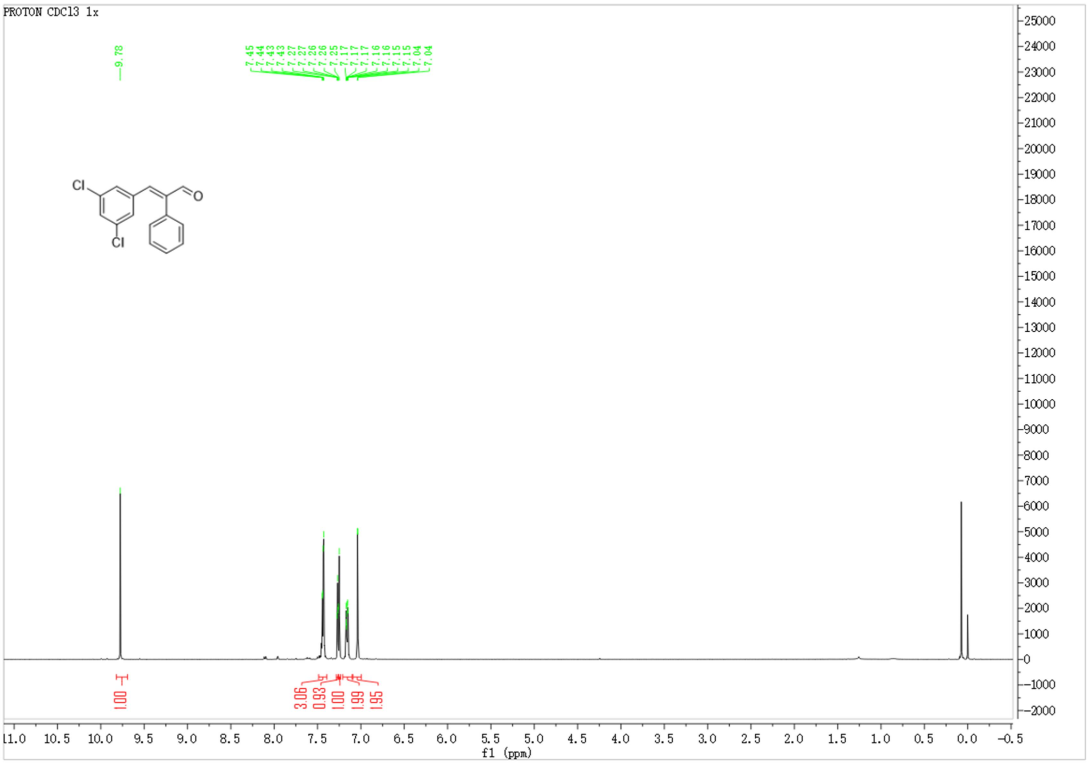

Supplementary Figure 83. ^{1}H NMR spectrum of **1x** in $CDCl_3$.

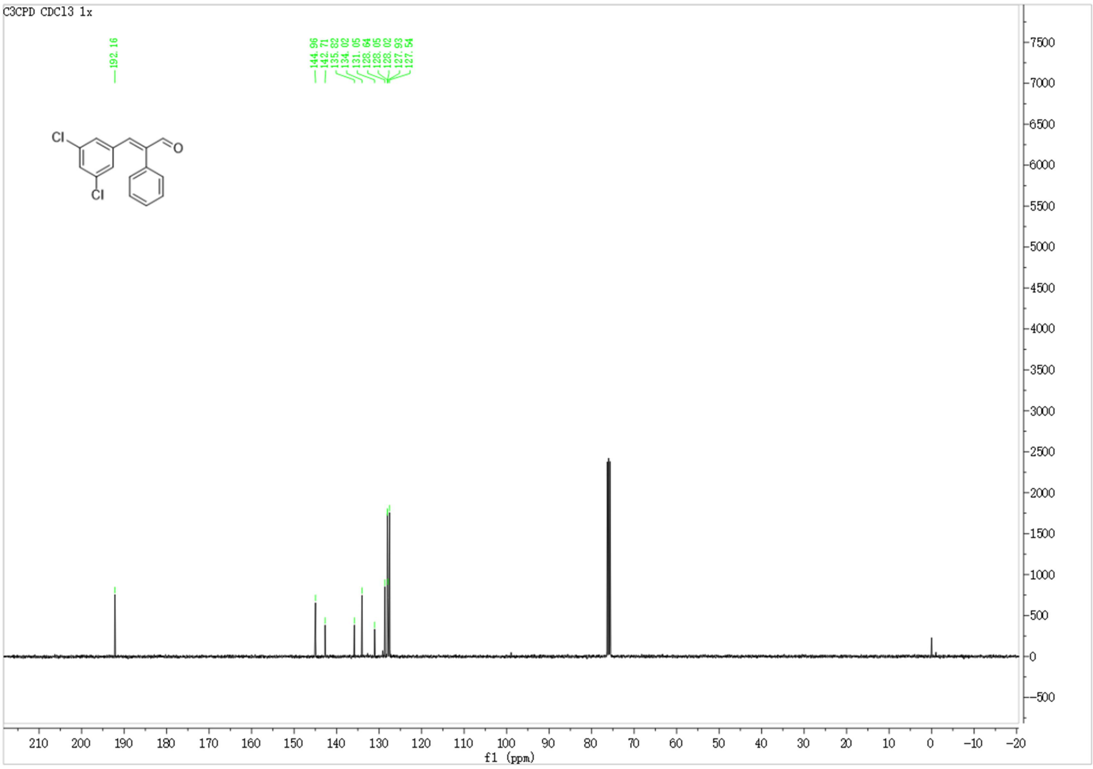

Supplementary Figure 84. ^{13}C NMR spectrum of 1x in $CDCl_3$.

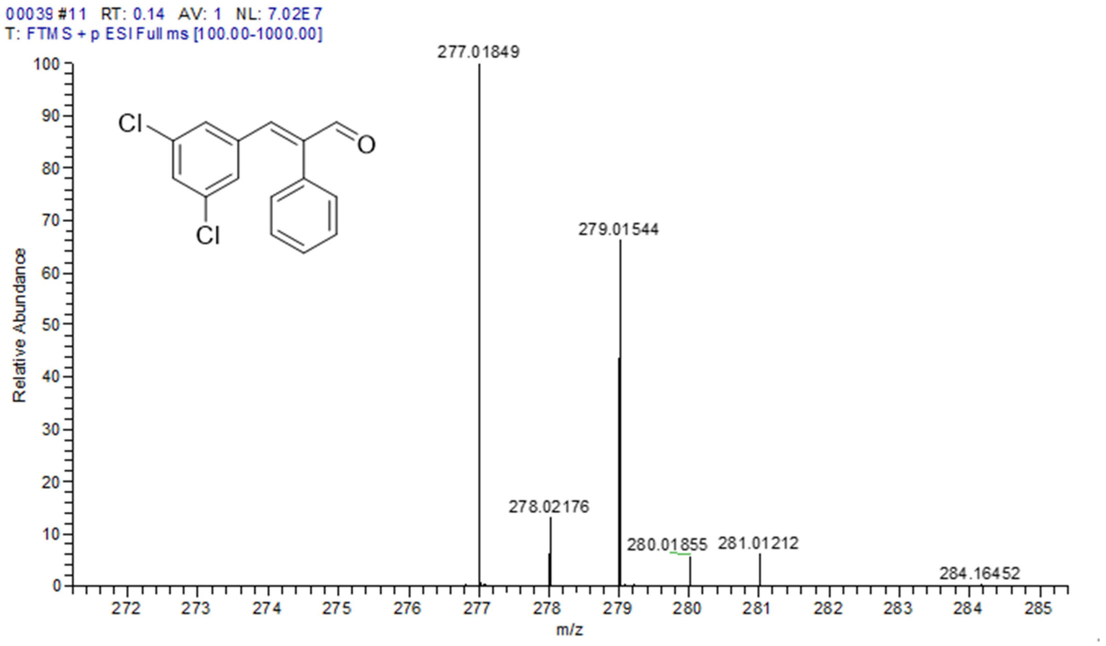

Supplementary Figure 85. HRMS of **1x**.

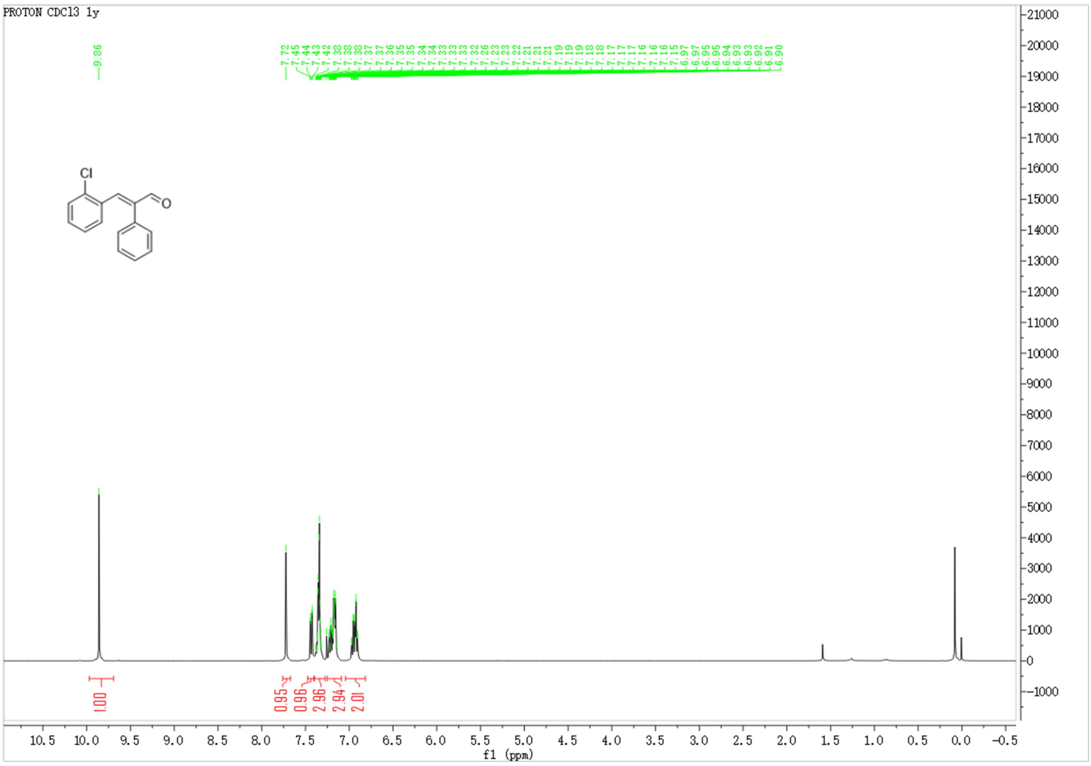

Supplementary Figure 86. ^{1}H NMR spectrum of **1y** in $CDCl_3$.

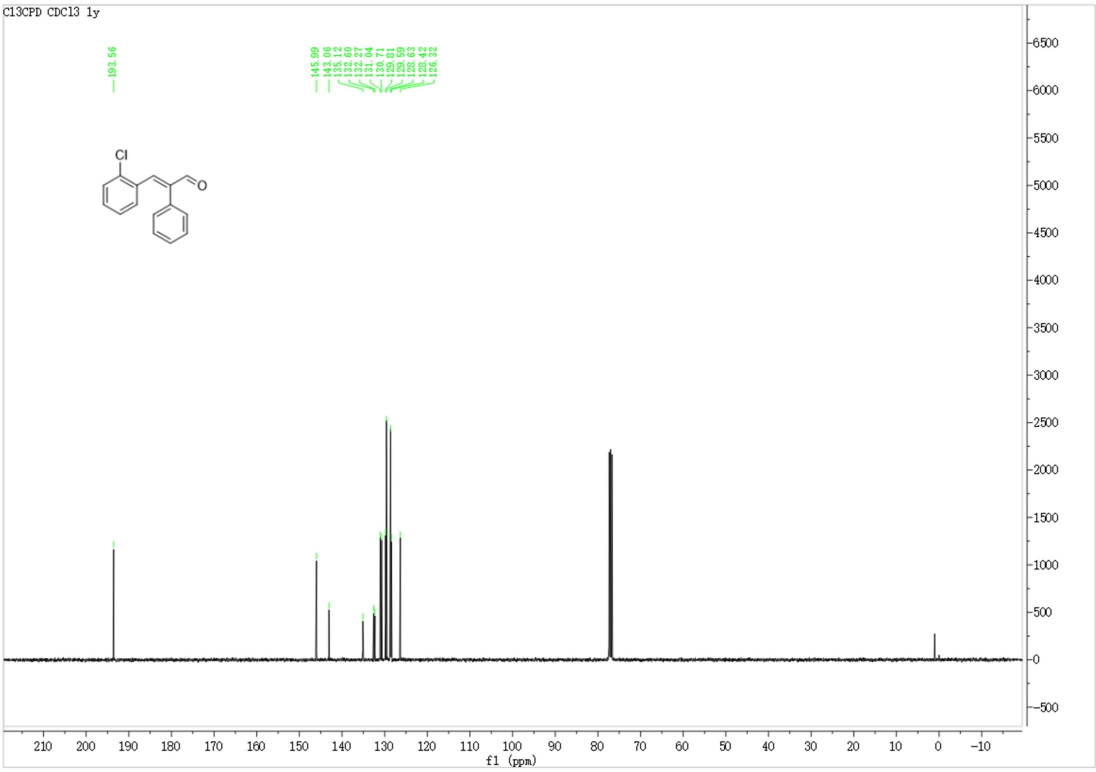

Supplementary Figure 87. ^{13}C NMR spectrum of **1y** in $CDCl_3$.

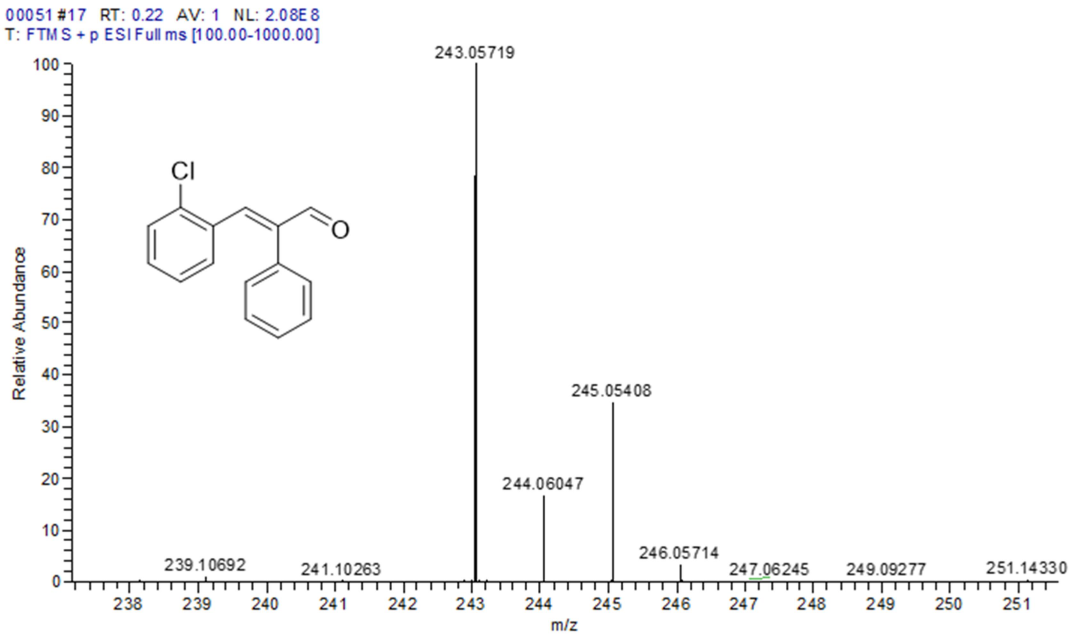

Supplementary Figure 88. HRMS of **1y**.

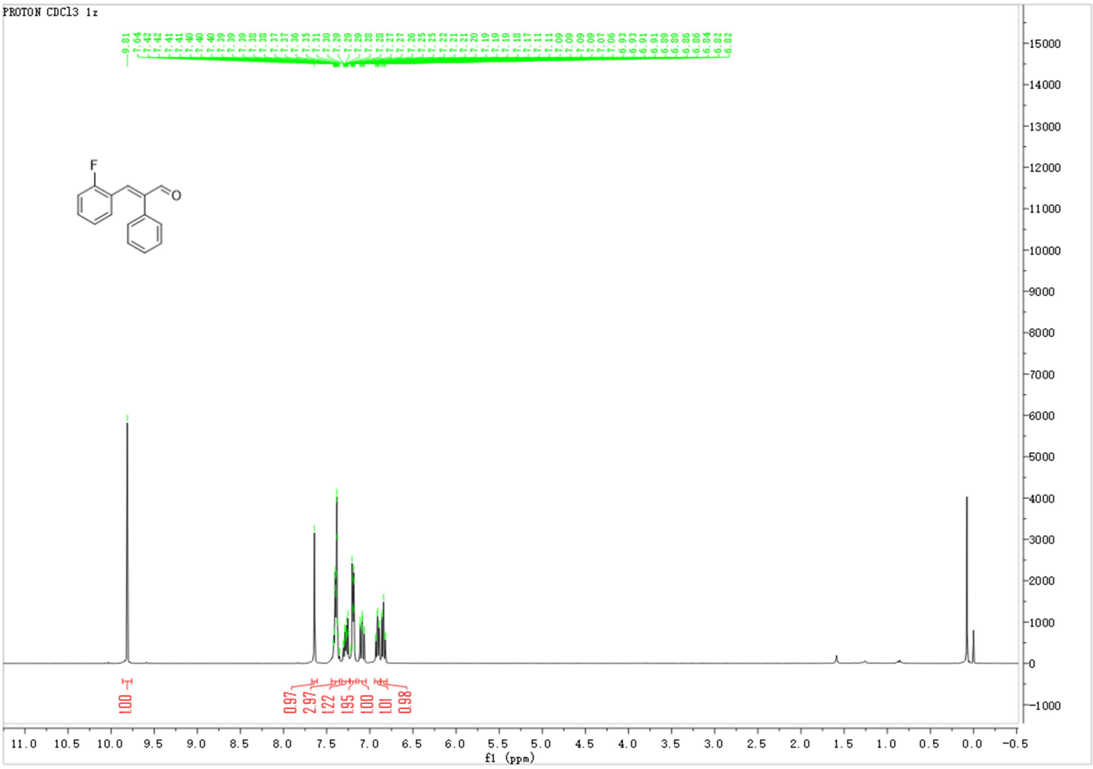

Supplementary Figure 89. ^{1}H NMR spectrum of **1z** in $CDCl_3$.

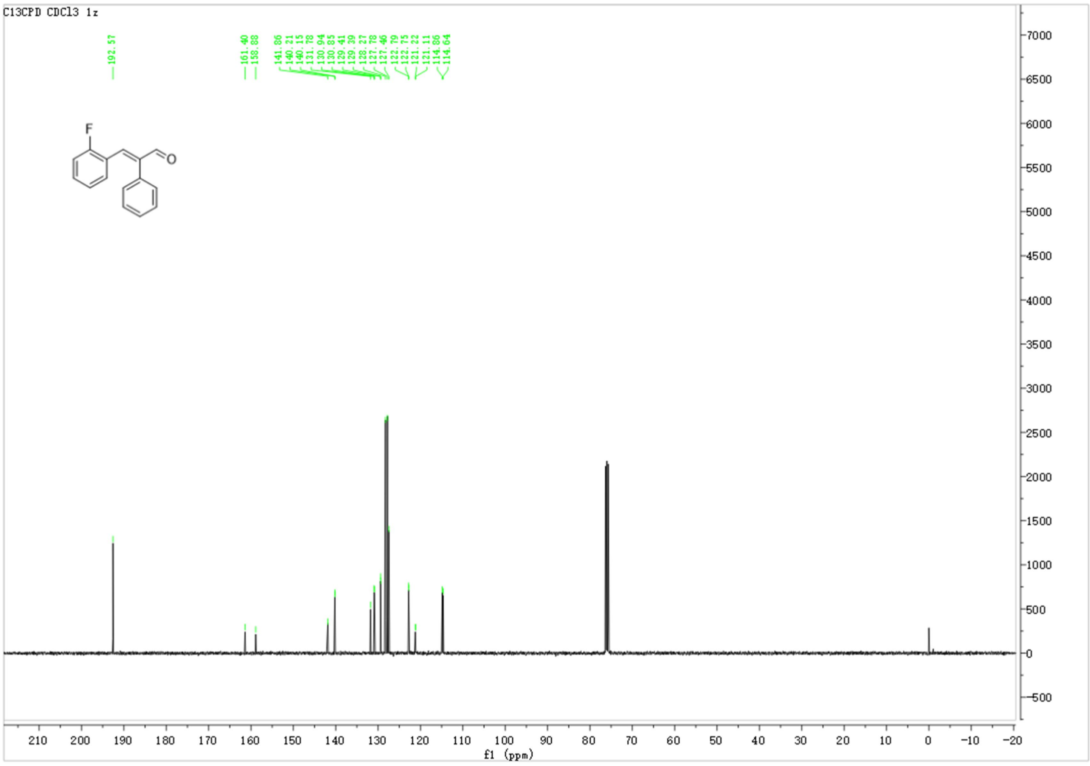

Supplementary Figure 90. ^{13}C NMR spectrum of **1z** in $CDCl_3$.

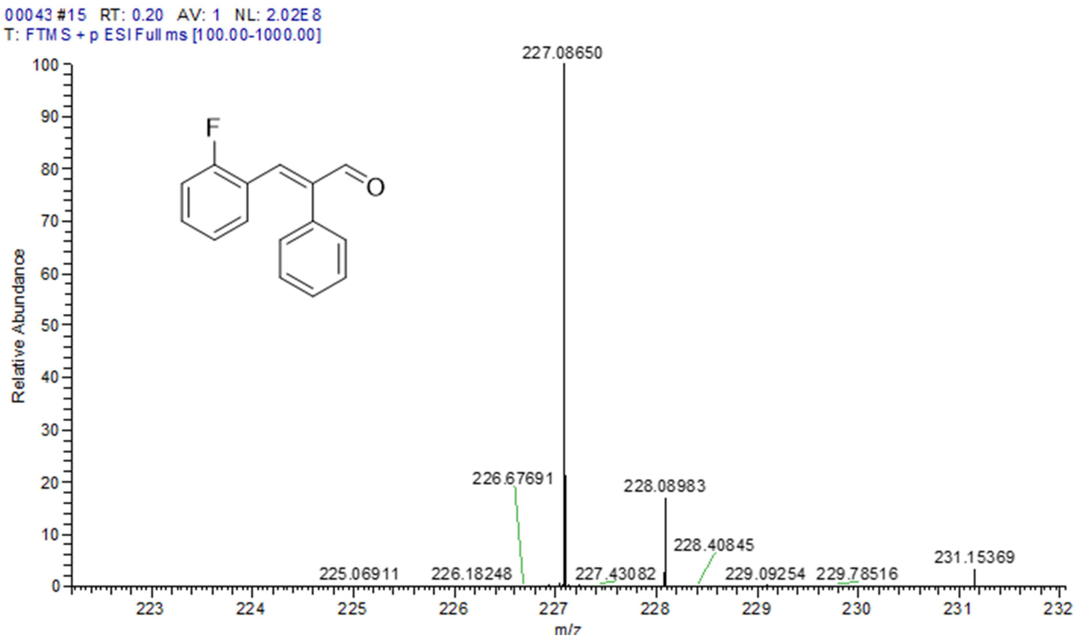

Supplementary Figure 91. HRMS of **1z**.

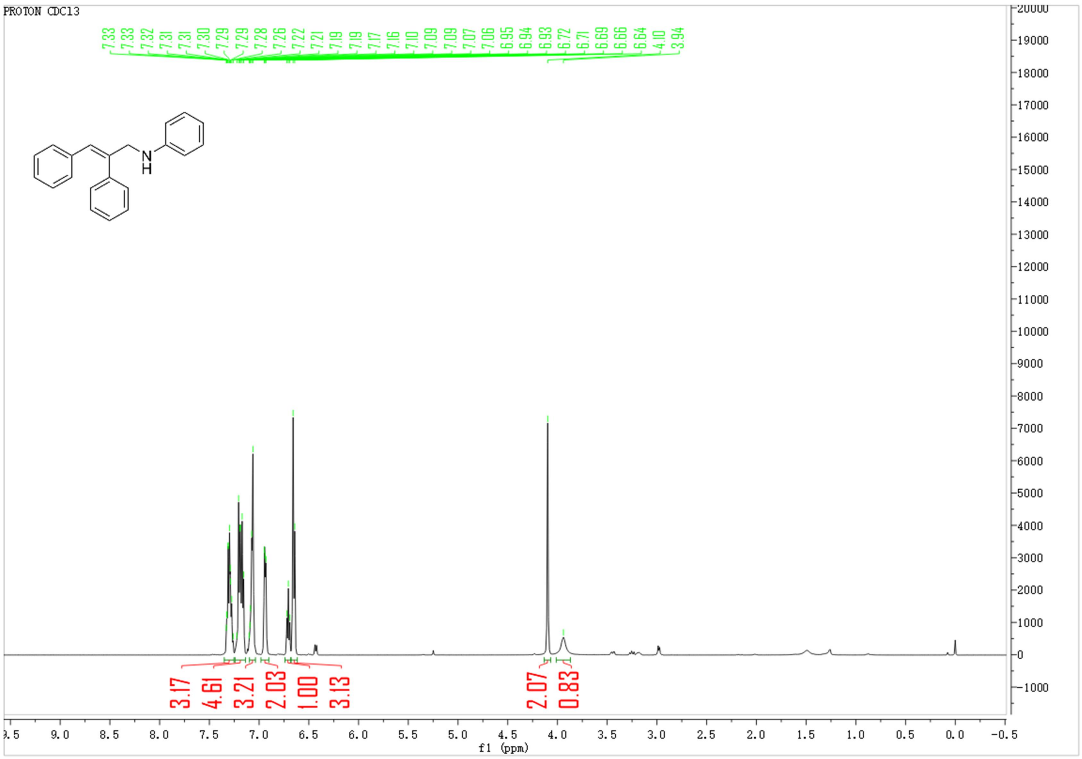

Supplementary Figure 92. ^{1}H NMR spectrum of **4** in $CDCl_3$.

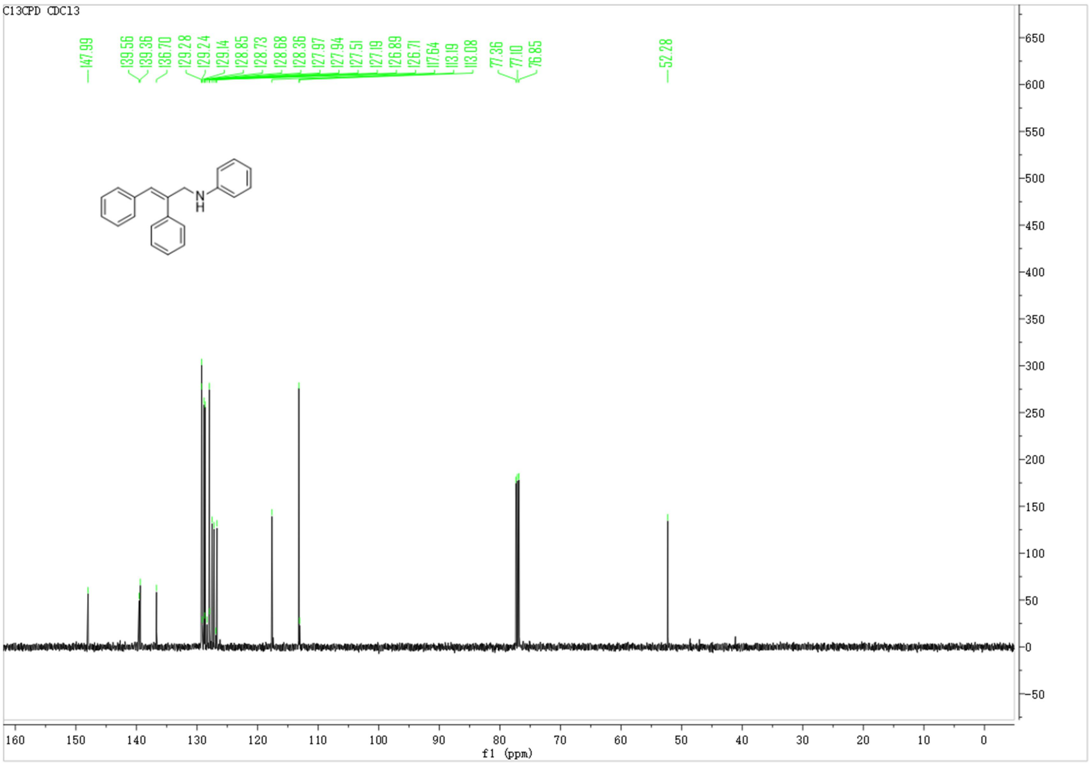

Supplementary Figure 93. ^{13}C NMR spectrum of **4** in $CDCl_3$.

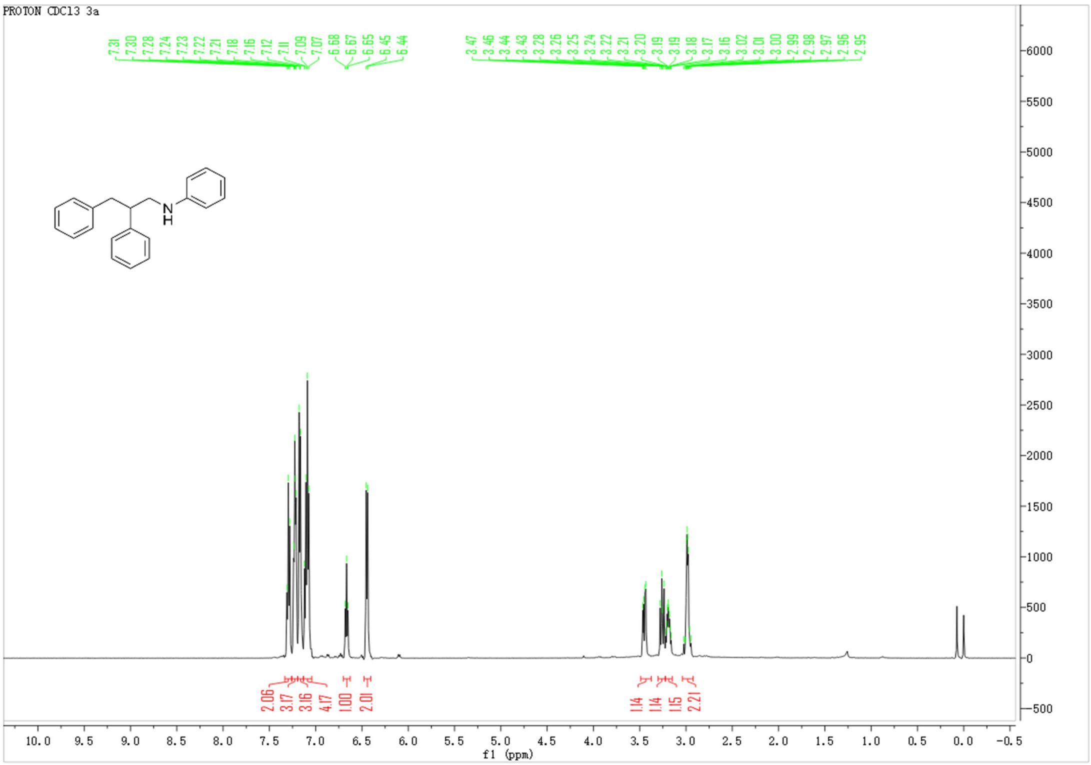

Supplementary Figure 94. ^{1}H NMR spectrum of **3a** in $CDCl_3$.

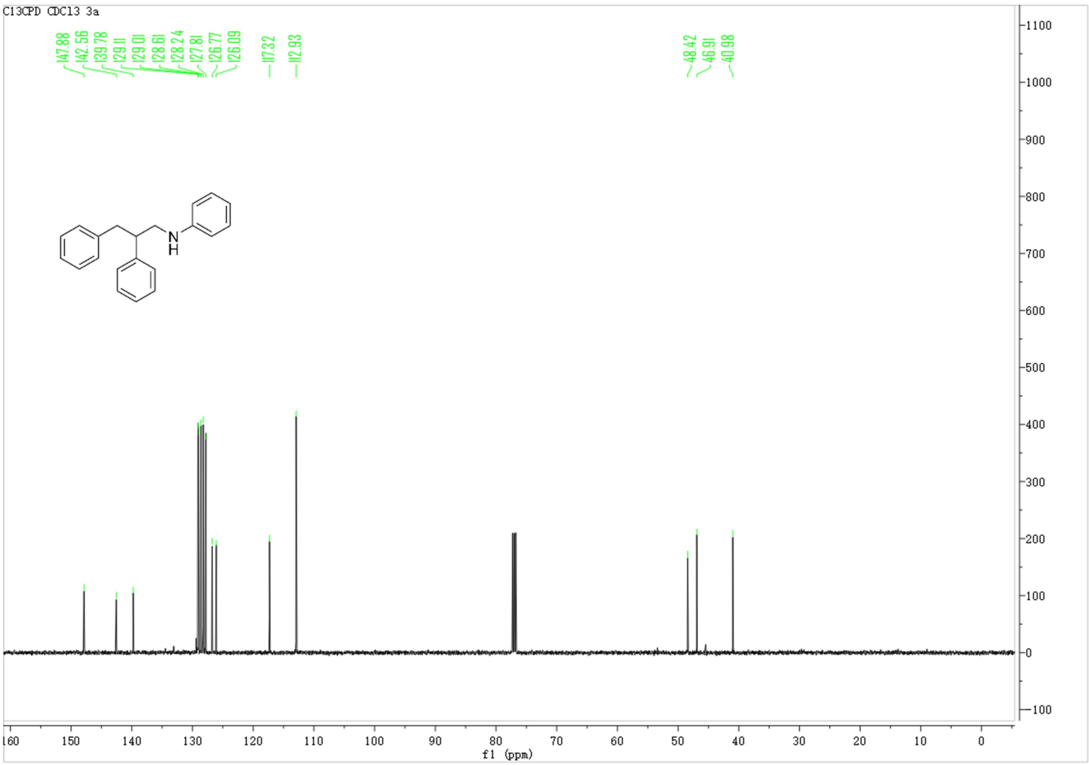

Supplementary Figure 95. ^{13}C NMR spectrum of **3a** in $CDCl_3$.

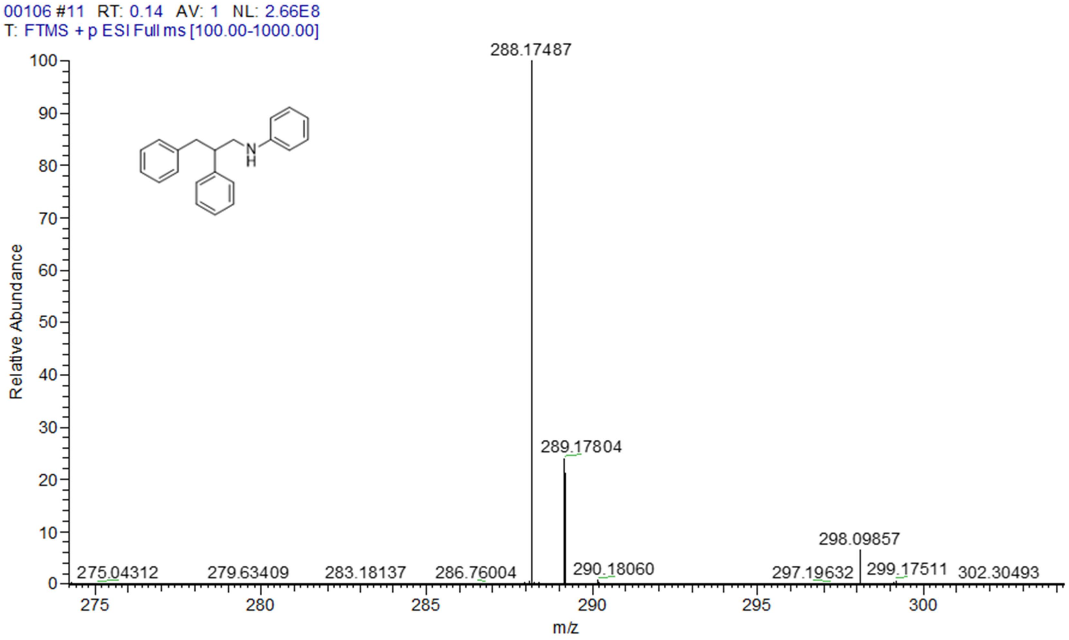

Supplementary Figure 96. HRMS of **3a**.

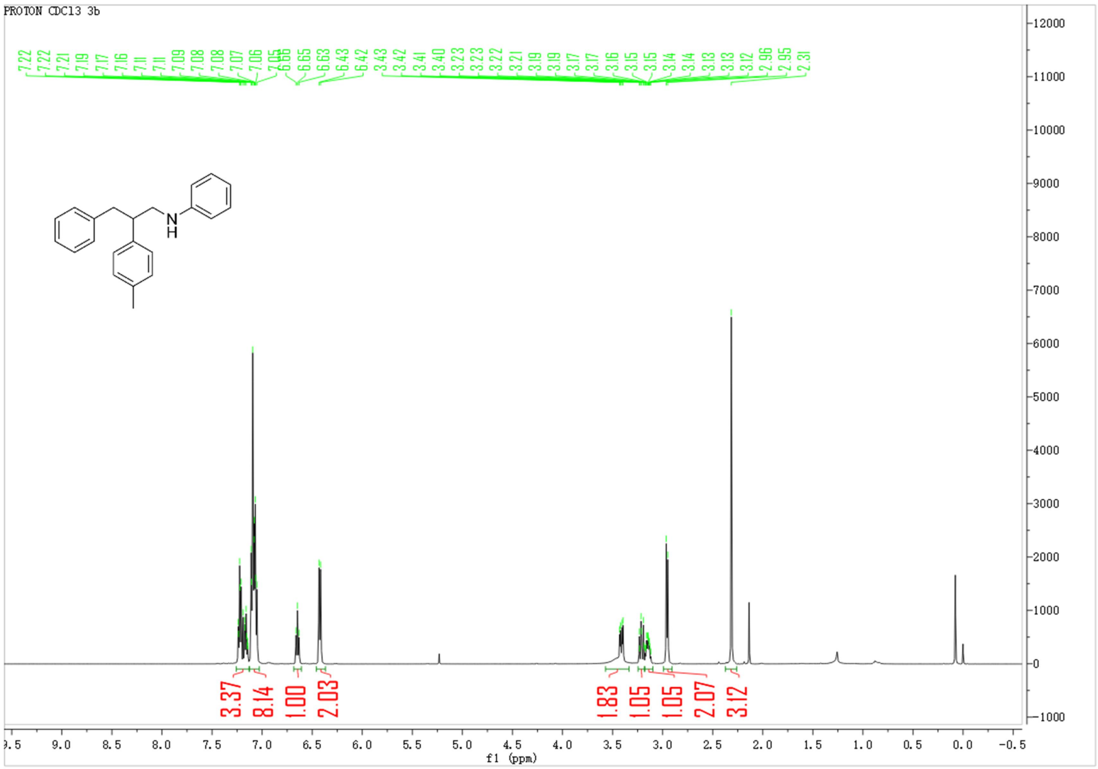

Supplementary Figure 97. ^{1}H NMR spectrum of **3b** in $CDCl_3$.

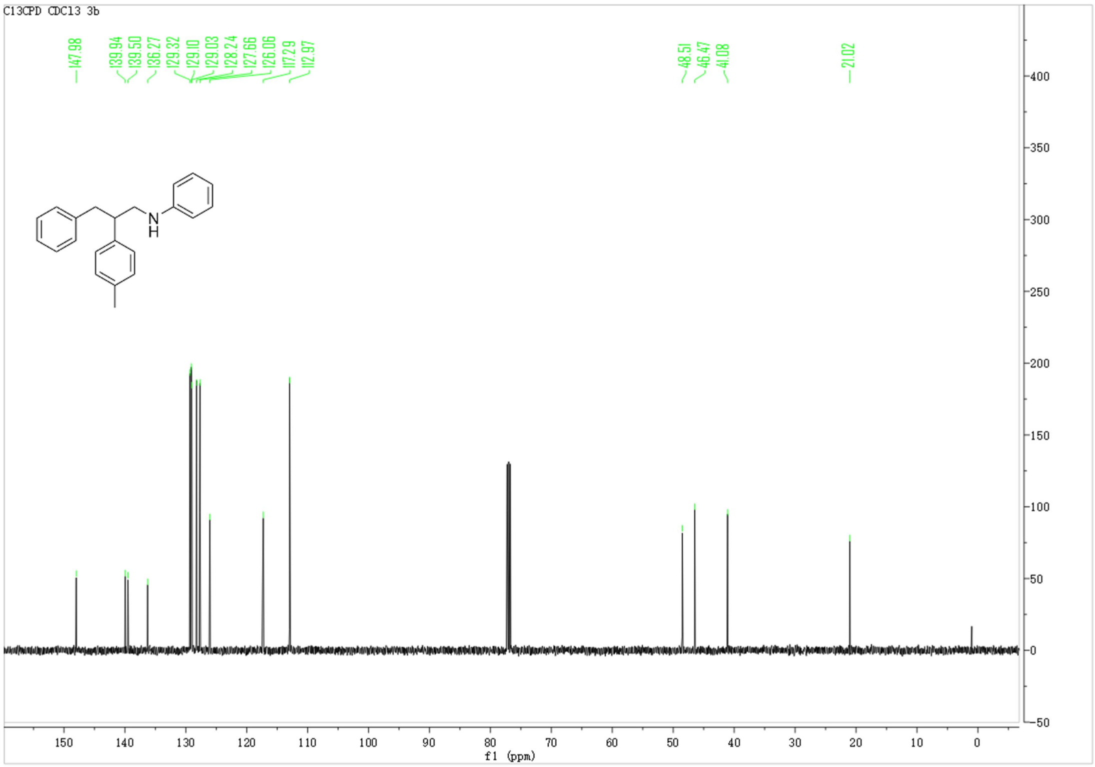

Supplementary Figure 98. ^{13}C NMR spectrum of **3b** in $CDCl_3$.

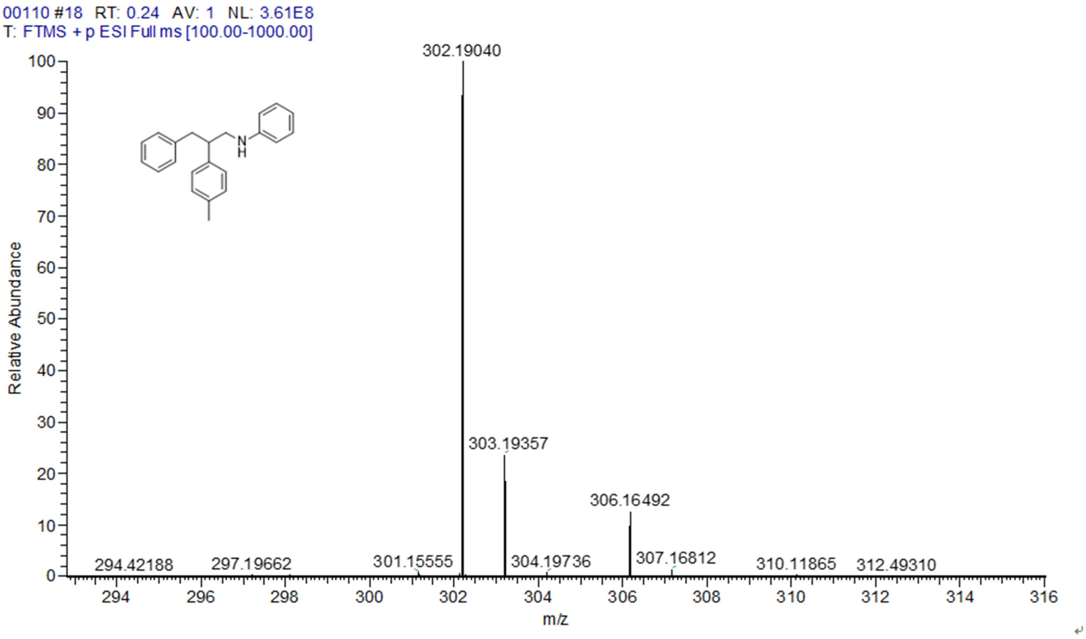

Supplementary Figure 99. HRMS of **3d**.

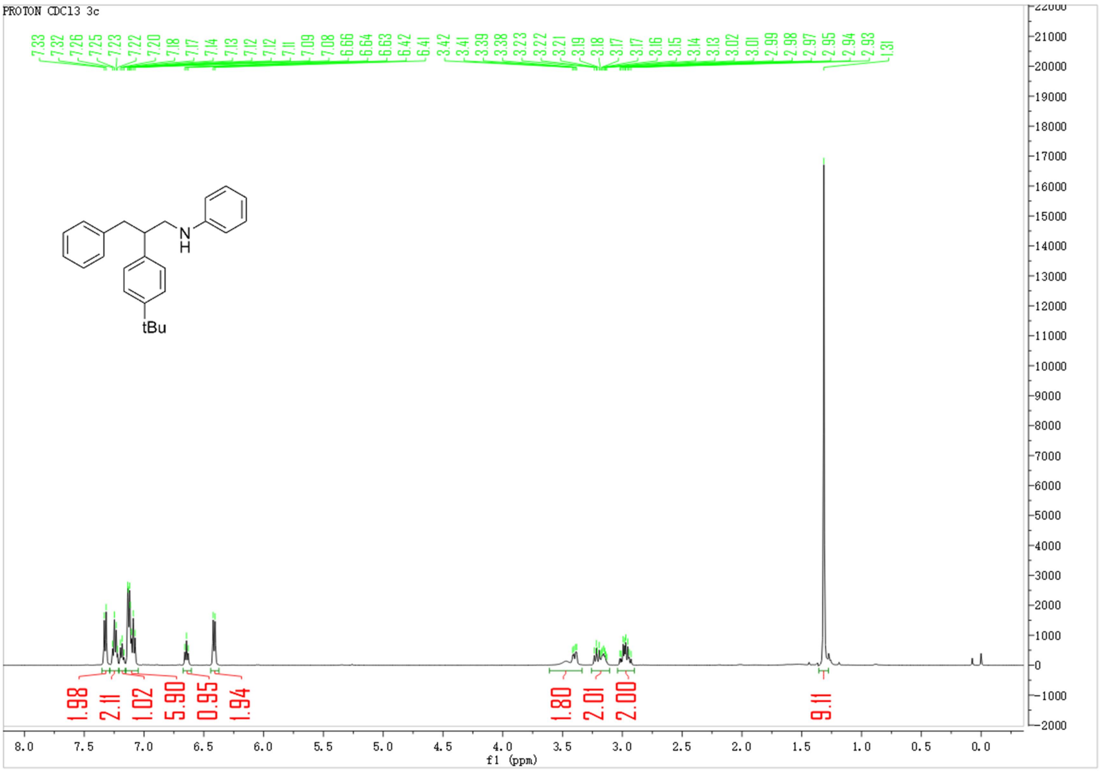

Supplementary Figure 100. ^{1}H NMR spectrum of **3c** in $CDCl_3$.

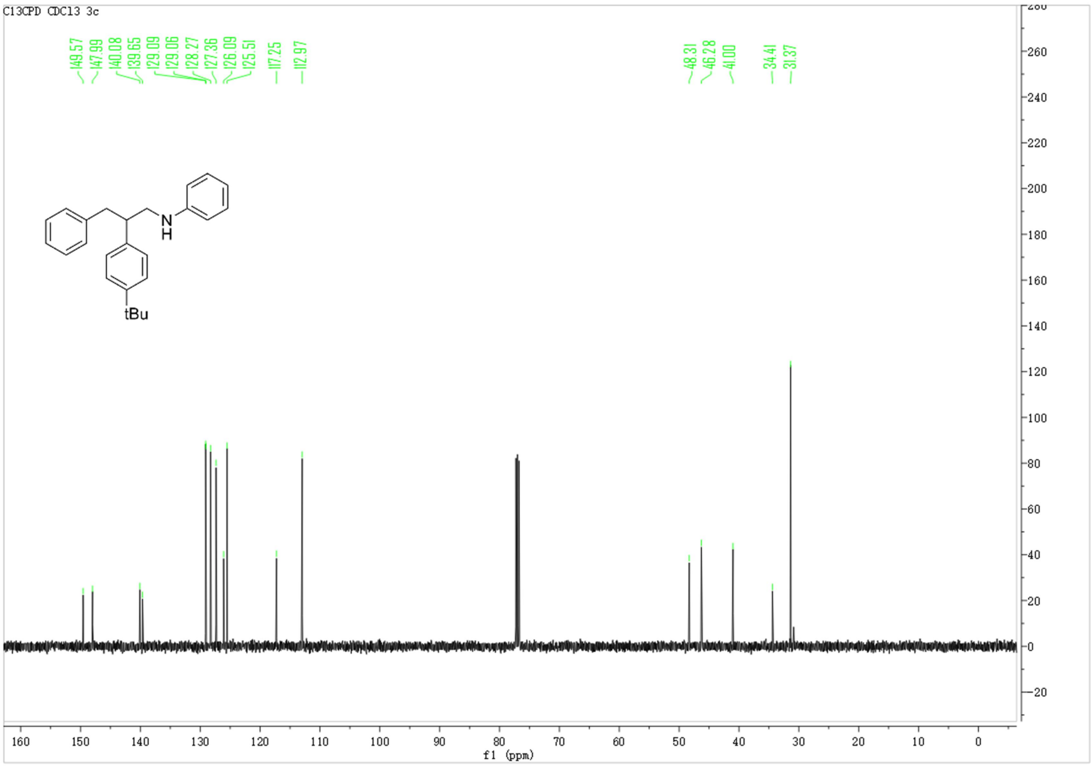

Supplementary Figure 101. ^{13}C NMR spectrum of **3c** in $CDCl_3$.

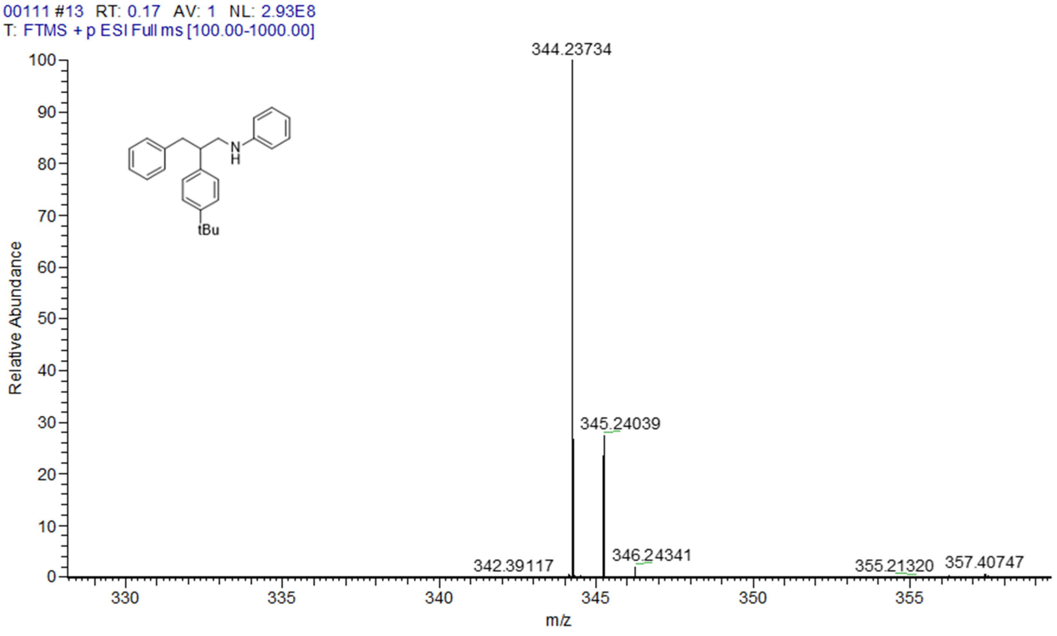

Supplementary Figure 102. HRMS of **3c**.

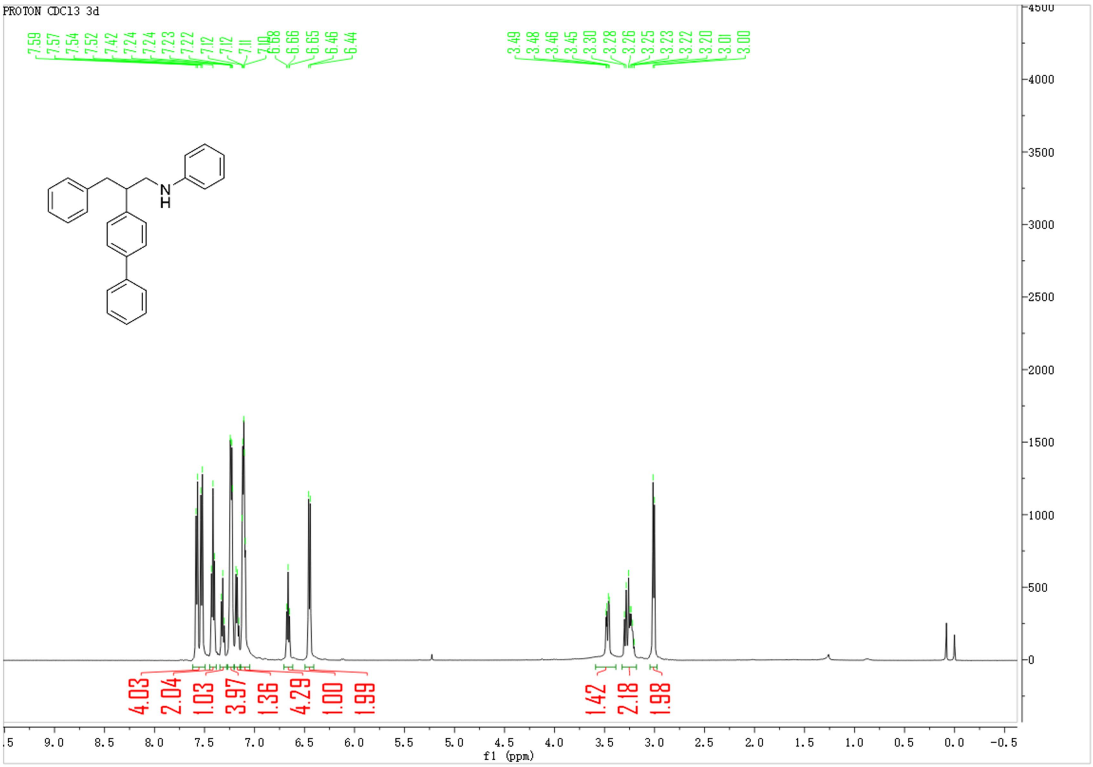

Supplementary Figure 103. ^{1}H NMR spectrum of **3d** in $CDCl_3$.

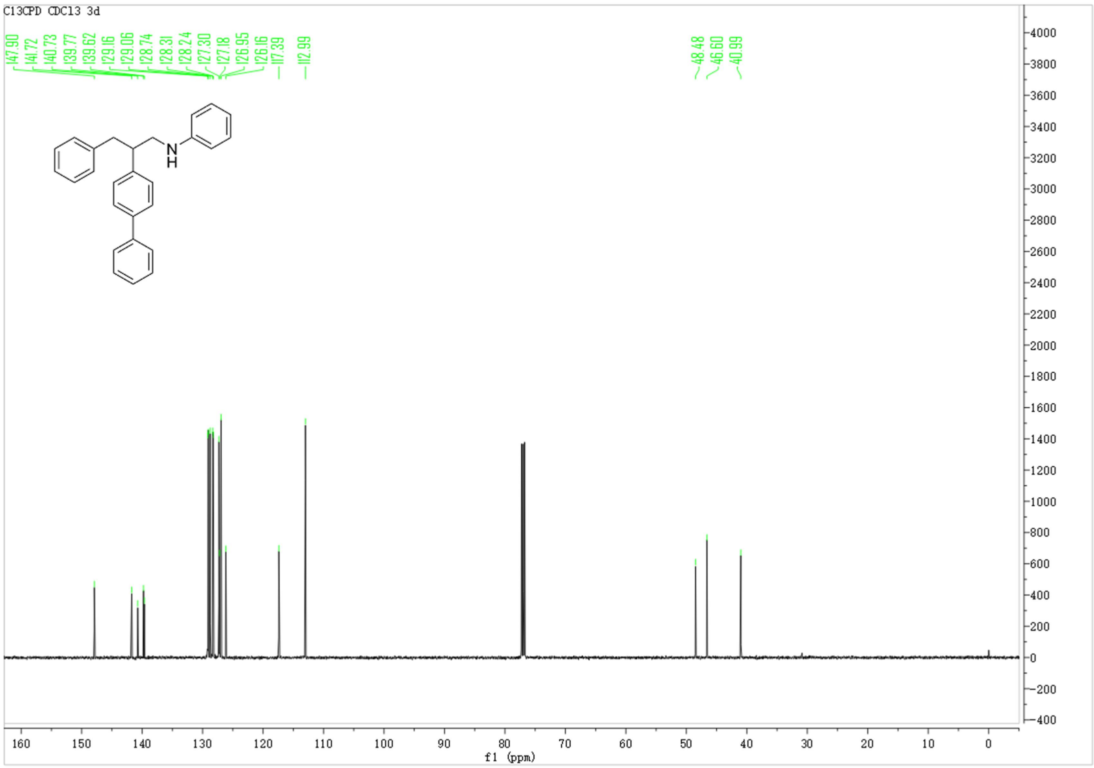

Supplementary Figure 104. ^{13}C NMR spectrum of **3d** in $CDCl_3$.

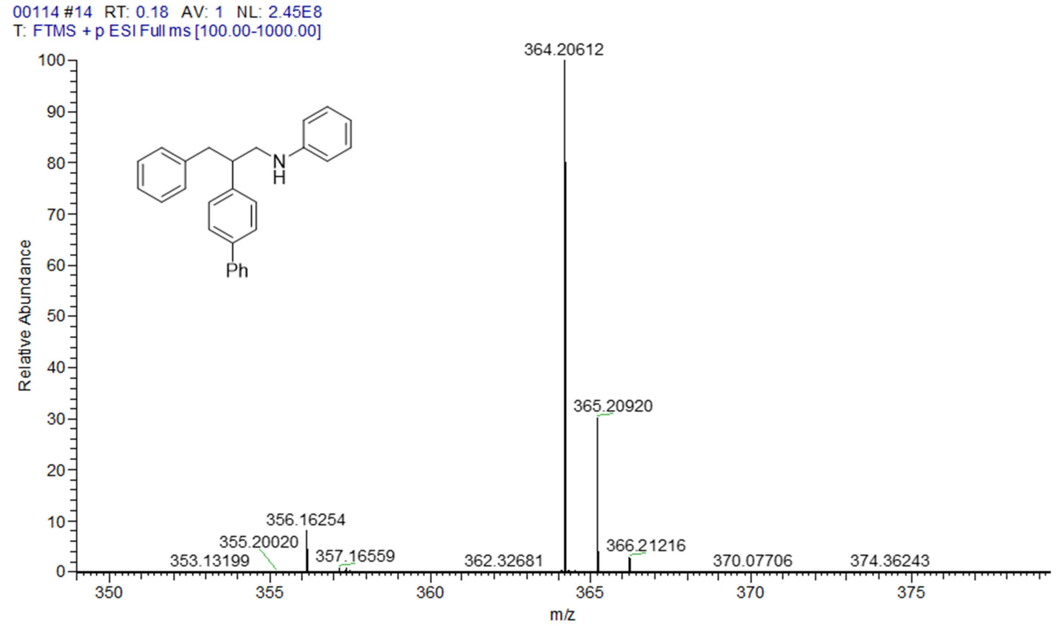

Supplementary Figure 105. HRMS of **3d**.

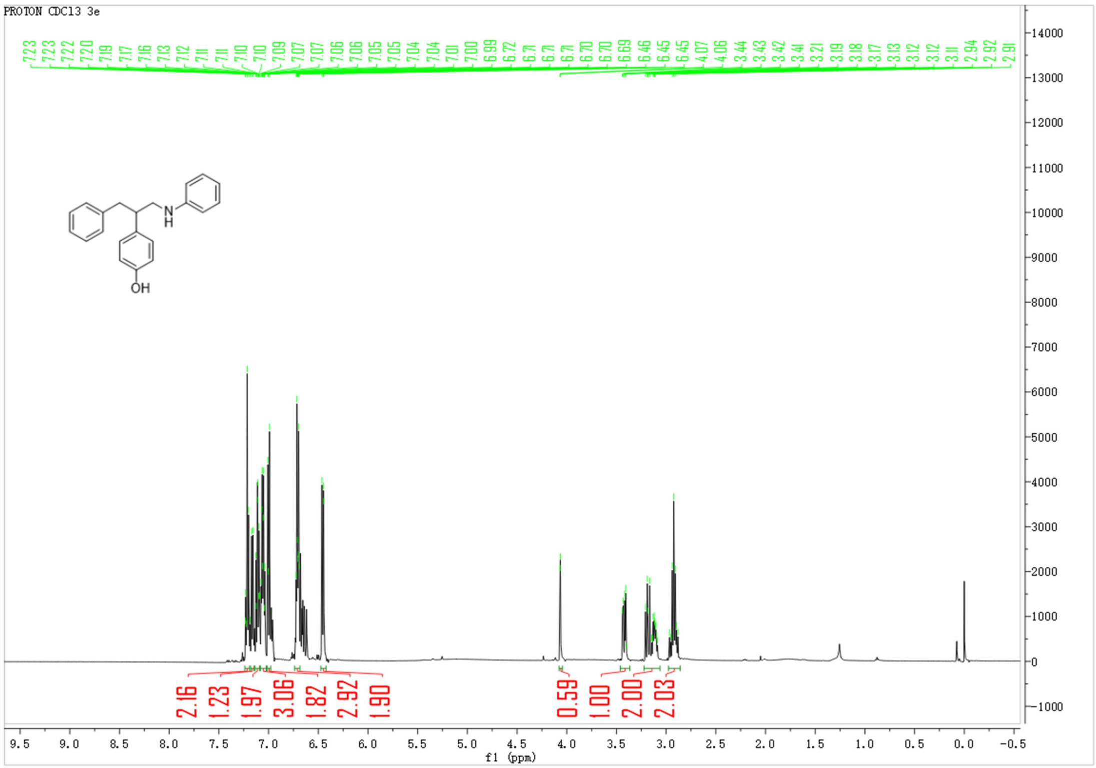

Supplementary Figure 106. ^{1}H NMR spectrum of **3e** in $CDCl_3$.

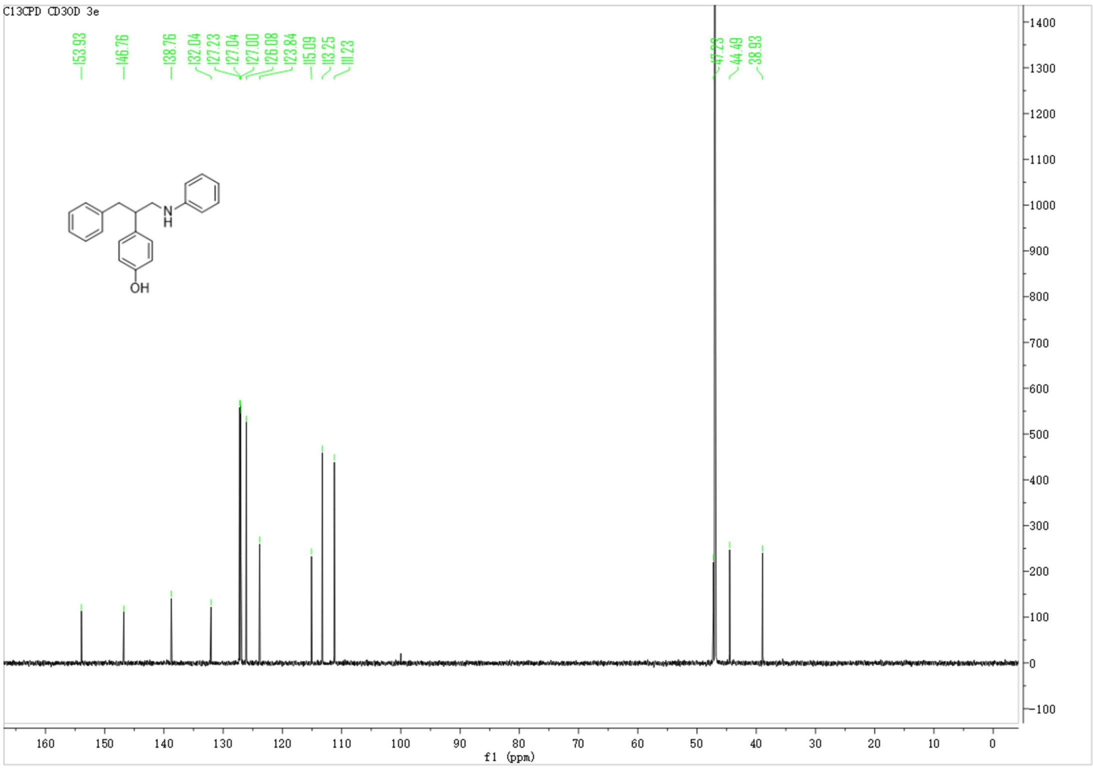

Supplementary Figure 107. ^{13}C NMR spectrum of **3e** in CD_3OD.

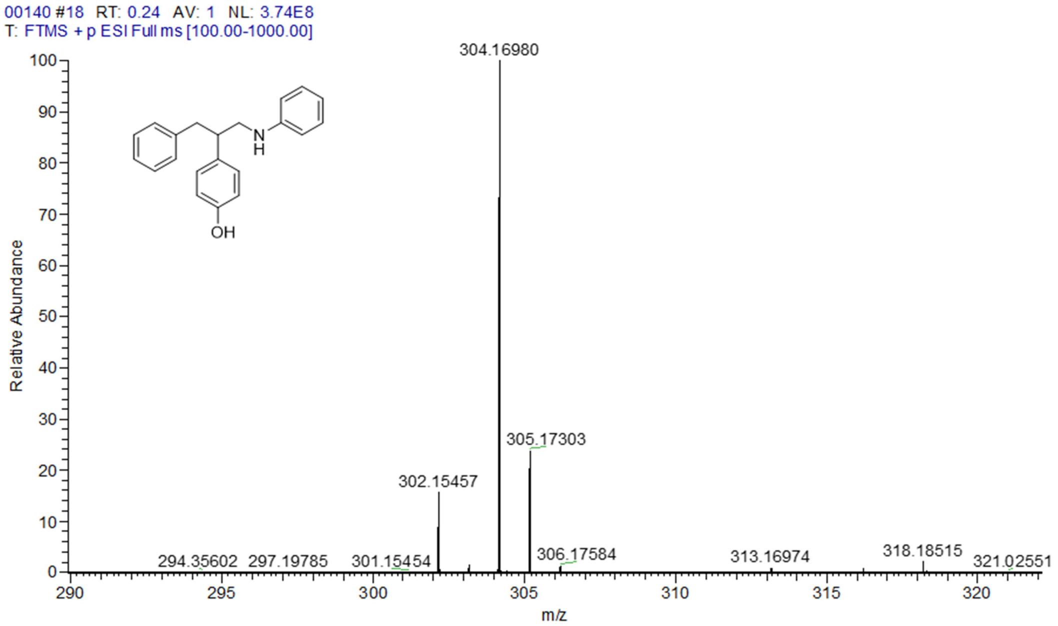

Supplementary Figure 108. HRMS of **3e**.

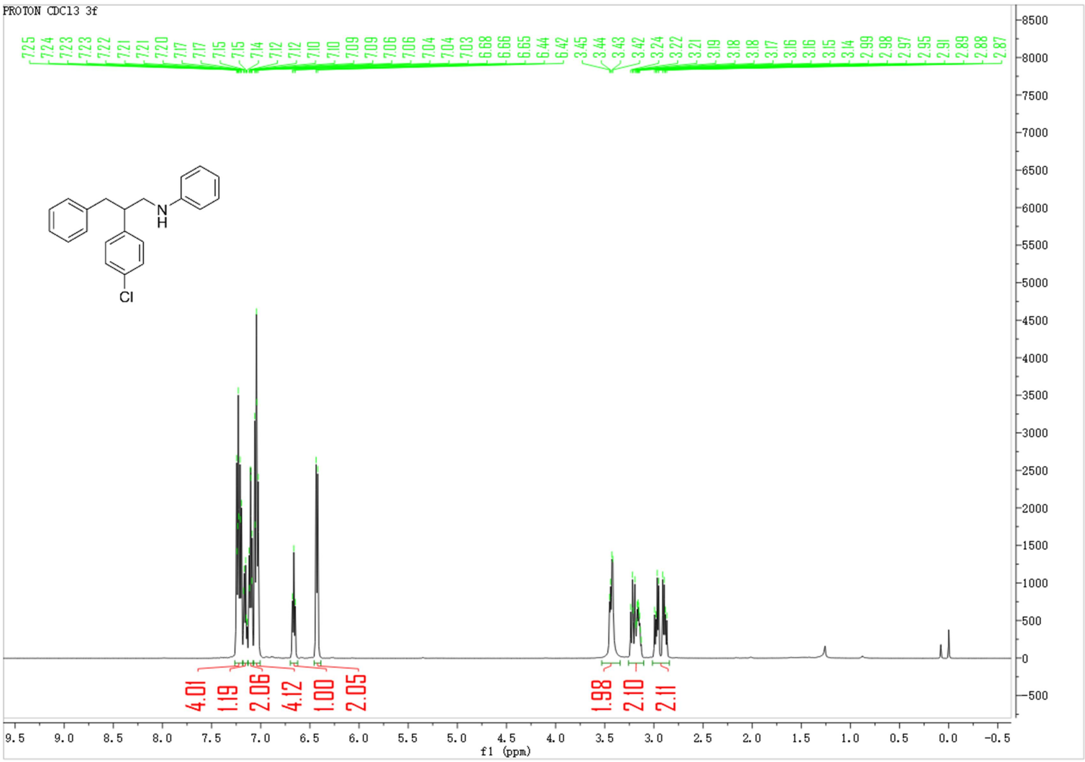

Supplementary Figure 109. ^{1}H NMR spectrum of **3f** in $CDCl_3$.

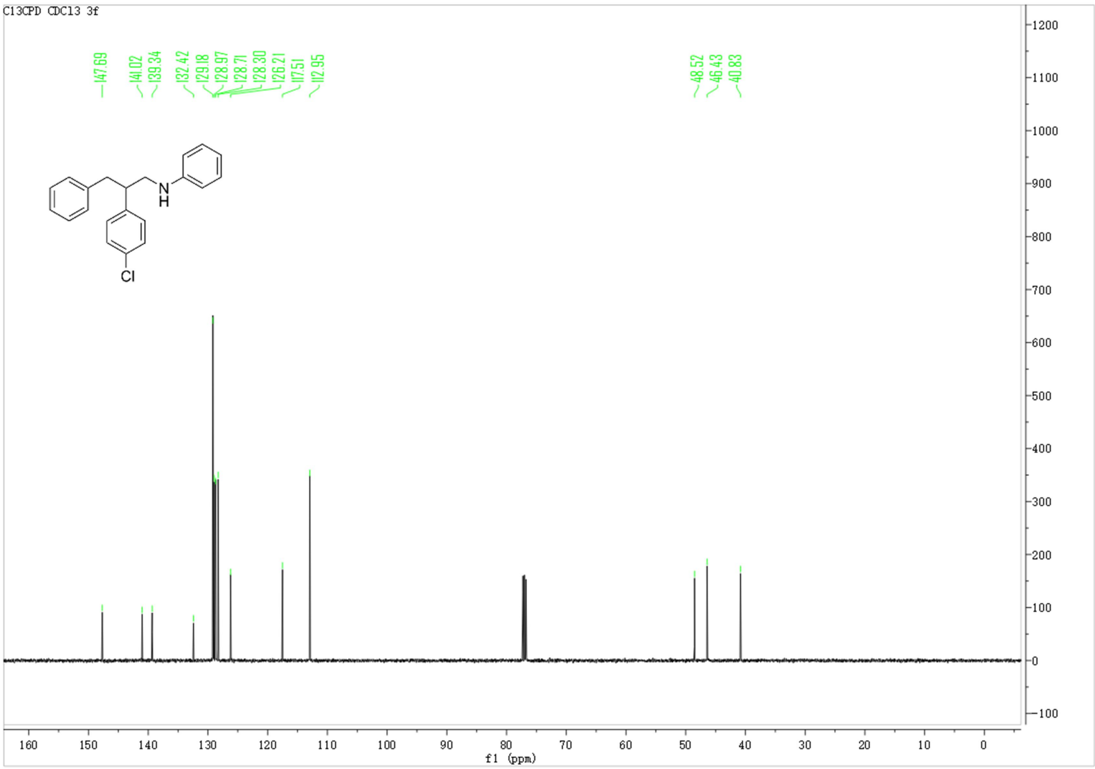

Supplementary Figure 110. ^{13}C NMR spectrum of **3f** in $CDCl_3$.

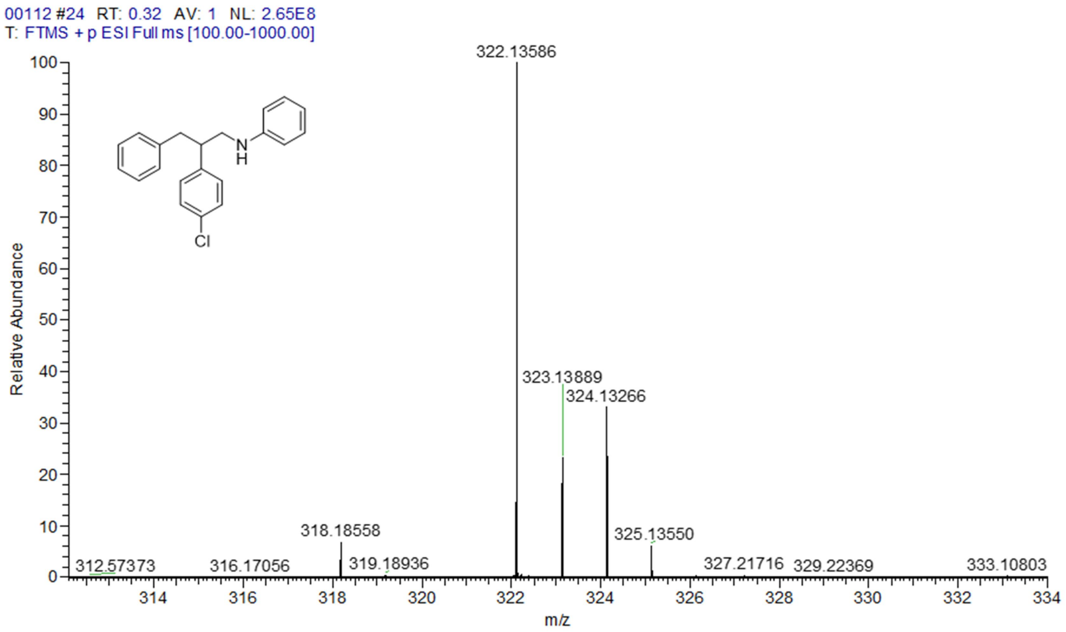

Supplementary Figure 111. HRMS of **1f**.

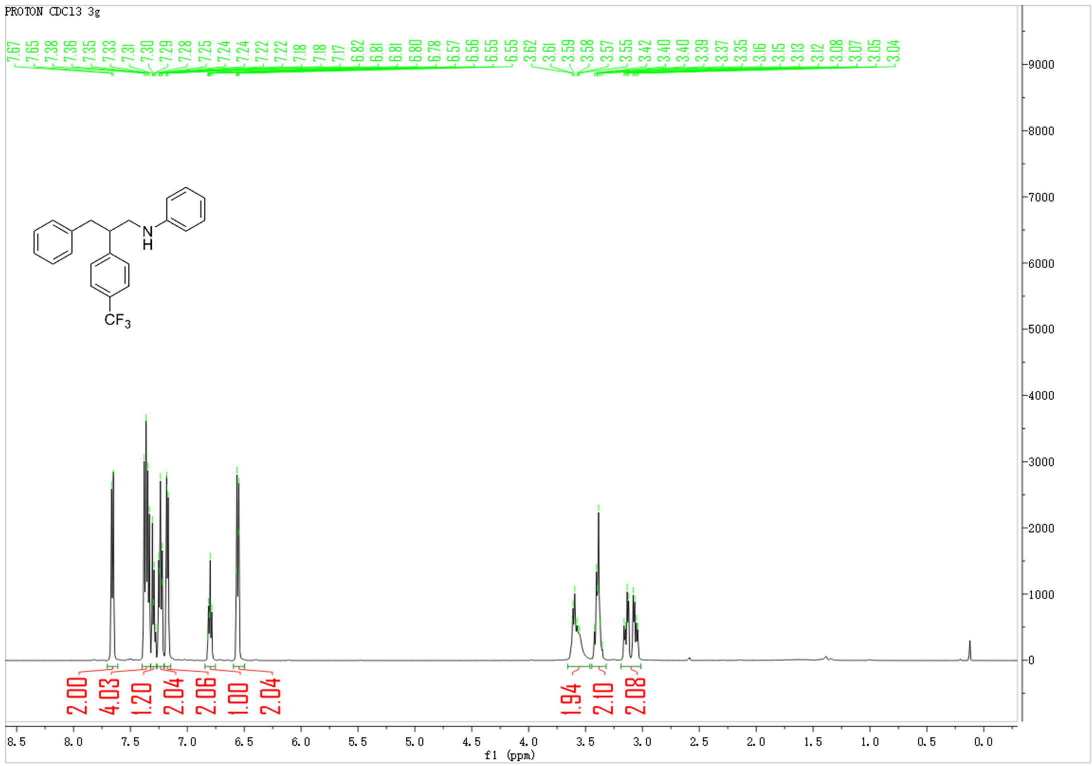

Supplementary Figure 112. ^{1}H NMR spectrum of **3g** in $CDCl_3$.

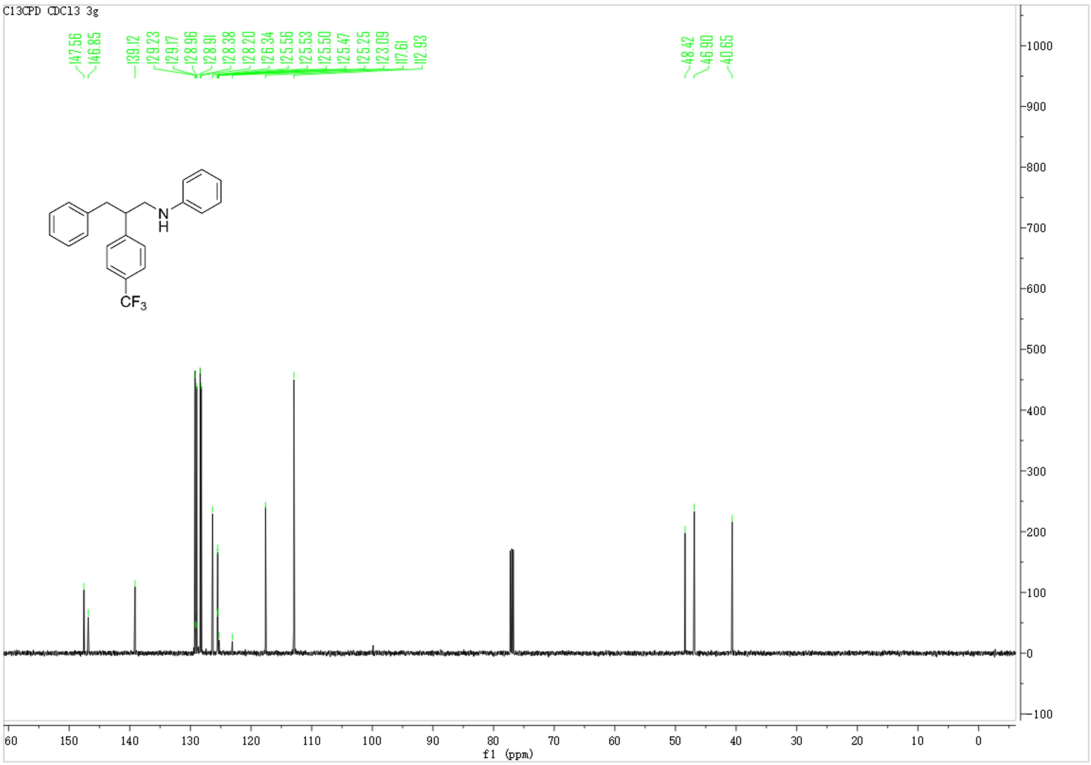

Supplementary Figure 113. ^{13}C NMR spectrum of **3g** in $CDCl_3$.

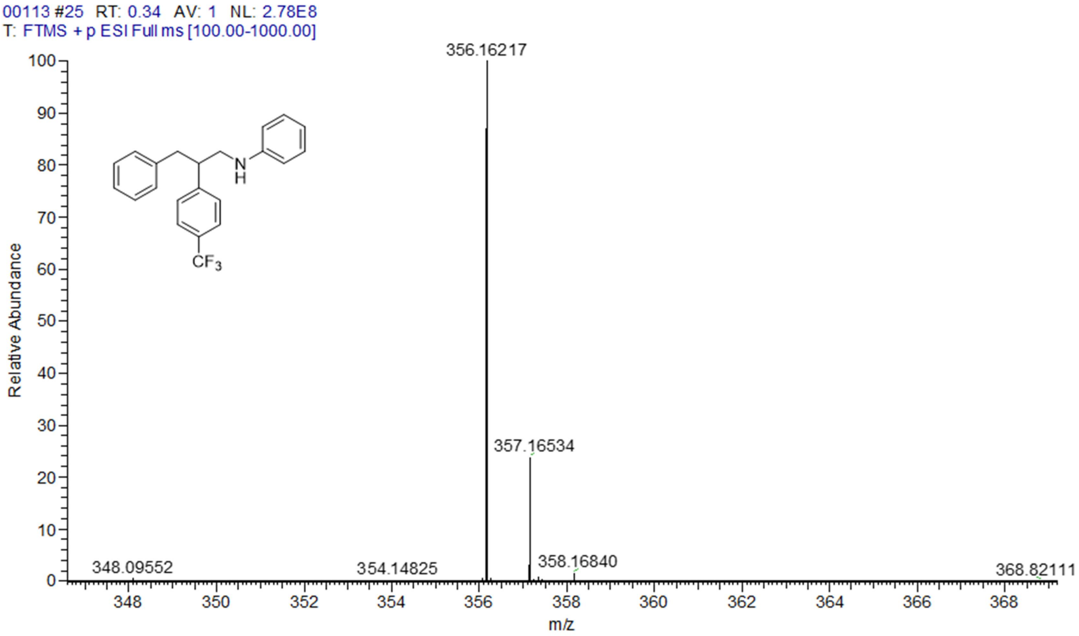

Supplementary Figure 114. HRMS of **3g**.

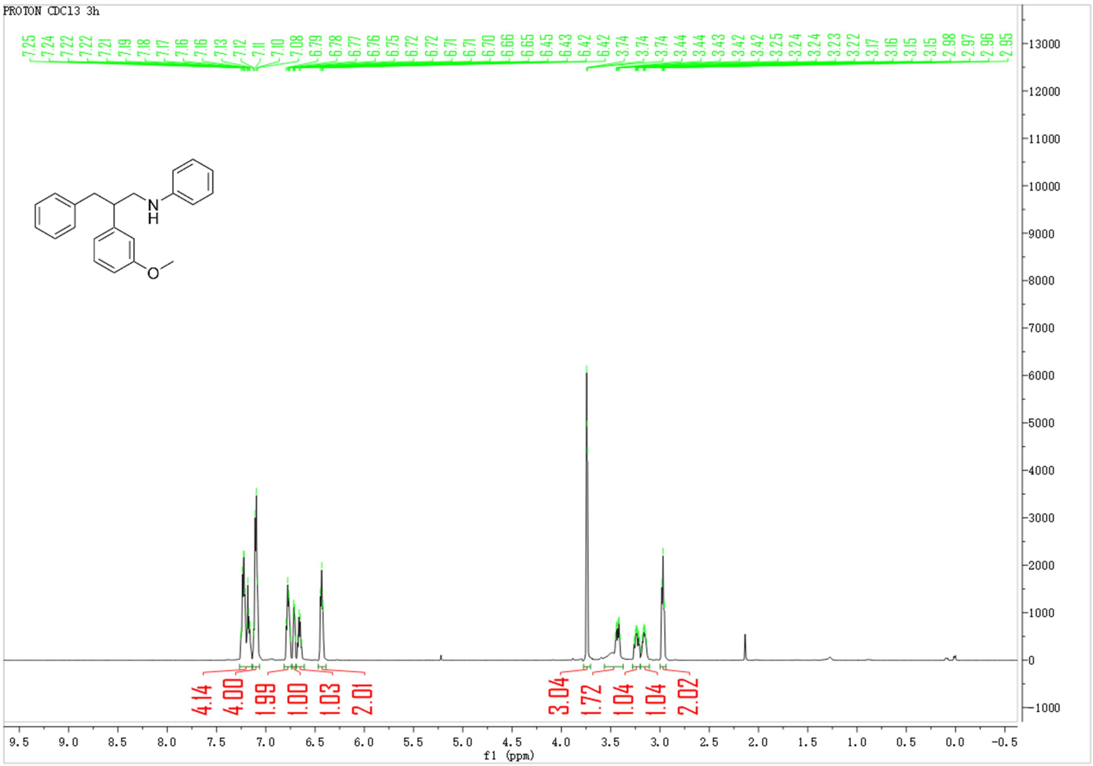

Supplementary Figure 115. ^{1}H NMR spectrum of **3h** in $CDCl_3$.

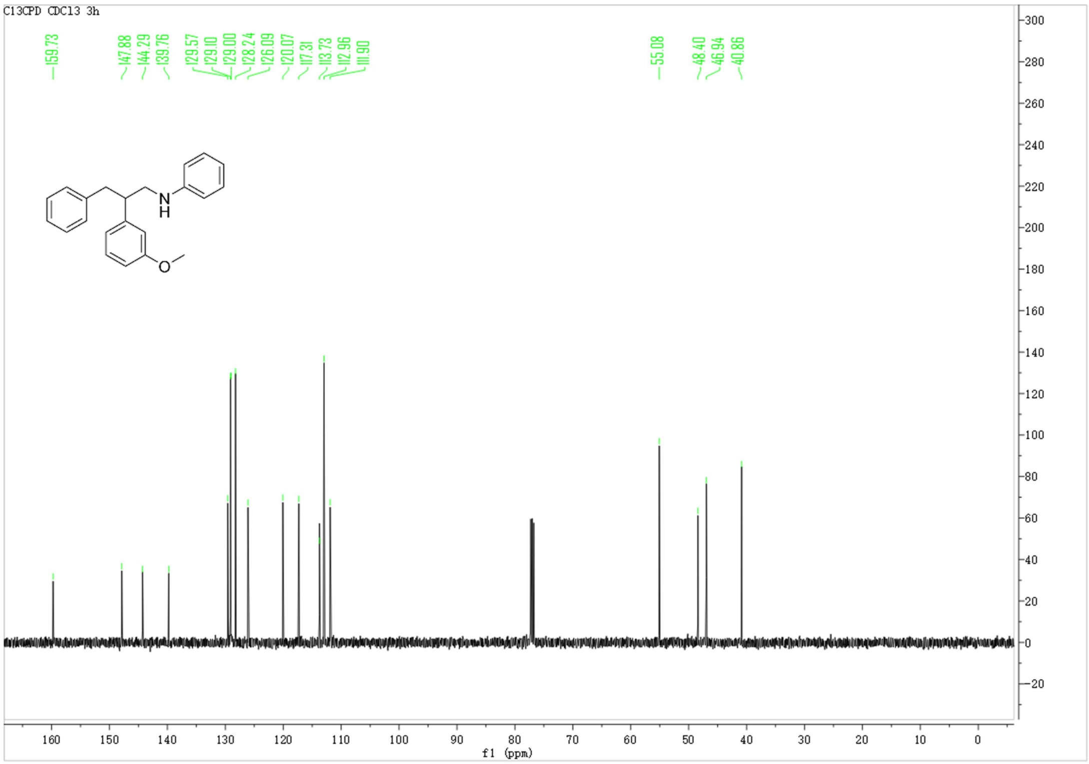

Supplementary Figure 116. ^{13}C NMR spectrum of **3h** in $CDCl_3$.

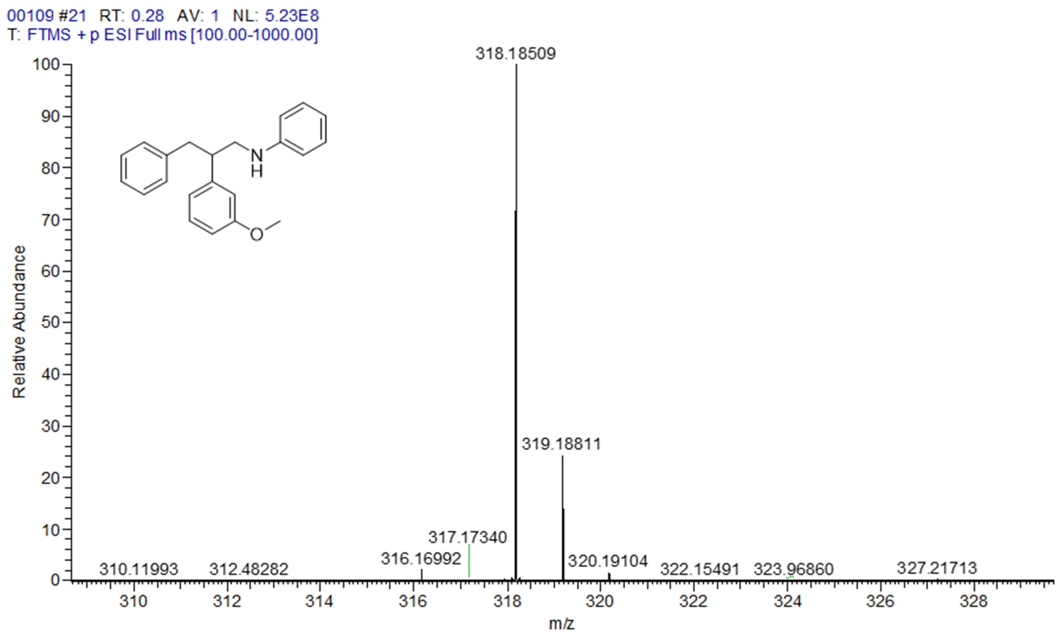

Supplementary Figure 117. HRMS of **3h**.

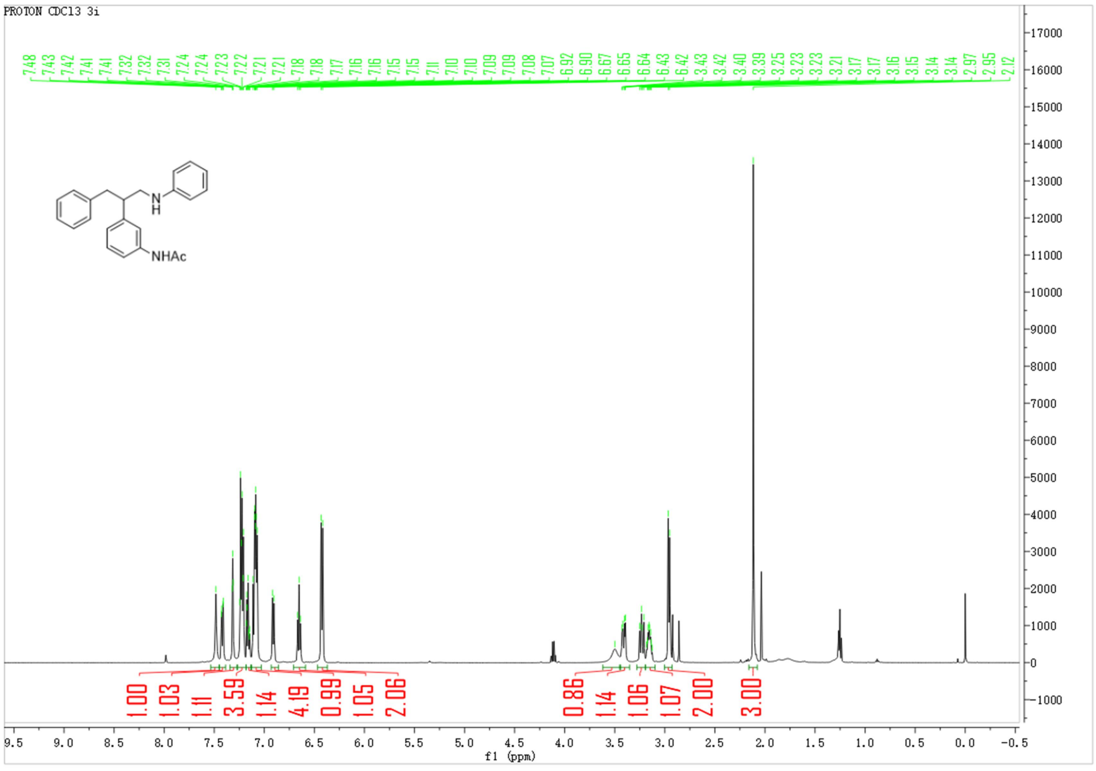

Supplementary Figure 118. ^{1}H NMR spectrum of **3i** in $CDCl_3$.

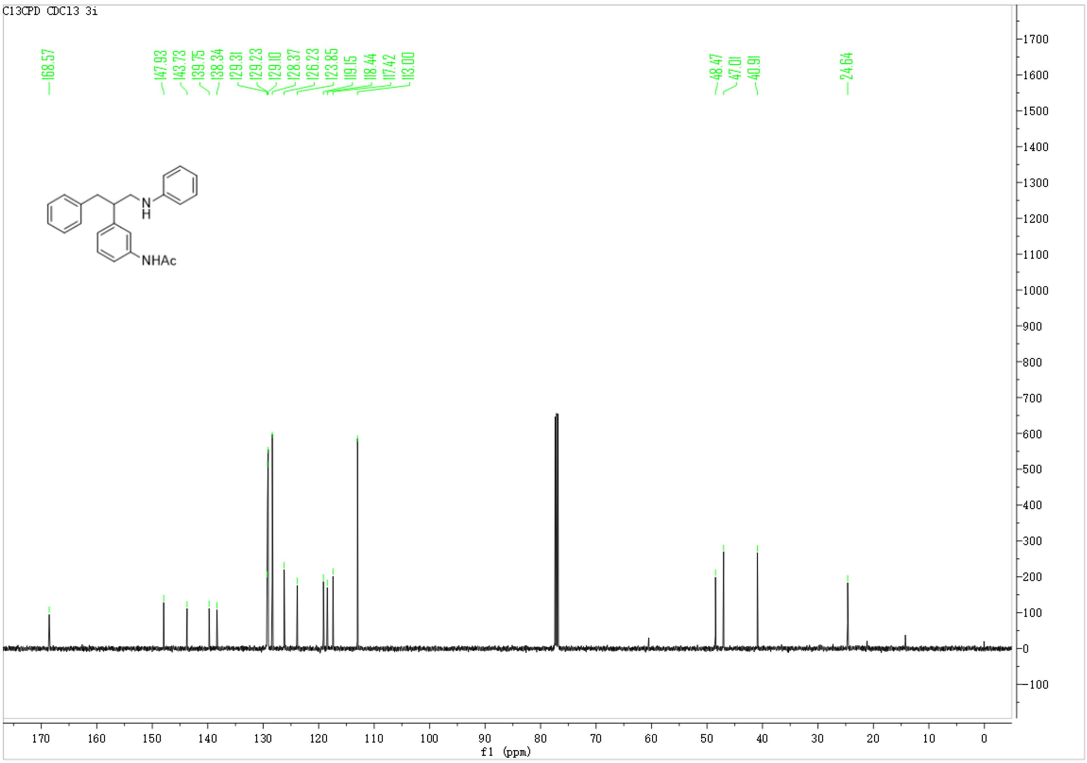

Supplementary Figure 119. ^{13}C NMR spectrum of **3i** in $CDCl_3$.

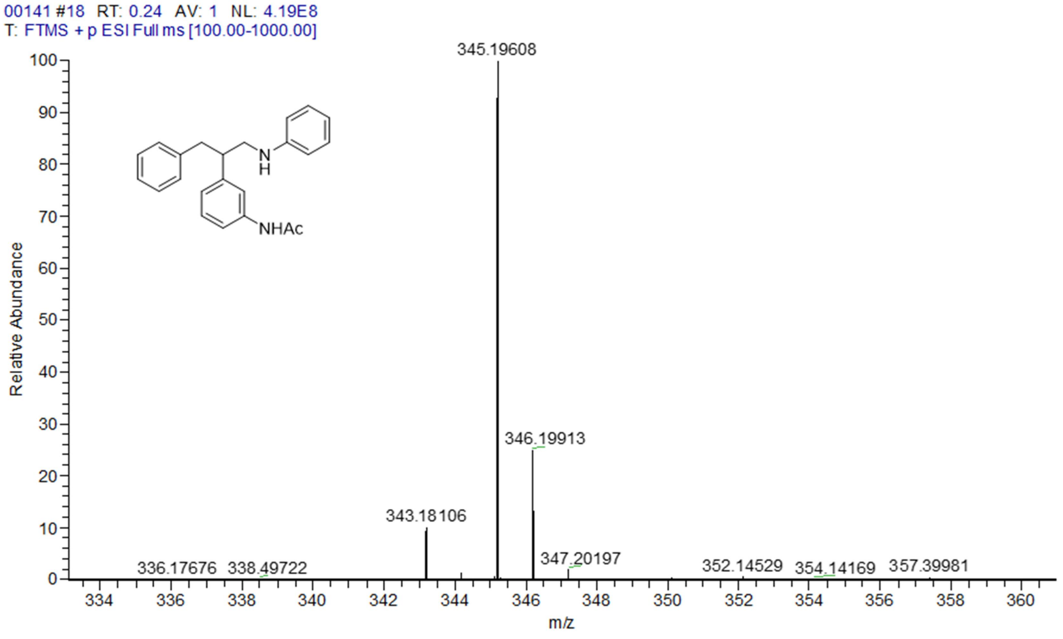

Supplementary Figure 120. HRMS of **3i**.

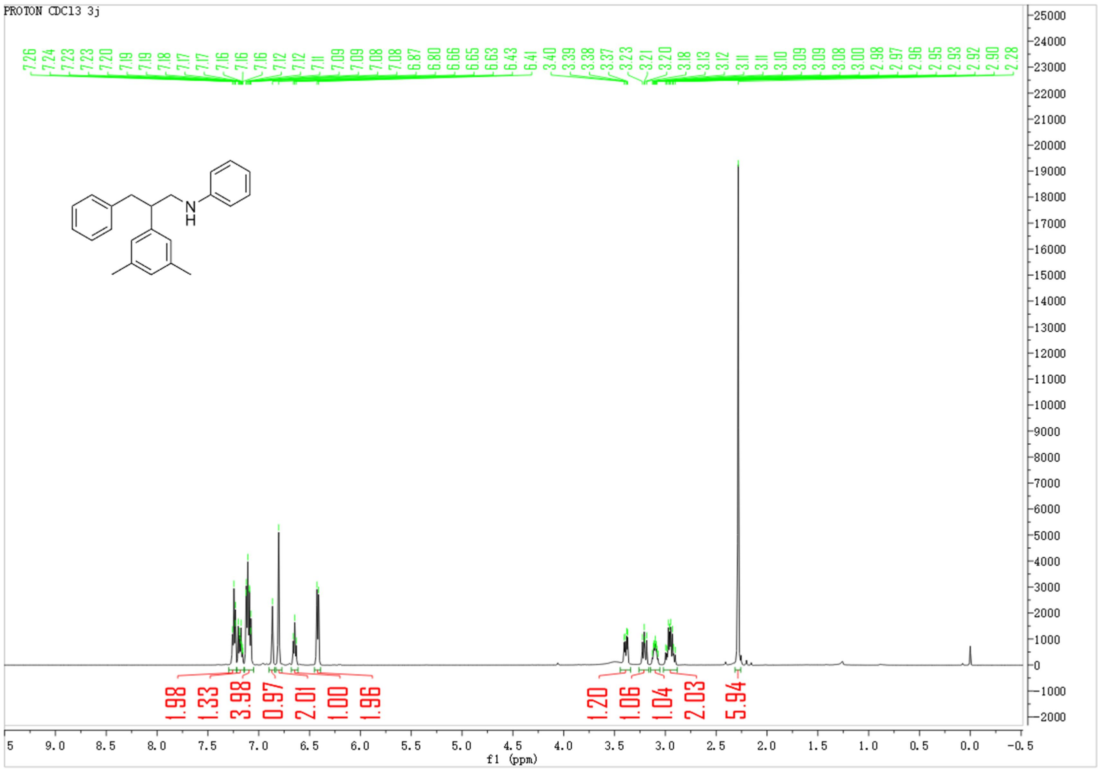

Supplementary Figure 121. ^{1}H NMR spectrum of **3j** in $CDCl_3$.

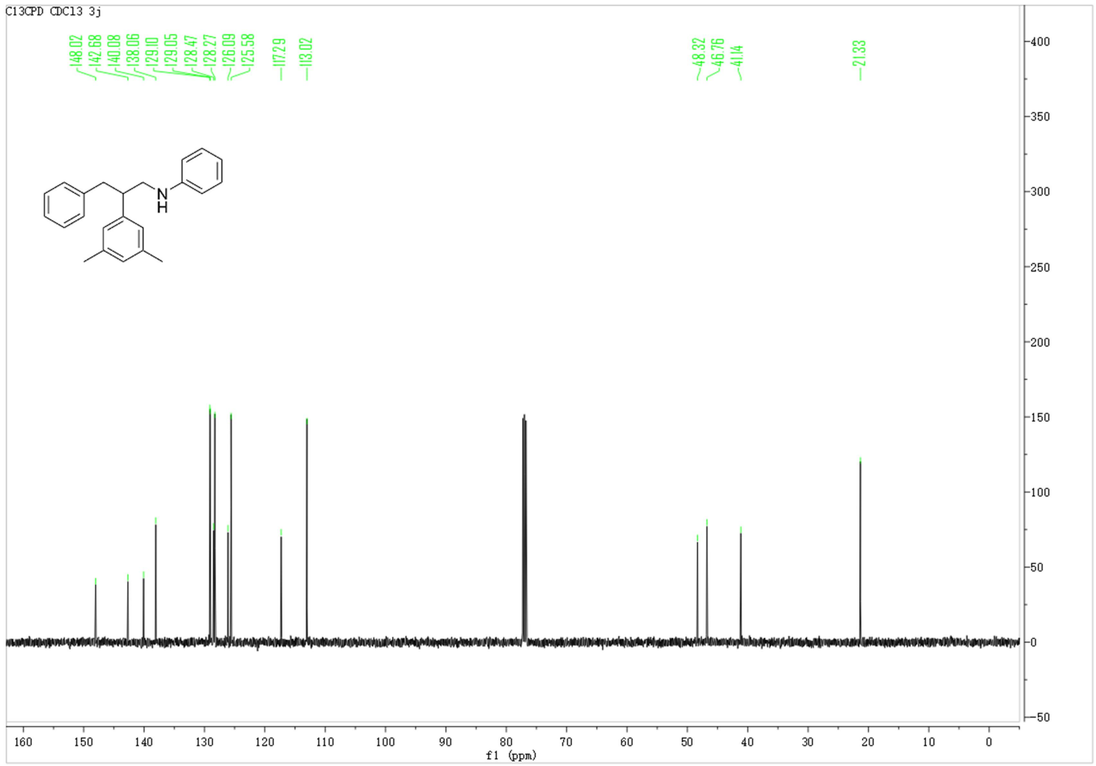

Supplementary Figure 122. ^{13}C NMR spectrum of **3j** in $CDCl_3$.

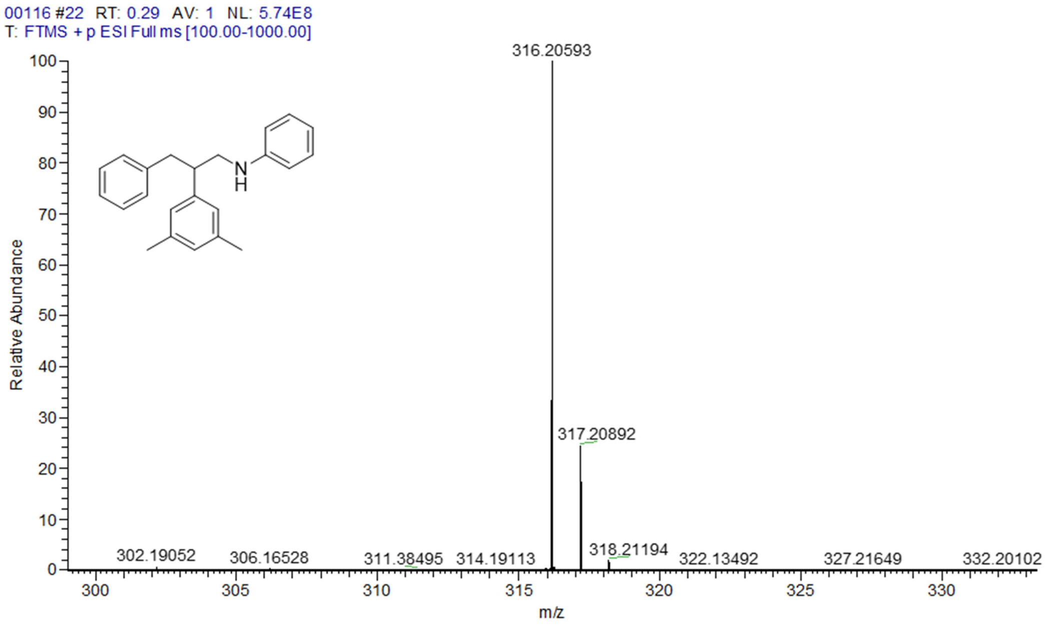

Supplementary Figure 123. HRMS of **3j**.

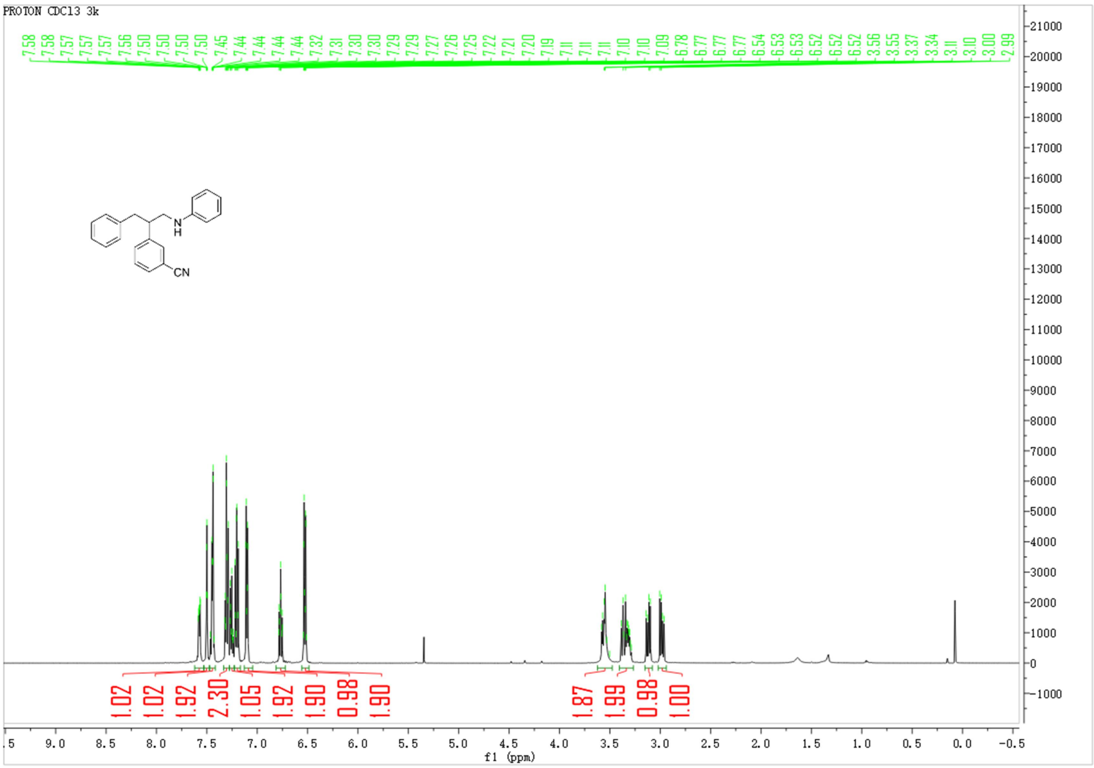

Supplementary Figure 124. ^{1}H NMR spectrum of **3k** in $CDCl_3$.

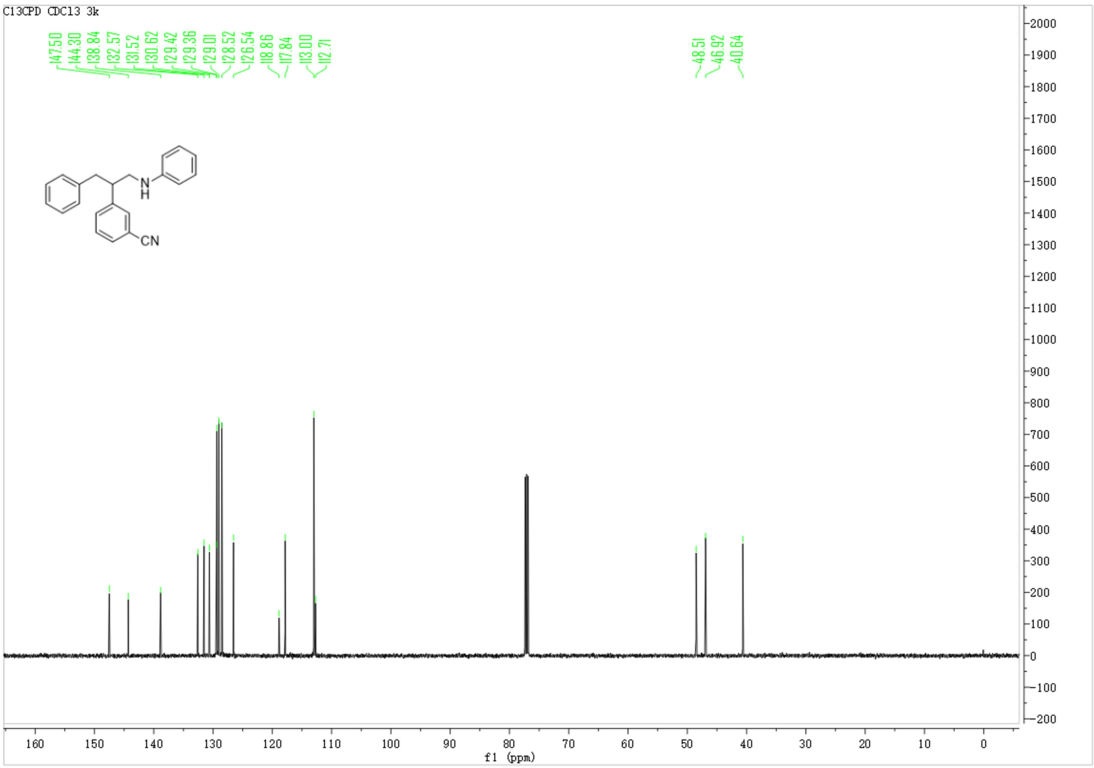

Supplementary Figure 125. ^{13}C NMR spectrum of **3k** in $CDCl_3$.

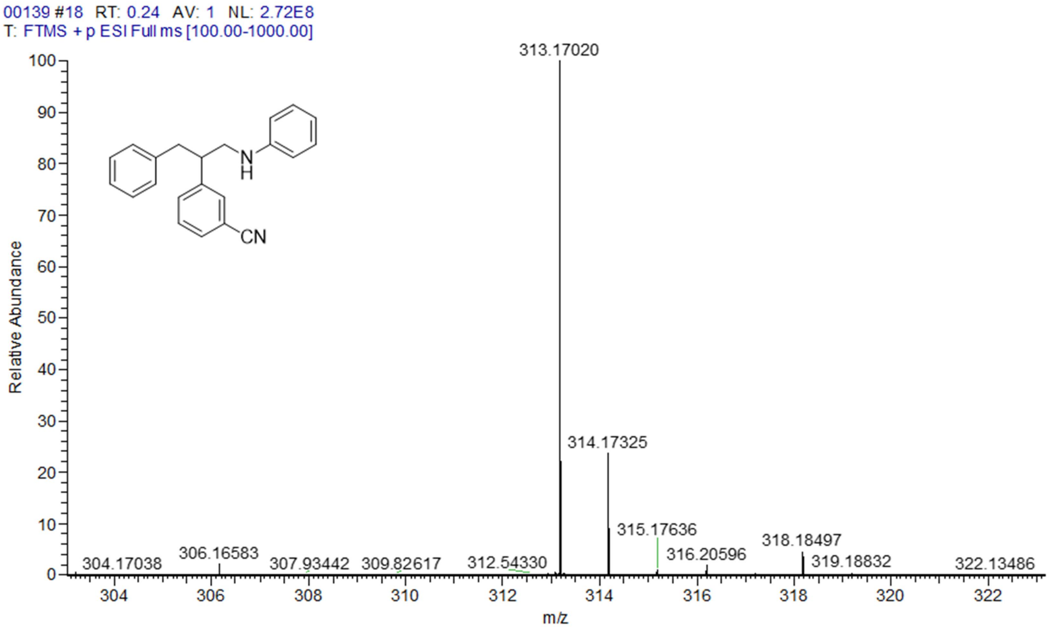

Supplementary Figure 126. HRMS of **3k**.

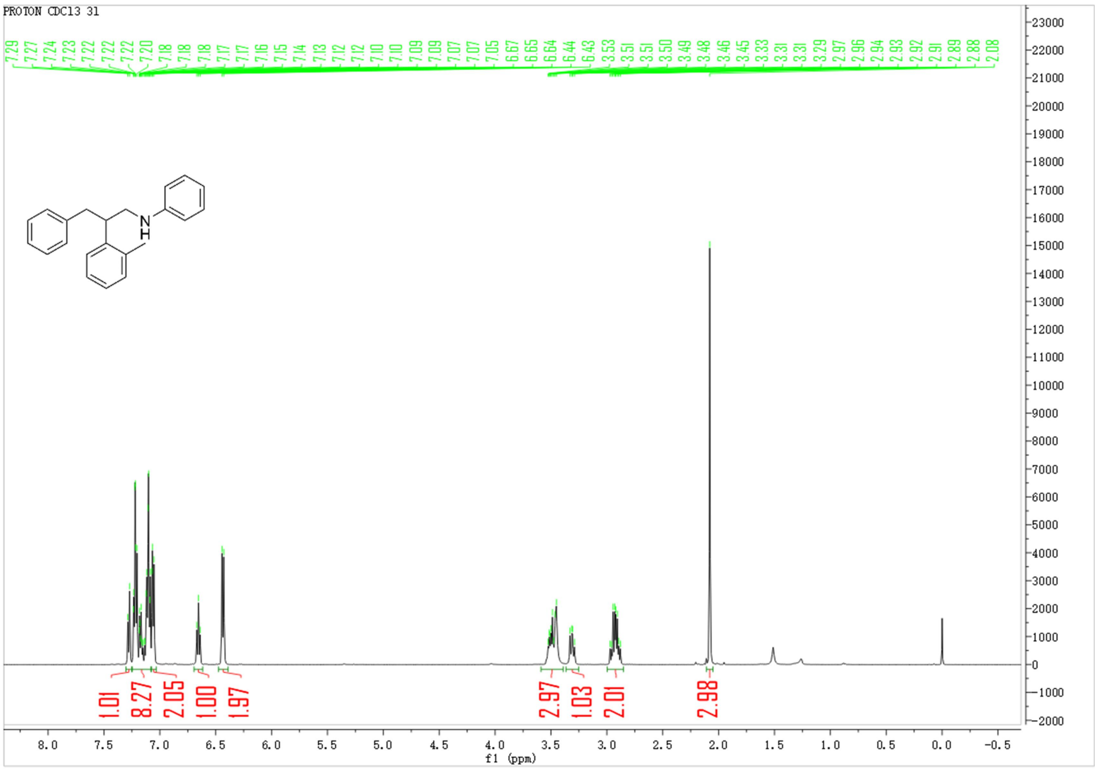

Supplementary Figure 127. ^{1}H NMR spectrum of **3l** in $CDCl_3$.

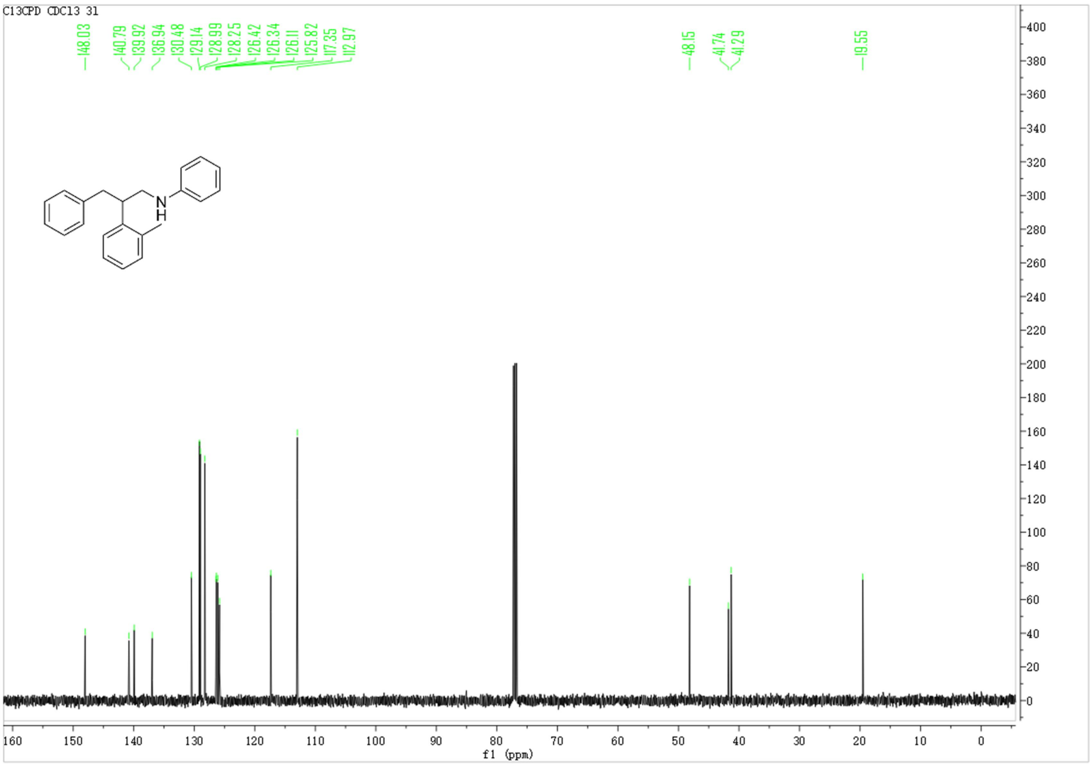

Supplementary Figure 128. ^{13}C NMR spectrum of **3l** in $CDCl_3$.

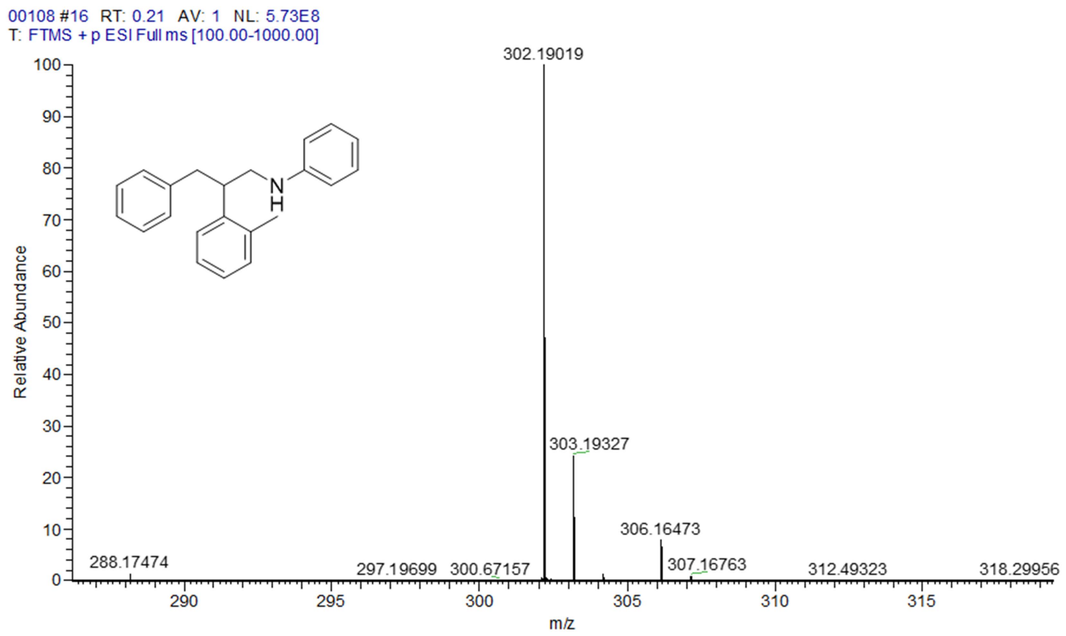

Supplementary Figure 129. HRMS of **3l**.

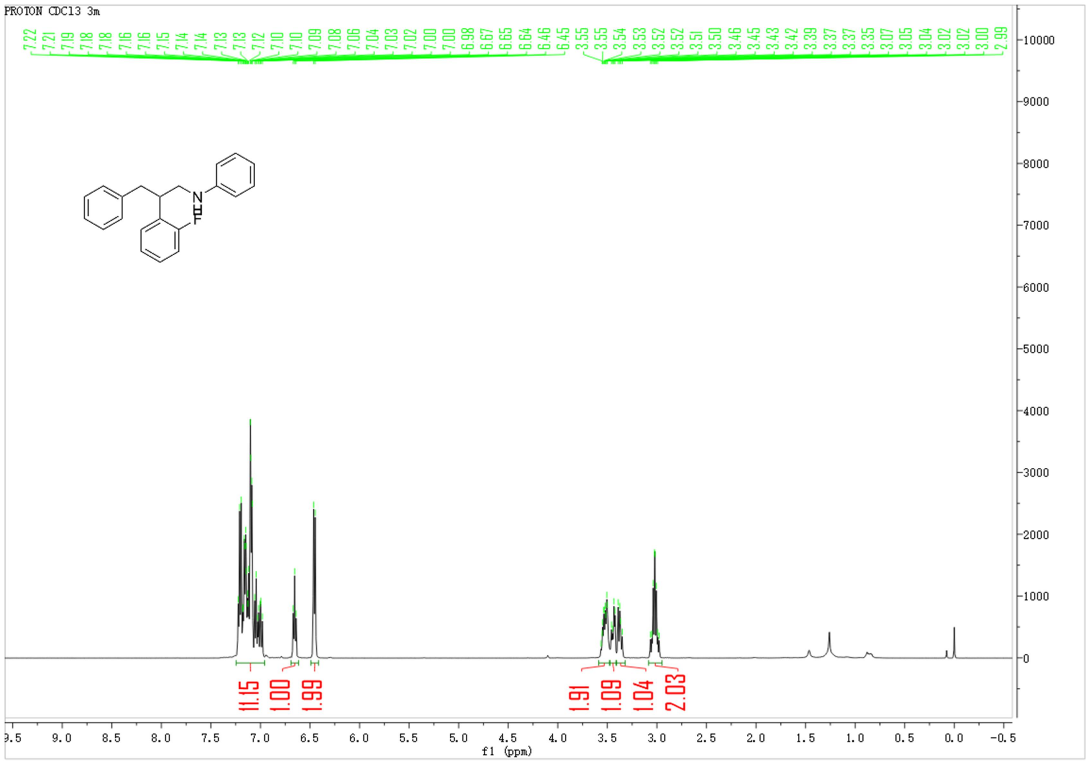

Supplementary Figure 130. ^{1}H NMR spectrum of **3m** in $CDCl_3$.

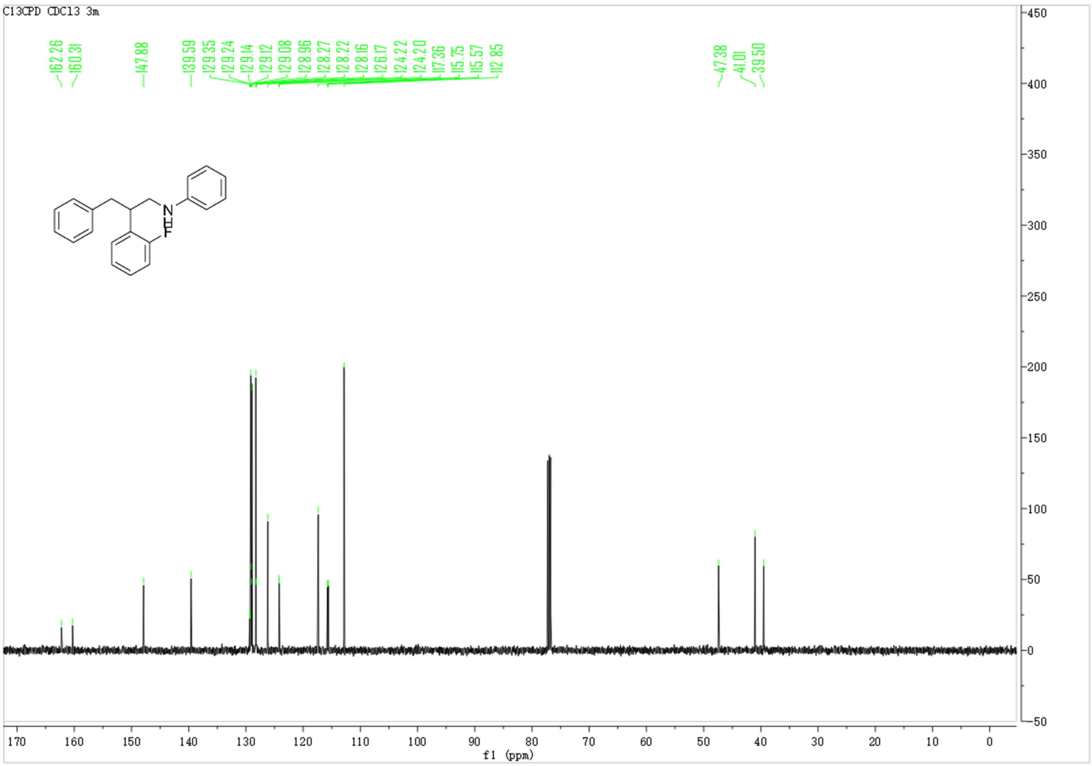

Supplementary Figure 131. ^{13}C NMR spectrum of **3m** in $CDCl_3$.

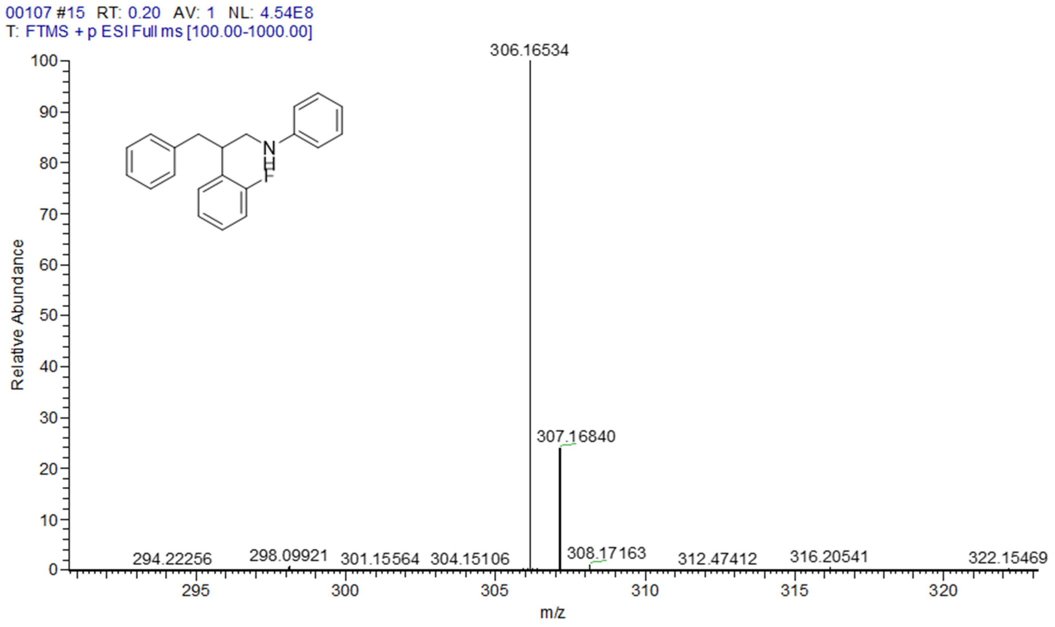

Supplementary Figure 132. HRMS of **3m**.

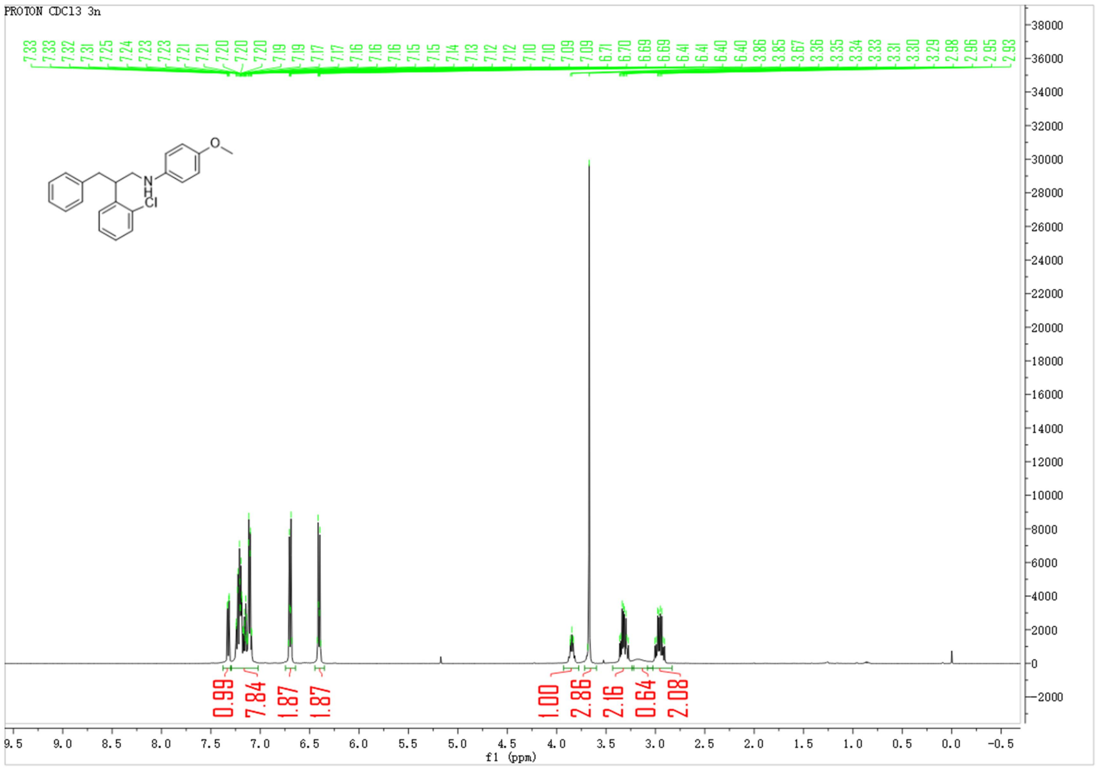

Supplementary Figure 133. ^{1}H NMR spectrum of **3n** in $CDCl_3$.

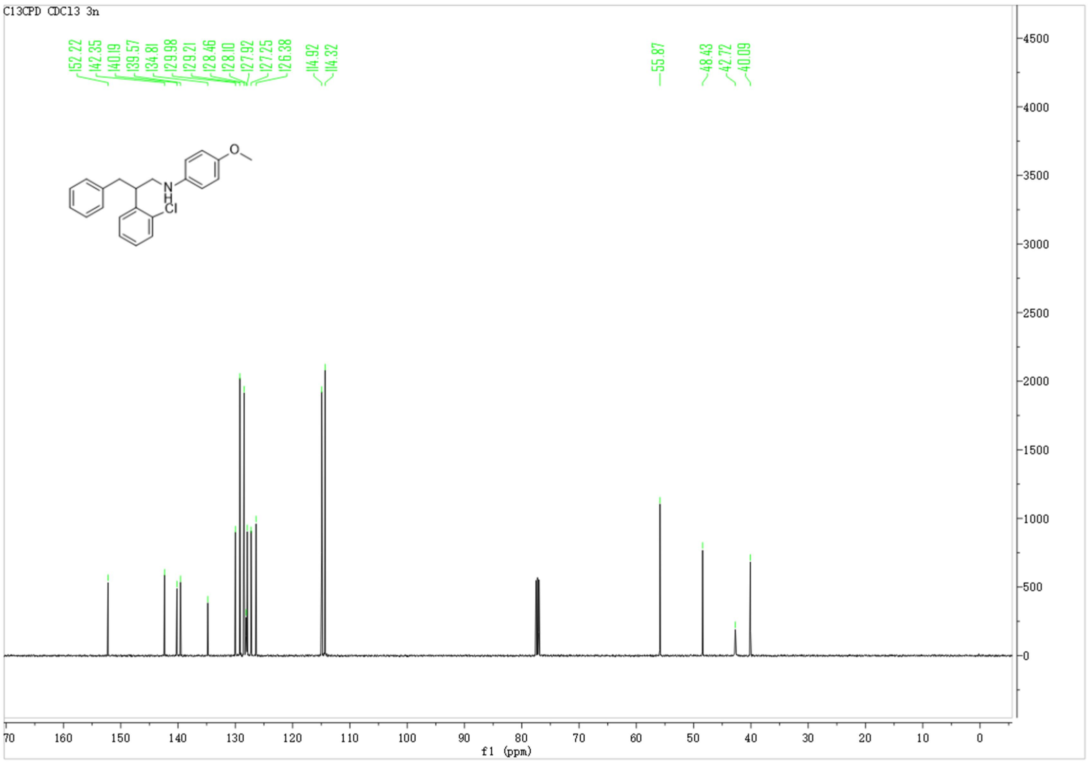

Supplementary Figure 134. ^{13}C NMR spectrum of **3n** in $CDCl_3$.

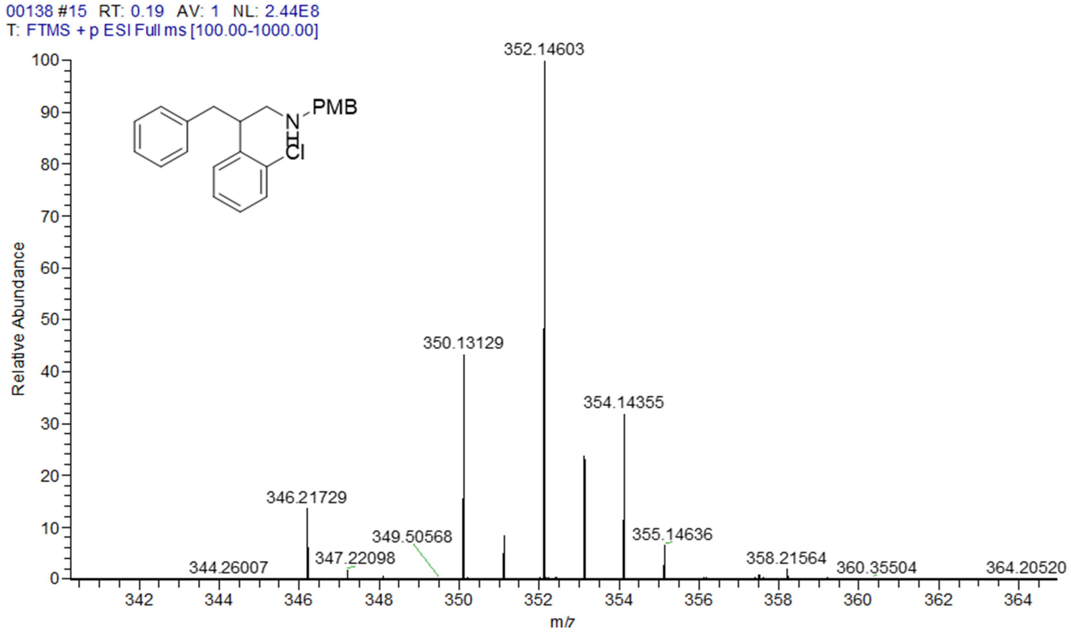

Supplementary Figure 135. HRMS of **3n**.

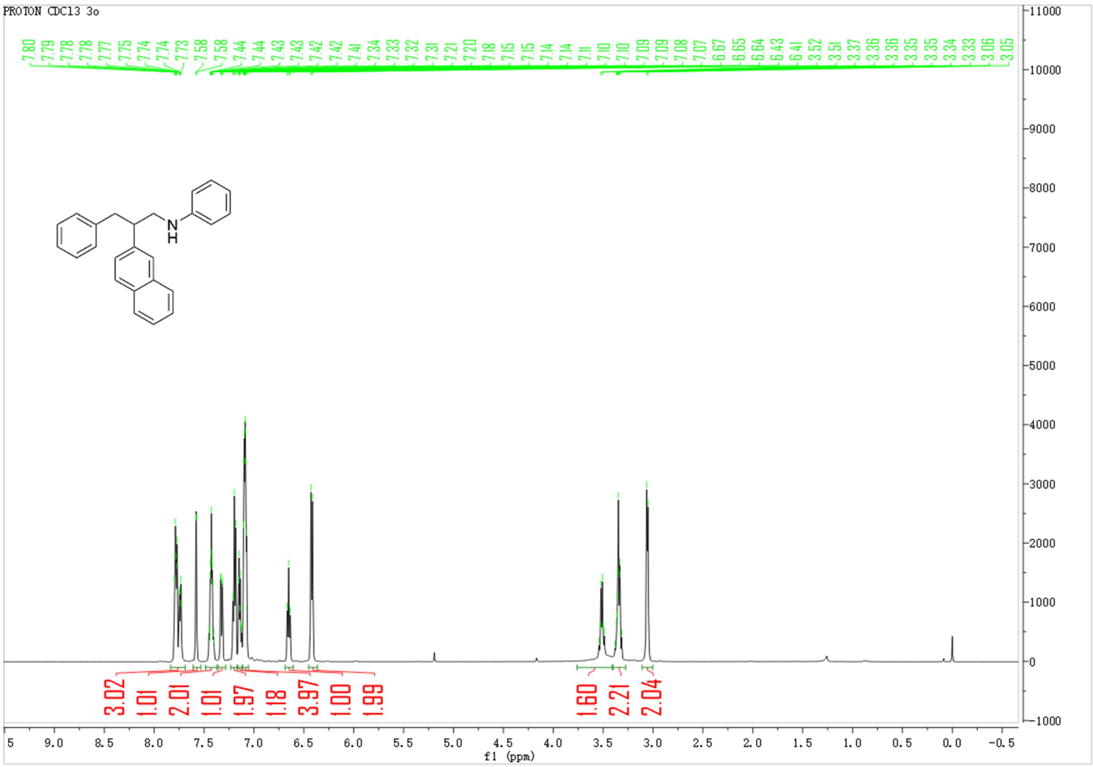

Supplementary Figure 136. ^{1}H NMR spectrum of **3o** in $CDCl_3$.

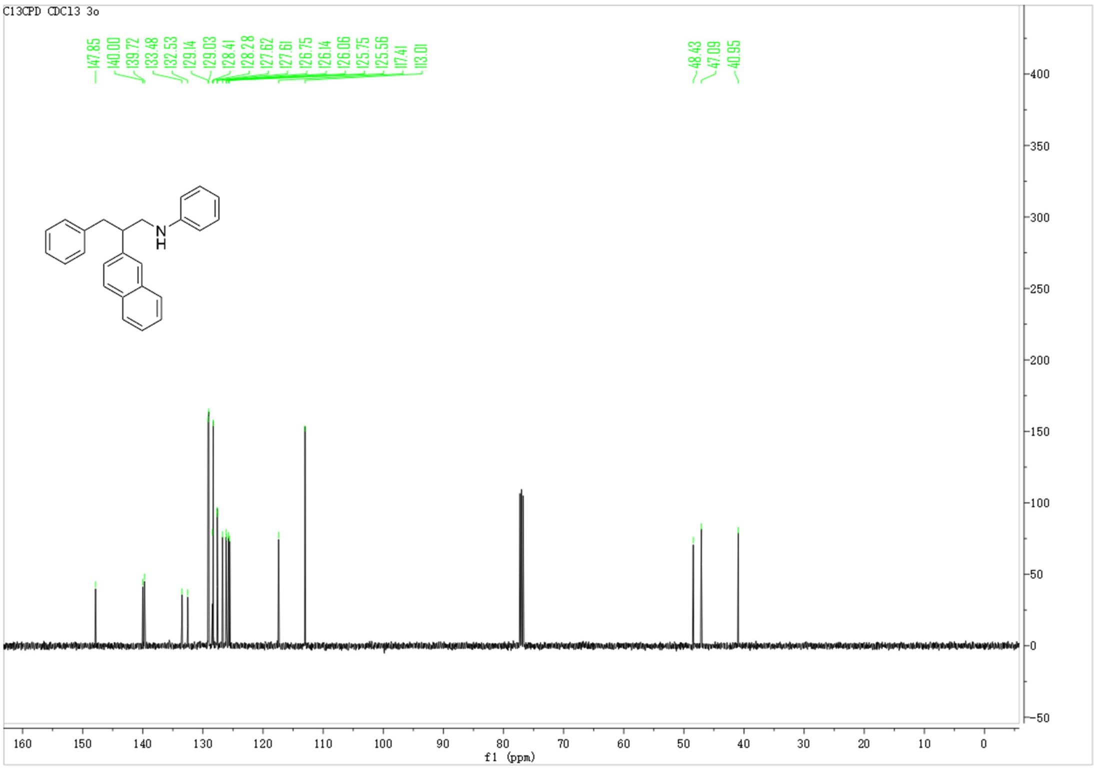

Supplementary Figure 137. ^{13}C NMR spectrum of **3o** in $CDCl_3$.

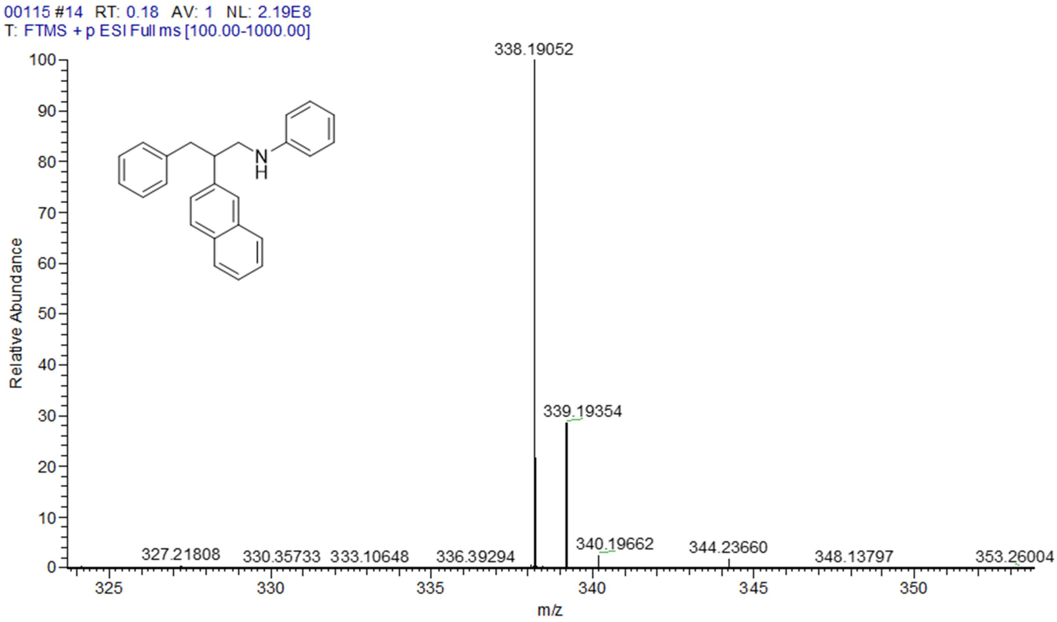

Supplementary Figure 138. HRMS of **1o**.

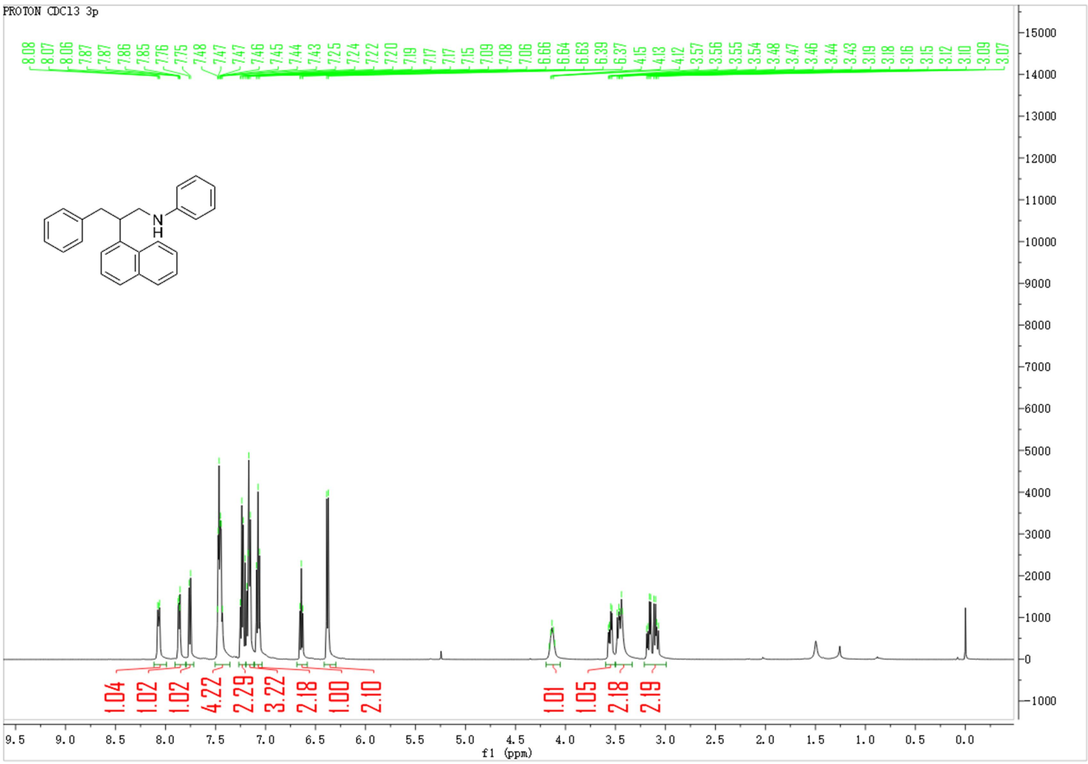

Supplementary Figure 139. ^{1}H NMR spectrum of **3p** in $CDCl_3$.

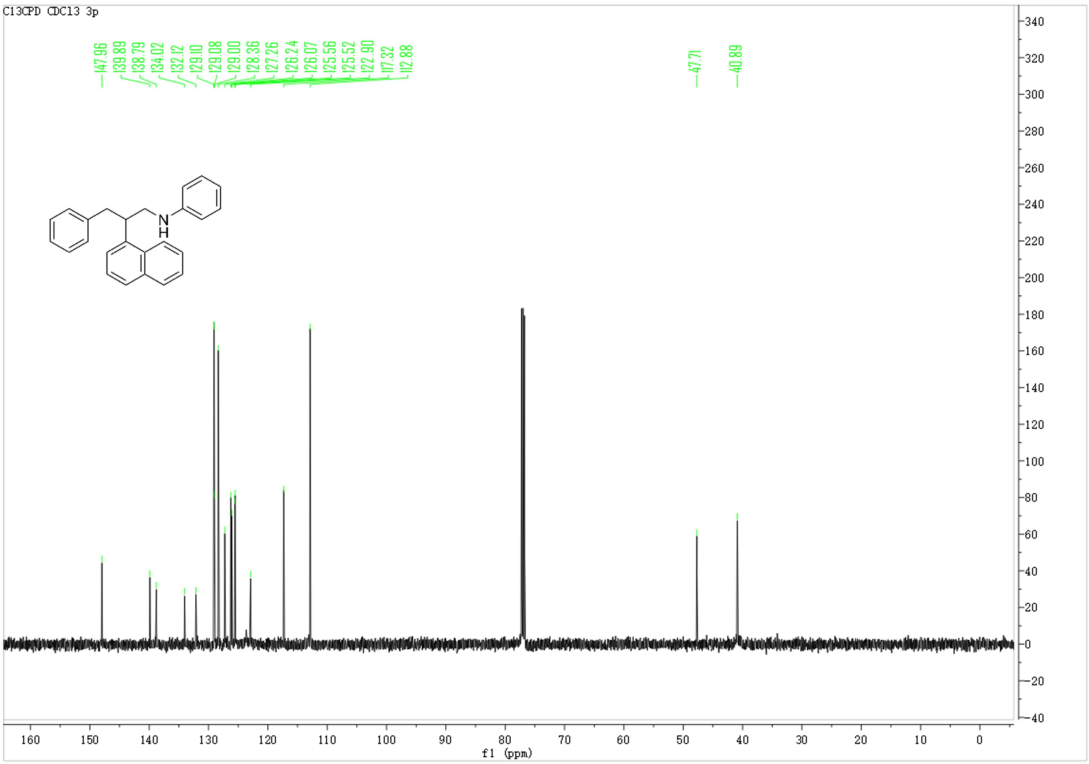

Supplementary Figure 140. ^{13}C NMR spectrum of **3p** in $CDCl_3$.

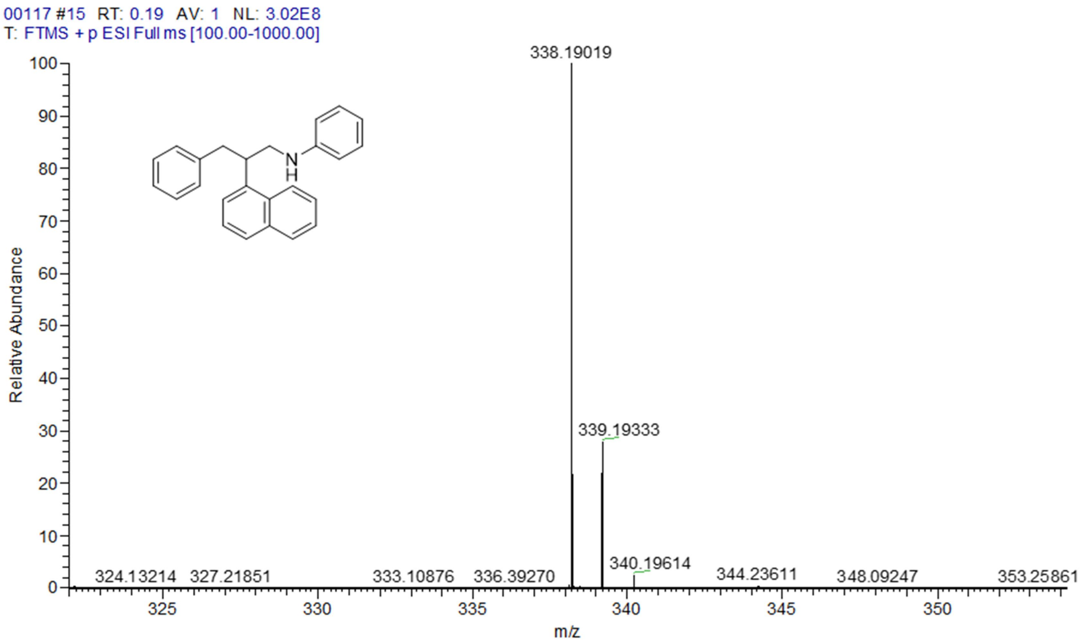

Supplementary Figure 141. HRMS of **3p**.

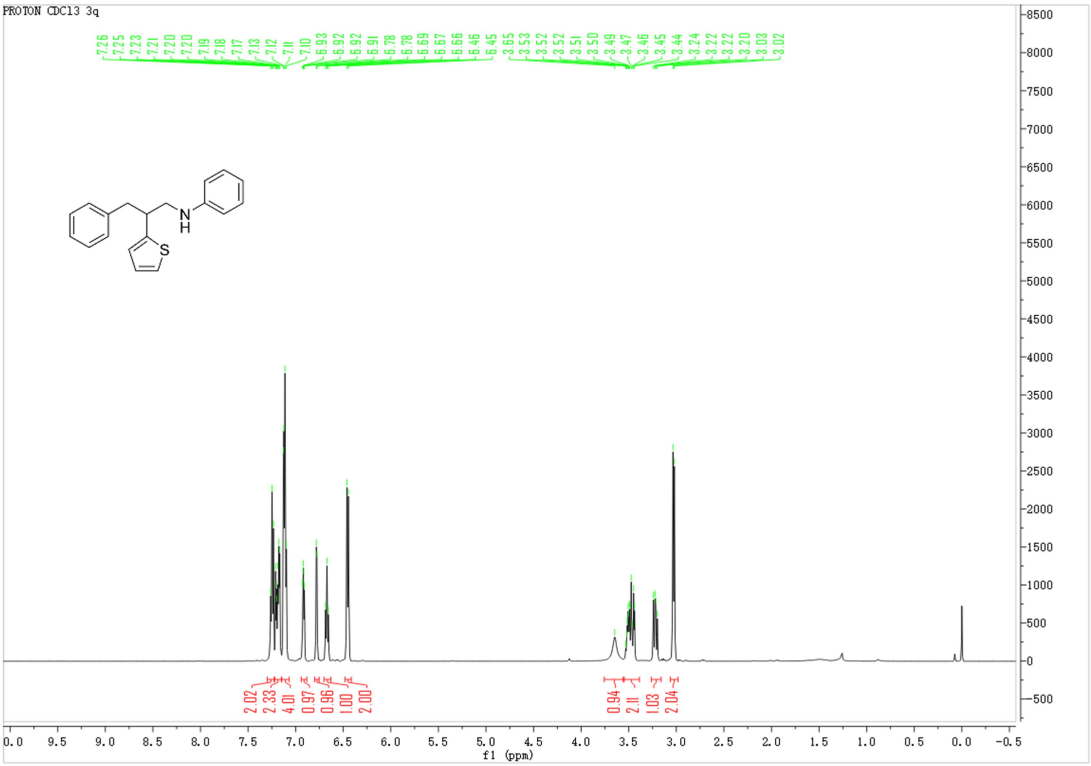

Supplementary Figure 142. ^{1}H NMR spectrum of **3q** in $CDCl_3$.

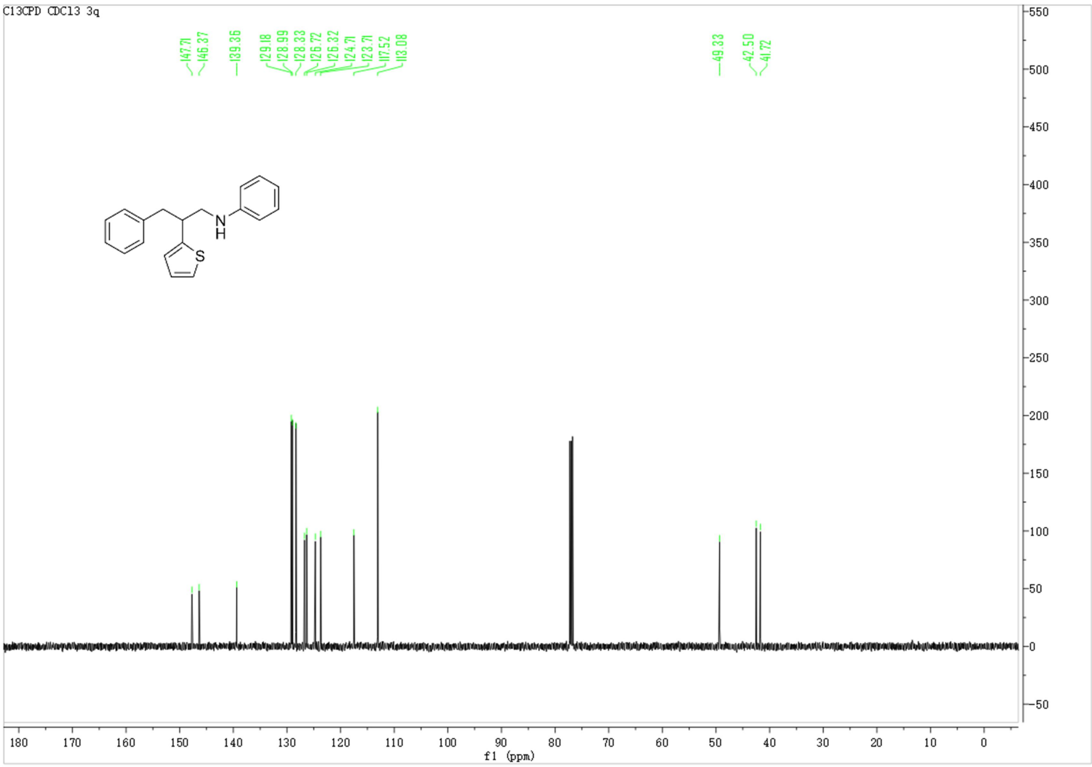

Supplementary Figure 143. ^{13}C NMR spectrum of **3q** in $CDCl_3$.

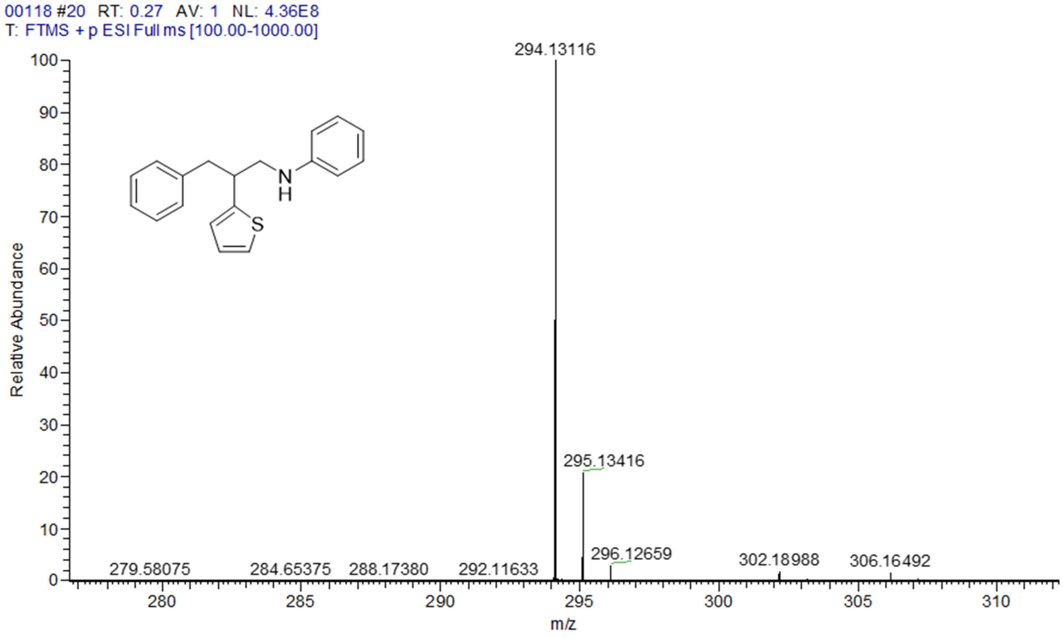

Supplementary Figure 144. HRMS of **3q**.

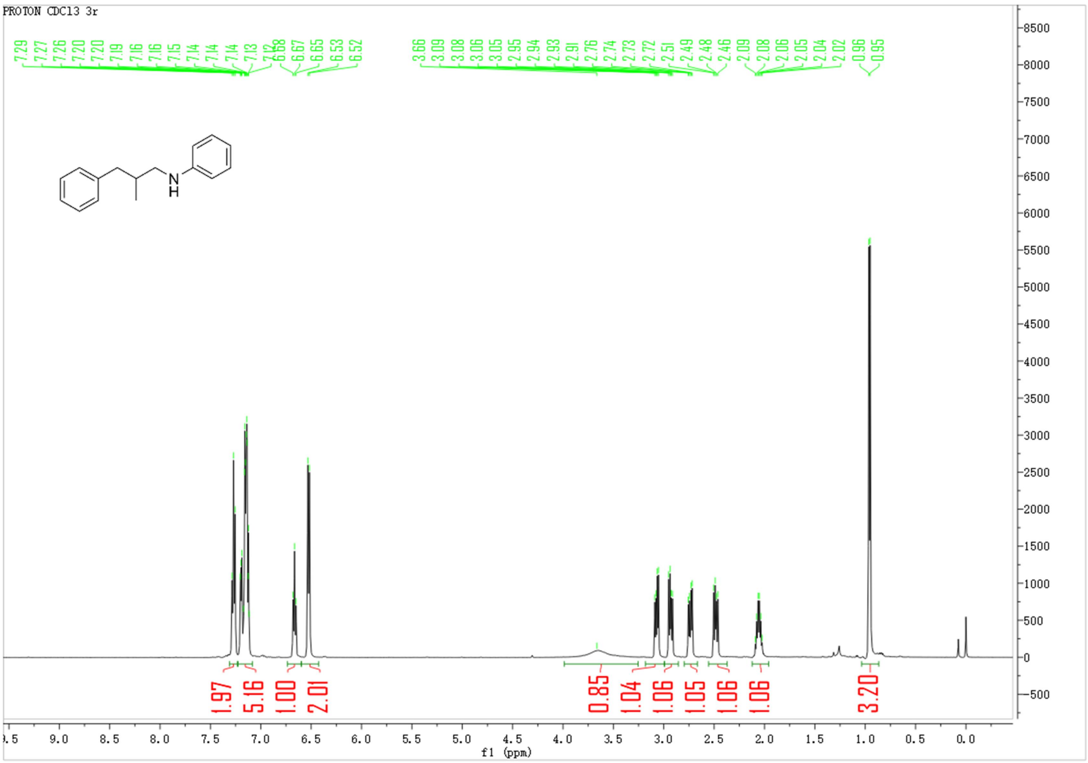

Supplementary Figure 145. ^{1}H NMR spectrum of **3r** in $CDCl_3$.

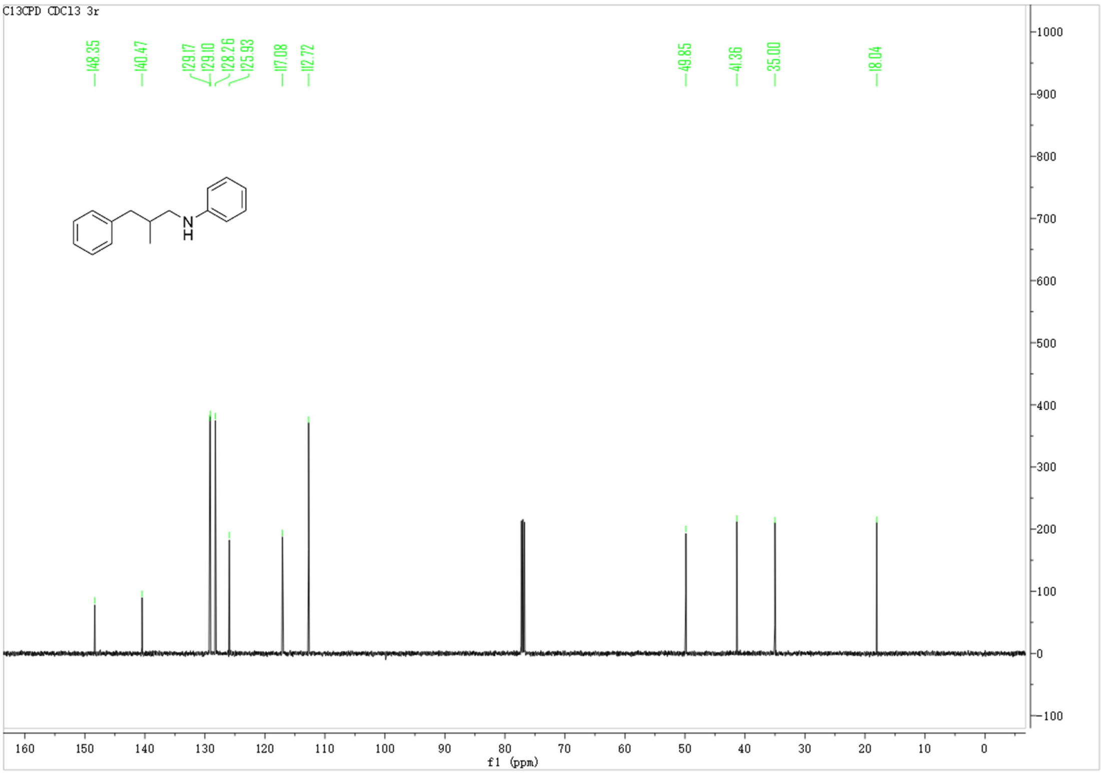

Supplementary Figure 146. ^{13}C NMR spectrum of **3r** in $CDCl_3$.

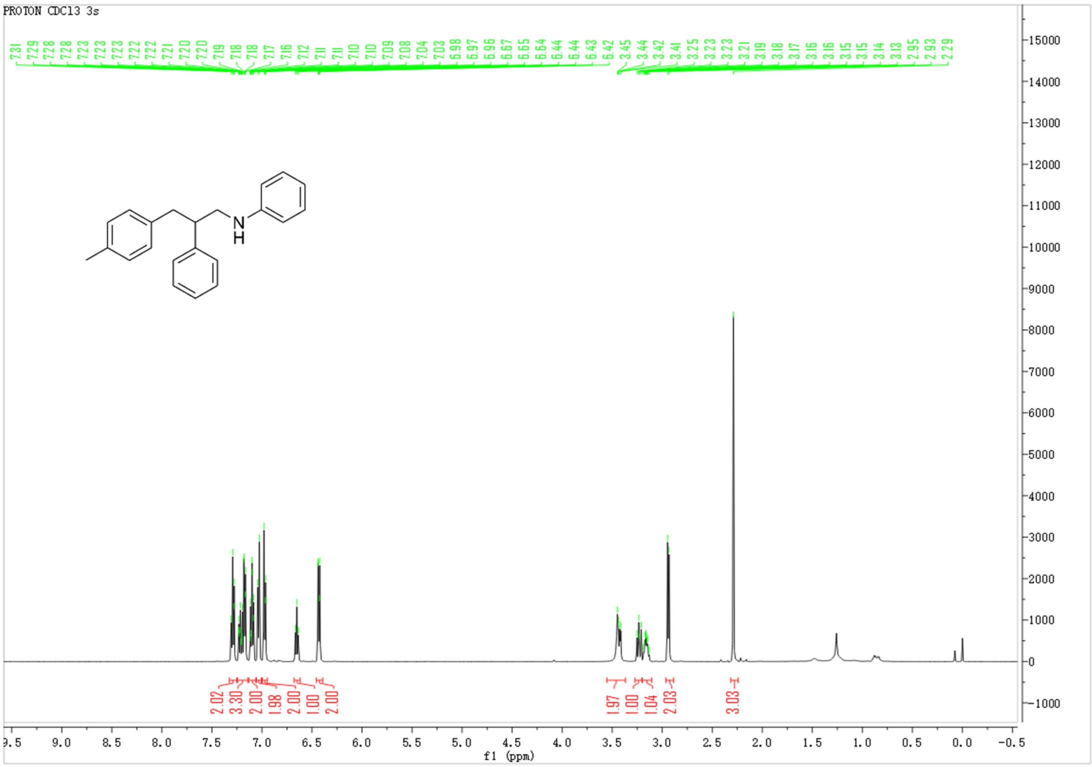

Supplementary Figure 147. ^{1}H NMR spectrum of **3s** in $CDCl_3$.

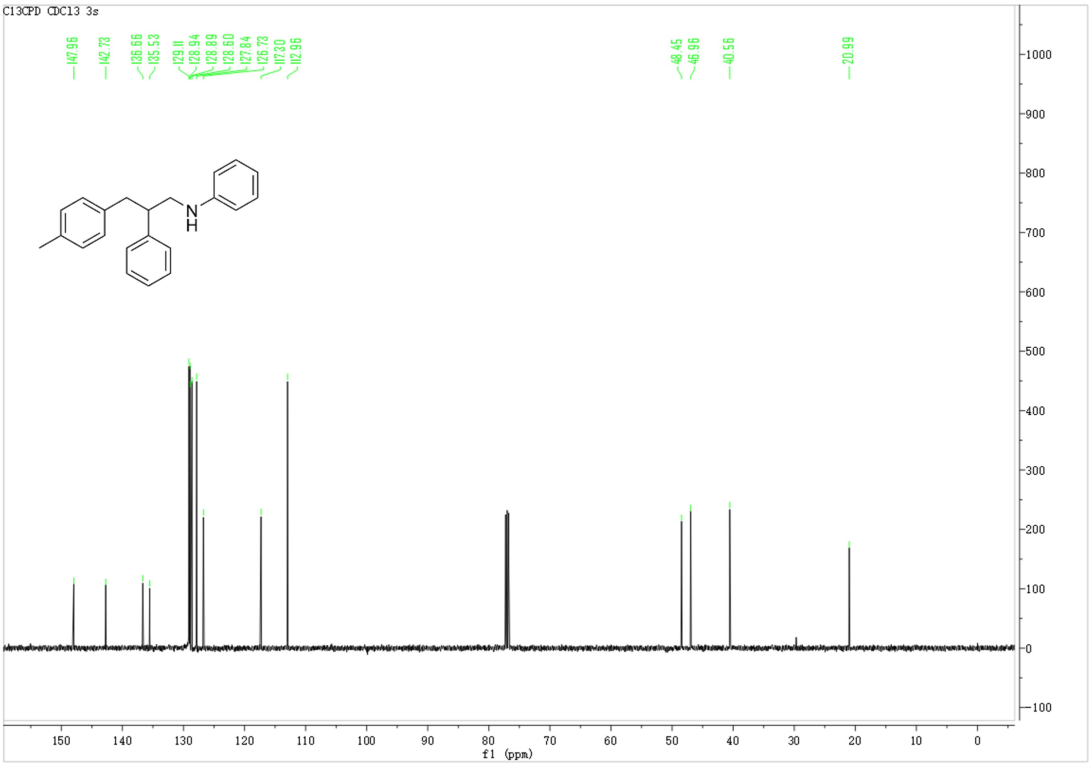

Supplementary Figure 148. ^{13}C NMR spectrum of **3s** in $CDCl_3$.

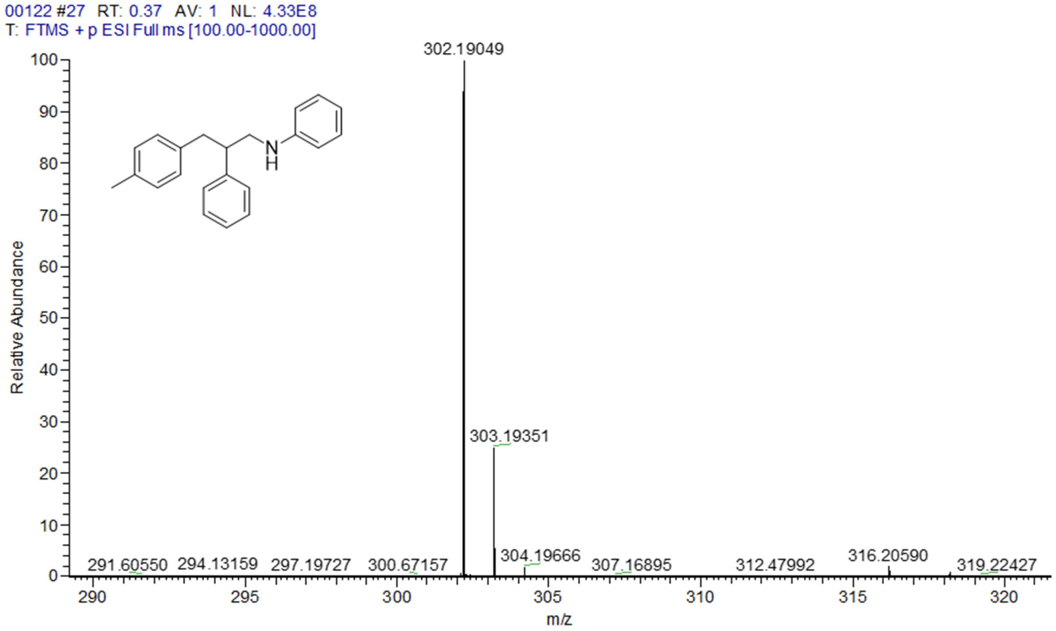

Supplementary Figure 149. HRMS of **3s**.

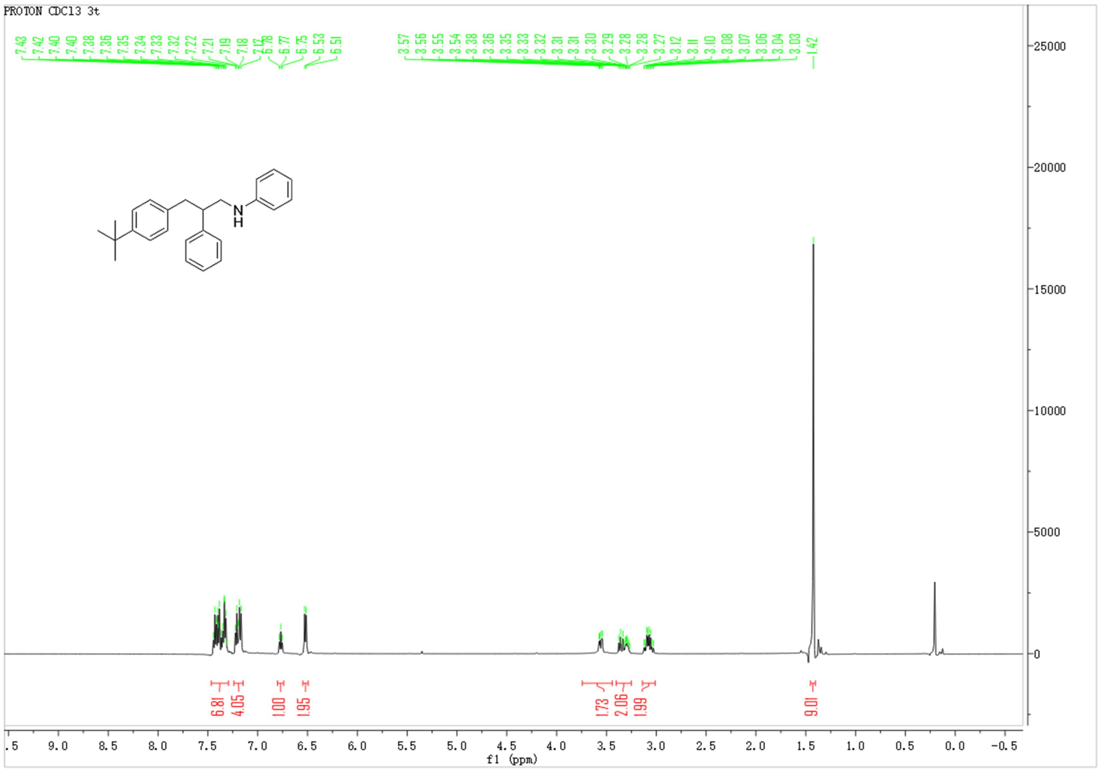

Supplementary Figure 150. ^{1}H NMR spectrum of **3t** in $CDCl_3$.

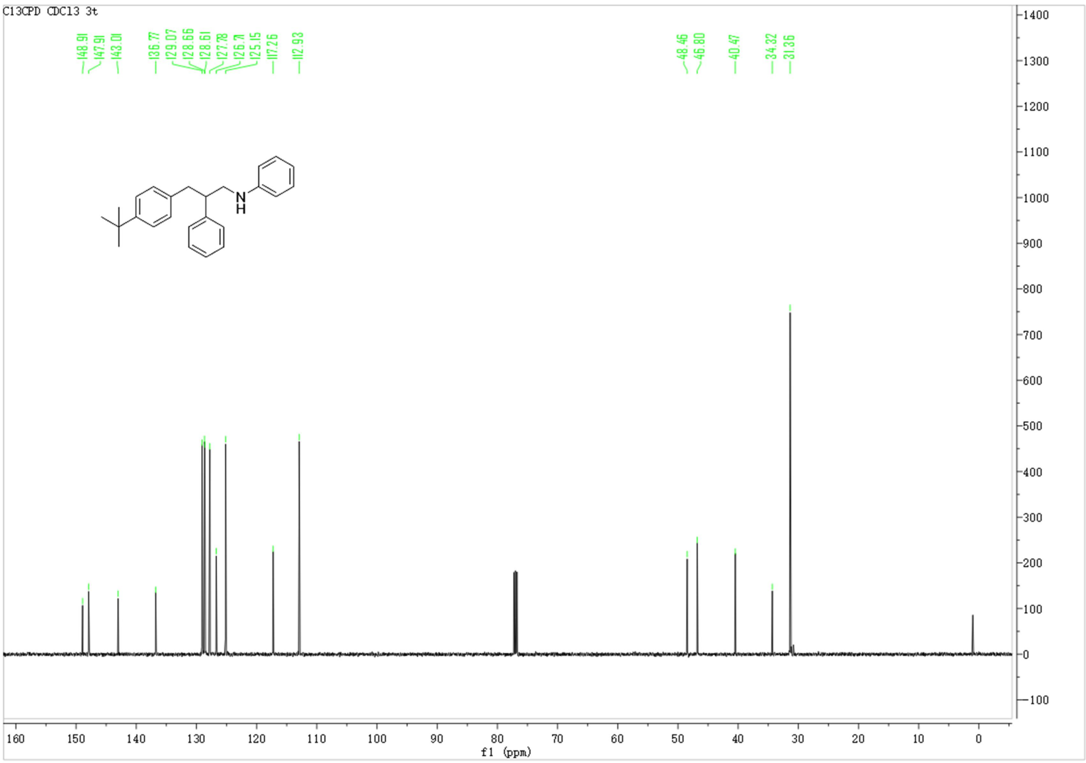

Supplementary Figure 151. ^{13}C NMR spectrum of **3t** in $CDCl_3$.

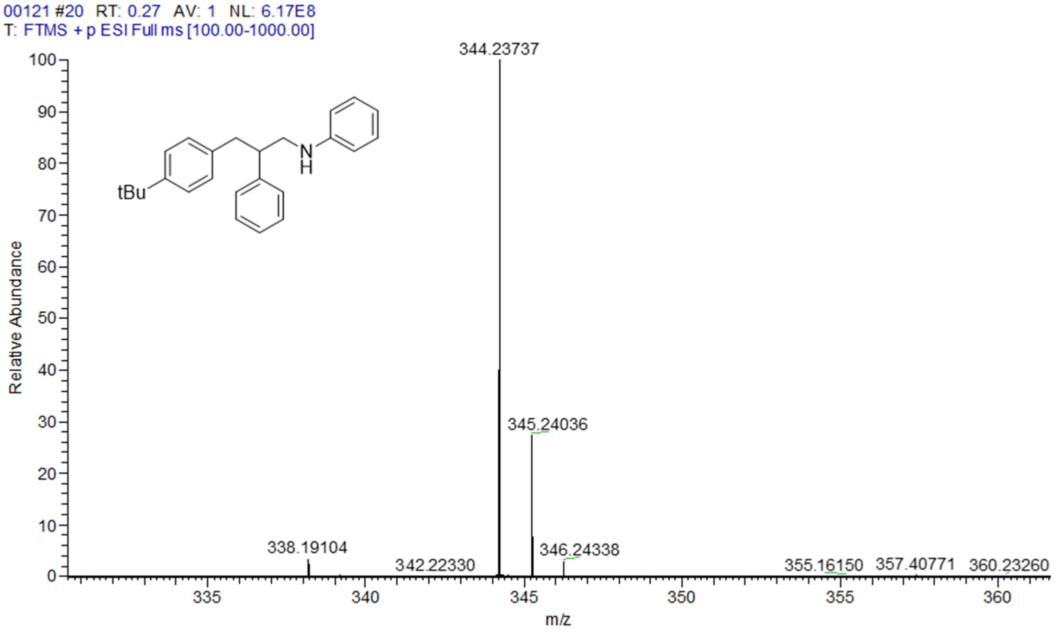

Supplementary Figure 152. HRMS of **3t**.

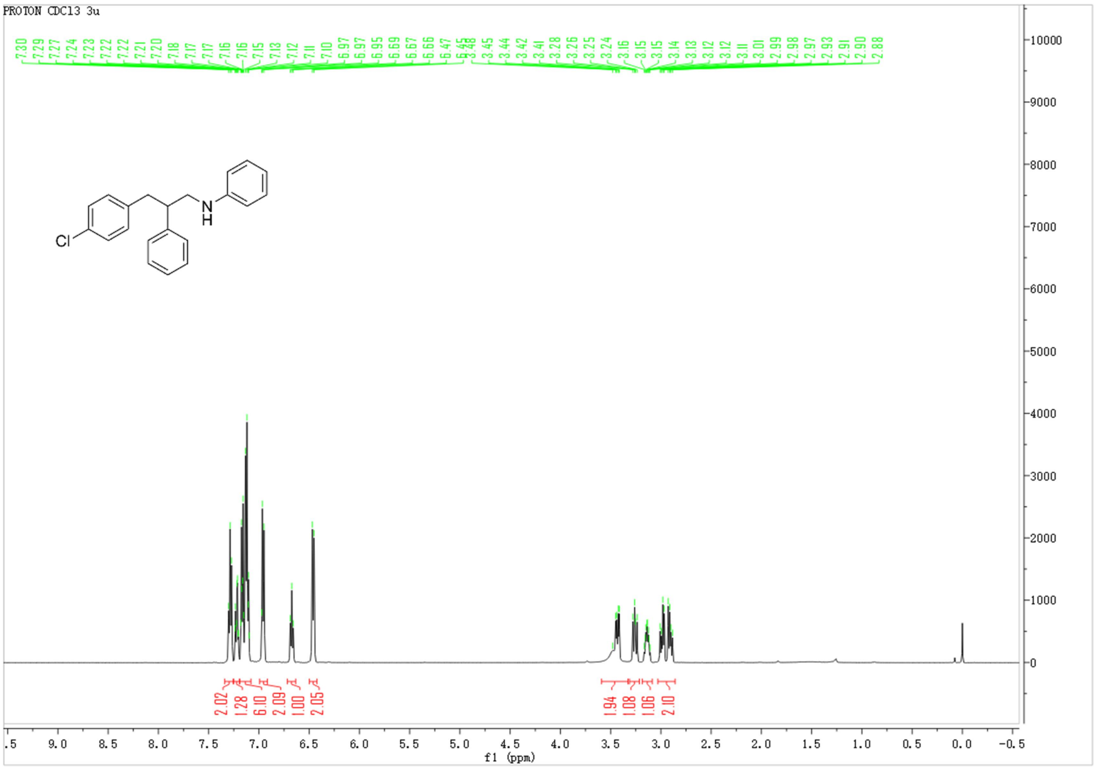

Supplementary Figure 153. ^{1}H NMR spectrum of **3u** in $CDCl_3$.

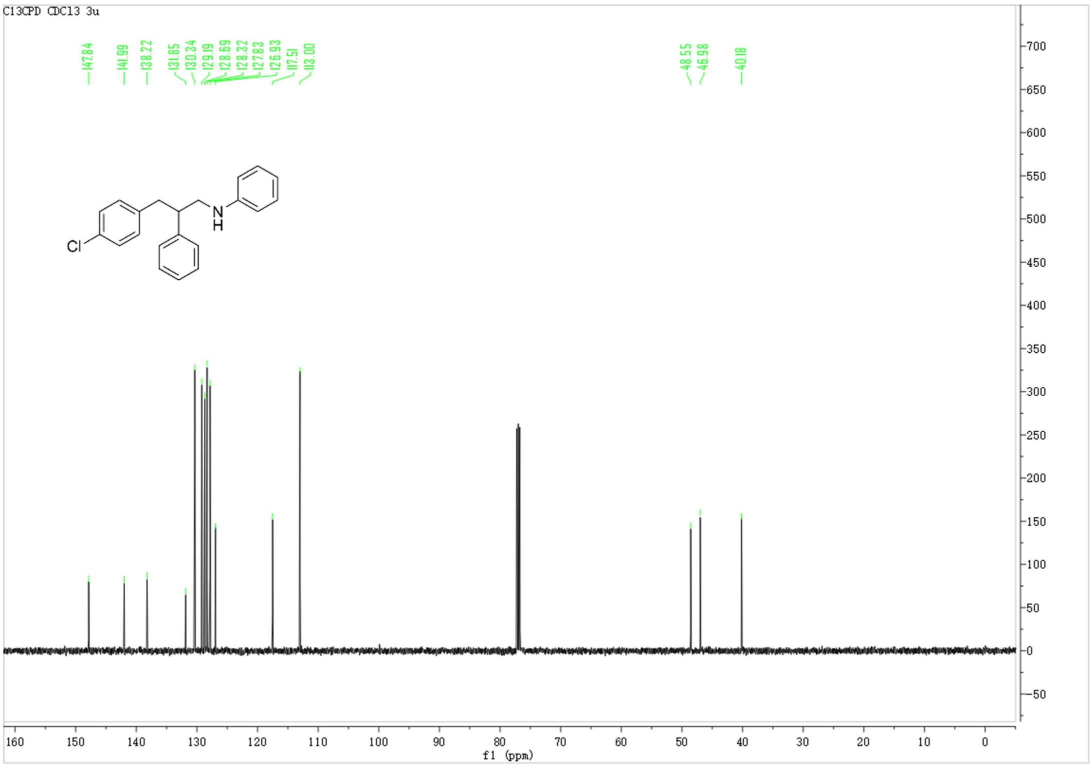

Supplementary Figure 154. ^{13}C NMR spectrum of **3u** in $CDCl_3$.

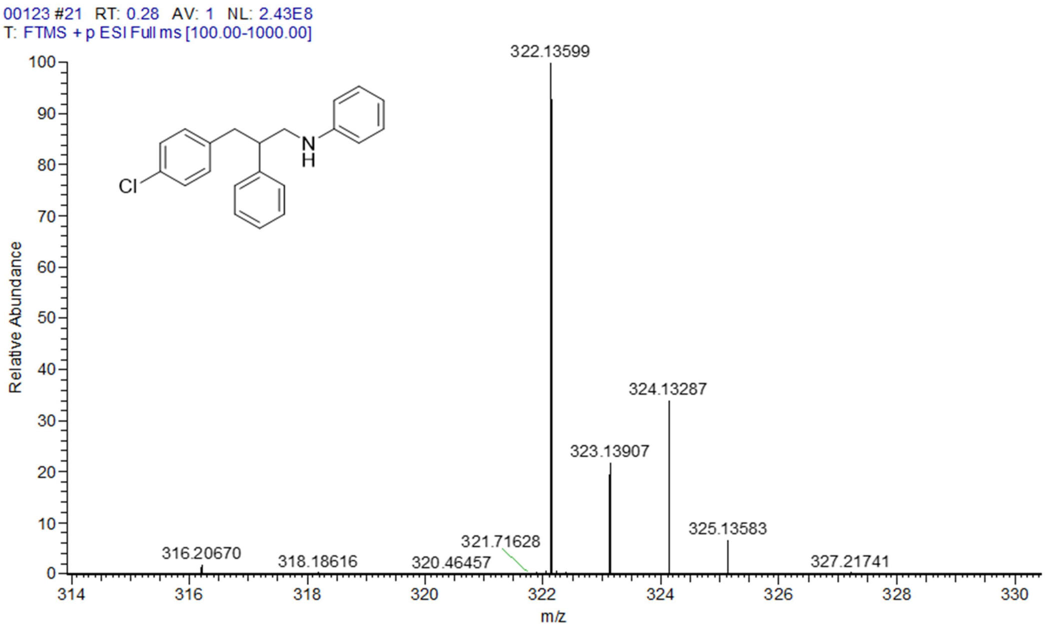

Supplementary Figure 155. HRMS of **3u**.

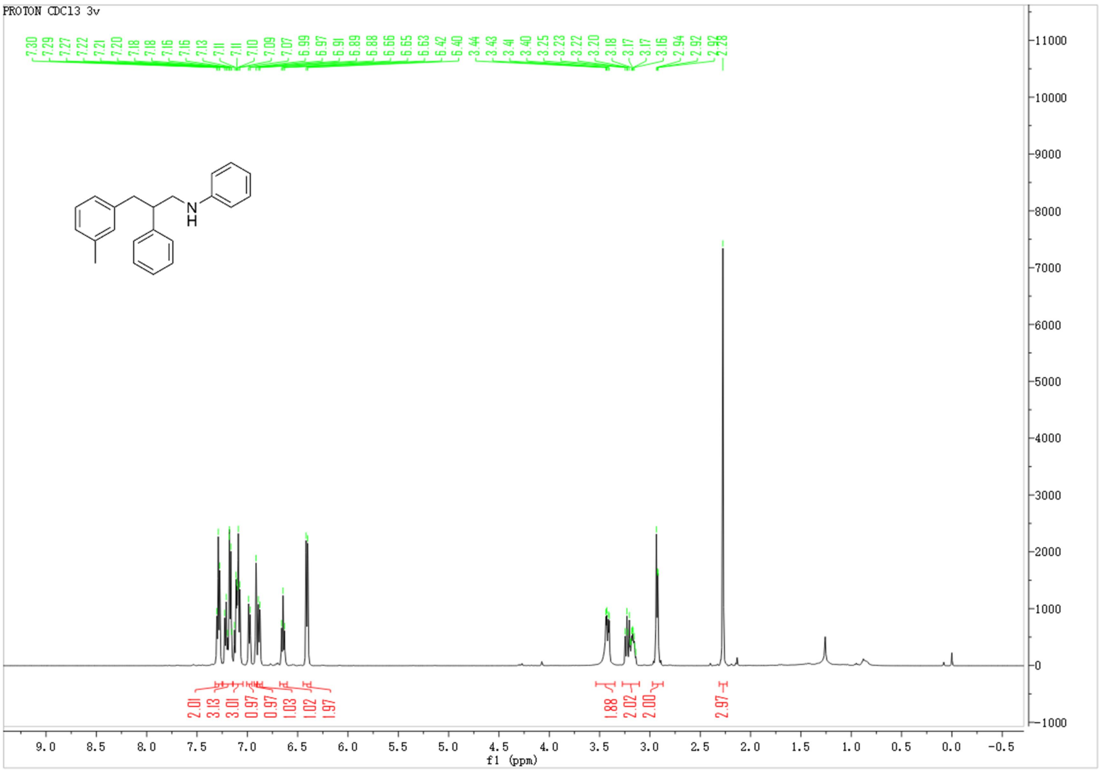

Supplementary Figure 156. ^{1}H NMR spectrum of **3v** in $CDCl_3$.

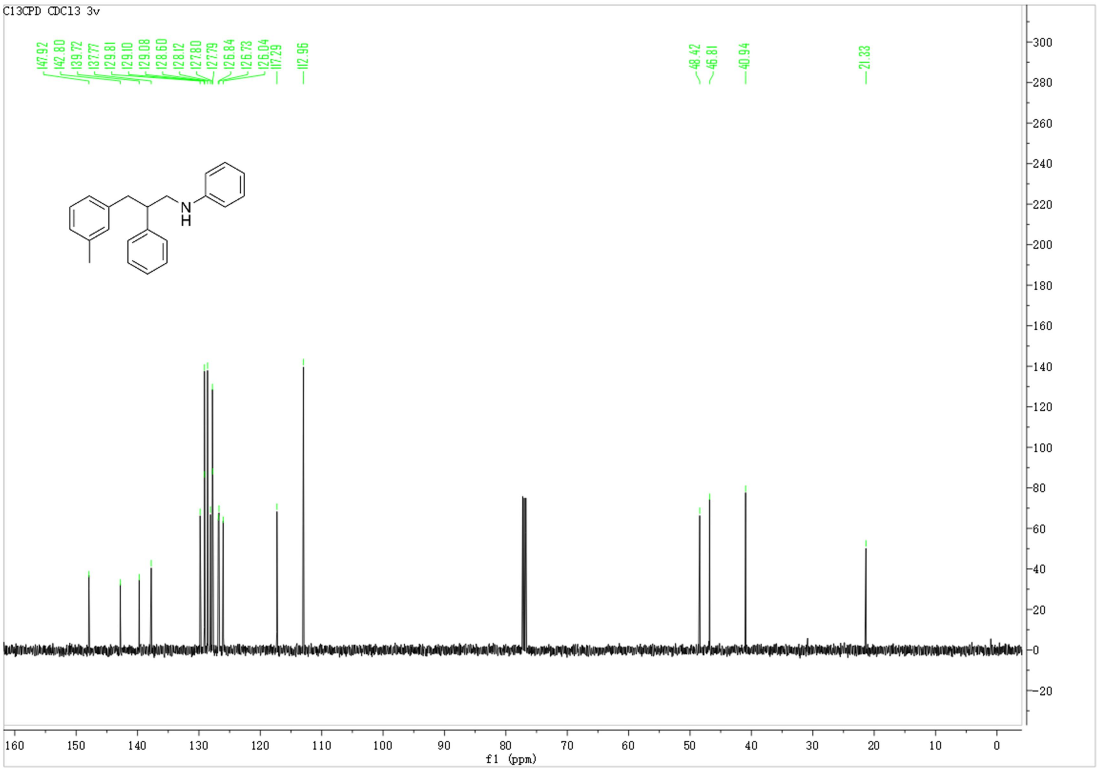

Supplementary Figure 157. ^{13}C NMR spectrum of **3v** in $CDCl_3$.

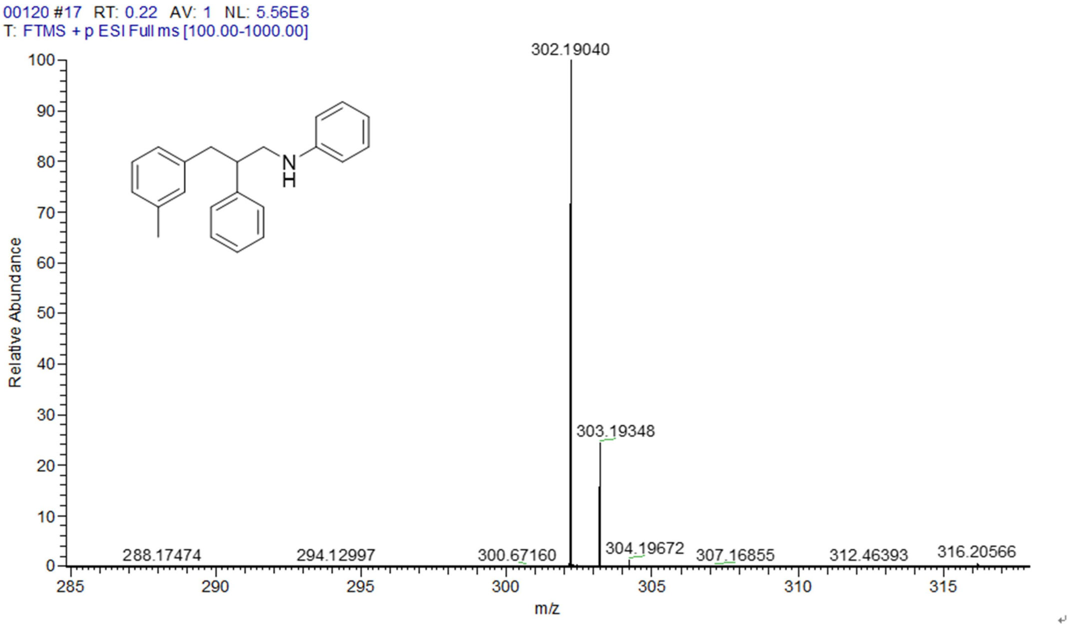

Supplementary Figure 158. HRMS of **3v**.

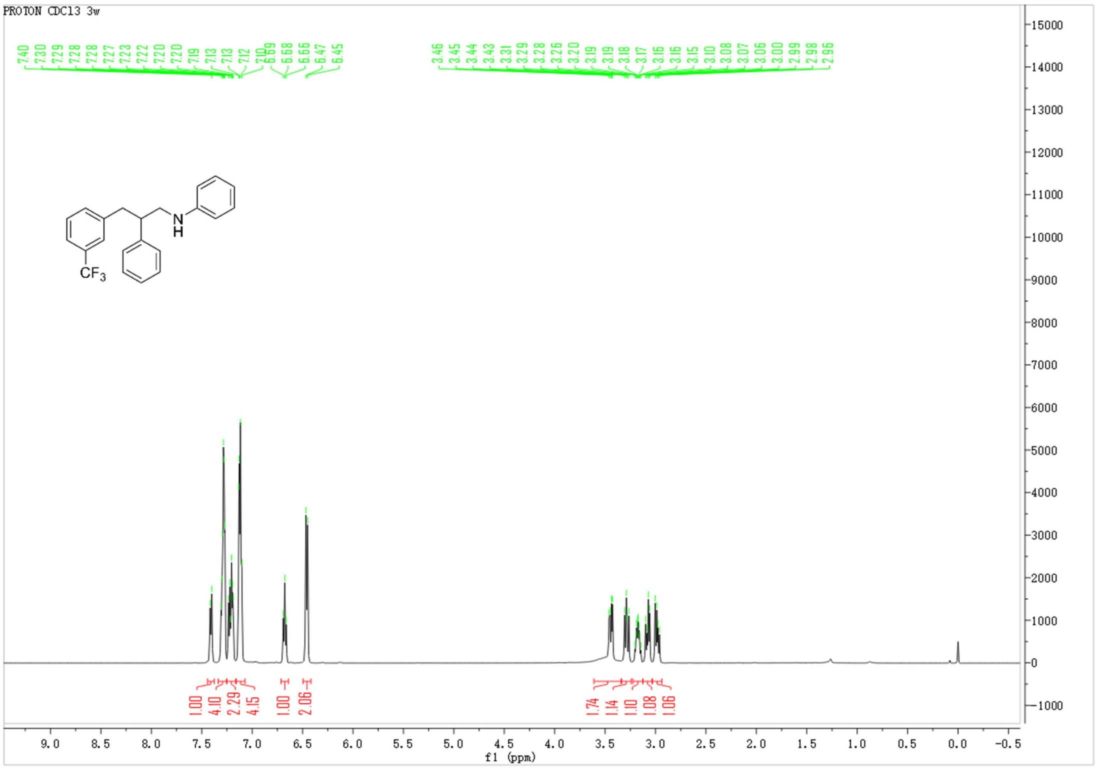

Supplementary Figure 159. ^{1}H NMR spectrum of **3w** in $CDCl_3$.

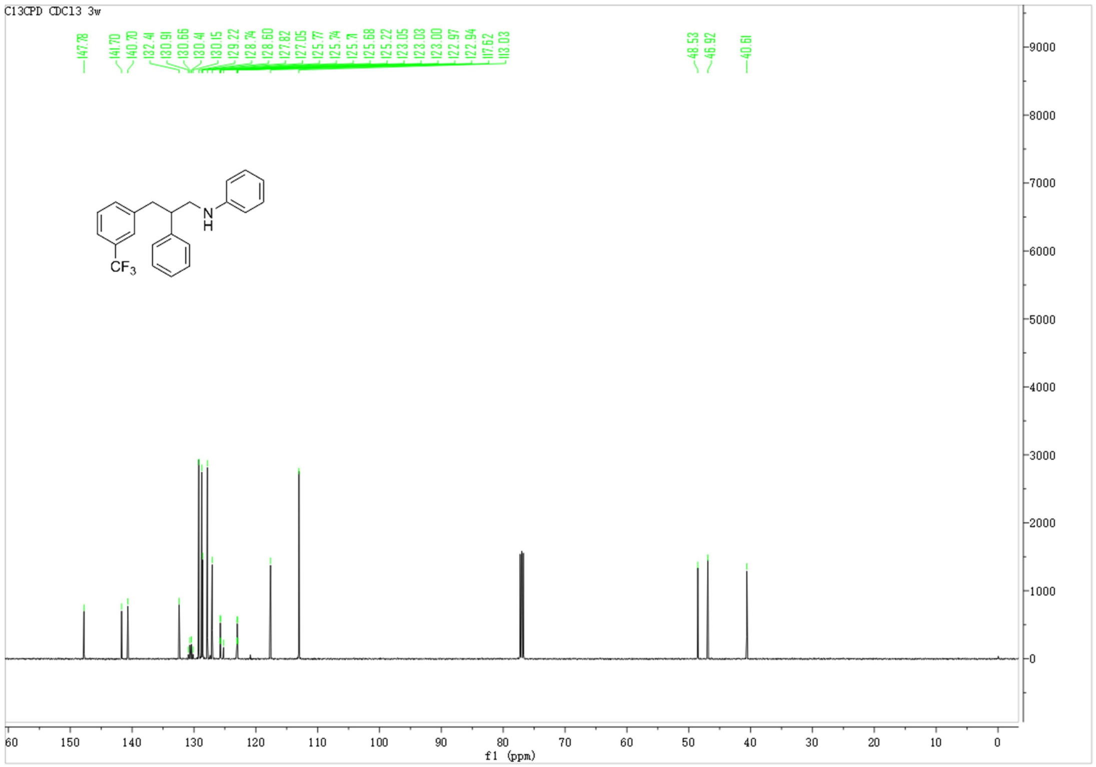

Supplementary Figure 160. ^{13}C NMR spectrum of **3w** in $CDCl_3$.

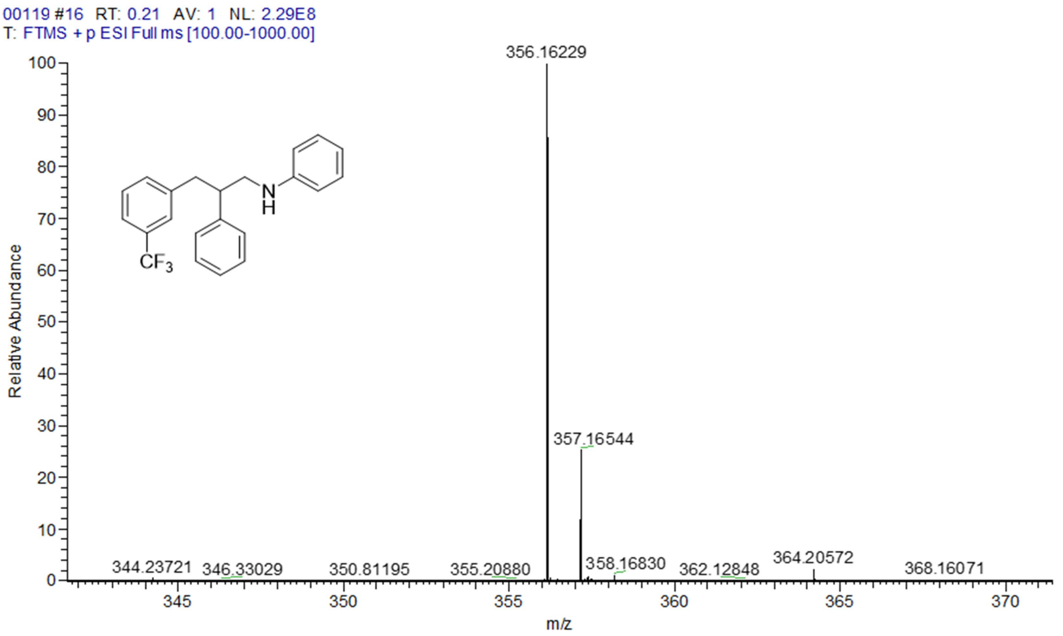

Supplementary Figure 161. HRMS of **3w**.

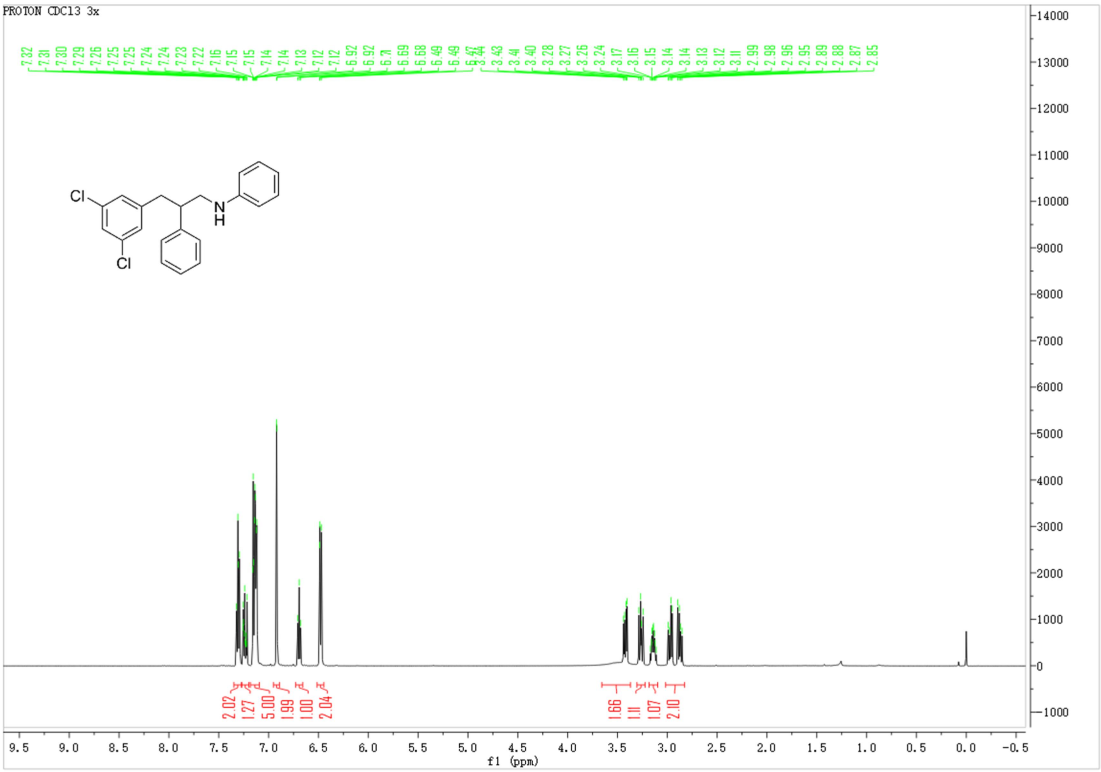

Supplementary Figure 162. ^{1}H NMR spectrum of **3x** in $CDCl_3$.

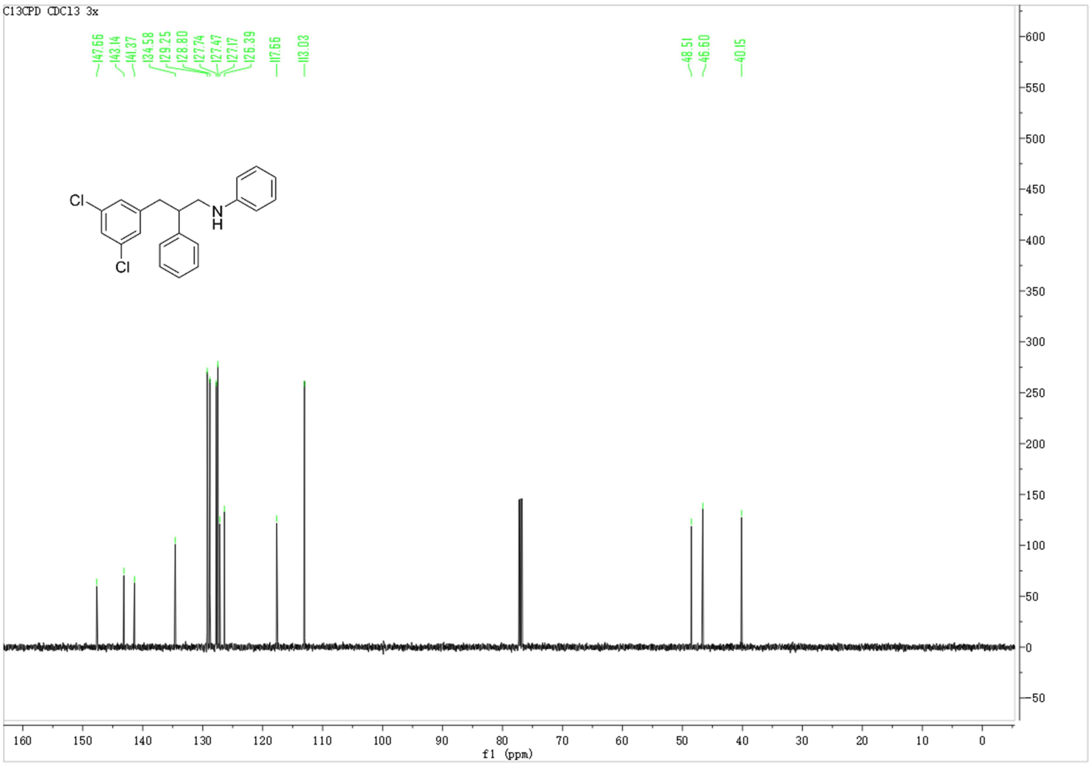

Supplementary Figure 163. ^{13}C NMR spectrum of **3x** in $CDCl_3$.

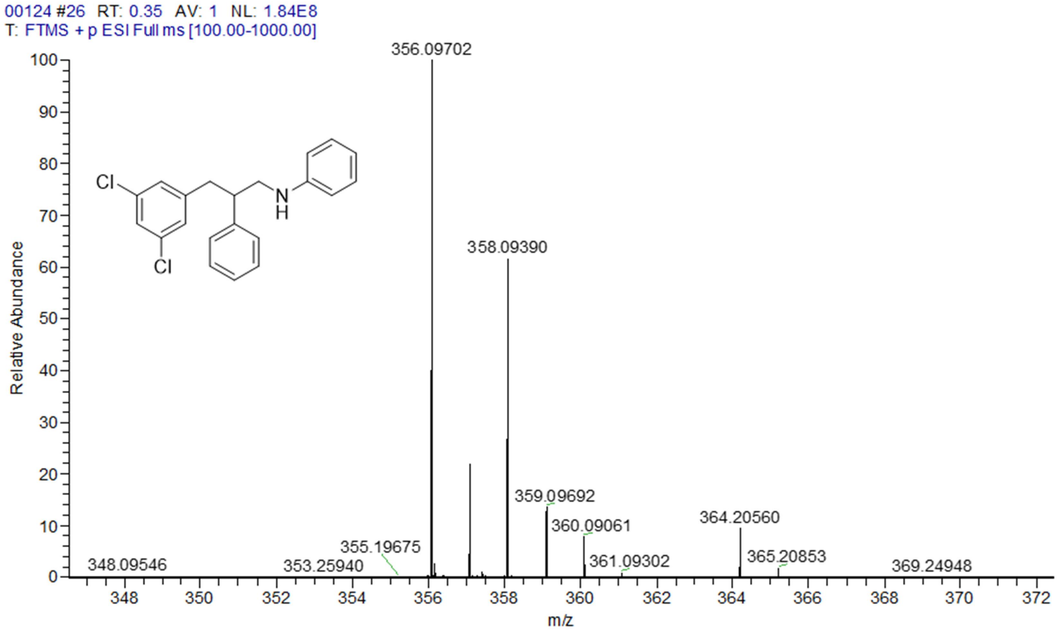

Supplementary Figure 164. HRMS of **3x**.

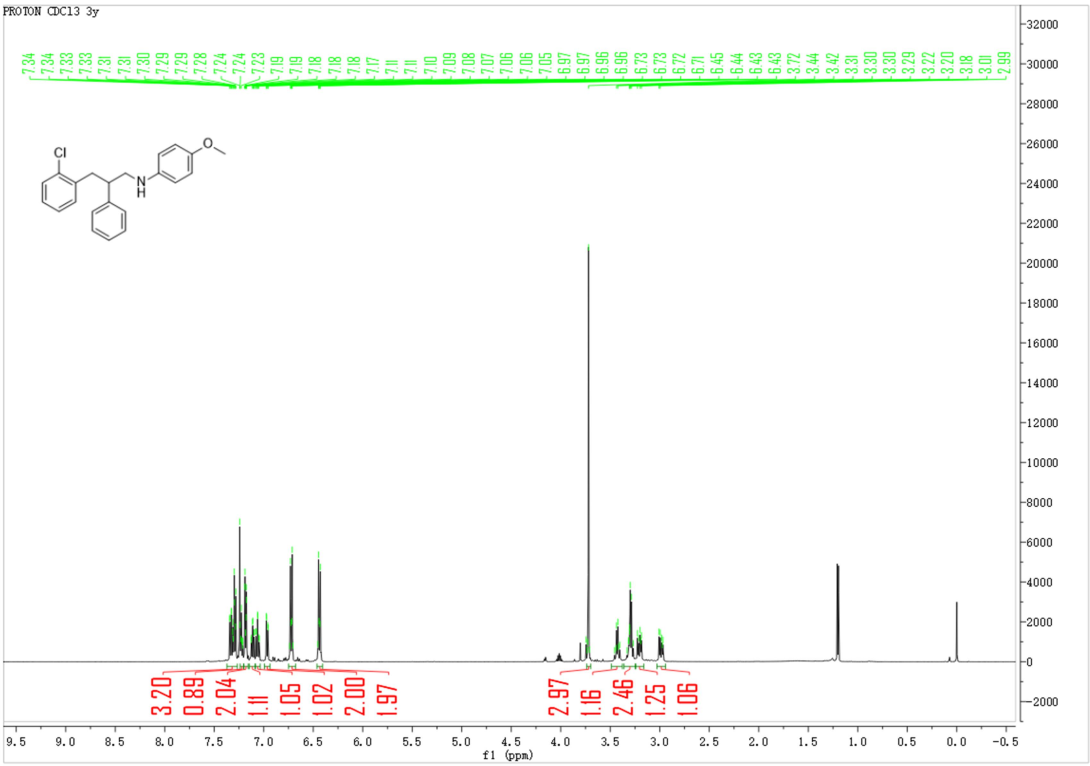

Supplementary Figure 165. ^{1}H NMR spectrum of **3y** in $CDCl_3$.

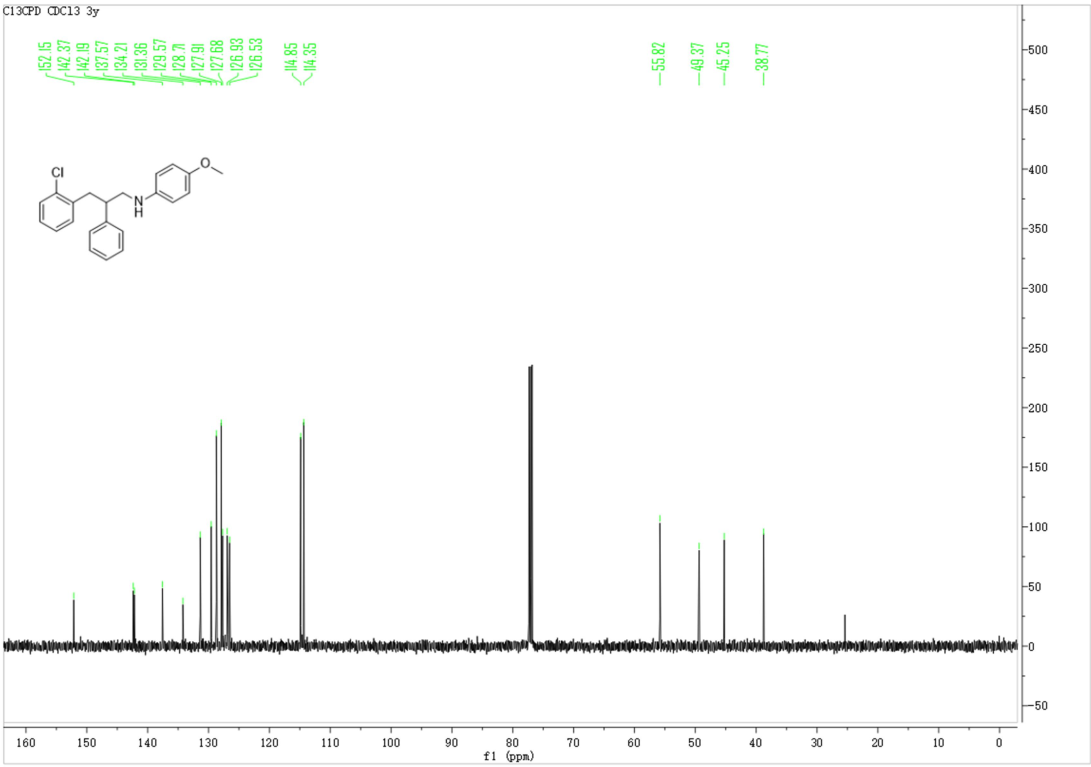

Supplementary Figure 166. ^{13}C NMR spectrum of **3y** in $CDCl_3$.

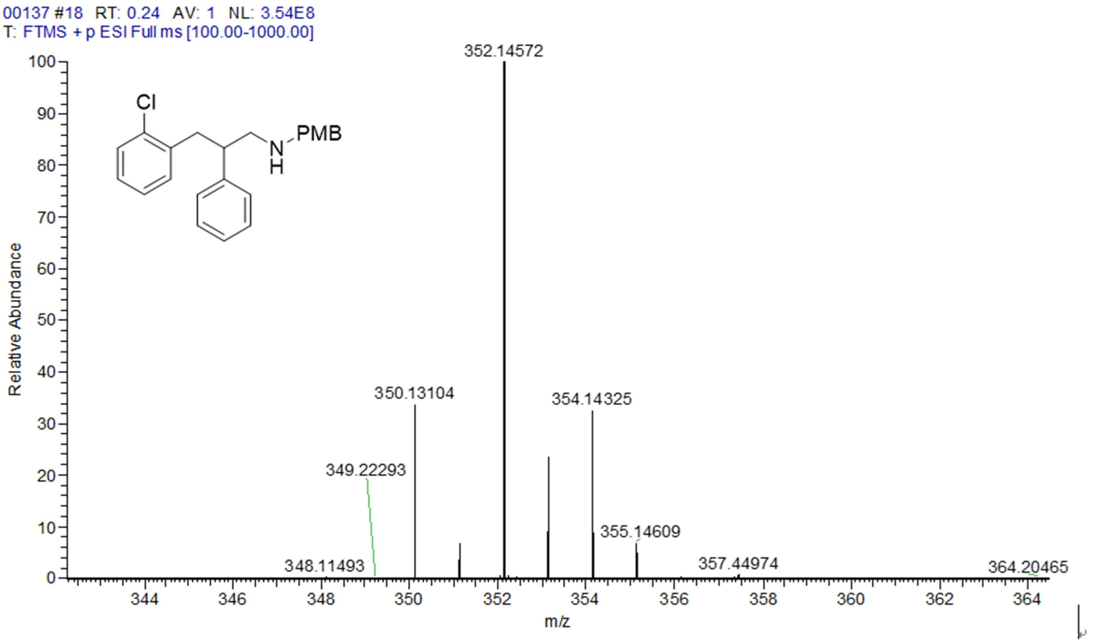

Supplementary Figure 167. HRMS of **3y**.

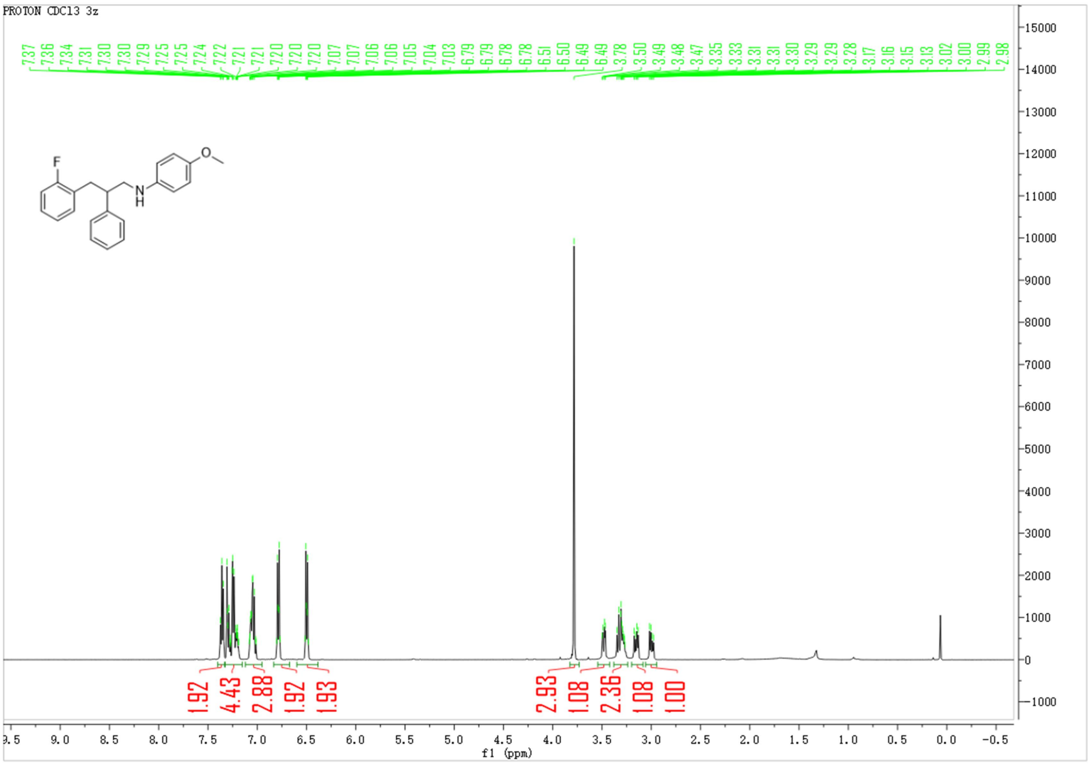

Supplementary Figure 168. ^{1}H NMR spectrum of **3z** in $CDCl_3$.

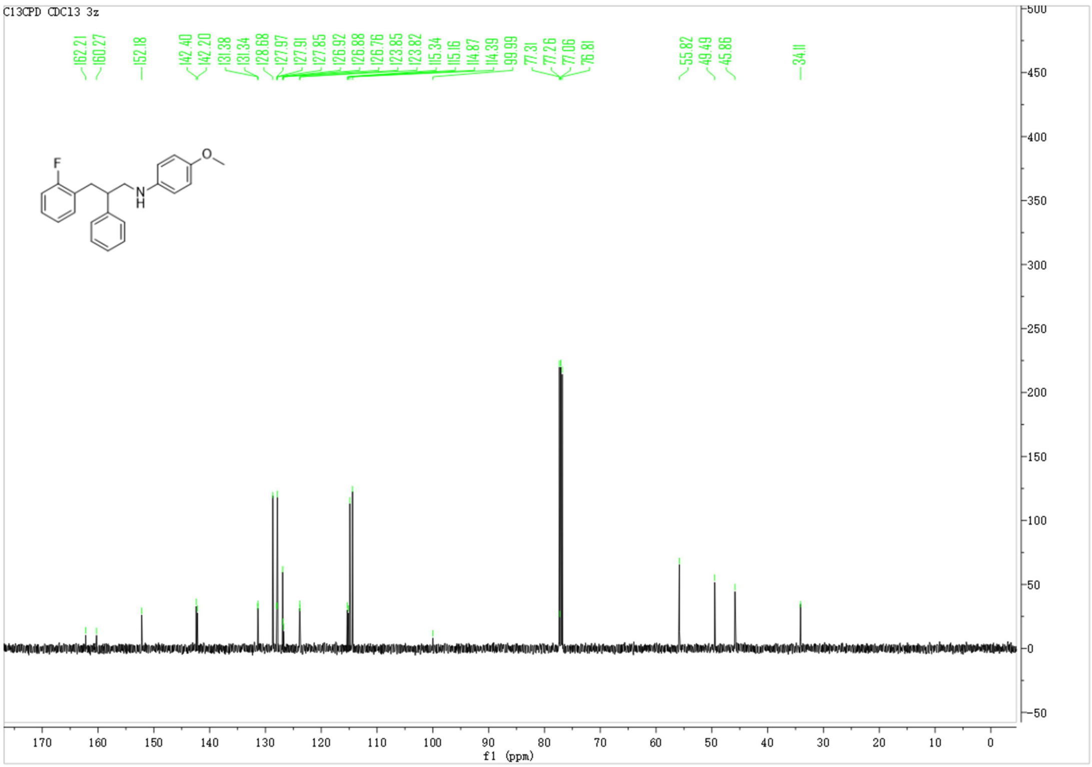

Supplementary Figure 169. ^{13}C NMR spectrum of **3z** in $CDCl_3$.

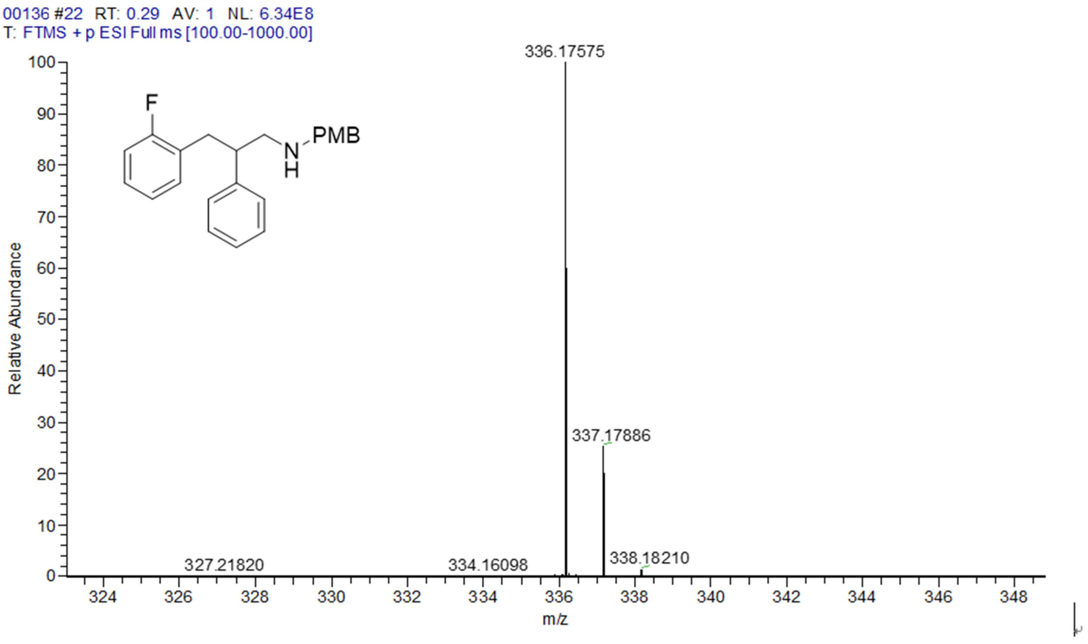

Supplementary Figure 170. HRMS of **3z**.

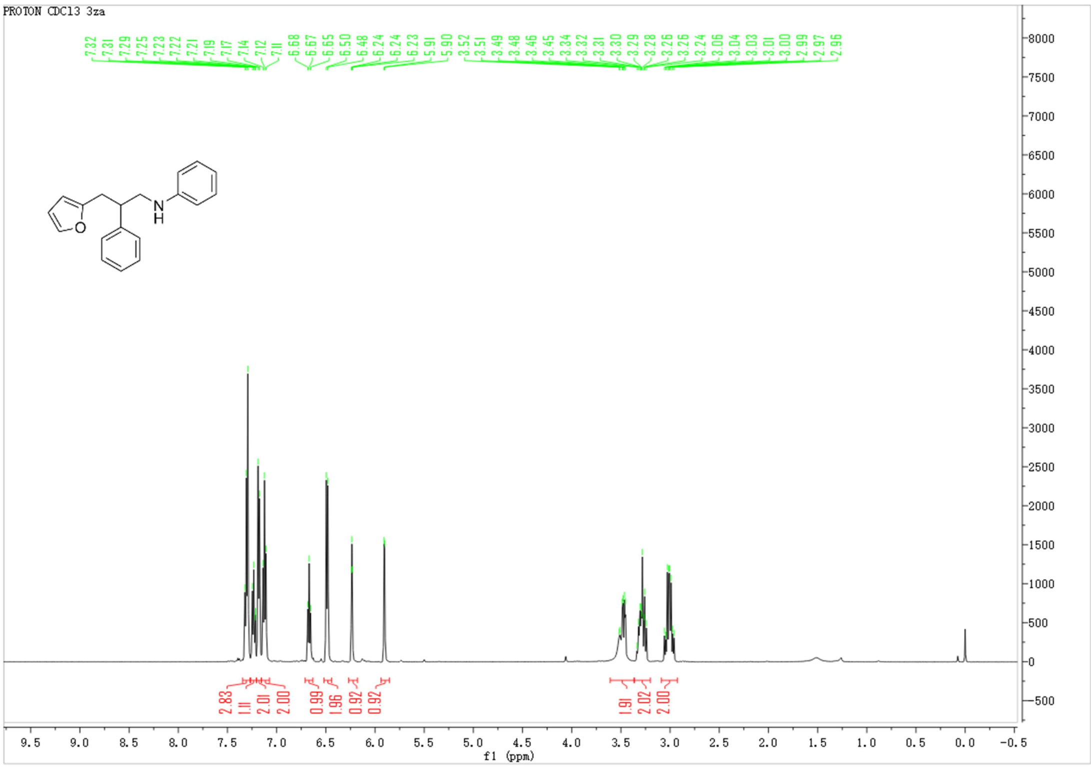

Supplementary Figure 171. ^{1}H NMR spectrum of **3za** in $CDCl_3$.

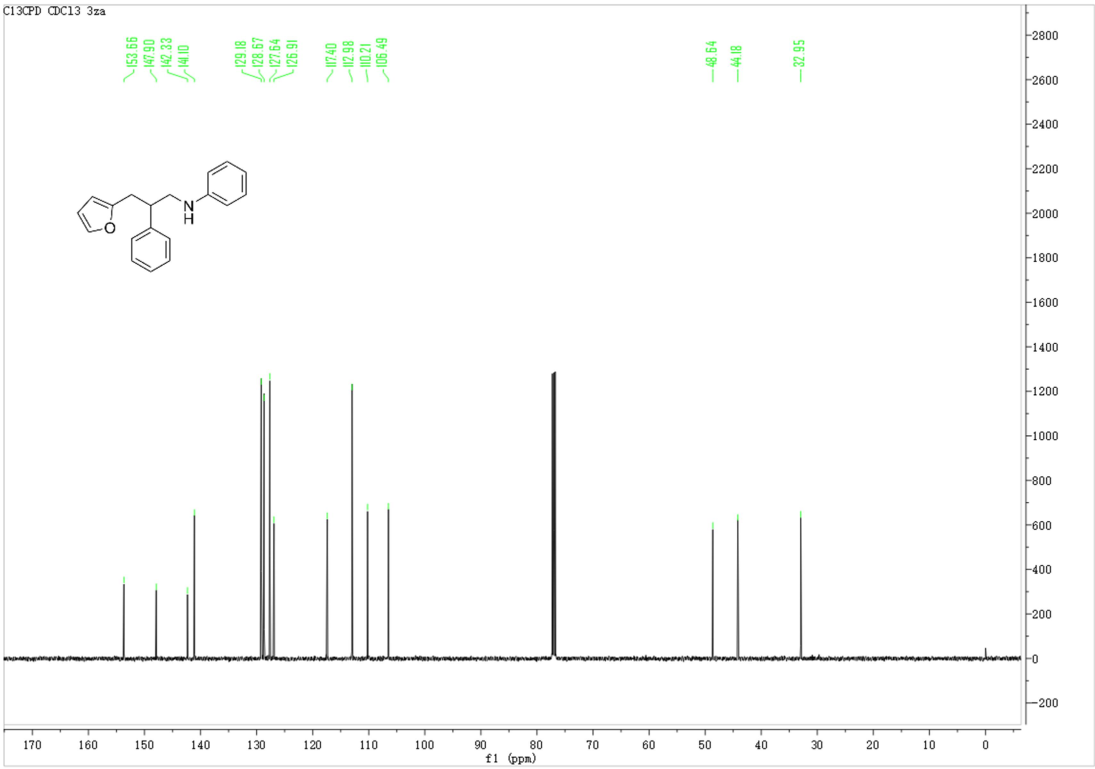

Supplementary Figure 172. ^{13}C NMR spectrum of **3za** in $CDCl_3$.

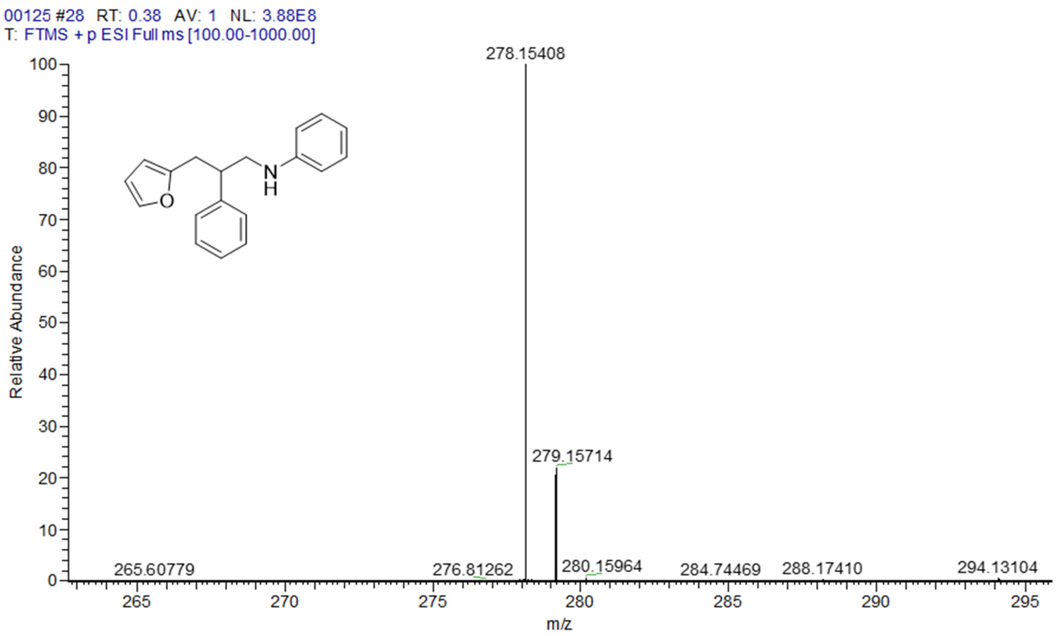

Supplementary Figure 173. HRMS of **3za**.

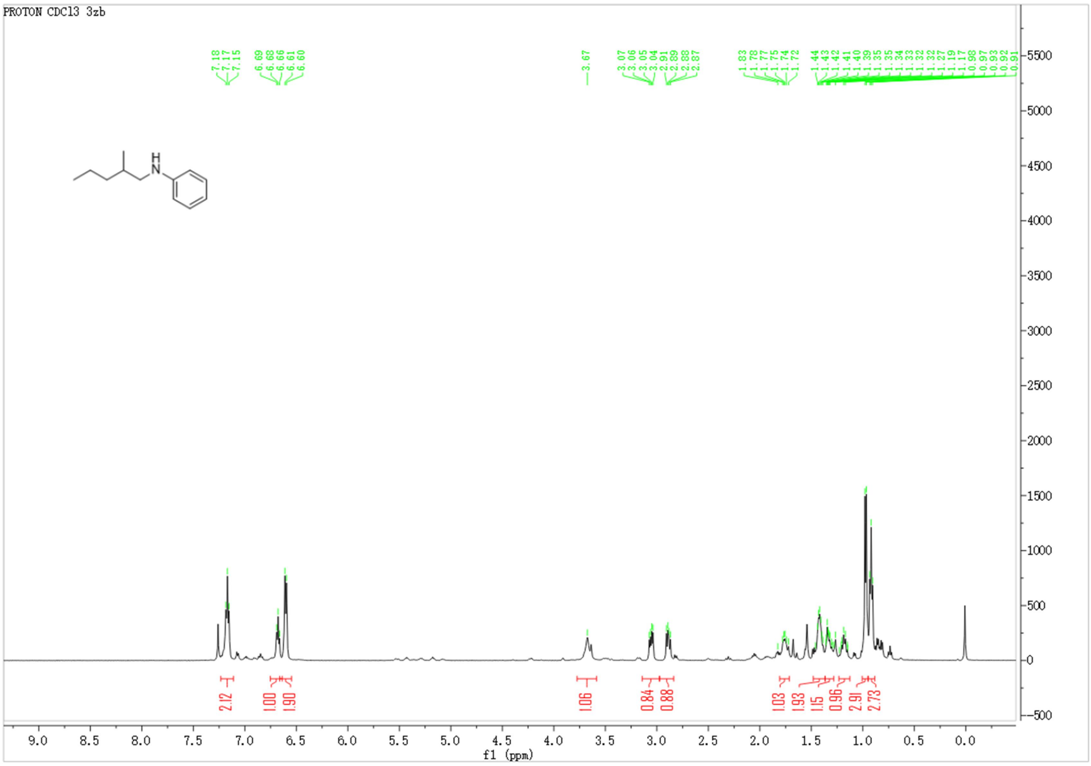

Supplementary Figure 174. ^{1}H NMR spectrum of **3zb** in $CDCl_3$.

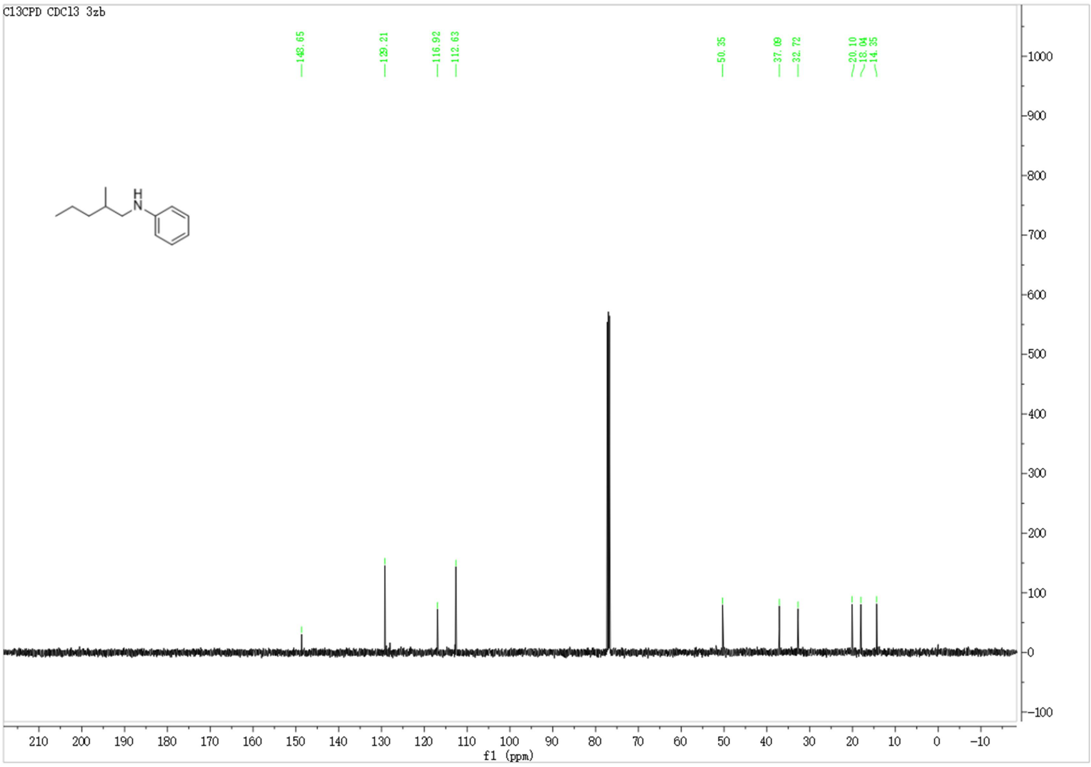

Supplementary Figure 175. ^{13}C NMR spectrum of **3zb** in $CDCl_3$.

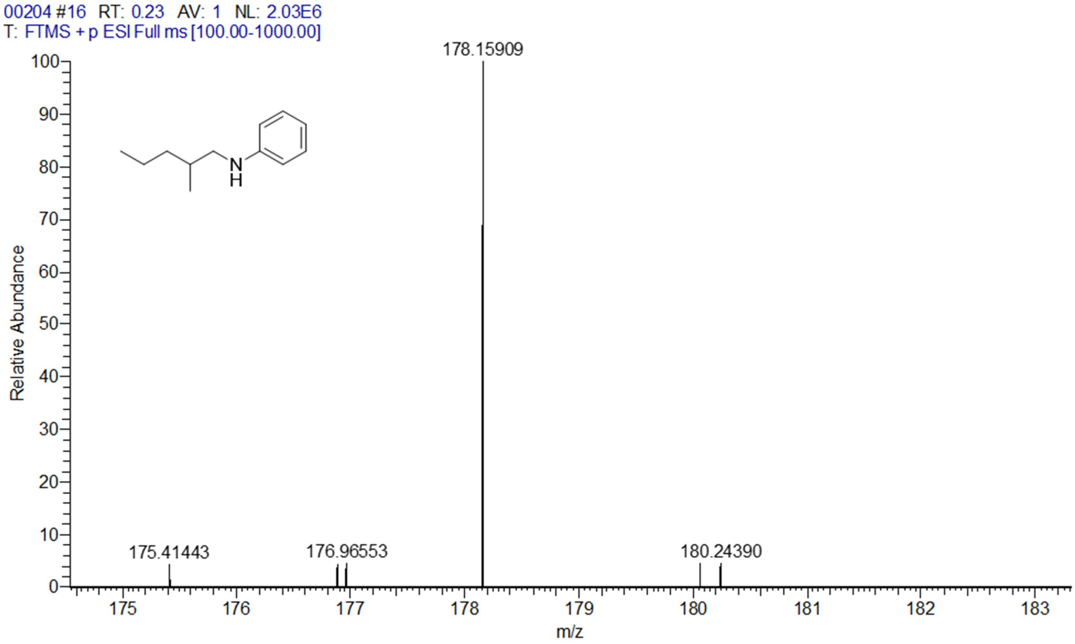

Supplementary Figure 176. HRMS of **3zb**.

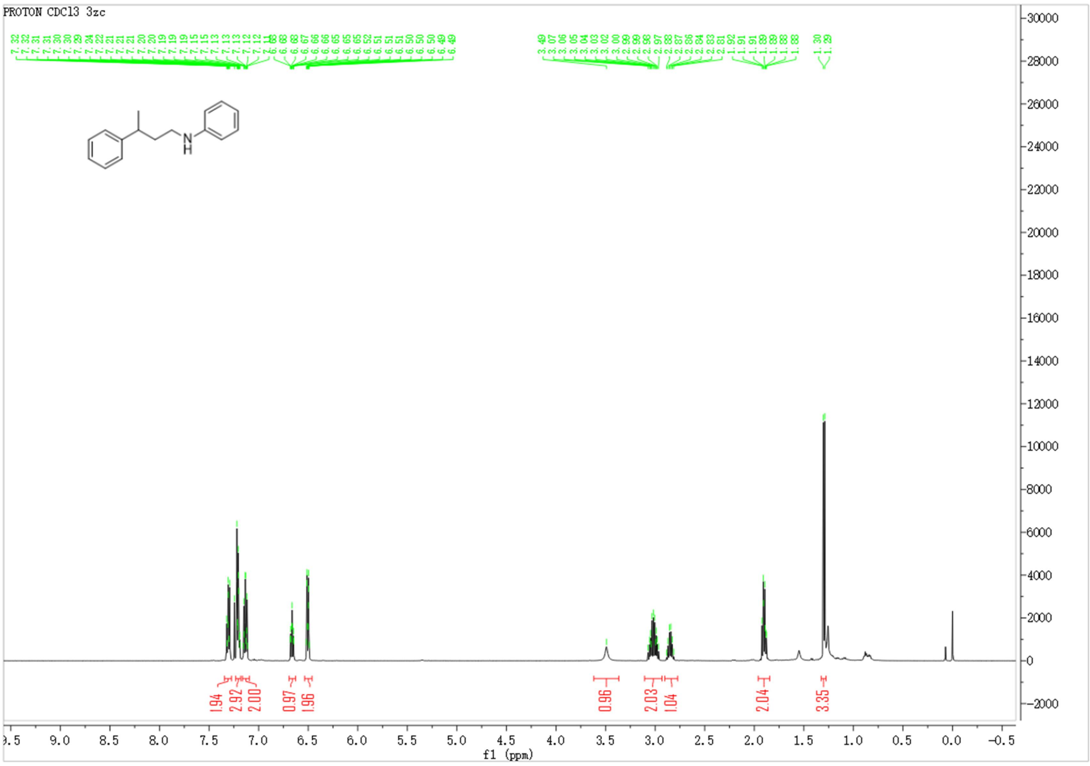

Supplementary Figure 177. ^{1}H NMR spectrum of **3zc** in $CDCl_3$.

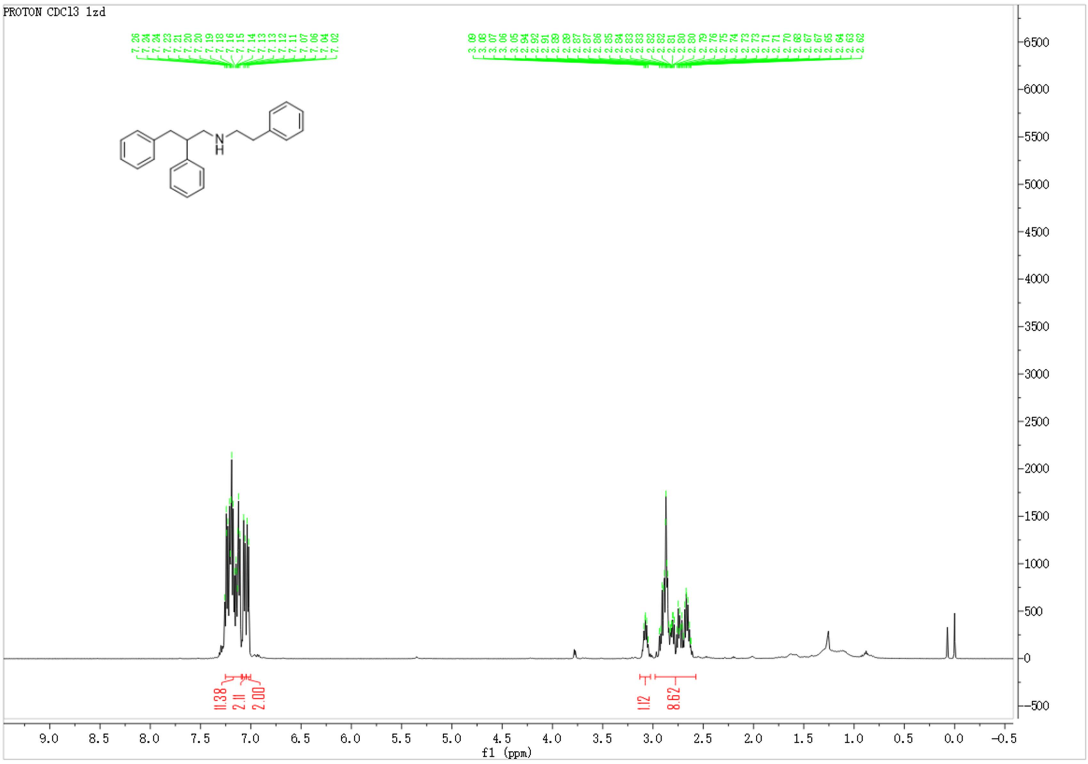

Supplementary Figure 178. ^{1}H NMR spectrum of **3zd** in $CDCl_3$.

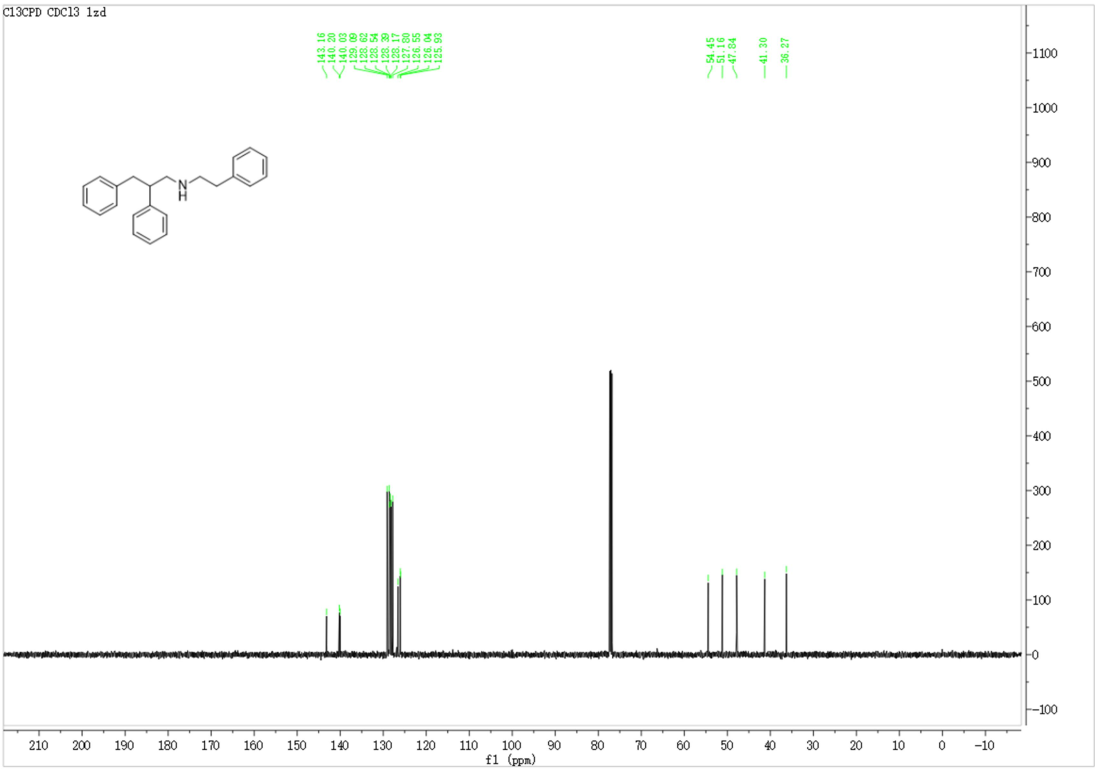

Supplementary Figure 179. ^{13}C NMR spectrum of **3zd** in $CDCl_3$.

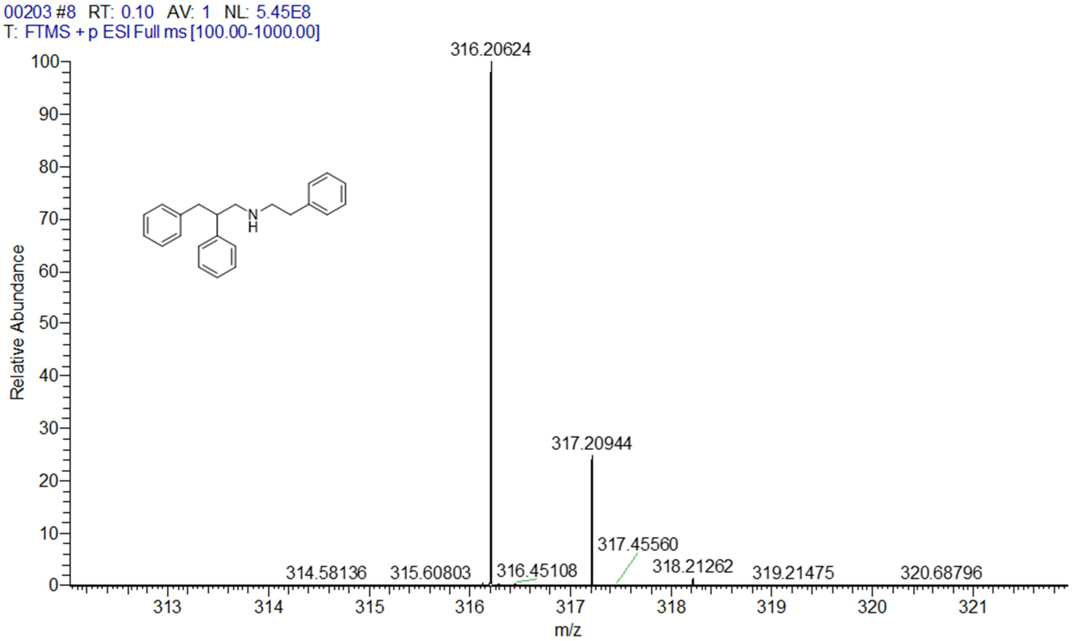

Supplementary Figure 180. HRMS of **3zd**.

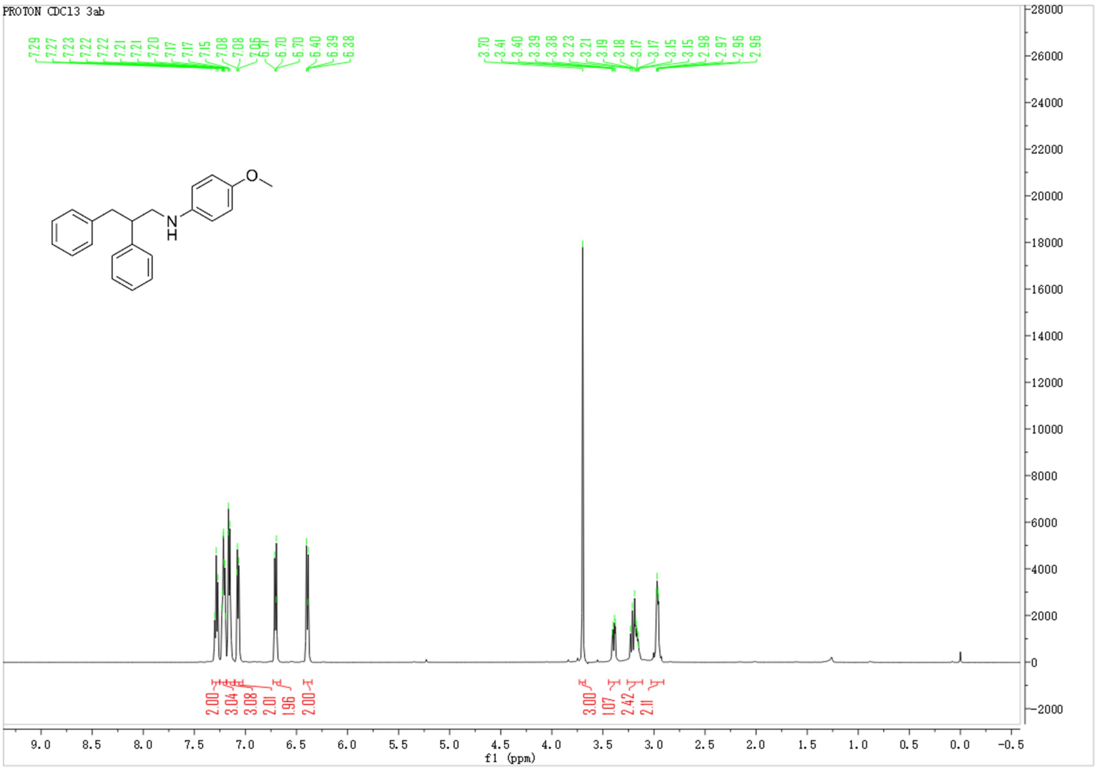

Supplementary Figure 181. ^{1}H NMR spectrum of **3ab** in $CDCl_3$.

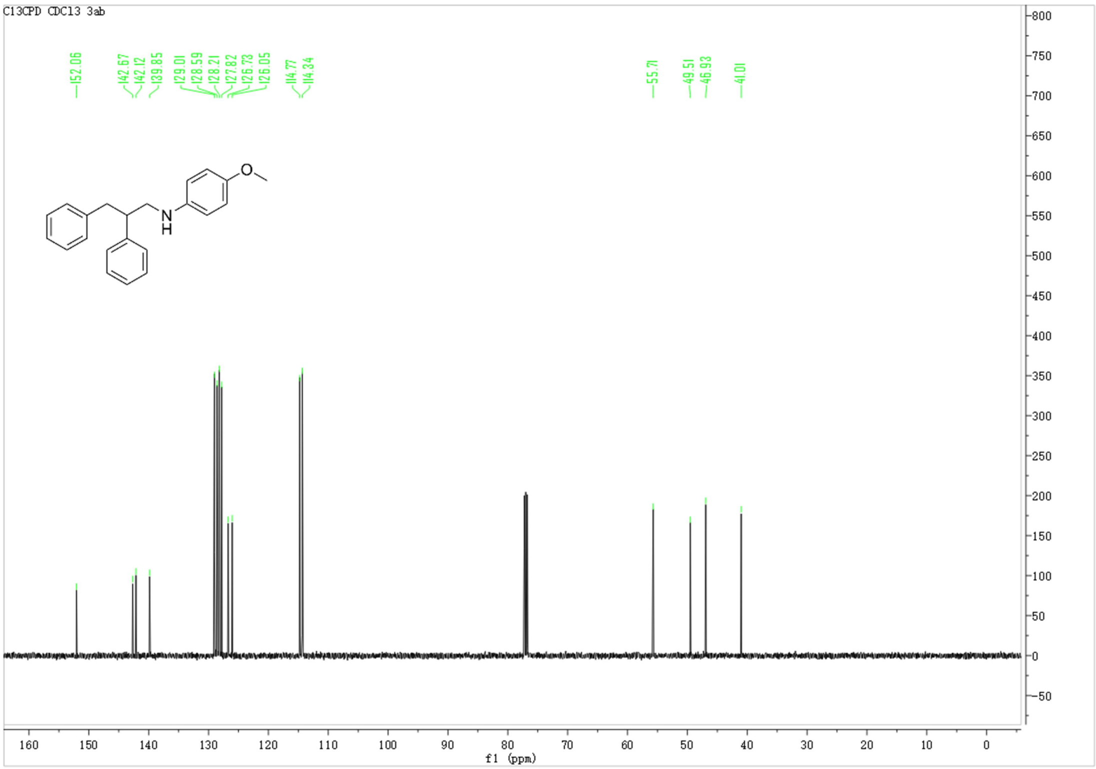

Supplementary Figure 182. ^{13}C NMR spectrum of **3ab** in $CDCl_3$.

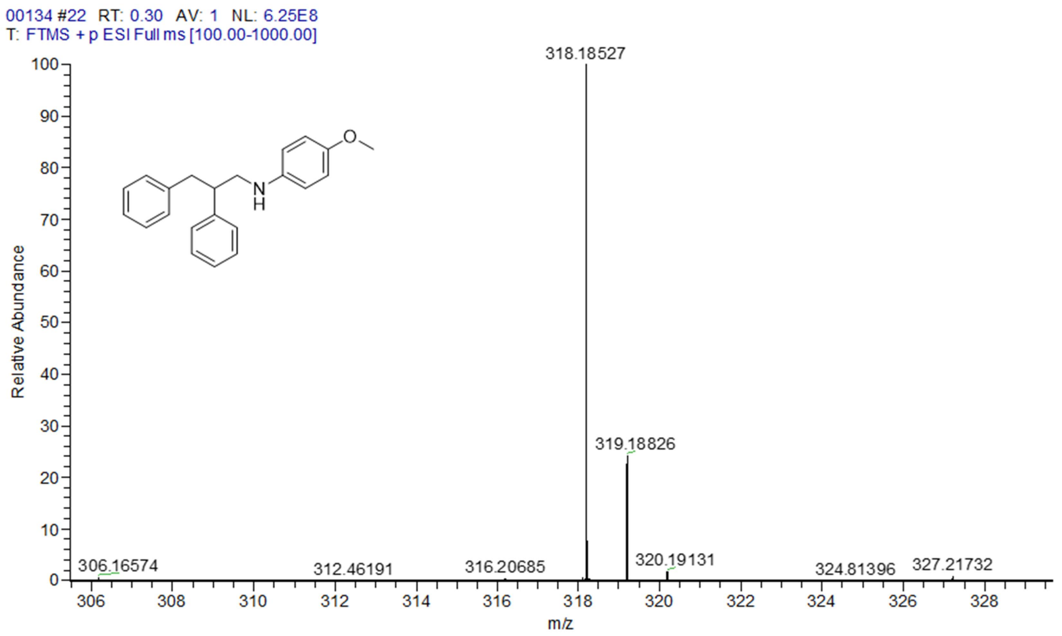

Supplementary Figure 183. HRMS of **3ab**.

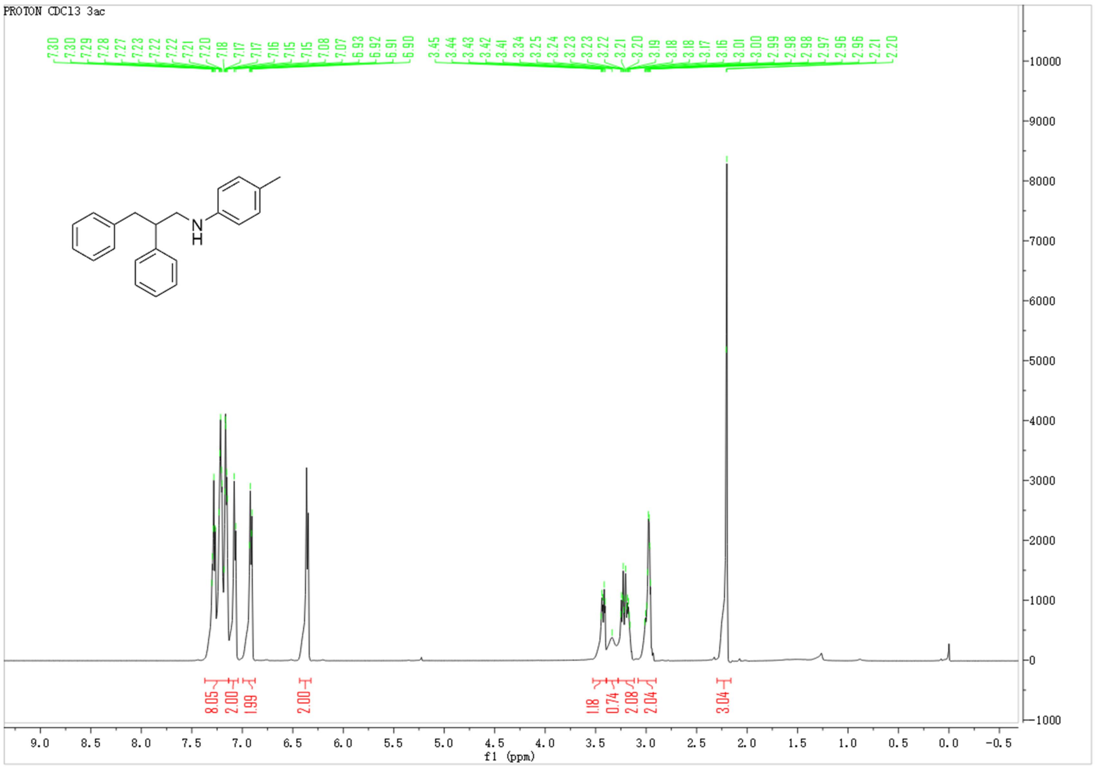

Supplementary Figure 184. ^{1}H NMR spectrum of **3ac** in $CDCl_3$.

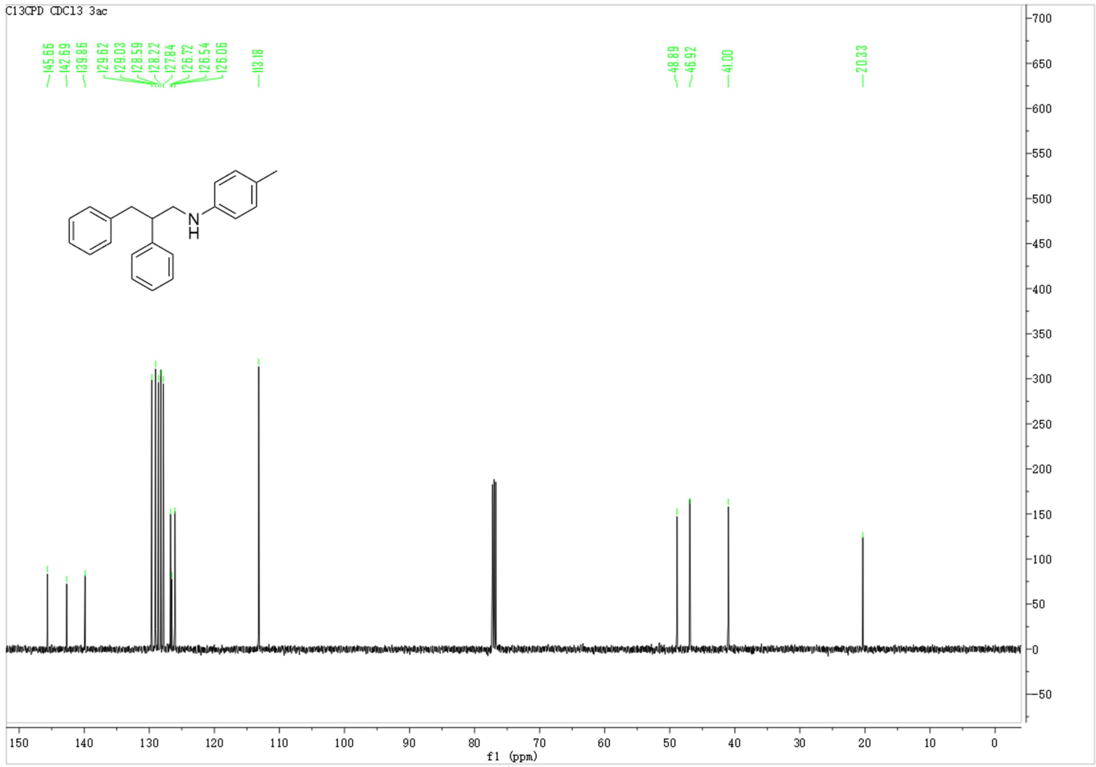

Supplementary Figure 185. ^{13}C NMR spectrum of **3ac** in $CDCl_3$.

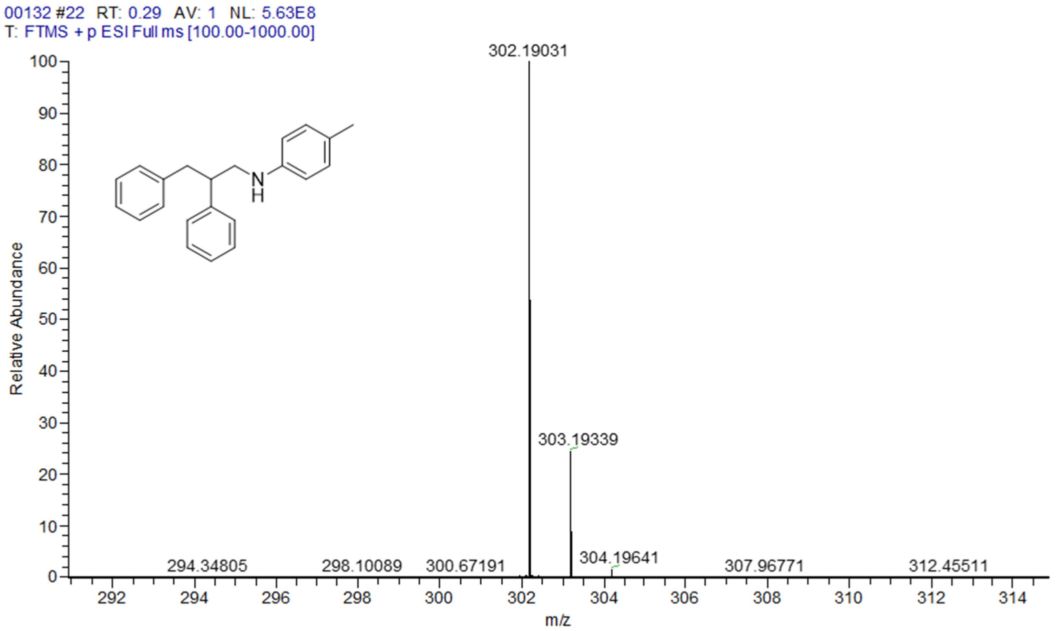

Supplementary Figure 186. HRMS of **3ac**.

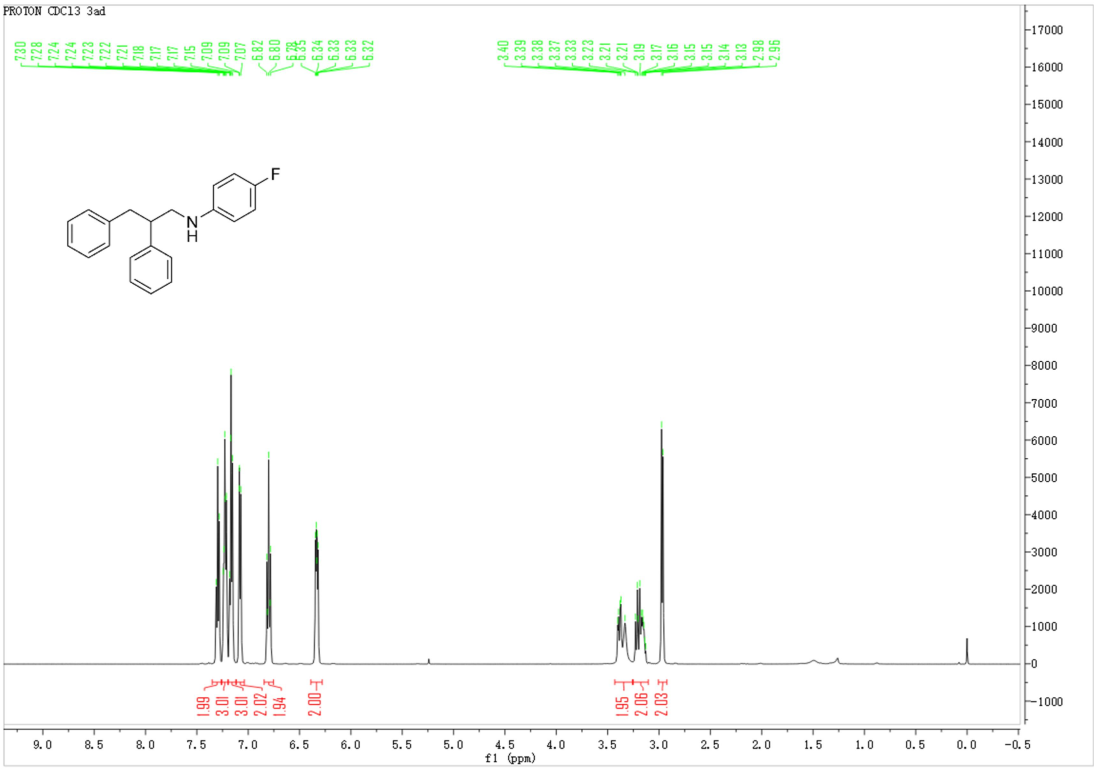

Supplementary Figure 187. ^{1}H NMR spectrum of **3ad** in $CDCl_3$.

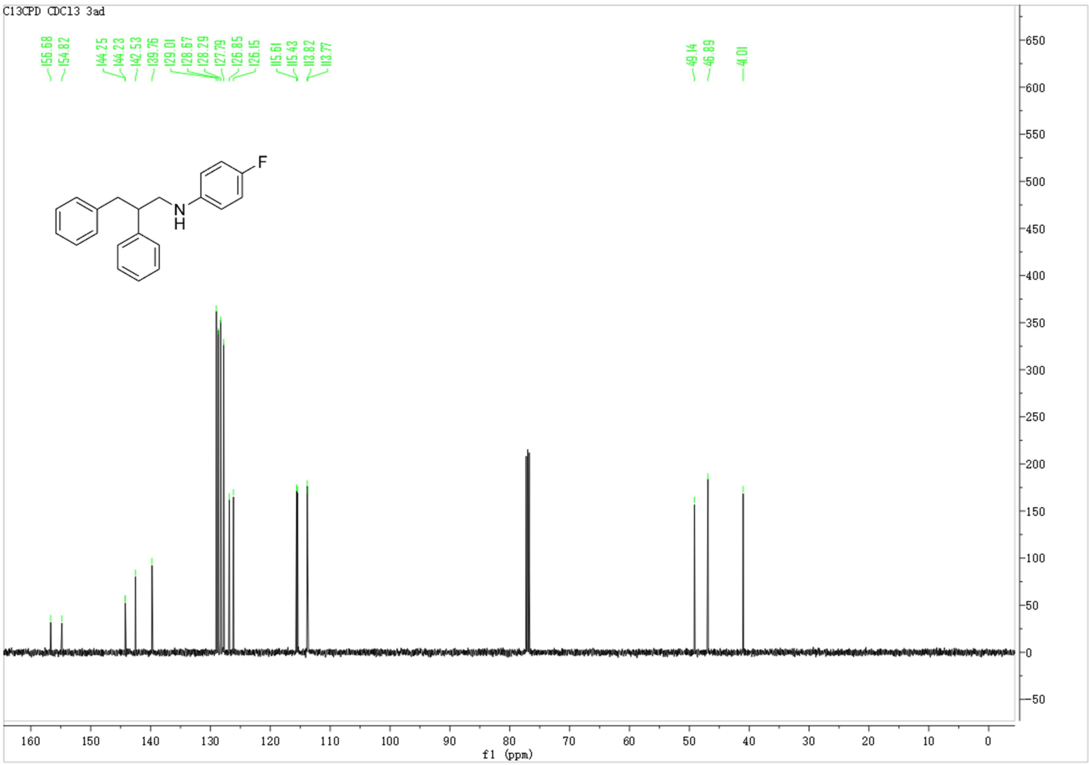

Supplementary Figure 188. ^{13}C NMR spectrum of **3ad** in $CDCl_3$.

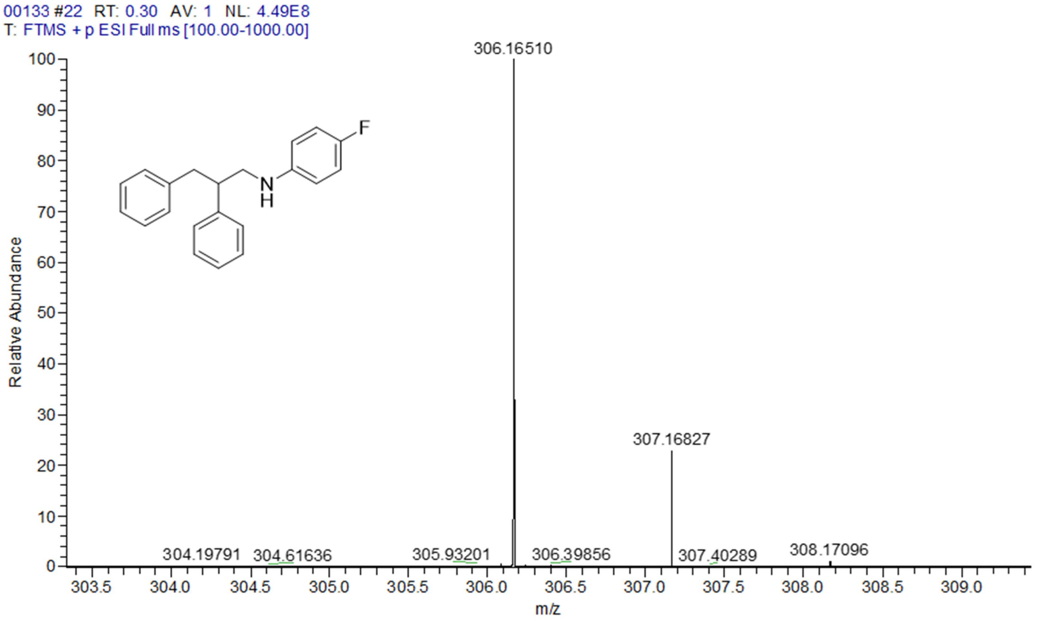

Supplementary Figure 189. HRMS of **3ad**.

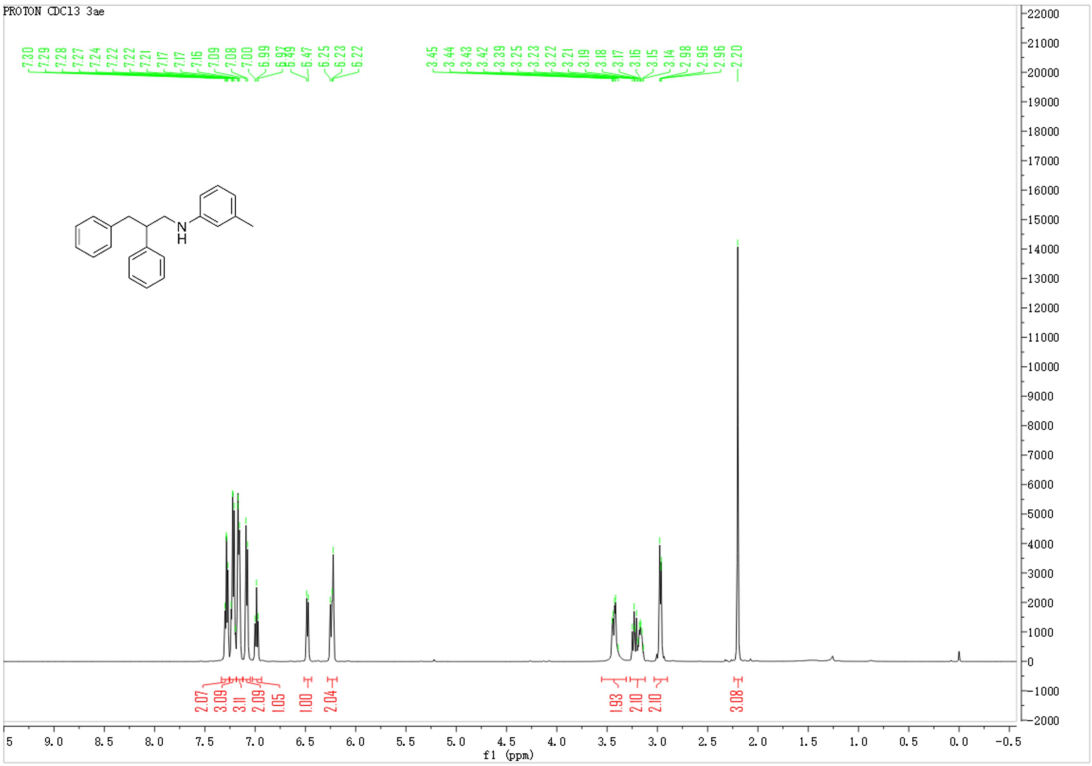

Supplementary Figure 190. ^{1}H NMR spectrum of **3ae** in $CDCl_3$.

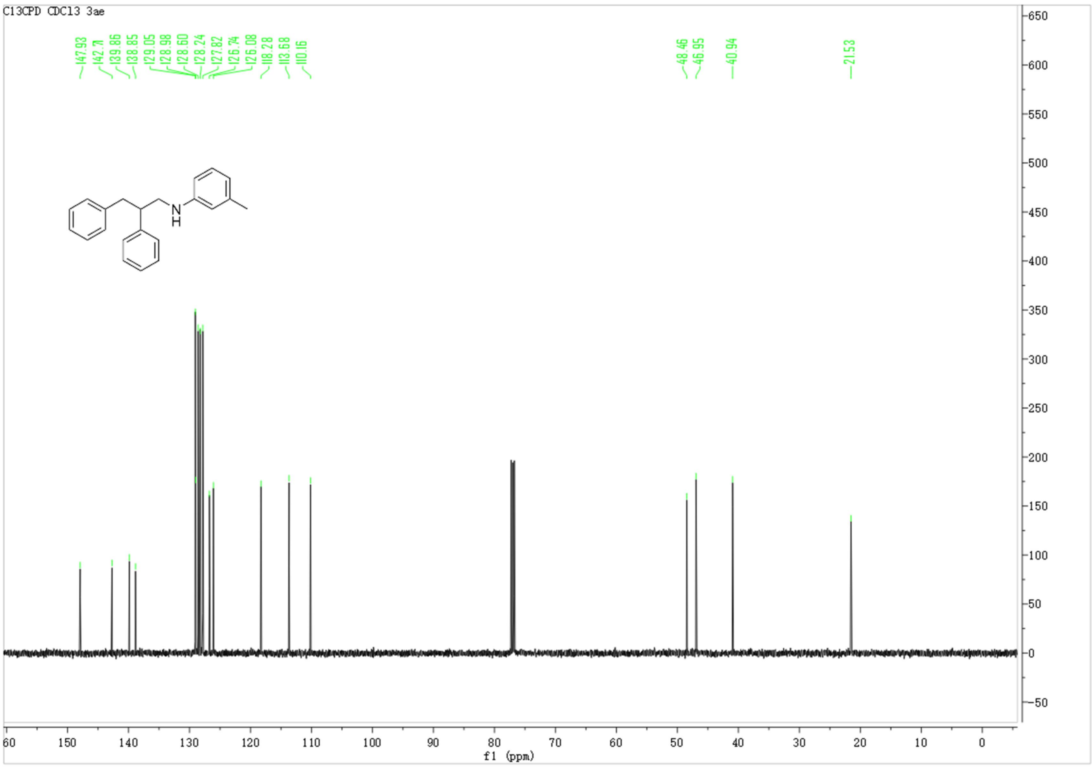

Supplementary Figure 191. ^{13}C NMR spectrum of **3ae** in $CDCl_3$.

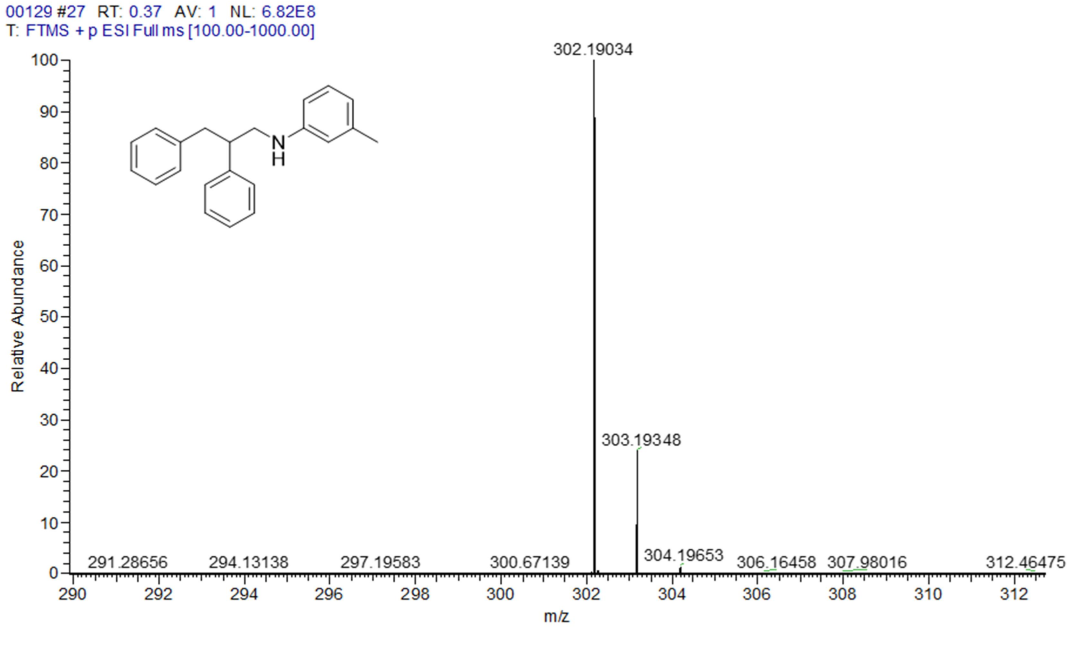

Supplementary Figure 192. HRMS of **3ae**.

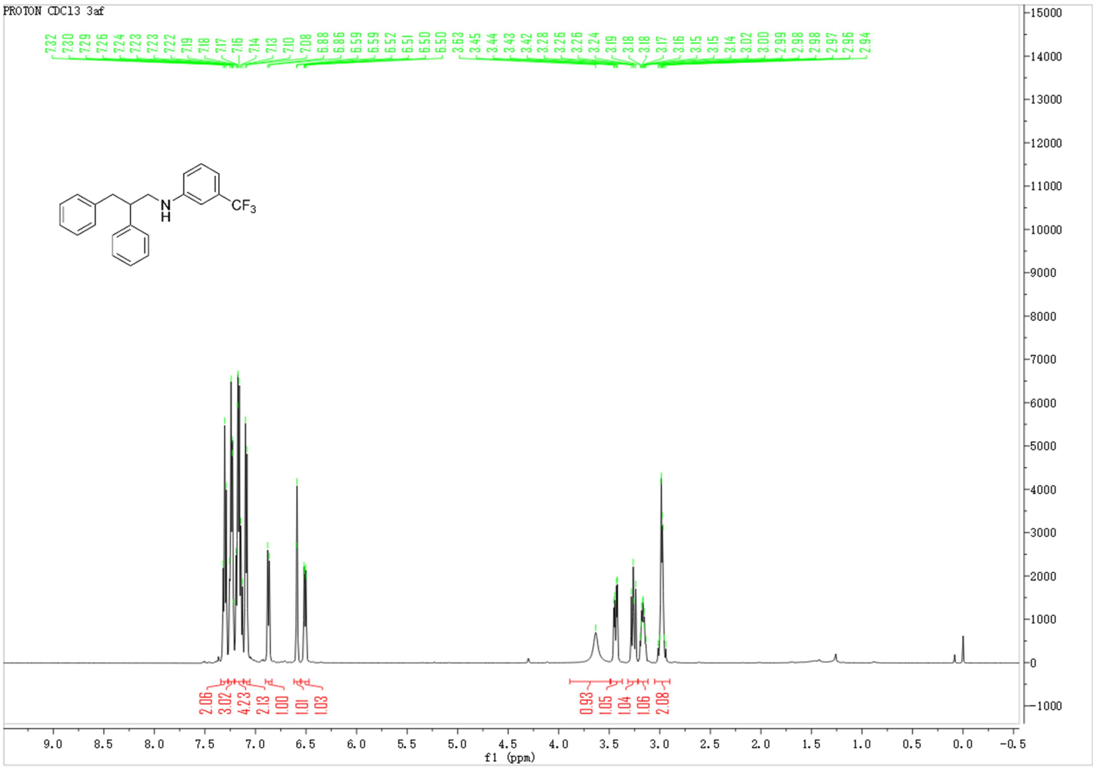

Supplementary Figure 193. ^{1}H NMR spectrum of **3af** in $CDCl_3$.

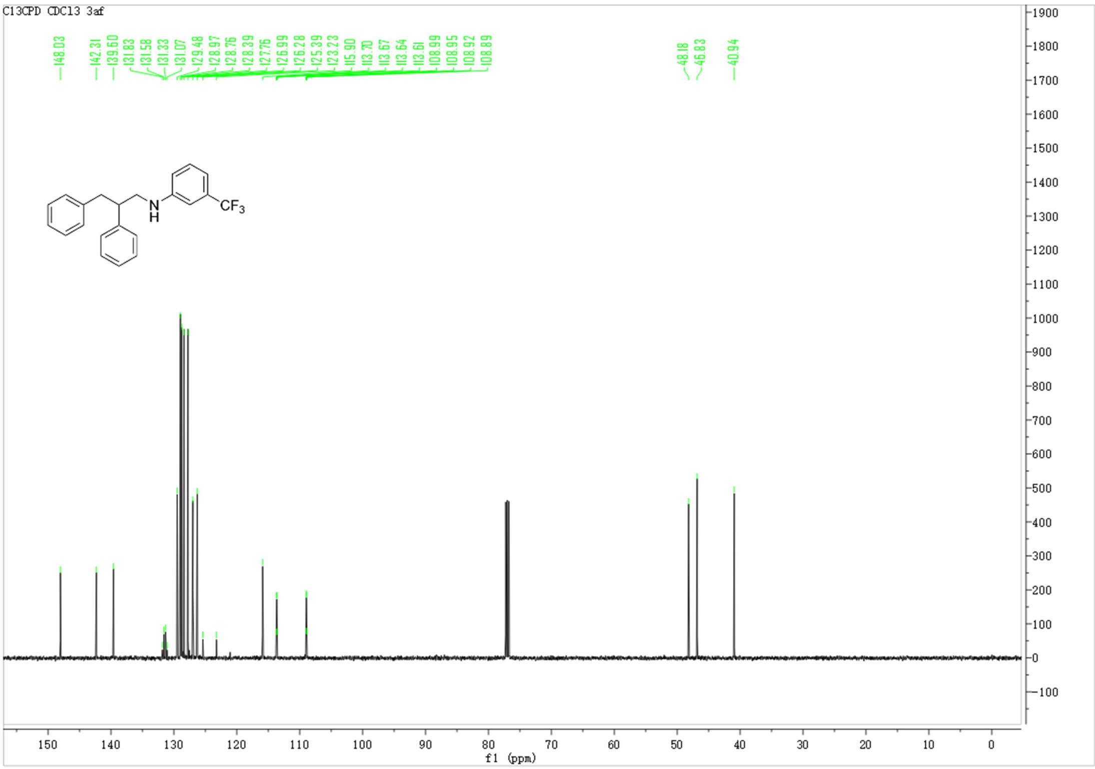

Supplementary Figure 194. ^{13}C NMR spectrum of **3af** in $CDCl_3$.

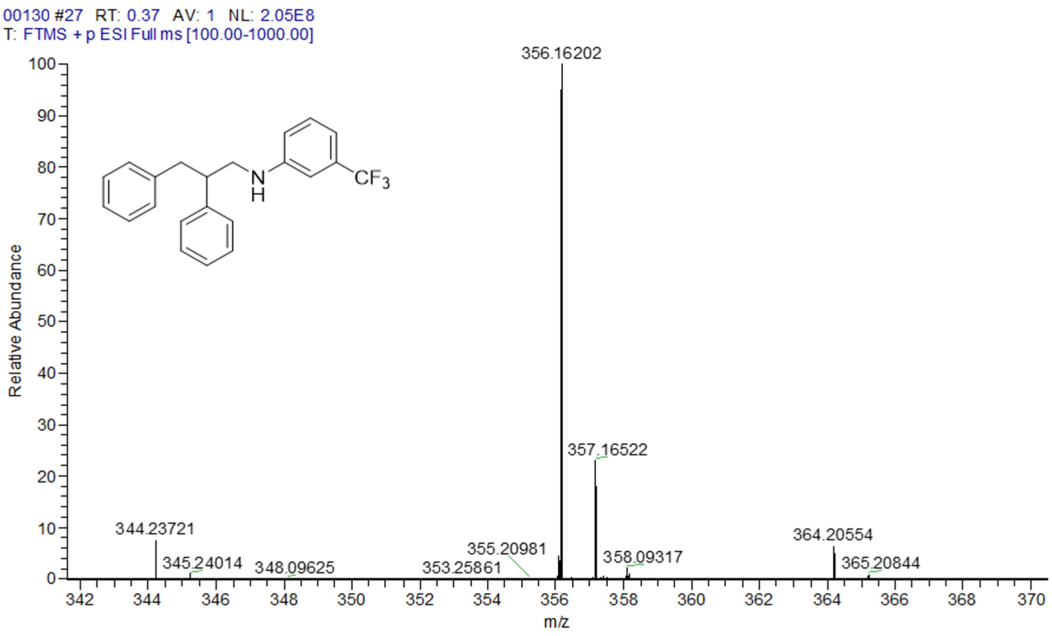

Supplementary Figure 195. HRMS of **3af**.

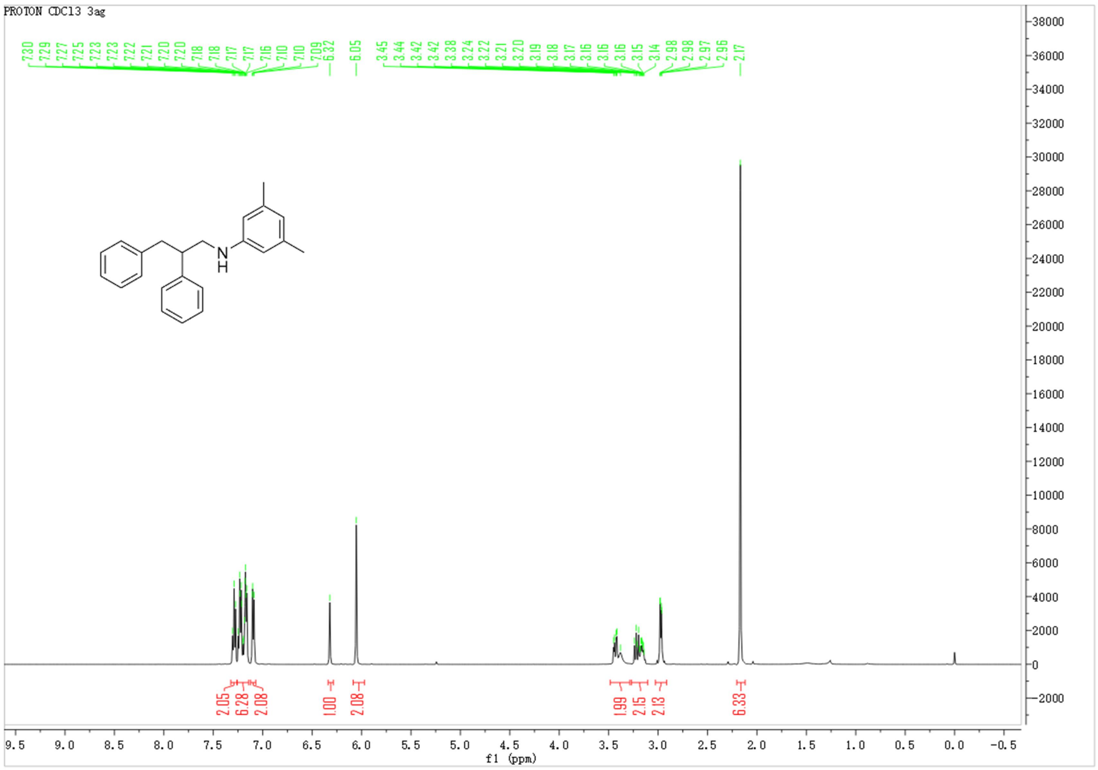

Supplementary Figure 196. ^{1}H NMR spectrum of **3ag** in $CDCl_3$.

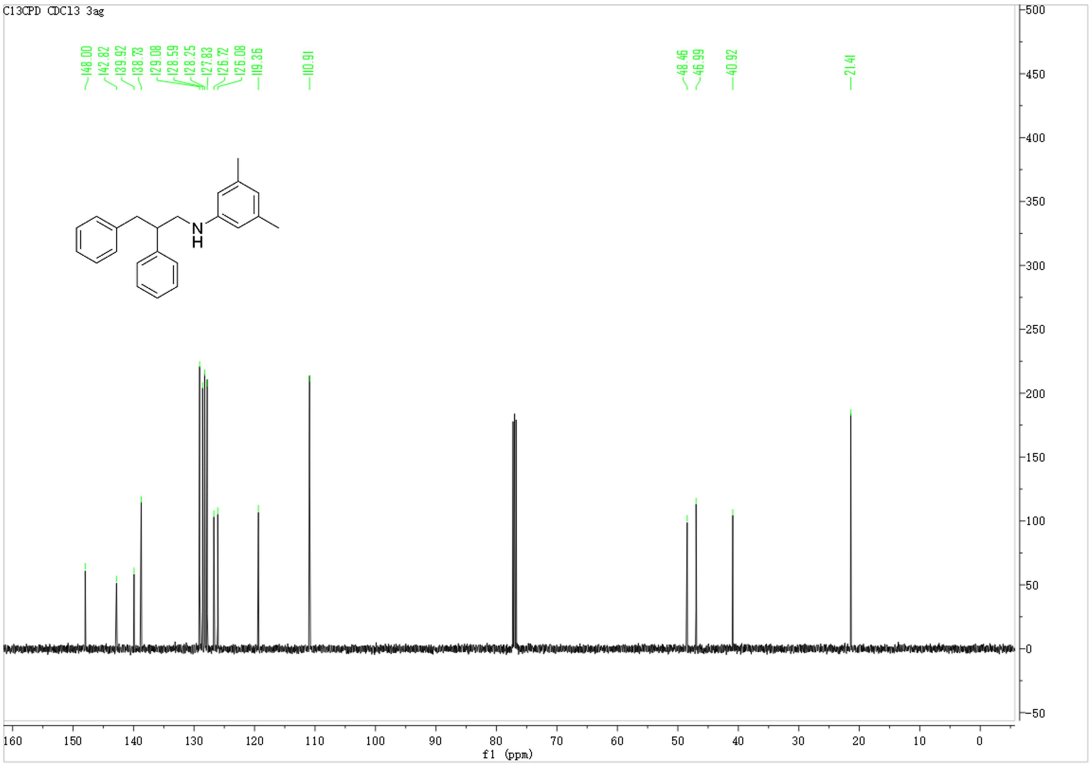

Supplementary Figure 197. ^{13}C NMR spectrum of **3ag** in $CDCl_3$.

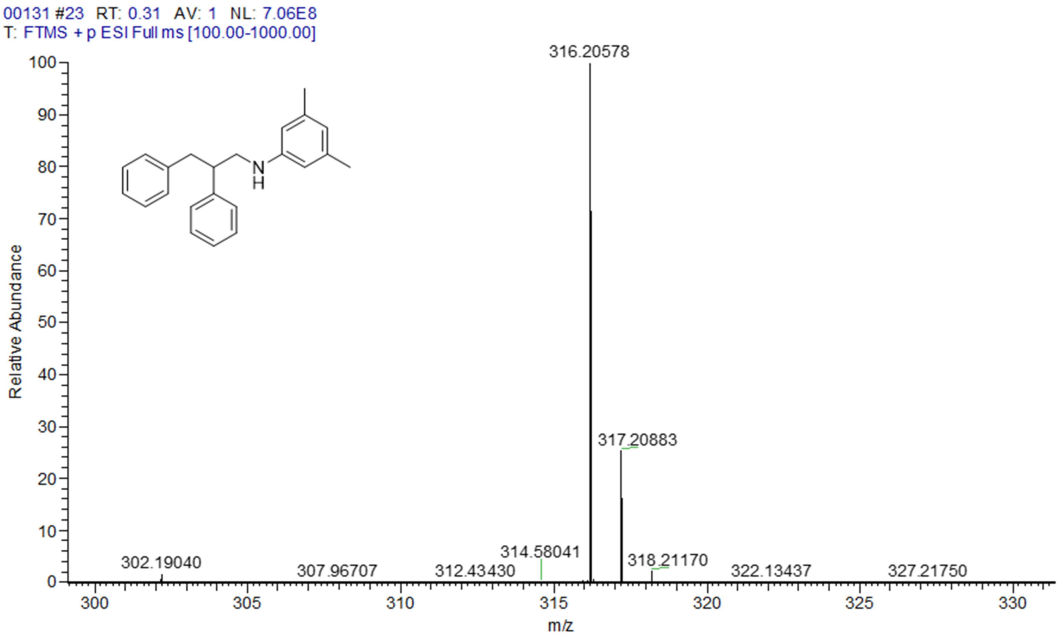

Supplementary Figure 198. HRMS of **3ag**.

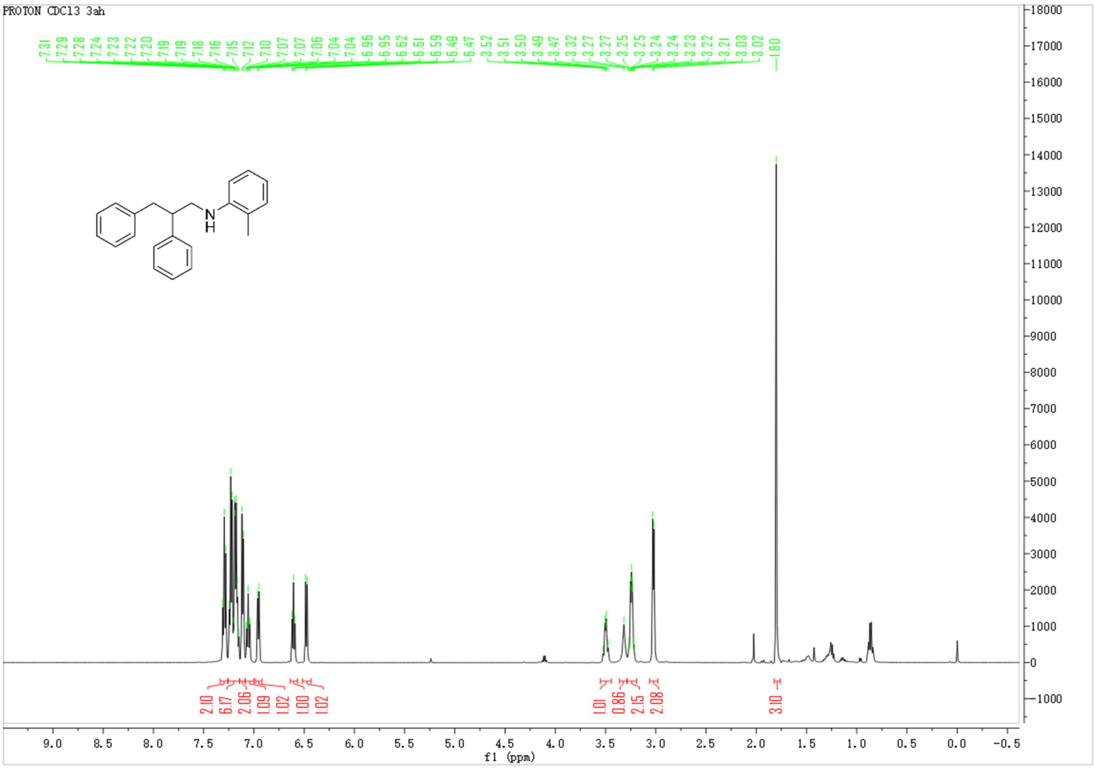

Supplementary Figure 199. ^{1}H NMR spectrum of **3ah** in $CDCl_3$.

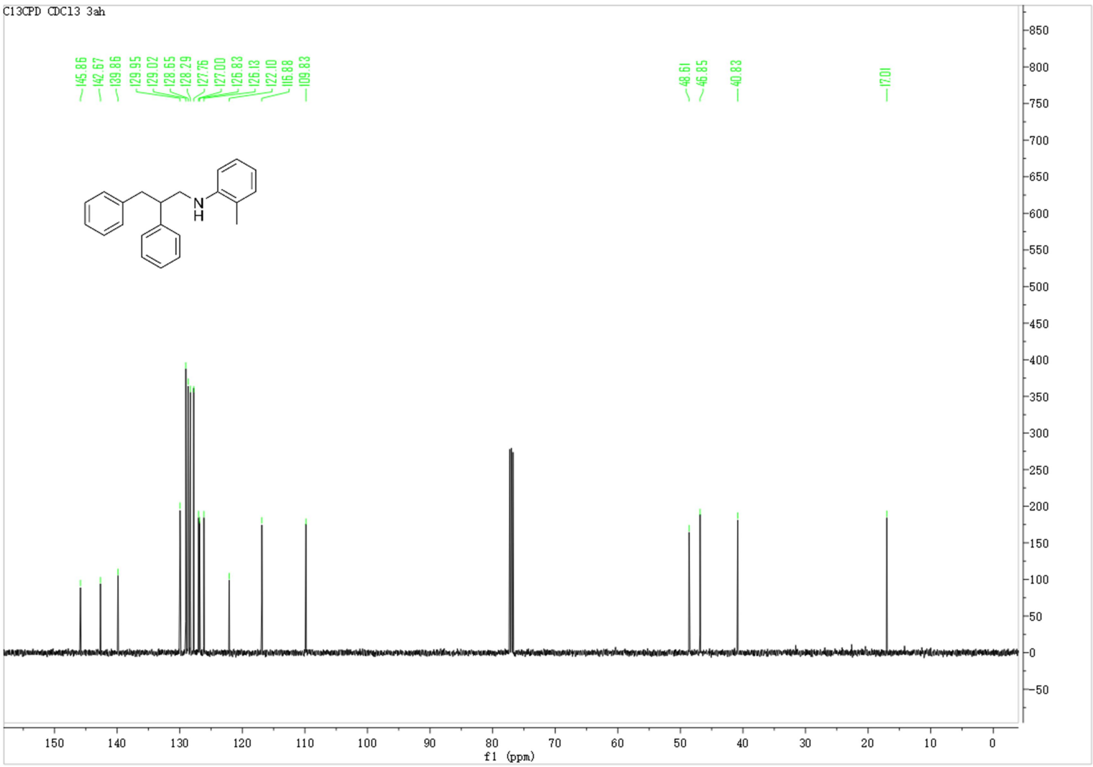

Supplementary Figure 200. ^{13}C NMR spectrum of **3ah** in $CDCl_3$.

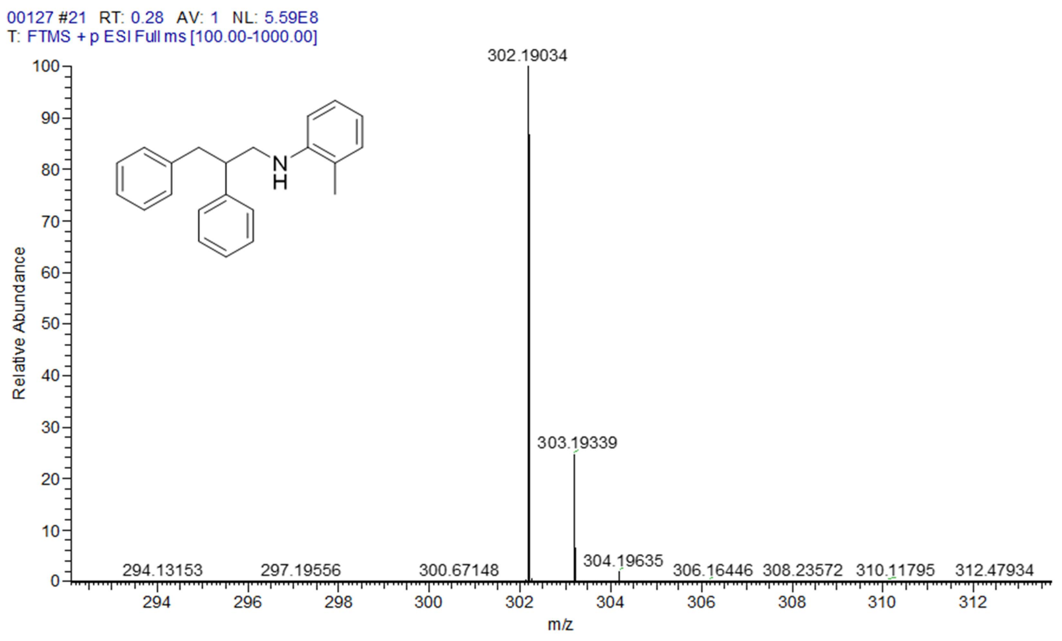

Supplementary Figure 201. HRMS of **3ah**.

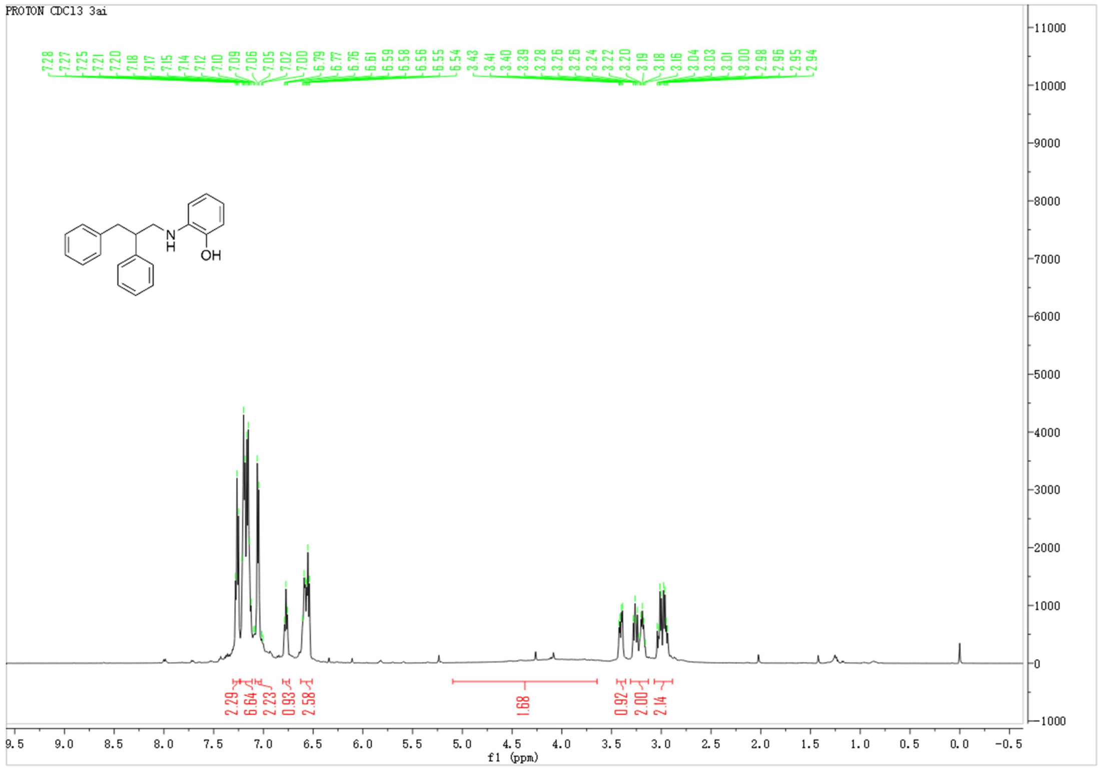

Supplementary Figure 202. ^{1}H NMR spectrum of **3ai** in $CDCl_3$.

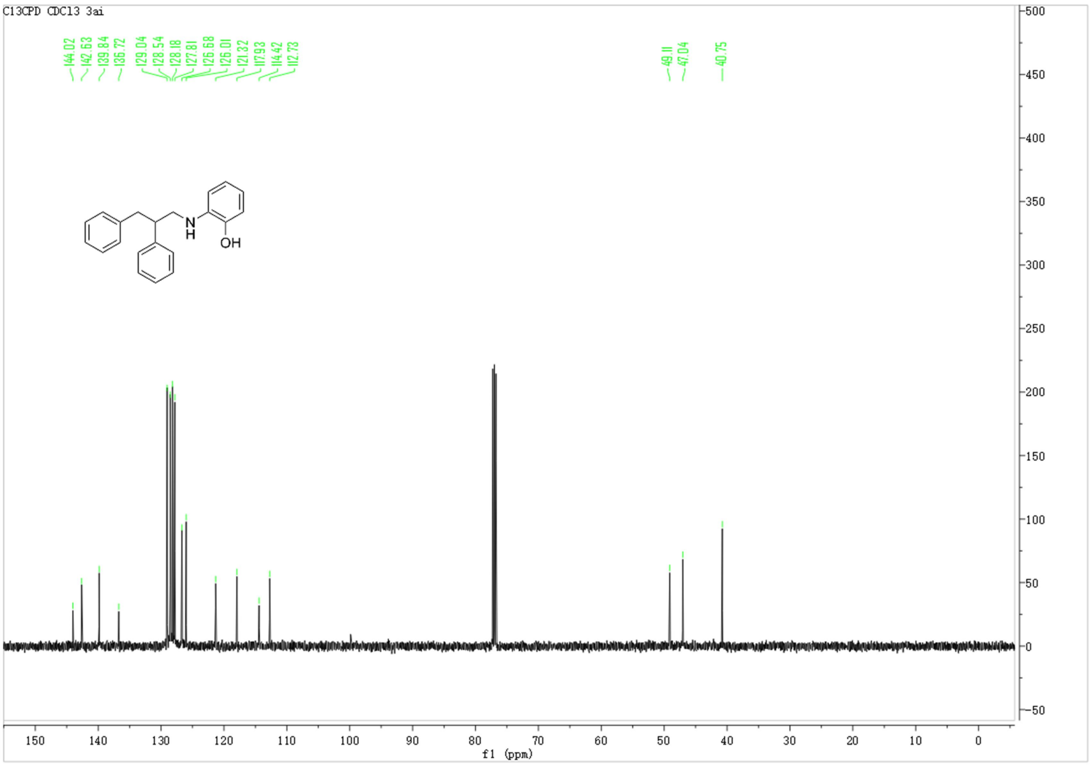

Supplementary Figure 203. ^{13}C NMR spectrum of **3ai** in $CDCl_3$.

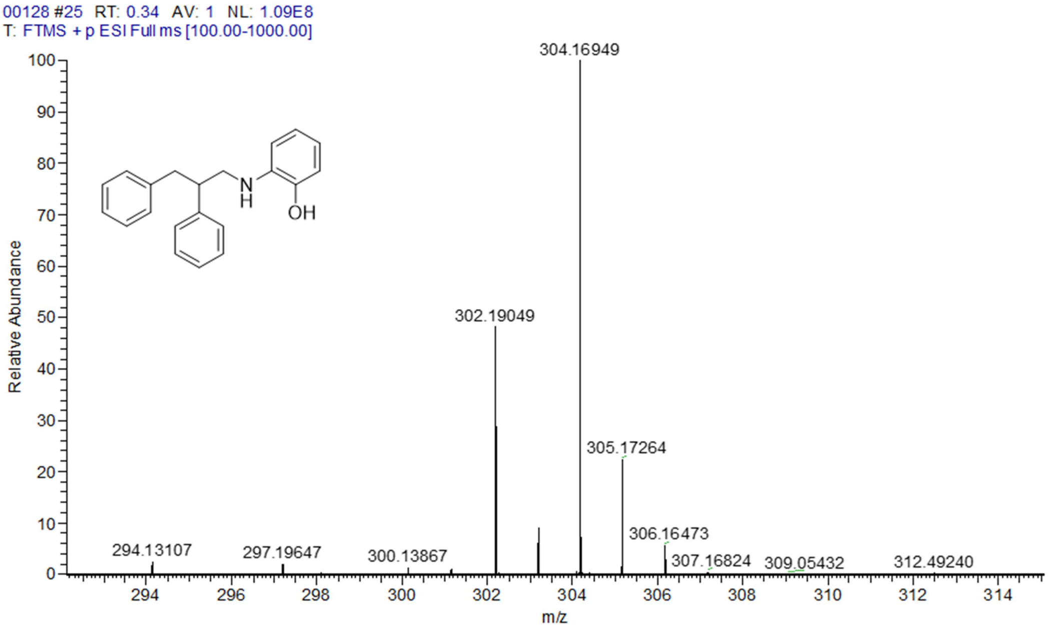

Supplementary Figure 204. HRMS of **3ai**.

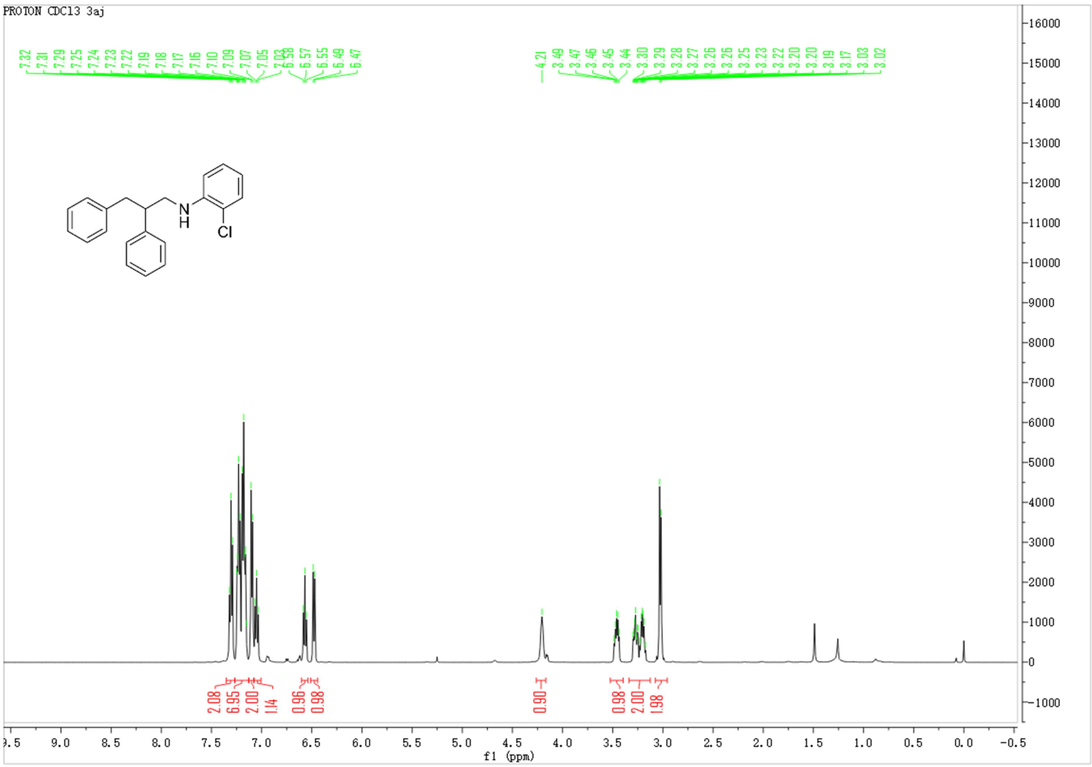

Supplementary Figure 205. ^1H NMR spectrum of **3aj** in $CDCl_3$.

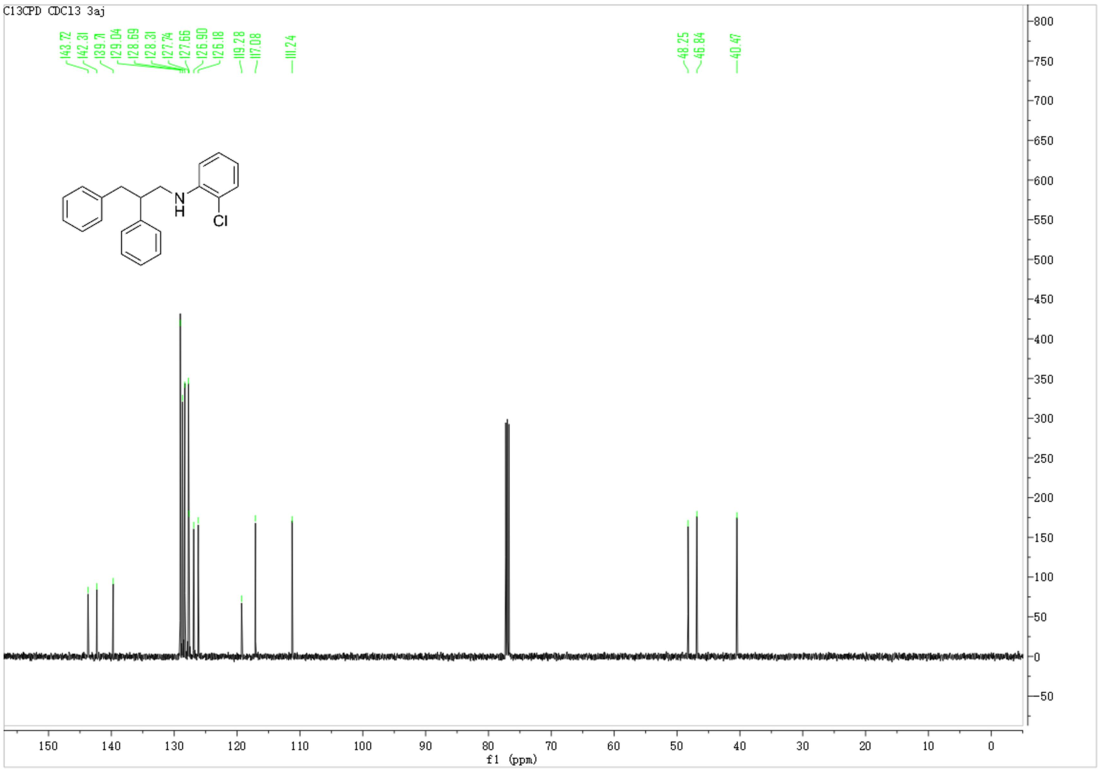

Supplementary Figure 206. ^{13}C NMR spectrum of **3aj** in $CDCl_3$.

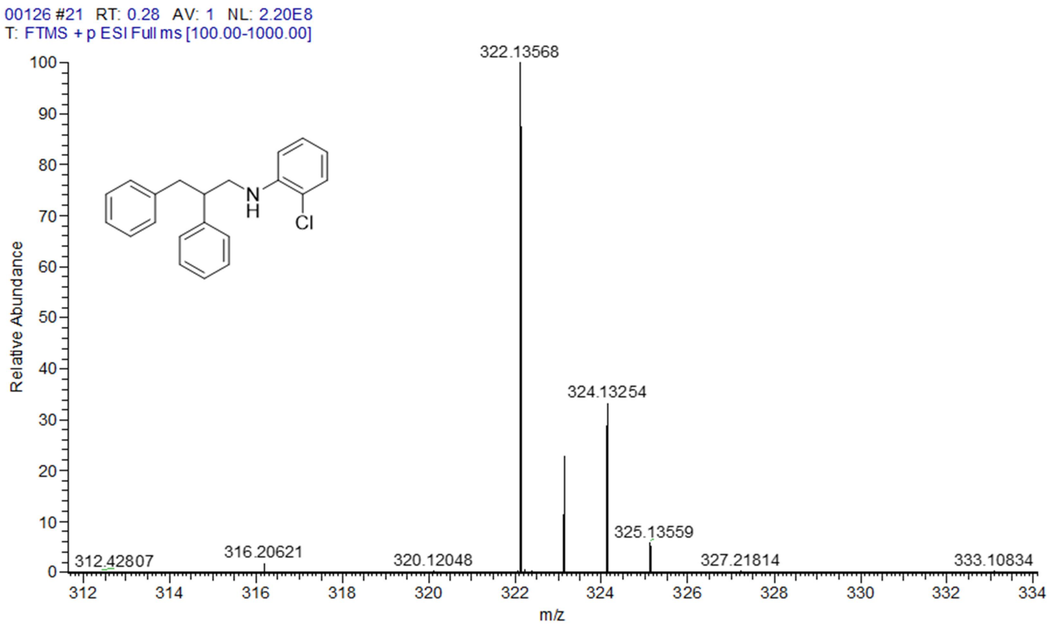

Supplementary Figure 207. HRMS of **3aj**.

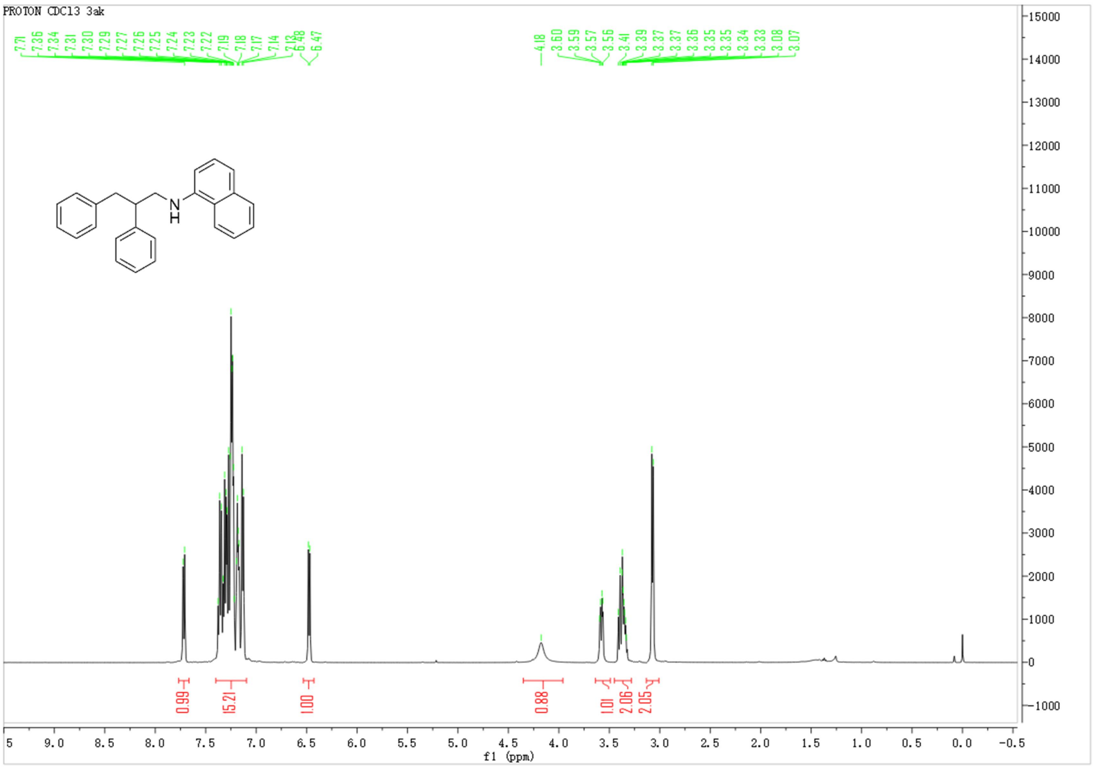

Supplementary Figure 208. ^{1}H NMR spectrum of **3ak** in $CDCl_3$.

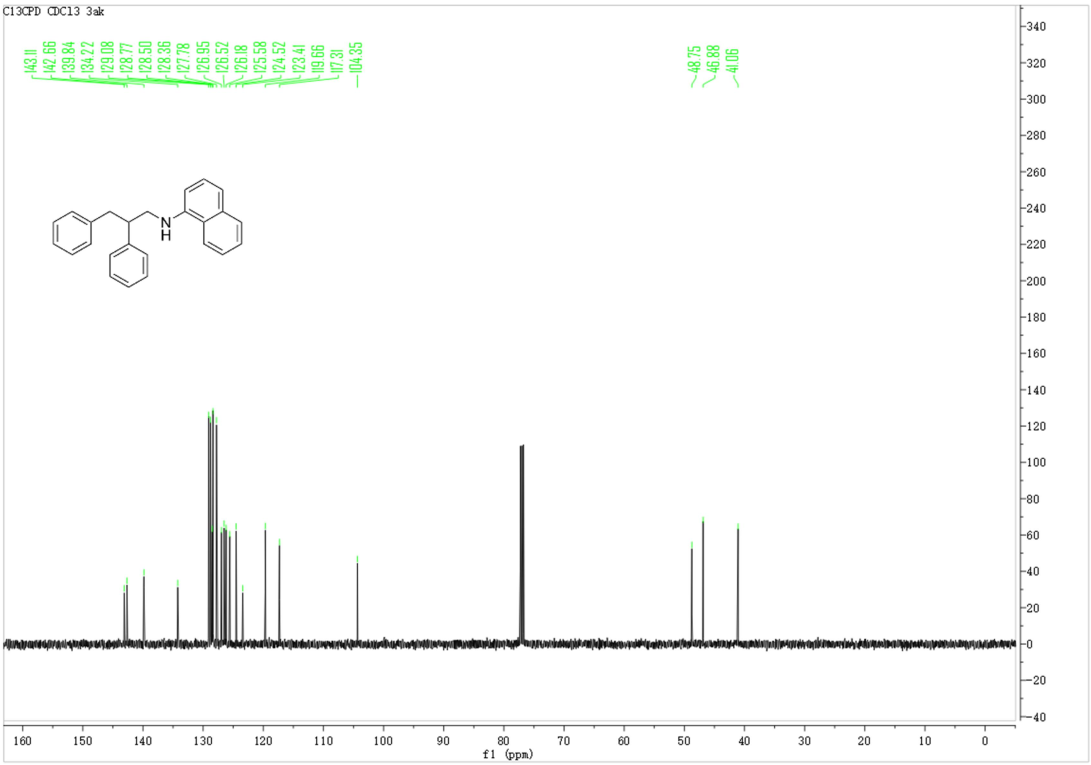

Supplementary Figure 209. ^{13}C NMR spectrum of **3ak** in $CDCl_3$.

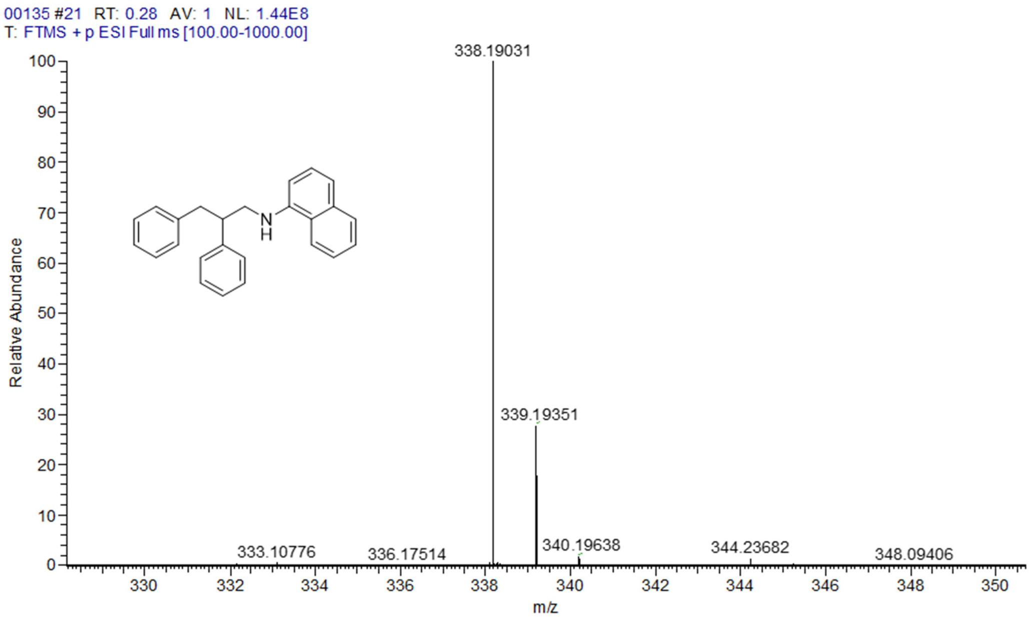

Supplementary Figure 210. HRMS of **3ak**.

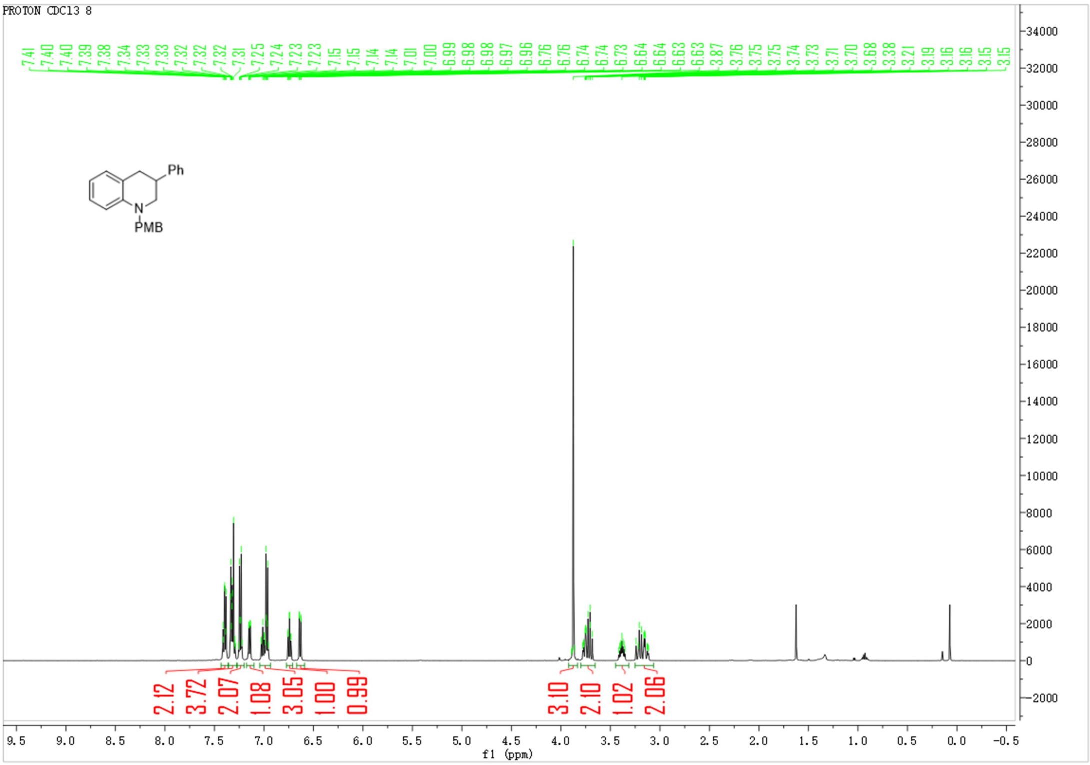

Supplementary Figure 211. ^{1}H NMR spectrum of **6** in $CDCl_3$.

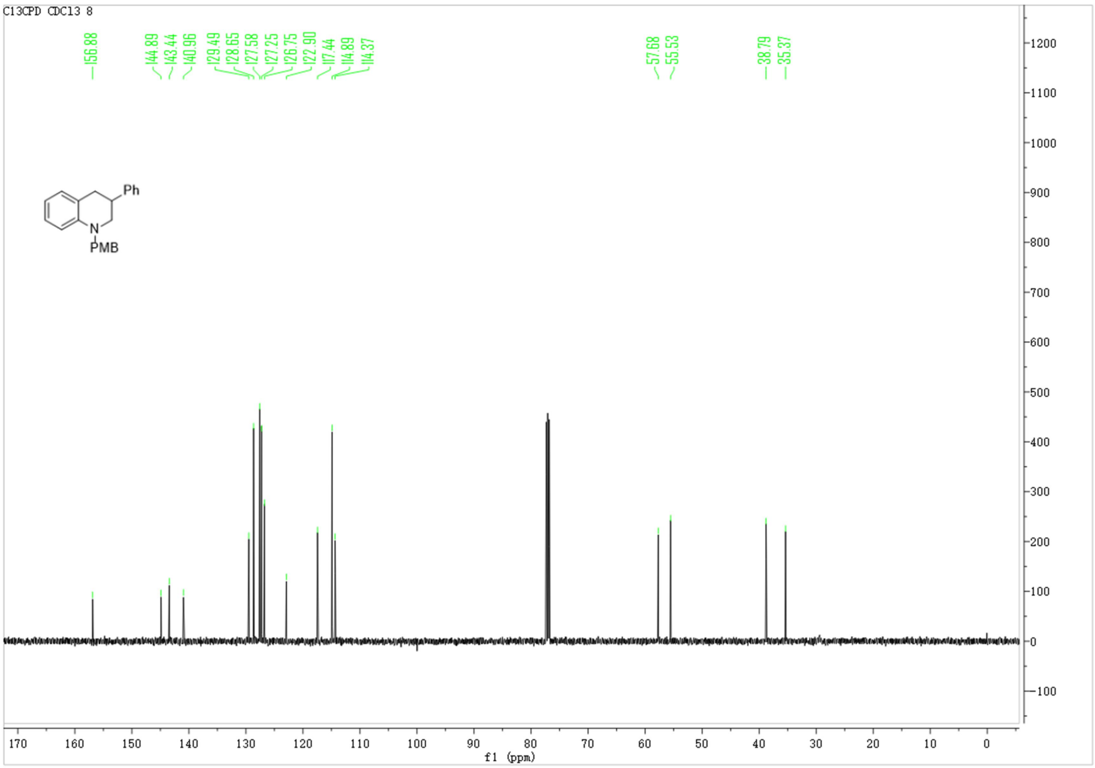

Supplementary Figure 212. ^{13}C NMR spectrum of **6** in $CDCl_3$.

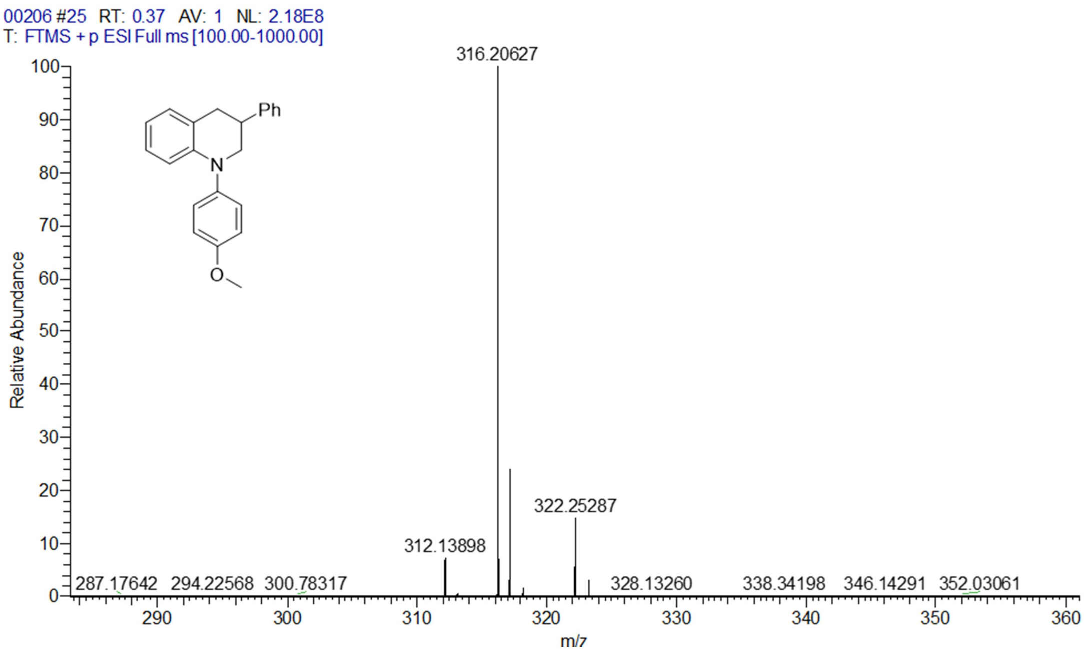

Supplementary Figure 213. HRMS of **6**.

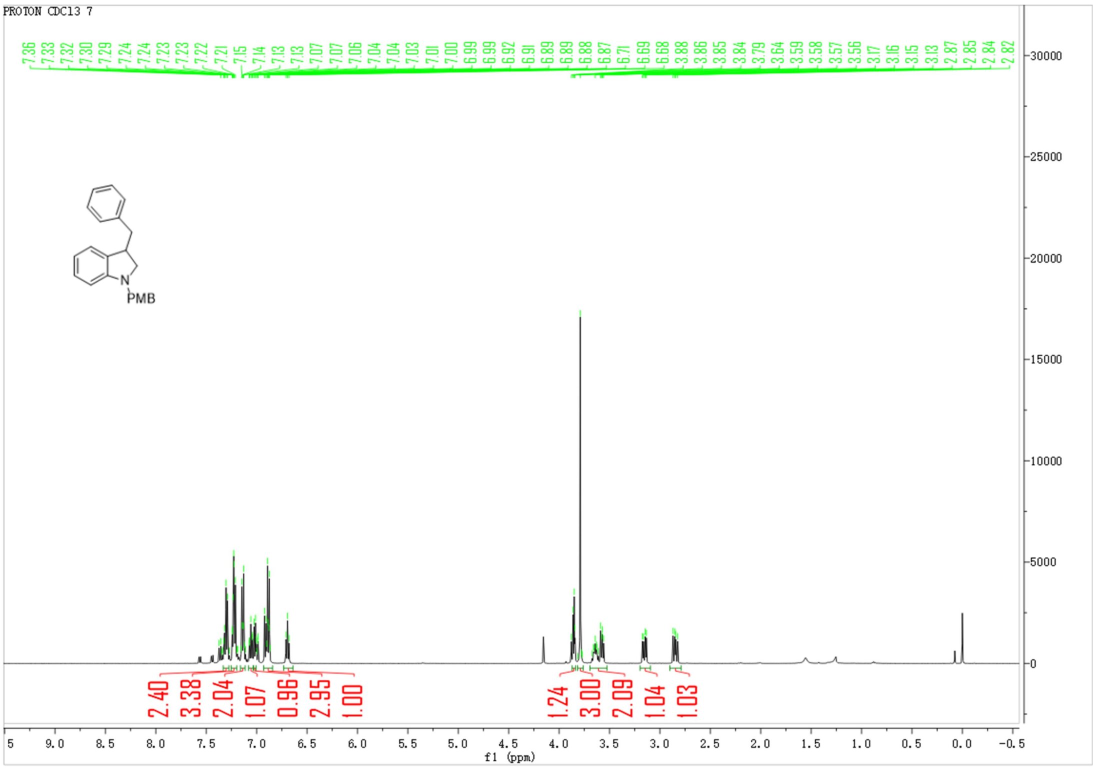

Supplementary Figure 214. ^{1}H NMR spectrum of **7** in $CDCl_3$.

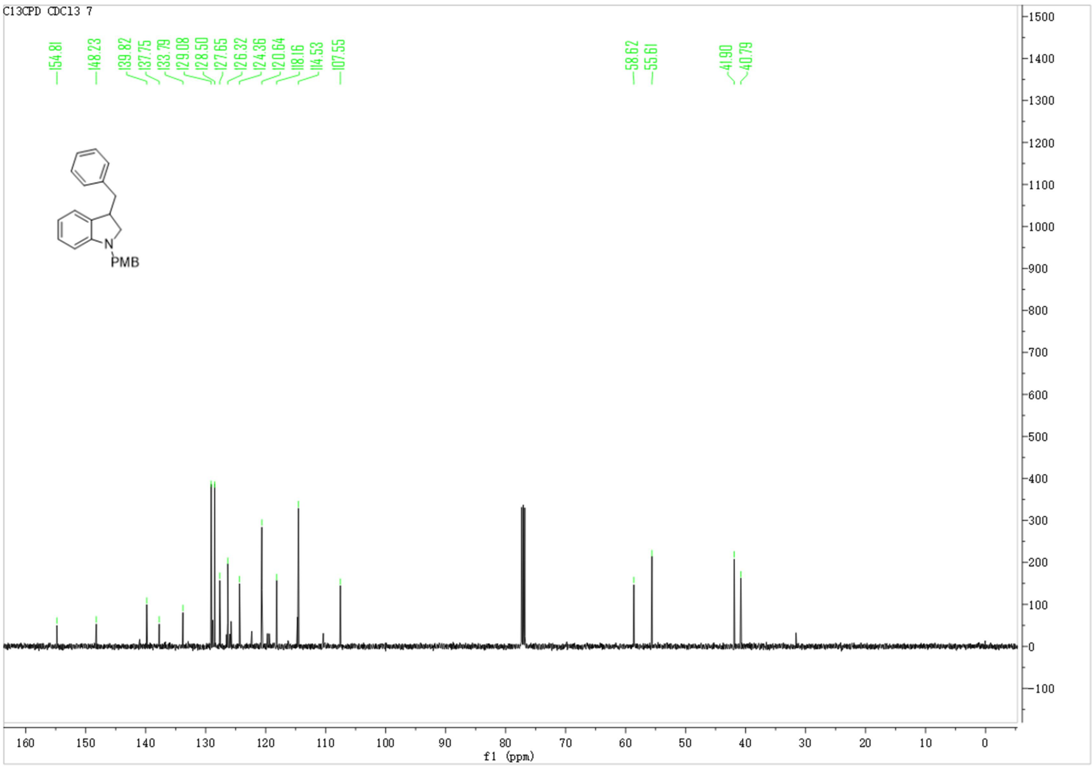

Supplementary Figure 215. ^{13}C NMR spectrum of **7** in $CDCl_3$.

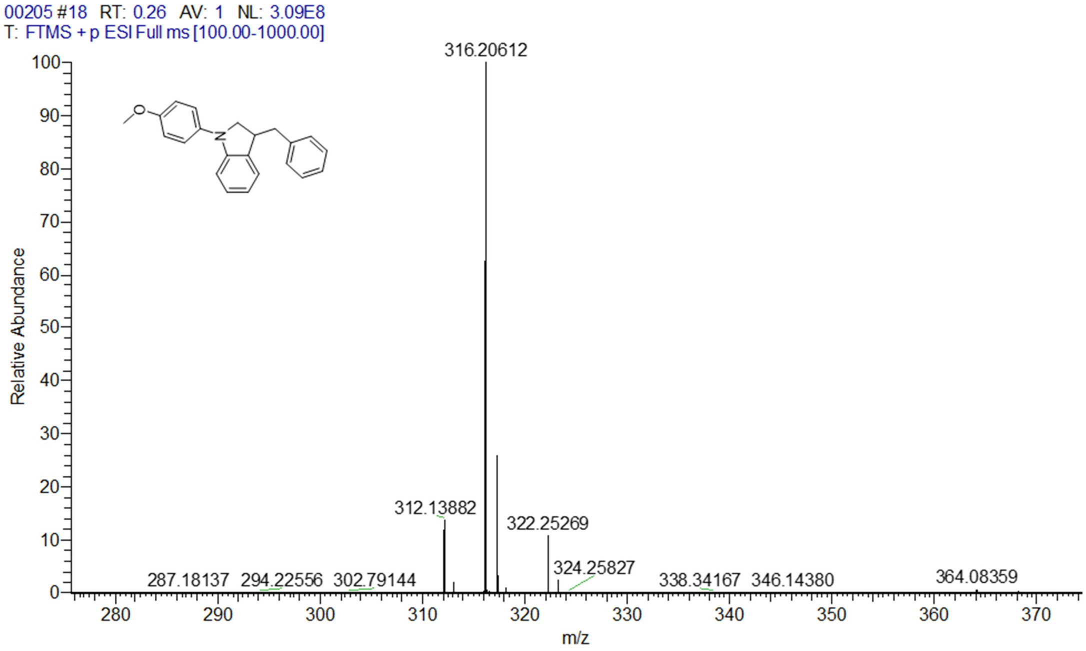

Supplementary Figure 216. HRMS of **7**.

Supplementary References:

1. C. B. Gilley, M. J. Buller and Y. Kobayashi. New entry to convertible isocyanides for the Ugi reaction and its application to the stereocontrolled formal total synthesis of the proteasome inhibitor omuralide. *Org. Lett.* **2007**, 9, 3631.
2. T. Matsuda, Y. Sakurai. Palladium–catalyzed ring–opening alkynylation of cyclopropenones. *Eur. J. Org. Chem*. **2013**, 20, 4219.
3. M. Larsson, H. E. Hogberg. Diastereoselective addition of methylmetal reagents to 2-methylaldehydes. *Tetrahedron* **2001**, *57,* 7541.
4. Parthasarathy Gandeepan, P. Rajamalli, Chien-Hong Cheng. Palladium-catalyzed dehydrogenative β-Arylation of simple saturated carbonyls by aryl halides. *ACS Catalysis* **2014**, 4(12), 4485-4489.
5. B. O. A. Tasch, L. Bensch, T. J. J. Müller. Masuda borylation–Suzuki coupling (MBSC) sequence of vinylhalides and its application in a one-pot synthesis of 3,4-biarylpyrazoles. *Org. Biomol. Chem*. **2013**, 11,6113-6118.
6. X.-L. Zhang, Y.-Q. Wang. Dehydrogenative β-Arylation of saturated aldehydes using transient directing groups. *Org. Lett.* **2019**, 21, 2731−2735.
7. X. Song, A. Song, W. Wang. Direct stereoselective α-arylation of unmodified enals using an organocatalytic cross-coupling-like reaction. *Nature commun.* **2011**, 2, 524.
8. J. Liu, X. Wen, C. Qin, N. Jiao. Oxygenation of simple olefins through selective allylic C−C bond cleavage: A direct approach to cinnamyl aldehydes. *Angew. Chem. Int. Ed.* **2017**, 56, 11940-11944.
9. N. Lahmar, J. Aatar, T. B. Ayed, M. Bellassoued. A general route to α-alkyl (E)-α,β-unsaturated aldehydes. *J. Organomet. Chem.* **2006**, 691, 3018-3026.
10. J.-H. Kim, R. J. Kulawiec. A tandem epoxide isomerization−aldol condensation process catalyzed by palladium acetate−tributylphosphine. *J. Org. Chem.* **1996**, 61, 7656-7657.
11. I. Choi, S. Chun, Y. K. Chung. Bimetallic Cobalt–Rhodium canoparticle-catalyzed reductive amination of aldehydes with nitroarenes under atmospheric hydrogen. *J. Org. Chem.* **2017**, 82, 12771-12777.
12. H. Doušová, R. Horák, Z. Růžičková. An intramolecular C–N cross-coupling of β-enaminones: a simple and efficient way to precursors of some alkaloids of Galipea officinalis. *Beilstein J. Org. Chem*. **2015**, 11, 884–892.
